# Cancer Incidence in Europe: An Ecological Analysis of Nutritional and Other Environmental Factors

**DOI:** 10.3389/fonc.2018.00151

**Published:** 2018-06-13

**Authors:** Pavel Grasgruber, Eduard Hrazdira, Martin Sebera, Tomas Kalina

**Affiliations:** Faculty of Sports Studies, Masaryk University, Brno, Czechia

**Keywords:** food consumption, nutrition, raised cholesterol, smoking, cancer, epidemiology, Europe

## Abstract

The aim of this work was to offer an ecological alternative to conventional observational studies and identify factors potentially associated with cancer incidence in Europe. The incidence of 24 types of cancer in 39 European countries (2012) was compared with a long-term mean supply of 68 food items from the FAOSTAT database (1993–2011) and some other variables such as smoking, body mass index, raised cholesterol, and socioeconomic indicators. In addition to simple Pearson linear correlations, the data were analyzed *via* factor analyses and penalized regression methods. This comparison identified two main groups of cancers that are characteristically associated with the same variables. The first group consists of cancers of the prostate, breast, white blood cells, and melanoma. Their incidence increases with rising gross domestic product (GDP) per capita, a prevalence of raised cholesterol and a high intake of animal products. The second group includes primarily cancers of the digestive tract and is most consistently correlated with alcoholic beverages, lard, and eggs. In addition, we found specific correlations between certain variables and some other types of cancer (smoking—lung and larynx cancer; low GDP per capita and high carbohydrate consumption—stomach and cervical cancer; tea drinking—esophageal cancer; maize consumption and wine drinking—liver cancer). The documented findings often remarkably agree with the current scientific consensus, and when combined with evidence based on different methodologies, they can further extend our knowledge of the etiology of cancer. In addition, our study also identifies several foods with possible preventive effects and indicates that various dairy products may markedly differ in their relationship to cancer incidence. All these data can potentially be of fundamental importance for clinical practice and the survival of cancer patients.

## Introduction

The examination of the relationship between nutrition and diseases is a very controversial topic, chiefly because most of the available data are based on long-term observational studies which rely on the self-reported consumption of selected food items. The reliability of such studies is, therefore, questionable and differs from food to food (1). Consequently, it is not surprising that these studies often produce conflicting results. Some scientists even regard this type of data as pseudoscientific and unusable (2). Interventional (clinical) studies, which prescribe a specific diet, are very demanding and time-limited, which is another serious weakness because the development of certain diseases may take many years.

Research on the dietary and other exogenous causes of cancer is particularly difficult because the development of cancer is a long-term process, and it cannot be examined in controlled clinical trials. The report of the WCRF [World Cancer Reasearch Fund] and the AICR [American Institute for Cancer Research] explicitly stated that “…it is difficult to identify single methodological approaches that can be seen as inherently superior. With a body of evidence comprising very different approaches, from observational epidemiology to basic science, and where the generalisability of clinical trials is limited, robust conclusions can only be drawn from a review of the totality of the relevant evidence, allowing for the advantages and disadvantages of different methods” (3).

The most reliable sources of knowledge are currently case–control studies (a type of observational studies that try to identify a causal factor between two groups with different health outcomes) that are supplemented by short interventional studies in patients with various grades of cancer progression.

Bad dietary habits are undoubtedly one of the main environmental factors involved in cancer development. They can directly contribute to an increased risk of cancer through carcinogens contained in food (e.g., alcohol or chemicals produced during excessive food processing), but diet can also influence hormonal and other metabolic processes in the body that promote cell growth (4). When taken together, the current evidence indicates that prostate cancer—the most frequent tumor in European men—is associated with red meat, dietary fat, and dairy products (5). Breast cancer—the most frequent cancer of European women—has been linked with red meat, alcoholic beverages, and high-glycemic load (6). The development of colorectal cancer is most consistently related to the consumption of (processed) red meat and alcoholic beverages (7).

In general, the accumulated evidence indicates that at least in middle-aged subjects, high blood levels of IGF1 (insulin-like growth factor), stimulated by the intake of high-quality proteins, are one of the fundamental physiological factors involved in cancer progression, including melanoma (8). Higher IGF1 levels are a prerequisite for greater physical growth, which explains why cancer mortality increases with increasing adult stature (9, 10). High cholesterol levels probably play an important role in cancer progression as well (6, 11, 12). Extremely high IGF1 levels can also explain the development of cancer in children (13), who suffer mainly from cancers of the brain and white blood cells (Hodgkin’s lymphoma, leukemia) (4).

However, the WCRF, which is regarded as one of the world’s most comprehensive sources of information on cancer prevention, still lists only few factors whose influence on the development of cancer is convincing, mostly body fatness, higher attained height, and alcohol (14). Because it is impossible to collect precise, long-term data on food consumption at the individual level, virtually the only possible way to overcome this problem is an ecological approach which compares official statistics of food intake and disease prevalence at the country level.

International statistics of food intake are available from the FAOSTAT database of food supply (15) and are defined as “the total quantity of foodstuffs produced in a country added to the total quantity imported and adjusted to any change in stocks that may have occurred since the beginning of the reference period”. These data, therefore, express food supply (food disappearance) in a country within a given year which must inevitably overestimate true food consumption because a certain proportion of food is wasted, consumed by foreigners, animals, etc. However, in our own research, we observed that the FAOSTAT statistics of annual per capita food supply produced very impressive results, especially in relation to the basic components of diet (fat, protein, carbohydrates). For example, the correlation between male height in 93 countries and four animal proteins of the highest quality reached *r* = 0.84 (*p* < 0.001) (16). In our subsequent study dealing with food consumption and the prevalence of cardiovascular diseases (CVDs) in 42 European countries (17), we found biologically relevant correlations reaching up to *r* = 0.92 (*p* < 0.001).

Therefore, although the data on food supply partially distort actual consumption, this limitation manifests in all countries in a similar way and the FAOSTAT database still reflects inter-country differences in per capita food consumption remarkably well. However, to our knowledge, only a few authors have recently tested such an inter-country comparison that included the FAOSTAT statistics and cancer incidence in Europe (18–21), and the number of examined food items and/or cancer types was still very limited. For example, the study of Grant (21) compared the incidence of 21 cancer types in 157 countries, but used only five items from the FAOSTAT database.

Although even strong findings based on such ecological studies cannot be regarded as definitive proof of causal relationships at the individual level, they can be used as a starting point of medical hypotheses and their validity can be supported by studies using different methodologies. The combination of different types of studies may, therefore, strengthen each other’s results which is of key importance in a complicated field such as dietology.

## Materials and Methods

### Aim, Design, and Working Hypotheses

Because the traditional flaw of ecological studies is the selection of a small number of variables that can be influenced by hidden confounding factors, the aim of the present study was to set the relationship between cancer incidence and exogenous risk factors into the widest possible context, using all the statistics that are available and potentially relevant—food supply from the FAOSTAT database, smoking and obesity rates, cholesterol levels, health expenditure, economic wealth, and the latest data on the incidence of cancer in Europe where we can expect the most accurate statistics. Our hypothesis was that we would find highly significant relationships between the incidence of cancer and some of these factors, and if they have a meaningful rationale and are supported by other studies, they could lead to the implementation of effective lifestyle guidelines aimed at cancer prevention and increased patient survival.

### Data Collection

The database FAOSTAT (food balance > food supply) (15) was accessed in October 2015 and an average daily supply of 58 food items (grams/day per capita) between 1993 and 2011 was obtained from all European countries included. Ten additional items or combinations [e.g., total fat and total protein, milk and vegetables, % energy from carbohydrates and alcohol (% CA energy), % energy from potato, cereal carbohydrates (% PC CARB energy), etc.] were specially computed by us, in order to show a more detailed relationship to certain health statistics. Because the FAOSTAT database lists only information on the average supply of food, protein, fat, and energy, the energy from carbohydrates was computed from the supply of protein and fat, assuming 4.1 kcal per gram of protein and 9 kcal per gram of fat. To examine the relationship between cancer and high dietary protein quality, we also added the “protein index” (the ratio between dairy and wheat proteins) which predicts male height in Europe (22). Altogether, 68 food items from the FAOSTAT database were included (see Data Sheet S1 in Supplementary Material, Sheet 1–2).

The estimated age-standardized rates of cancer incidence divided by sex (for 24 different types of cancer and total cancer incidence) in 40 European countries (for 2012) were found in the report of Ferlay et al. (23) (Data Sheet S1 in Supplementary Material, Sheet 3). These statistics are defined as projections of the most recent national rates available prior to 2012 (usually 2009 or 2010). In some cases, cancer incidence was estimated from cancer mortality, using the mean ratio between these variables from neighboring countries. Because both mortality and incidence for Montenegro was only estimated, we excluded Montenegro from the sample and reduced the number of countries to 39. Maps of cancer incidence in Europe from the EUCAN website (24), based on the same report, are listed in the Figures S1–S26 in Supplementary Material.

The list of independent variables was further supplemented by the statistics of smoking rates (a mean for 1990–2009), body mass index (BMI) (a mean for 1990–2008), and the prevalence of raised cholesterol (above >5.0 mmol/L, for 2008) from the report of Nichols et al. (25), which we used in our previous paper dealing with CVDs in Europe (17). The report also includes self-reported statistics of physical activity (the prevalence of insufficiently active adults aged 15 + years, for 2008), but these data are available for only 33 countries. In addition, we also took into consideration values of health expenditure per capita (for 1995–2011 and 2012), gross domestic product (GDP) per capita (1995–2012), and average life expectancy (2012) according to the World Bank (26) (Data Sheet S1 in Supplementary Material, Sheet 4).

### Statistical Analyses

Using the software SPSS Statistics 24.0, the relationships between cancer rates and the examined variables were first investigated using simple Pearson linear correlations. Subsequently, we conducted factor analyses that group variables according to certain similar characteristics (“factors”). Two-dimensional (or three-dimensional) plots of such factors can graphically visualize mutual relationships among a large number of variables which solves a whole range of problems associated with multicollinearity—the key statistical problem in the present study.

Another tools that we used for the reduction of multicollinearity are the ridge regression, LASSO [least absolute shrinkage and selection operator] regression and elastic net regression. These regression methods are aimed at identifying the best predictors out of a set of variables that are mutually correlated. They work with all independent variables at once and are based on the penalization (artificial lowering) of *beta* regression coefficients. The changing size of the penalization creates different models with different prediction errors, and a model with the lowest prediction error (ideally using low penalization) is selected as optimal. In the results of the ridge regression, all variables are ranked according to the size of their *beta* coefficients. The LASSO regression is more selective and with the increasing penalization, it shrinks *beta* coefficients in the majority of variables to 0. The elastic net regression is basically a combination of these two methods (27).

To improve the quality of regression models, we used cross-validation and the bootstrapping method. Cross-validation repeatedly tests the results on complementary subsets of samples and, subsequently, a mean of these tests is computed. Bootstrapping works with random combinations of independent variables with replacement, creates many additional models for each penalization level, and then also computes their mean result. This helps to eliminate various anomalies (see SPSS Statistics, http://ibm.com). For each regression treated *via* cross-validation and bootstrapping, two types of models were selected: “Optimal models” with the lowest prediction error, and “parsimonious (economical) models” that achieve the best balance between the prediction error and the number of selected predictors (or the number of tested variables for each penalization level in the ridge regression). Altogether, 12 regression models for each individual case of cancer were calculated, and the frequency of variables emerging among the top 5 with the highest absolute *beta* coefficients was counted.

Finally, we performed an analogy of fixed-effects models and examined temporal changes in the correlation between cancer incidence (2012) and food consumption in single years between 1993 and 2011. In some cases, food consumption between 1961 and 2011 was used, but only with a limited sample of 24 countries. Because there is usually a long delay between cancer onset and cancer detection, this procedure might identify a time period that was critical for the development of cancer. In addition, it could also reveal a long-term collinearity between some food items which would help in identifying confounding factors. On the other hand, some foodstuffs whose mean consumption rates are highly correlated may not show any close connection in the temporal comparison. This could indicate that their relationship to cancer incidence is in fact independent. The inter-item collinearity was examined *via* the regression slope test that compares the slope of two regression trend lines. The higher the probability value (*p*-value) in this test, the more two trend lines run parallel to each other (28). As a general rule, *p*-values above 0.05 were regarded as statistically significant.

## Results

Among the 24 types of cancer listed by Ferlay et al. (23), a mere four types (prostate, breast, colorectal, lung) are responsible for 51.6 and 48.9% of all listed cases in men and women, respectively. The total incidence rate in women is only 73% of that in men.

### Pearson Linear Correlations

The main results of Pearson linear correlations are presented in Tables 1 and 2 (for more detailed results, see the Data Sheet S1 in Supplementary Material, Sheet 5). Correlations of cancer with life expectancy are disparate and vary greatly, from *r* = −0.82 in men’s stomach cancer to *r* = 0.70 in breast cancer. Although our experience shows that short life expectancy (especially in men) may partly decrease age-standardized incidence of CVDs, these numbers show that it does not influence trends in cancer incidence rates in a significant way. Health expenditure and GDP do not correlate consistently with cancer incidence either, and their relationship to cancers that are subject to modern preventive programs (breast, colorectal, cervical) differs as well. However, there are distinct relationships between certain types of cancer and variables examined in this study. Several main groups can be distinguished.

**Table 1:** Correlations (*r*-values) between nine types of cancer and variables examined in this study (for more detailed results, see Data Sheet S1 in Supplementary Material, Sheet 5).

	Prostate	Testes	Breast	Melanoma		Hodgkin lymphoma		Non-Hodgkin lymphoma		Multiple myeloma		Leukemia		Cervix
									
	Men	Men	Women	Men	Women	Men	Women	Men	Women	Men	Women	Men	Women	Women
Fruits total	0.42	0.44	0.55	0.45	0.47	0.17	0.24	0.50	0.49	0.46	0.47	0.29	0.38	−0.65
Apples	0.28	0.30	0.26	0.34	0.40	−0.04	−0.01	0.35	0.35	0.25	0.26	0.03	0.23	−0.21
Bananas	0.67	0.61	0.57	0.65	0.68	0.14	0.23	0.67	0.70	0.60	0.49	0.30	0.32	−0.54
Grapes	−0.23	0.09	0.01	−0.04	−0.06	0.08	−0.30	−0.21	−0.22	−0.13	−0.21	−0.17	−0.16	−0.25
Oranges and mandarins	0.58	0.39	0.58	0.48	0.52	0.14	0.38	0.59	0.58	0.56	0.64	0.42	0.43	−0.58
Alcoholic beverages total	0.49	0.54	0.47	0.53	0.51	0.26	0.39	0.57	0.62	0.43	0.54	0.35	0.31	−0.15
Beer	0.42	0.46	0.40	0.51	0.49	0.19	0.23	0.45	0.53	0.36	0.43	0.32	0.24	−0.06
Distilled beverages	−0.16	−0.32	−0.45	−0.29	−0.32	−0.28	0.14	−0.30	−0.32	−0.33	−0.24	−0.06	0.00	0.38
Wine	0.30	0.42	0.40	0.27	0.28	0.28	0.43	0.50	0.48	0.34	0.43	0.17	0.23	−0.35
Cocoa beans	0.47	0.32	0.33	0.24	0.29	−0.16	0.09	0.28	0.22	0.33	0.24	0.18	0.09	−0.25
Coffee	0.71	0.54	0.62	0.69	0.69	0.04	0.21	0.65	0.65	0.55	0.54	0.18	0.30	−0.50
Tea	0.28	0.15	0.35	0.25	0.31	0.16	0.16	0.29	0.33	0.32	0.29	0.34	0.16	−0.16
Ref. sugar and sweeteners	0.45	0.42	0.51	0.41	0.52	0.23	0.37	0.40	0.45	0.38	0.39	0.38	0.30	−0.34
Refined sugar	0.42	0.29	0.45	0.38	0.49	0.36	0.28	0.39	0.39	0.43	0.34	0.38	0.27	−0.29
Oilcrops total	−0.21	−0.12	0.02	−0.20	−0.20	0.14	−0.06	−0.19	−0.17	−0.05	−0.07	0.21	0.29	−0.46
Olives	−0.32	−0.25	−0.17	−0.35	−0.35	0.05	−0.10	−0.35	−0.35	−0.21	−0.22	0.09	0.18	−0.34
Treenuts	0.17	0.20	0.28	0.21	0.26	0.45	0.24	0.26	0.23	0.33	0.25	0.27	0.36	−0.57
Plant oils total	0.22	0.24	0.30	0.25	0.23	0.44	0.30	0.37	0.35	0.36	0.42	0.36	0.39	−0.40
Olive oil	−0.09	−0.12	−0.03	−0.14	−0.13	0.24	0.21	0.05	−0.02	0.09	0.10	0.20	0.20	−0.33
Soybean oil	0.58	0.49	0.48	0.50	0.60	0.29	0.13	0.62	0.61	0.68	0.60	0.27	0.30	−0.32
Sunflower oil	−0.60	−0.33	−0.43	−0.48	−0.54	−0.10	−0.08	−0.49	−0.52	−0.52	−0.53	−0.30	−0.35	0.41
Cereals total	−0.59	−0.48	−0.62	−0.56	−0.59	−0.28	−0.27	−0.61	−0.62	−0.53	−0.44	−0.48	−0.45	0.48
Maize	−0.37	−0.28	−0.35	−0.30	−0.32	−0.33	−0.31	−0.34	−0.33	−0.38	−0.38	−0.60	−0.62	0.32
Rye	0.03	−0.22	−0.29	−0.06	−0.07	−0.02	0.01	−0.15	−0.19	−0.09	−0.01	0.12	0.19	0.22
Wheat	−0.47	−0.20	−0.25	−0.39	−0.41	−0.07	−0.08	−0.39	−0.37	−0.29	−0.22	−0.15	−0.15	0.17
Potatoes	−0.04	−0.36	−0.27	−0.18	−0.08	0.12	0.27	−0.12	−0.15	−0.05	0.02	0.17	0.11	0.26
Legumes total	−0.39	−0.22	−0.12	−0.31	−0.36	0.06	−0.01	−0.23	−0.24	−0.21	−0.27	−0.12	−0.13	0.04
Vegetables total	−0.56	−0.43	−0.28	−0.59	−0.56	−0.10	−0.01	−0.45	−0.48	−0.41	−0.41	−0.17	−0.13	−0.11
Onions	−0.75	−0.61	−0.57	0.69	−0.68	−0.24	−0.21	−0.67	−0.67	−0.69	−0.65	−0.34	−0.35	0.25
Tomatoes	−0.38	−0.28	−0.14	−0.38	−0.37	0.08	0.07	−0.25	−0.30	−0.20	−0.21	0.03	0.07	−0.24
Spices	−0.23	0.00	−0.21	−0.11	−0.13	−0.35	−0.23	−0.23	−0.22	−0.26	−0.34	−0.43	−0.43	0.14
Plant protein	−0.54	−0.49	−0.47	−0.56	−0.56	−0.26	−0.16	−0.50	−0.54	−0.40	−0.39	−0.36	−0.36	0.24
Plant fat	0.24	0.24	0.35	0.22	0.23	0.36	0.28	0.37	0.34	0.39	0.39	0.39	0.39	−0.52
Meat total	0.53	0.46	0.59	0.44	0.47	0.19	0.36	0.63	0.63	0.59	0.59	0.58	0.57	−0.44
Beef	0.56	0.36	0.53	0.49	0.54	0.29	0.48	0.53	0.48	0.53	0.55	0.40	0.50	−0.49
Pork	0.31	0.42	0.32	0.36	0.31	0.19	0.25	0.42	0.48	0.34	0.42	0.39	0.45	−0.20
Poultry	0.30	0.22	0.40	0.15	0.21	0.09	0.14	0.44	0.44	0.37	0.35	0.47	0.37	−0.22
Meat protein	0.55	0.42	0.61	0.43	0.49	0.17	0.35	0.65	0.64	0.61	0.59	0.55	0.52	−0.48
Meat fat	0.64	0.45	0.62	0.58	0.54	0.21	0.37	0.72	0.69	0.63	0.59	0.47	0.43	−0.47
Beef and pork fat	0.60	0.45	0.55	0.63	0.55	0.28	0.40	0.68	0.68	0.57	0.57	0.38	0.42	−0.41
Dairy total (excl. butter)	0.66	0.36	0.50	0.63	0.65	0.27	0.12	0.58	0.54	0.58	0.50	0.25	0.39	−0.47
Milk	−0.21	−0.38	−0.26	−0.26	−0.25	−0.17	−0.15	−0.27	−0.26	−0.27	−0.29	−0.18	−0.12	0.22
Cheese	0.61	0.52	0.61	0.54	0.59	0.28	0.26	0.59	0.55	0.67	0.59	0.40	0.41	−0.58
Dairy protein	0.64	0.28	0.50	0.51	0.57	0.11	0.07	0.48	0.43	0.52	0.40	0.23	0.36	−0.43
Dairy fat	0.45	0.18	0.43	0.31	0.37	0.09	0.07	0.36	0.33	0.44	0.33	0.24	0.26	−0.45
Milk protein	−0.18	−0.36	−0.25	−0.28	−0.24	−0.23	−0.22	−0.27	−0.27	−0.25	−0.26	−0.17	−0.12	0.09
Milk fat	−0.17	−0.32	−0.22	−0.26	−0.23	−0.23	−0.27	−0.24	−0.24	−0.21	−0.25	−0.12	−0.09	0.05
Butter and ghee	0.60	0.24	0.48	0.39	0.41	0.26	0.18	0.46	0.38	0.52	0.45	0.37	0.27	−0.34
Edible offals	0.33	0.12	0.26	0.09	0.20	0.00	0.21	0.23	0.18	0.21	0.27	0.28	0.16	0.06
Fish and seafood	0.55	0.10	0.39	0.22	0.34	0.01	0.26	0.54	0.44	0.51	0.38	0.26	0.17	−0.35
Fish and seafood fat	0.68	0.25	0.45	0.41	0.49	0.11	0.23	0.60	0.53	0.63	0.52	0.29	0.25	−0.35
Eggs total	0.19	0.28	0.22	0.23	0.29	0.13	0.30	0.23	0.24	0.21	0.26	0.27	0.31	−0.02
Lard	0.13	0.57	0.27	0.26	0.28	0.12	0.17	0.27	0.35	0.15	0.22	0.15	0.17	0.02
Honey	0.04	0.06	−0.06	0.14	0.11	0.19	0.02	−0.01	−0.02	0.06	0.01	0.13	0.23	−0.28
Animal protein	0.75	0.38	0.66	0.52	0.62	0.14	0.31	0.72	0.66	0.71	0.62	0.48	0.48	−0.50
Animal fat	0.77	0.64	0.75	0.67	0.71	0.26	0.34	0.77	0.75	0.71	0.67	0.49	0.46	−0.47
Anim. fat and anim. protein	0.80	0.55	0.75	0.63	0.70	0.22	0.35	0.79	0.75	0.74	0.68	0.51	0.49	−0.51
Total protein	0.55	0.18	0.48	0.29	0.40	0.03	0.26	0.54	0.45	0.57	0.48	0.35	0.35	−0.42
Total fat	0.64	0.56	0.69	0.56	0.59	0.37	0.39	0.72	0.69	0.69	0.66	0.54	0.52	−0.60
Total fat and tot. protein	0.66	0.47	0.67	0.51	0.57	0.28	0.37	0.71	0.66	0.70	0.65	0.52	0.50	−0.59
% CA energy	−0.65	−0.56	−0.71	−0.58	−0.60	−0.31	−0.30	−0.71	−0.68	−0.68	−0.61	−0.55	−0.53	0.61
% PC CARB energy	−0.68	−0.61	−0.76	−0.62	−0.66	−0.30	−0.35	−0.72	−0.72	−0.66	−0.58	−0.56	−0.54	0.60
% Plant food energy	−0.79	−0.61	−0.70	−0.69	−0.71	−0.16	−0.31	−0.77	−0.76	−0.66	−0.61	−0.47	−0.46	0.36
Total energy	0.51	0.32	0.48	0.36	0.43	0.25	0.41	0.55	0.51	0.56	0.57	0.42	0.40	−0.42
Vegetables and cereals	−0.66	−0.52	−0.48	−0.67	−0.66	−0.20	−0.14	−0.60	−0.62	−0.53	−0.49	−0.34	−0.30	0.14
Milk and vegetables	−0.53	−0.55	−0.37	−0.58	−0.56	−0.19	−0.11	−0.49	−0.51	−0.47	−0.48	−0.24	−0.17	0.07
Milk and veget. and cereals	−0.62	−0.60	−0.51	−0.66	−0.65	−0.25	−0.18	−0.60	−0.62	−0.55	−0.53	−0.35	−0.30	0.23
Protein index	0.69	0.30	0.48	0.59	0.65	0.14	0.12	0.53	0.49	0.49	0.40	0.23	0.37	−0.38
Smoking (men)	−0.69	−0.60		−0.73		−0.19		−0.78		−0.68		−0.27		
Smoking (women)			0.38		0.36		0.12		0.22		0.18		−0.04	−0.29
Raised cholesterol	0.76	0.61		0.59		0.20		0.78		0.76		0.58		
Raised cholesterol (women)			0.76		0.63		0.40		0.76		0.71		0.51	−0.43
GDP Per Capita	0.74	0.61	0.72	0.70	0.73	0.17	0.36	0.75	0.77	0.67	0.72	0.45	0.48	−0.61

*% CA energy, the proportion of energy from carbohydrates and alcohol (as % of total energy intake); % PC CARB energy, the proportion of energy from potato and cereal carbohydrates (as % of total energy intake); Protein index, indicator of dietary protein quality (the ratio between proteins from dairy and wheat); GDP per capita, gross domestic product per capita*.

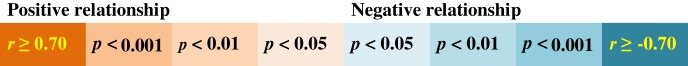

#### Cancers Correlated With High GDP Per Capita, Animal Fat and Animal Protein, and Raised Cholesterol

**Figure 1:**
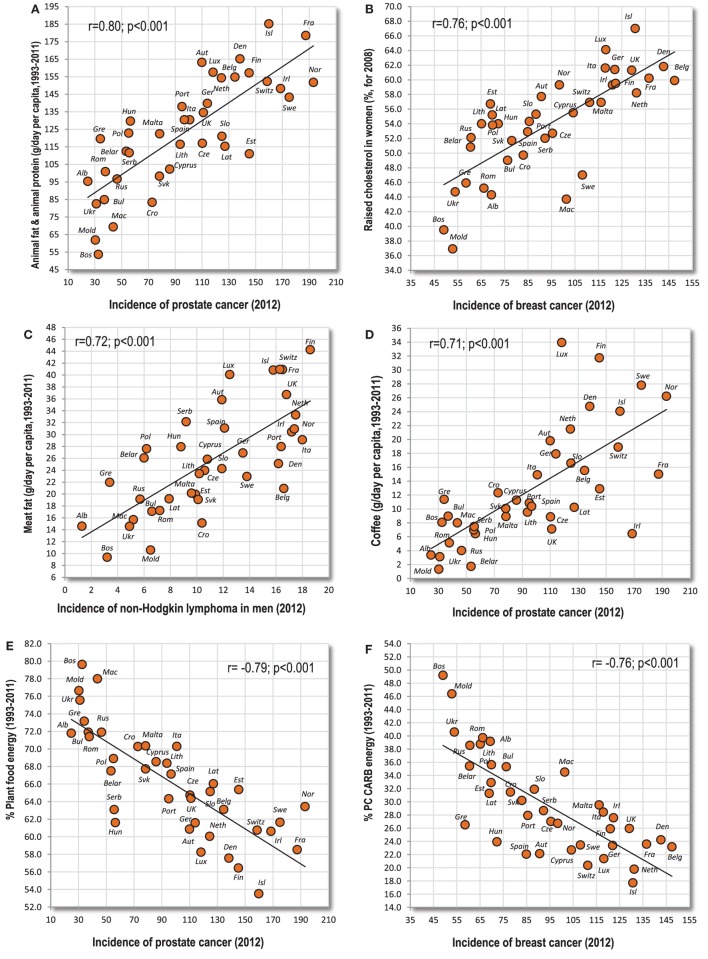
**(A–F)** Relationship between major independent variables and cancers of the prostate, breast, and white blood cells (non-Hodgkin lymphoma).

This first group consists mainly of cancers of the prostate, breast, white blood cells [especially non-Hodgkin lymphoma (NHL), but only partly Hodgkin lymphoma], melanoma, and largely even the cancer of testes (Table 1). These cancers strongly correlate with GDP per capita and their incidence is the highest in the nations of Northwestern and Northern Europe (e.g., Ireland, Switzerland, and France). They are typically associated with the high consumption of animal fat, and particularly with the combination of animal fat and animal protein which correlates exceptionally strongly with prostate cancer (*r* = 0.80, *p* < 0.001) (Figure 1A) and NHL in men (*r* = 0.79, *p* < 0.001). Raised cholesterol is another prominent factor which is not surprising since animal fat and animal protein is the strongest predictor of raised cholesterol in our comparison (*r* = 0.89 in men, *r* = 0.84 in women; *p* < 0.001). Raised cholesterol is strongly connected especially with men’s NHL (*r* = 0.78, *p* < 0.001), and it emerges as the strongest positive correlate in several other types of cancer, including breast cancer (Figure 1B). Values over *r* > 0.70 were found even between meat fat and men’s NHL (Figure 1C), and between coffee and prostate cancer (Figure 1D). Most of these cancers also correlate significantly with high dietary protein quality (the “protein index”), especially prostate cancer (*r* = 0.69) and melanoma (*r* = 0.59 in men, *r* = 0.65 in women; *p* < 0.001).

Variables with the strongest negative correlations are the proportion of plant food energy (*r* = −0.79, *p* < 0.001 with prostate cancer) (Figure 1E), % PC CARB energy (*r* = −0.76, *p* < 0.001 with breast cancer) (Figure 1F), onions (*r* = −0.75, *p* < 0.001 with prostate cancer), % CA energy (*r* = −0.71, *p* < 0.001 with breast cancer), and smoking in men (*r* = −0.78, *p* < 0.001 with men’s NHL).

#### Cancers Correlated With Alcoholic Beverages, Lard and Eggs

This second group is most typical for Central Europe and neighboring areas (the Czech Republic, Slovakia, Hungary, etc.), and primarily includes cancers of the digestive system. These cancers tend to be most strongly and most consistently associated with alcohol, lard, and eggs. Only stomach cancer is a striking exception because its relationship to many variables is completely opposite (Table 2). Some of these cancers also correlate quite strongly with animal fat and partly even with the “protein index” (oral cavity and pharynx in women, esophageal cancer), and hence they stand somewhere midway between the first and second group. Something similar applies even to testicular cancer. The individual correlation coefficients are weaker than in the first group, but this could be ascribed to the combined role of multiple factors.

**Table 2:** Correlations (*r*-values) between seven types of cancer, total cancer incidence, and variables examined in this study (for more detailed results, see Data Sheet S1 in Supplementary Material, Sheet 5).

	Esophagus		Colorectal		Pancreas		Kidney		Bladder		Stomach		Lung		Cancer total	
								
	Men	Women	Men	Women	Men	Women	Men	Women	Men	Women	Men	Women	Men	Women	Men	Women
Fruits total	−0.01	0.29	0.09	0.22	−0.29	0.11	−0.17	−0.23	0.23	0.26	−0.56	−0.45	−0.34	0.36	0.25	0.40
Apples	0.31	0.18	0.19	0.20	0.04	0.24	0.13	0.10	0.18	0.29	−0.14	−0.17	−0.07	0.28	0.33	0.31
Bananas	0.21	0.37	0.31	0.49	−0.17	0.34	0.13	0.18	0.35	0.56	−0.58	−0.48	−0.60	0.37	0.45	0.56
Grapes	−0.43	−0.18	−0.21	−0.18	0.05	−0.04	−0.34	−0.32	0.07	0.03	0.08	0.18	0.10	0.04	−0.22	−0.09
Oranges and mandarins	0.16	0.47	0.14	0.36	−0.34	0.09	−0.04	−0.09	0.09	0.19	−0.61	−0.53	−0.45	0.37	0.33	0.48
Alcoholic beverages total	0.53	0.53	0.60	0.42	0.19	0.51	0.42	0.40	0.18	0.41	−0.42	−0.42	−0.06	0.45	0.64	0.57
Beer	0.54	0.56	0.56	0.40	0.26	0.53	0.46	0.52	0.11	0.43	−0.36	−0.36	−0.05	0.45	0.55	0.55
Distilled beverages	0.15	−0.15	−0.08	−0.26	0.04	−0.26	0.26	0.25	−0.38	−0.29	0.40	0.37	0.13	−0.33	−0.15	−0.34
Wine	0.10	0.12	0.30	0.24	−0.13	0.17	−0.06	−0.28	0.32	0.17	−0.37	−0.36	−0.08	0.20	0.40	0.29
Cocoa beans	0.01	0.17	0.16	0.31	0.03	0.24	0.17	0.11	0.13	0.22	−0.31	−0.26	−0.16	0.29	0.36	0.35
Coffee	0.10	0.31	0.15	0.41	−0.15	0.32	0.03	0.07	0.21	0.37	−0.62	−0.55	−0.47	0.42	0.45	0.59
Tea	0.55	0.74	0.10	0.22	−0.04	0.14	0.06	0.21	−0.26	0.07	−0.18	−0.19	−0.21	0.32	0.15	0.34
Ref. sugar and sweeteners	0.51	0.44	0.29	0.47	0.06	0.24	0.29	0.31	0.29	0.49	−0.26	−0.28	−0.17	0.37	0.46	0.56
Refined sugar	0.45	0.39	0.23	0.46	0.06	0.19	0.21	0.28	0.30	0.32	−0.25	−0.28	−0.15	0.27	0.40	0.45
Oilcrops total	−0.36	−0.10	−0.37	−0.35	−0.26	−0.25	−0.36	−0.40	0.22	−0.05	−0.20	−0.07	−0.17	−0.13	−0.38	−0.25
Olives	−0.47	−0.26	−0.51	−0.51	−0.27	−0.34	−0.35	−0.43	0.10	−0.12	−0.02	0.11	−0.09	−0.21	−0.48	−0.41
Treenuts	−0.11	0.04	−0.06	0.01	−0.27	−0.03	−0.23	−0.30	0.42	0.11	−0.41	−0.34	−0.19	0.04	0.07	0.04
Plant oils total	0.07	0.13	0.26	0.17	−0.12	0.10	0.00	−0.13	0.39	0.07	−0.42	−0.39	−0.02	0.08	0.28	0.16
Olive oil	−0.30	−0.21	−0.13	−0.16	−0.22	−0.15	−0.17	−0.32	0.25	−0.14	−0.19	−0.15	−0.05	−0.19	−0.13	−0.23
Soybean oil	0.32	0.33	0.38	0.61	−0.18	0.21	0.26	0.25	0.40	0.42	−0.38	−0.39	−0.22	0.36	0.55	0.55
Sunflower oil	−0.27	−0.43	−0.09	−0.28	0.17	−0.25	−0.30	−0.41	−0.03	−0.35	0.41	0.37	0.53	−0.31	−0.32	−0.46
Cereals total	−0.43	−0.44	−0.37	−0.51	0.16	−0.22	−0.13	−0.16	−0.28	−0.33	0.52	0.48	0.21	−0.49	−0.52	−0.58
Maize	−0.33	−0.24	−0.19	−0.26	−0.05	−0.13	−0.39	−0.36	−0.32	−0.32	0.09	0.03	0.06	−0.20	−0.41	−0.38
Rye	0.17	−0.16	−0.07	−0.11	0.17	−0.03	0.45	0.52	−0.18	−0.14	0.42	0.30	0.12	−0.18	0.07	−0.10
Wheat	−0.36	−0.24	−0.21	−0.32	0.13	−0.14	−0.09	−0.19	0.06	−0.01	0.26	0.34	0.17	−0.27	−0.34	−0.28
Potatoes	0.51	0.14	−0.01	−0.05	0.04	−0.21	0.36	0.43	−0.30	−0.30	0.44	0.26	0.24	−0.19	0.06	−0.15
Legumes total	−0.40	−0.20	−0.09	−0.26	−0.06	−0.28	−0.28	−0.34	0.16	−0.06	0.00	0.10	0.23	−0.17	−0.31	−0.26
Vegetables total	−0.54	−0.41	−0.45	−0.56	−0.18	−0.46	−0.50	−0.59	0.21	−0.24	0.14	0.20	0.15	−0.40	−0.57	−0.57
Onions	−0.30	−0.41	−0.32	−0.55	0.10	−0.34	−0.28	−0.29	−0.11	−0.37	0.52	0.45	0.35	−0.38	−0.59	−0.64
Tomatoes	−0.49	−0.34	−0.36	−0.38	−0.16	−0.29	−0.46	−0.57	0.22	−0.21	−0.01	0.04	0.01	−0.32	−0.43	−0.43
Spices	−0.15	−0.04	−0.06	−0.14	−0.11	−0.17	−0.21	−0.20	−0.10	−0.02	−0.10	−0.06	0.21	0.11	−0.22	−0.20
Plant protein	−0.39	−0.31	−0.47	−0.58	−0.03	−0.34	−0.21	−0.21	−0.18	−0.30	0.34	0.35	0.15	−0.41	−0.56	−0.56
Plant fat	0.02	0.12	0.18	0.14	−0.21	0.04	−0.06	−0.17	0.43	0.08	−0.47	−0.42	−0.10	0.08	0.23	0.15
Meat total	0.35	0.40	0.45	0.43	−0.07	0.38	0.22	0.14	0.41	0.36	−0.54	−0.54	−0.17	0.41	0.56	0.53
Beef	0.26	0.44	0.07	0.26	−0.22	0.14	0.11	0.10	0.04	0.16	−0.29	−0.24	−0.28	0.25	0.41	0.46
Pork	0.25	0.15	0.56	0.33	0.19	0.47	0.28	0.23	0.43	0.36	−0.39	−0.43	0.03	0.29	0.50	0.37
Poultry	0.35	0.40	0.41	0.36	0.01	0.21	0.10	0.01	0.29	0.10	−0.29	−0.33	−0.03	0.24	0.39	0.33
Meat protein	0.37	0.44	0.37	0.41	−0.15	0.30	0.15	0.10	0.34	0.30	−0.54	−0.53	−0.24	0.42	0.51	0.53
Meat fat	0.40	0.52	0.20	0.31	−0.17	0.32	0.12	0.11	0.06	0.20	−0.57	−0.52	−0.29	0.48	0.49	0.56
Beef and pork fat	0.38	0.45	0.22	0.28	−0.06	0.37	0.16	0.18	0.01	0.16	−0.49	−0.46	−0.24	0.41	0.49	0.52
Dairy total (excl. butter)	0.25	0.48	−0.11	0.15	−0.28	0.15	0.05	0.11	−0.08	0.22	−0.49	−0.37	−0.50	0.39	0.32	0.42
Milk	0.02	0.11	−0.45	−0.43	−0.23	−0.33	−0.23	−0.20	−0.50	−0.26	0.22	0.31	−0.10	−0.11	−0.40	−0.32
Cheese	0.17	0.36	0.14	0.34	−0.14	0.34	0.15	0.13	0.29	0.38	−0.67	−0.60	−0.33	0.43	0.44	0.53
Dairy protein	0.28	0.47	−0.17	0.11	−0.30	0.08	0.09	0.13	−0.06	0.26	−0.44	−0.31	−0.45	0.40	0.29	0.41
Dairy fat	0.12	0.47	−0.31	−0.02	−0.43	−0.02	−0.17	−0.15	−0.10	0.14	−0.51	−0.35	−0.47	0.36	0.06	0.25
Milk protein	0.00	0.11	−0.51	−0.44	−0.23	−0.35	−0.24	−0.21	−0.44	−0.28	0.24	0.33	−0.21	−0.16	−0.42	−0.36
Milk fat	−0.04	0.11	−0.53	−0.42	−0.25	−0.33	−0.31	−0.27	−0.39	−0.25	0.18	0.27	−0.27	−0.13	−0.45	−0.34
Butter and ghee	0.36	0.34	0.03	0.15	−0.04	0.16	0.30	0.39	0.05	0.03	−0.23	−0.21	−0.17	0.20	0.46	0.40
Edible offals	0.27	0.36	0.06	0.19	−0.06	0.07	0.16	0.08	−0.05	0.08	0.05	−0.02	−0.06	0.20	0.32	0.32
Fish and seafood	0.22	0.21	−0.03	0.29	−0.37	−0.07	0.05	0.03	0.20	0.19	−0.32	−0.30	−0.44	0.16	0.28	0.30
Fish and seafood fat	0.21	0.27	0.04	0.44	−0.26	0.09	0.12	0.14	0.26	0.31	−0.39	−0.39	−0.46	0.24	0.41	0.44
Eggs total	0.43	0.15	0.54	0.51	0.39	0.37	0.47	0.40	0.29	0.19	−0.01	−0.13	0.23	0.17	0.51	0.36
Lard	0.32	0.16	0.66	0.51	0.30	0.45	0.20	0.12	0.55	0.65	−0.13	−0.15	0.29	0.44	0.53	0.45
Honey	−0.09	−0.09	−0.18	−0.23	−0.14	0.01	−0.13	−0.17	0.05	−0.11	−0.19	−0.17	−0.24	−0.11	−0.10	−0.19
Animal protein	0.41	0.51	0.15	0.40	−0.29	0.20	0.18	0.16	0.24	0.36	−0.54	−0.50	−0.42	0.46	0.53	0.58
Animal fat	0.49	0.61	0.32	0.51	−0.09	0.44	0.20	0.20	0.29	0.51	−0.59	−0.54	−0.26	0.66	0.69	0.75
Anim. fat and anim. protein	0.48	0.59	0.26	0.48	−0.18	0.36	0.20	0.19	0.28	0.46	−0.60	−0.55	−0.34	0.60	0.65	0.71
Total protein	0.26	0.39	−0.06	0.16	−0.32	0.06	0.10	0.07	0.17	0.24	−0.42	−0.37	−0.38	0.29	0.30	0.36
Total fat	0.34	0.47	0.31	0.41	−0.18	0.32	0.10	0.04	0.43	0.38	−0.66	−0.59	−0.23	0.49	0.58	0.58
Total fat and tot. protein	0.34	0.48	0.21	0.36	−0.24	0.25	0.11	0.05	0.38	0.36	−0.63	−0.57	−0.30	0.46	0.53	0.55
% CA energy	-0.26	-0.42	-0.30	-0.45	0.21	-0.30	-0.06	-0.00	-0.44	-0.38	0.67	0.60	0.27	-0.51	-0.55	-0.60
% PC CARB energy	−0.36	−0.48	−0.38	−0.53	0.15	−0.32	−0.11	−0.08	−0.45	−0.48	0.67	0.59	0.28	−0.55	−0.60	−0.67
% Plant food energy	−0.55	−0.60	−0.38	−0.54	0.08	−0.44	−0.30	−−0.31	−0.17	−0.49	0.54	0.49	0.29	−0.65	−0.70	−0.76
Total energy	0.38	0.45	0.15	0.21	−0.15	0.19	0.18	0.12	0.28	0.29	−0.43	−0.41	−0.20	0.31	0.44	0.40
Vegetables and cereals	−0.58	−0.49	−0.49	−0.63	−0.05	−0.43	−0.41	−0.48	0.02	−0.32	0.34	0.36	0.20	−0.51	−0.64	−0.67
Milk and vegetables	−0.35	−0.20	−0.62	−0.68	−0.28	−0.55	−0.50	−0.54	−0.20	−0.35	0.25	0.35	0.03	−0.36	−0.67	−0.62
Milk and veget. and cereals	−0.43	−0.31	−0.62	−0.71	−0.17	−0.51	−0.44	−0.48	−0.25	−0.39	0.38	0.44	0.10	−0.45	−0.71	−0.69
Protein index	0.39	0.45	0.04	0.29	−0.24	0.13	0.14	0.22	−0.10	0.15	−0.39	−0.35	−0.39	0.37	0.40	0.45
Smoking (men)	−0.18		−0.45		0.07		−0.02		−0.31		0.73		0.41		−0.57	
Smoking (women)		0.35		0.21		0.17		0.02		0.38		−0.38		0.55		0.39
Raised chol. (men)	0.50		0.36		−0.16		0.27		0.39		−0.58		−0.36		0.65	
Raised chol. (women)		0.61		0.55		0.41		0.35		0.45		−0.43		0.51		0.76
GDP per capita	0.35	0.59	0.27	0.48	0.26	0.30	0.07	0.05	0.24	0.40	−0.70	−0.62	−0.49	0.51	0.53	0.67

*% CA energy, the proportion of energy from carbohydrates and alcohol (as % of total energy intake); % PC CARB energy, the proportion of energy from potato and cereal carbohydrates (as % of total energy intake); Protein index, indicator of dietary protein quality (the ratio between dairy and wheat proteins); GDP per capita, gross domestic product per capita*.

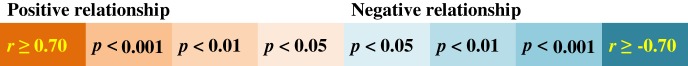

**Figure 2:**
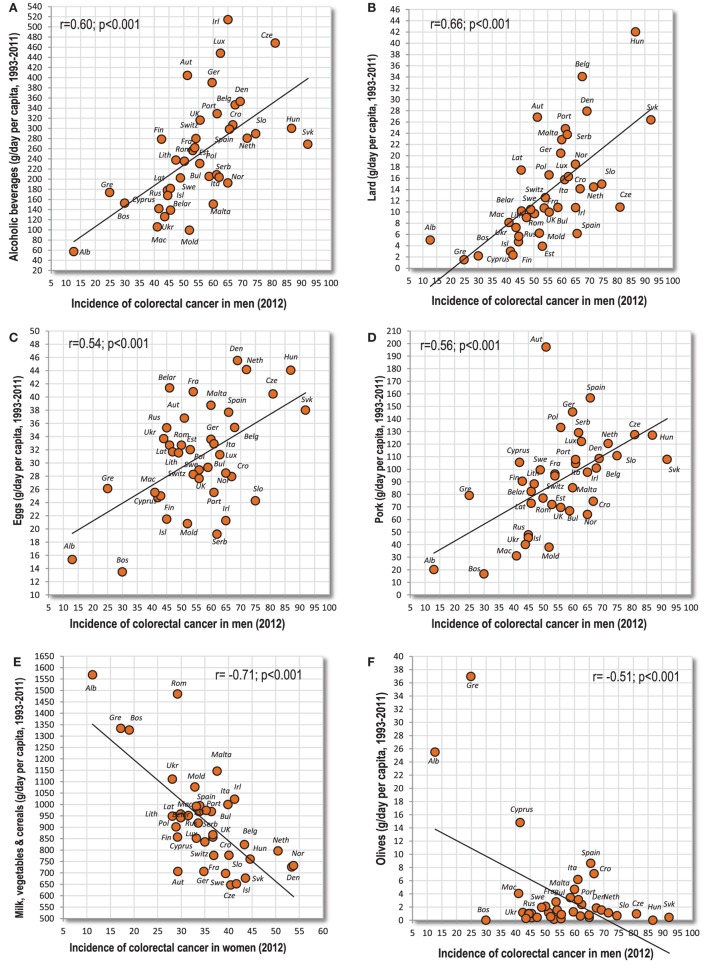
**(A–F)** Relationship between major independent variables and colorectal cancer.

Eleven cancers of the digestive system (esophagus, colon and rectum, gallbladder, and kidney in both sexes; women’s oral cavity and pharynx, pancreas and bladder) are significantly correlated with alcoholic beverages and beer. Alcoholic beverages are most strongly associated with men’s colorectal cancer (*r* = 0.60, *p* < 0.001) (Figure 2A). Lard and eggs show significant relationships with nine cancers. Lard reaches the highest positive *r*-value in the whole group—with men’s colorectal cancer (*r* = 0.66, *p* < 0.001) (Figure 2B). Eggs do not reach such high values, but they are also most strongly linked to men’s colorectal cancer (Figure 2C). Meat total and pork correlate with eight tumors, but only pork reaches high significance, with men’s colorectal cancer (*r* = 0.56, *p* < 0.001) (Figure 2D).

The most frequent negative correlates (with 10 cancers) are the combinations of milk with vegetables, and particularly milk with vegetables and cereals (*r* = −0.71 colorectal cancer in women) (Figure 2E). These food items are followed by the combination of vegetables and cereals (nine cancers), vegetables, olives and % PC CARB energy (eight cancers), tomatoes and the proportion of plant food energy (seven cancers), and milk, cereals, oilcrops and onions (six cancers). Interestingly, the negative *r*-values in olives are driven by three countries from the Mediterranean (Albania, Cyprus, Greece), where the consumption of olives reaches a sufficient level (Figure 2F).

#### Lung and Larynx Cancer

**Figure 3:**
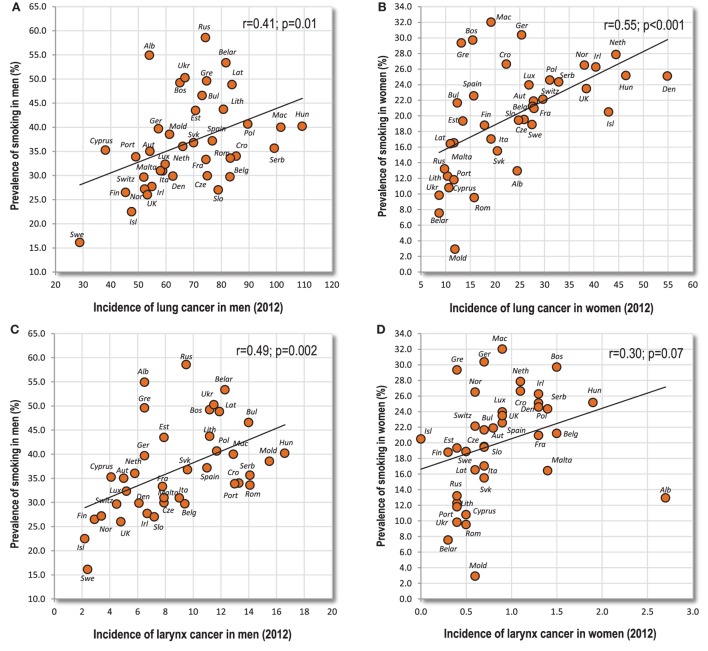
**(A–D)** Relationship between the prevalence of smoking, and lung and larynx cancer.

This group of cancers makes up a special category because it can be linked quite consistently with the prevalence of smoking, but this connection is not as strong as we would probably expect, both with lung cancer (*r* = 0.41, *p* = 0.01 in men; *r* = 0.55, *p* < 0.001 in women) and larynx cancer (*r* = 0.49, *p* = 0.002 in men; *r* = 0.30, *p* = 0.07 in women) (Figures 3A–D). The sum of incidence rates of these two cancers does not increase *r*-values either (*r* = 0.44, *p* = 0.005 in men; *r* = 0.55, *p* < 0.001 in women). Furthermore, the correlation between food and these cancers differs by sex (Table 2). This must be ascribed to the fact that men’s and women’s smoking rates in Europe tend to have an opposite geographical pattern (*r* = −0.21; *p* = 0.21). The only food item showing similar (negative) correlations with both sexes is fish and seafood in the case of larynx cancer.

#### Stomach and Cervical Cancer

**Figure 4:**
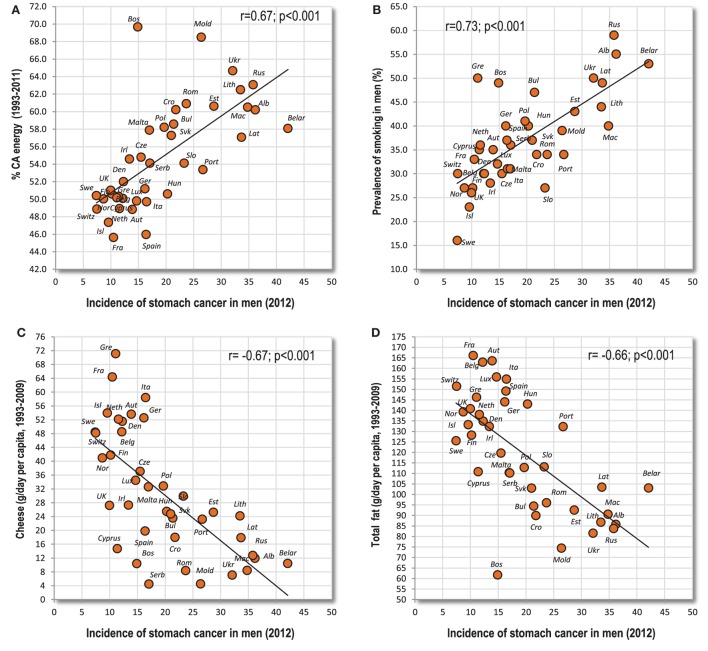
**(A–D)** Relationship between major independent variables and stomach cancer.

These cancers predominate in the eastern half of Europe. Even ovarian cancer shows similar tendencies, but they are substantially weaker. In sharp contrast to the first group, the strongest positive association can be found with lower GDP per capita, a plant-based diet, rather low cholesterol levels, and generally a high proportion of carbohydrates (% CA energy, % PC CARB energy) (Figure 4A). However, the strongest positive correlation can be found between stomach cancer and smoking in men (*r* = 0.73, *p* < 0.001) (Figure 4B). The strongest negative correlate is the intake of total fat and cheese (Figures 4C,D).

#### Cancers Correlated With a Specific Variable

**Figure 5:**
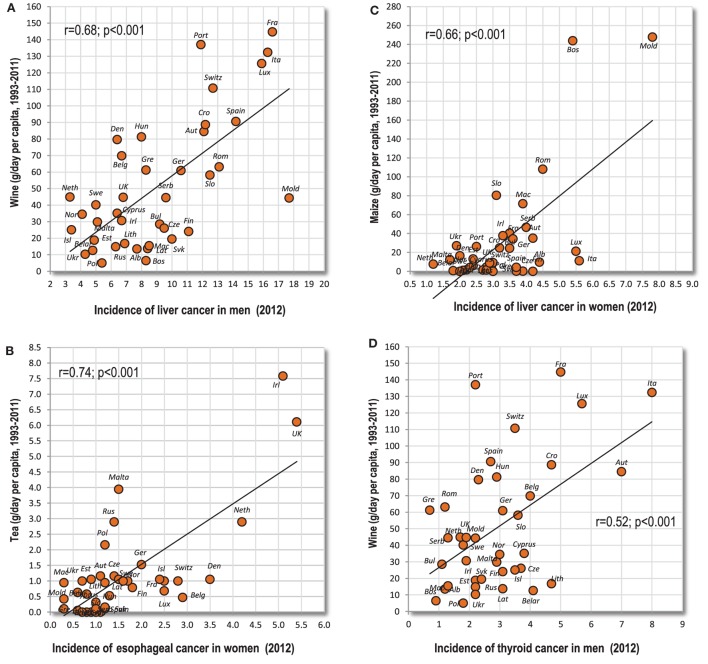
**(A–D)** Relationship between some types of cancer and highly correlated independent variables.

These cancers are characterized by a highly significant relationship to a specific food item. For example, there is quite a strong connection between liver cancer and wine drinking in men (*r* = 0.68, *p* < 0.001) (Figure 5A). For change, maize consumption correlates more strongly with liver cancer in women (*r* = 0.66, *p* < 0.001) (Figure 5B). Although the latter finding largely depends on two outliers (Bosnia and Herzegovina and Moldova), it retains moderate significance even when Spearman’s non-parametric correlation is used (*r* = 0.43, *p* = 0.006). Furthermore, esophageal cancer is significantly associated with multiple food items, especially alcohol, but the strongest relationship can be found with tea drinking, especially in women (*r* = 0.74; *p* < 0.001) (Figure 5C). Again, it largely depends on two countries (Ireland and UK), but its Spearman’s correlation coefficient remains quite strong (*r* = 0.61, *p* < 0.001). Another noteworthy example is that of men’s thyroid cancer and wine drinking (*r* = 0.52; *p* < 0.001) (Figure 5D).

#### Cancers Unrelated to Examined Factors

These include mainly cancers of the corpus uteri (endometrium) and thyroid in women, and brain cancer in both sexes. Here, very few (1–5) significant correlations with independent variables can be found, but the incidence of some of these cancers is relatively high: 8.6 cases (men) and 6.4 cases (women) of brain cancer, 9.3 cases of women’s thyroid cancer and 19.9 cases of uterine cancer.

The lack of any closer relationship to nutrition, obesity, smoking, and economic wealth could suggest that a more important role is played by some unknown environmental (or genetic) factors in certain regions. Indeed, the incidence of brain cancer is conspicuously concentrated in the Western Balkans and the Baltic region. Furthermore, these unknown factors must equally influence both sexes because the incidence of brain (*r* = 0.78) and thyroid (*r* = 0.86) cancer in men and women mutually highly correlates. On the other hand, the low correlation coefficients found in corpus uteri cancer can have an easier explanation because according to Ferlay et al. (23), the available statistics are unreliable and influenced by misdiagnosis. Still, the incidence of corpus uteri cancer tends to be the highest in Eastern Europe—similar to ovarian and cervical cancer (compare Figures S21 and S22 in Supplementary Material). For change, the incidence of brain cancer is visually somewhat lower in Eastern Europe (see Figures S23 and S24 in Supplementary Material).

#### Total Cancer Incidence

**Figure 6:**
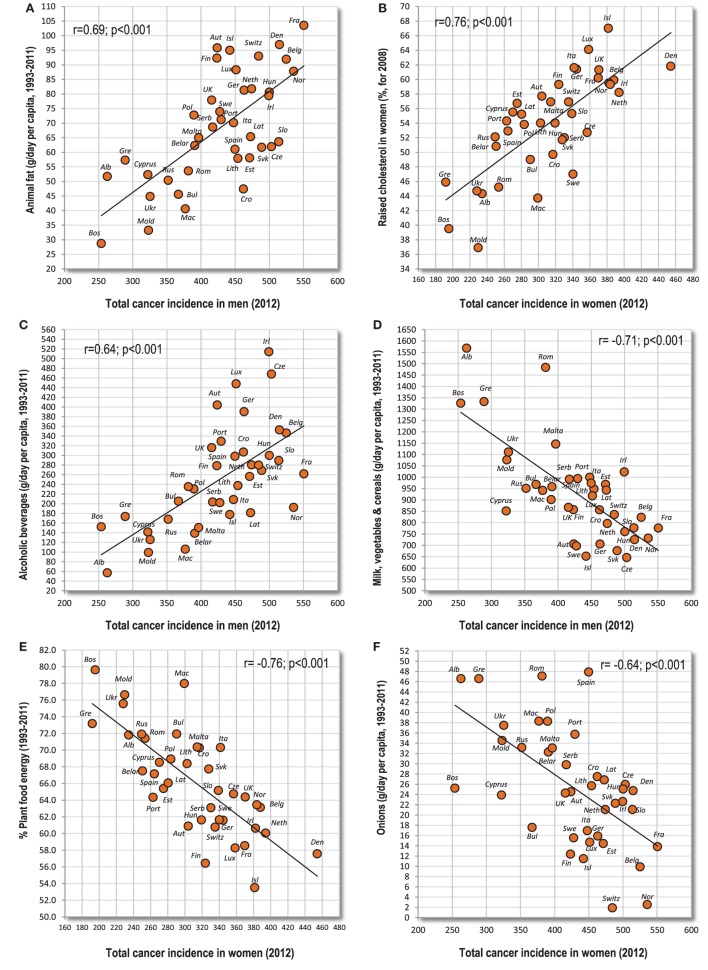
**(A–F)** Relationship between major independent variables and total cancer incidence.

For both sexes, the highest total cancer incidence is concentrated in Northwestern, Northern and Central Europe (particularly in Denmark, France, and Norway). Its strongest correlates are animal fat (*r* = 0.69 in men, *r* = 0.75 in women; *p* < 0.001) (Figure 6A) and raised cholesterol (*r* = 0.65 in men, *r* = 0.76 in women; *p* < 0.001) (Figure 6B). At the same time, meat fat, lard, and fish and seafood fat correlate with cancer far more strongly than dairy fat. Among individual food items, alcoholic beverages reach the highest *r*-values (*r* = 0.64 in men, *r* = 0.57 in women; *p* < 0.001) (Figure 6C), followed by meat, soybean oil, coffee, and bananas.

The list of negative correlates is dominated by the combined consumption of milk, vegetables and cereals (*r* = −0.71 in men, *r* = −0.69 in women; *p* < 0.001) (Figure 6D) and the proportion of plant food energy (*r* = −0.70, *p* < 0.001 in men; *r* = −0.76, *p* < 0.001 in women) (Figure 6E). Other items associated highly significantly (*p* < 0.001) are vegetables and cereals, milk and vegetables, % PC CARB energy, onions (Figure 6F), % CA energy, vegetables, plant protein, cereals, and smoking in men.

### Factor Analysis

**Figure 7:**
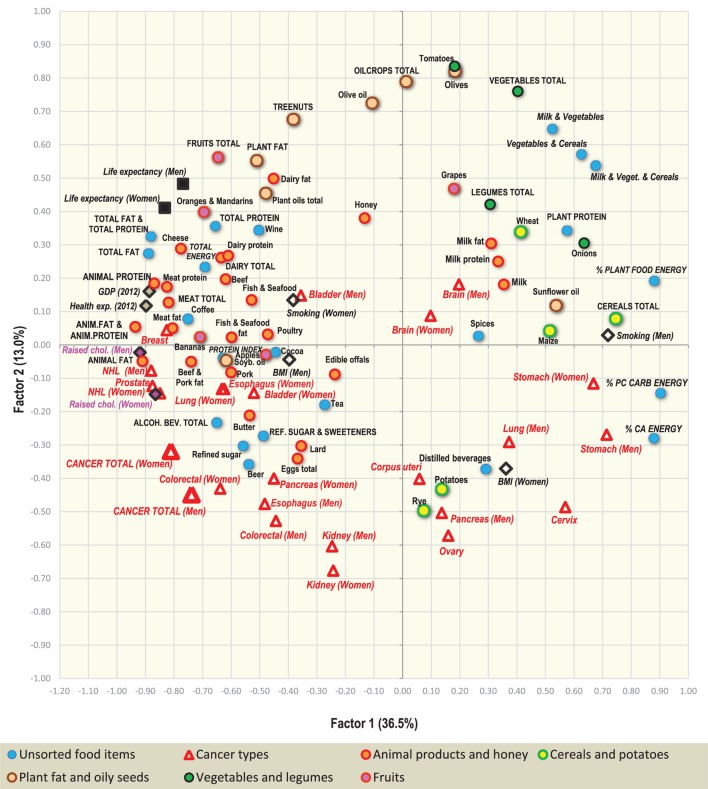
Factor analysis: a plot of Factor 1 and Factor 2 explaining 49.5% variability. For better clarity, only the main indicators of cancer incidence were selected (103 variables total). *Abbreviations*: NHL, non-Hodgkin lymphoma; % PC CARB energy, the proportion of energy from potato and cereal carbohydrates (as % of total energy intake); % CA energy, the proportion of energy from carbohydrates and alcoholic beverages (as % of total energy intake); Protein index, the ratio between milk and wheat proteins (an indicator of a high dietary protein quality).

**Figure 8:**
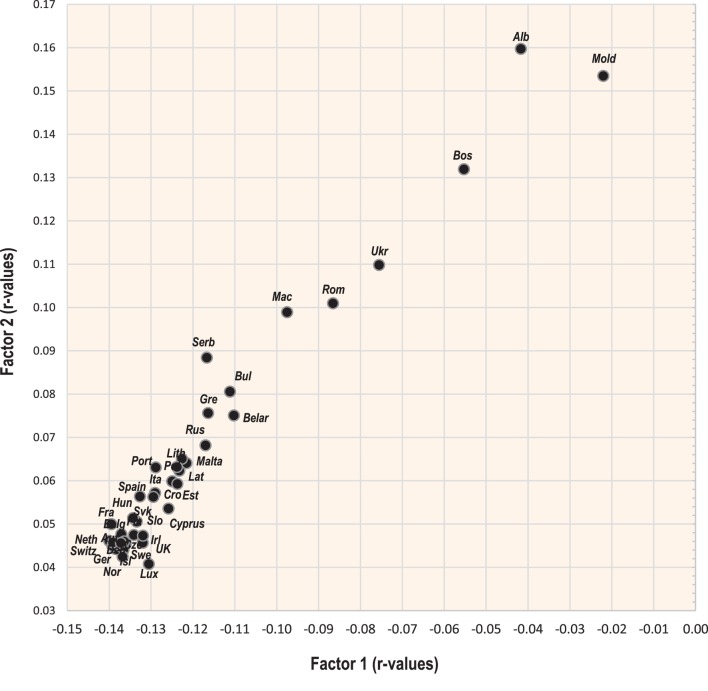
Projection of 39 countries on the factor plane of Figure 7.

**Figure 9:**
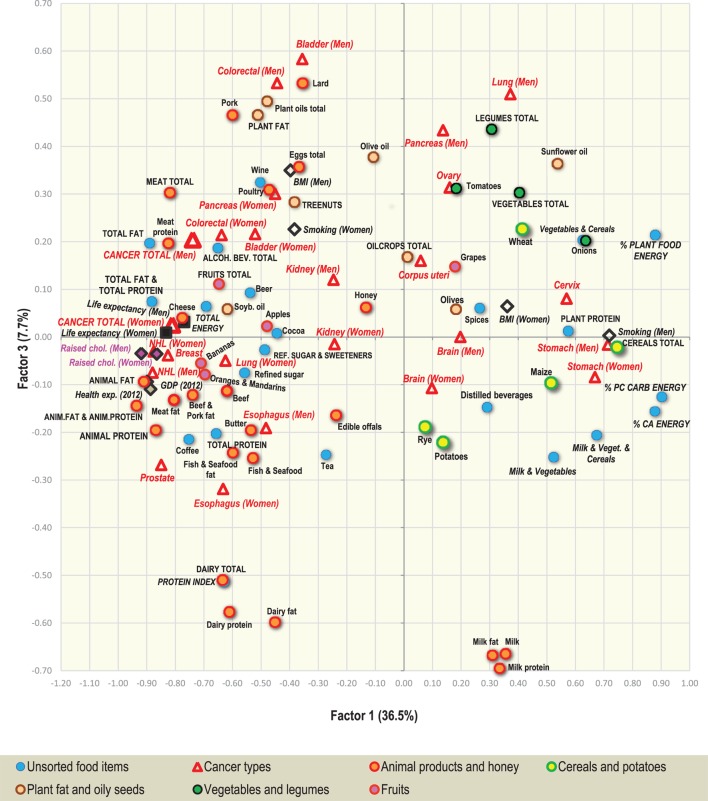
Factor analysis: a plot of Factor 1 and Factor 3 explaining 44.2% variability. For better clarity, only the main indicators of cancer incidence were selected (103 variables total). *Abbreviations*: NHL, non-Hodgkin lymphoma; % PC CARB energy, the proportion of energy from potato and cereal carbohydrates (as % of total energy intake); % CA energy, the proportion of energy from carbohydrates and alcoholic beverages (as % of total energy intake); Protein index, the ratio between milk and wheat proteins (an indicator of a high dietary protein quality).

Two-dimensional plots created by the combination of Factor 1 with Factor 2/Factor 3 Figures 7 and 9 display mutual relationships among 78 independent variables and 14 types of cancer and total cancer incidence. Factor 1 includes the largest proportion of variance by far (36.5%) and in the left half of the graph, it groups together the incidence of most types of cancer (particularly prostate, breast, and NHL), raised cholesterol and high animal fat and animal protein consumption. These variables are put in opposition against variables in the right half of the graph: cancers of the stomach and cervix, and a high intake of milk, vegetables, cereals, and plant food in general (Figure 7). This division corresponds with the striking difference between the diets of the wealthy West and North European countries, and the less developed countries of Southeastern Europe (Figure 8).

Factor 2 explains 13.0% of variance. Again, it largely separates the diets of Western and Northern Europe from those of Southeastern Europe, but this time, it highlights the polarity between cancers of the digestive tract on the one hand, and a diet rich in oilcrops (olives)/vegetables on the other hand. The combination of Factor 1 and Factor 2 emphasizes the polarity between the cancer-related diets of Northern, Western, and Central Europe, and those of Southeastern Europe, where total cancer incidence is the lowest (Albania, Moldova, Bosnia, and Herzegovina). Similar to Pearson linear correlations, total cancer incidence is positively associated with variables such as animal fat, alcohol, and lard, whereas the lowest cancer incidence is related to the consumption of cereals, olives, vegetables (onions, tomatoes), plant food, and the combined intake of milk, vegetables, and cereals. Fruits—which otherwise correlate positively with cancer incidence—are clearly separated from cancer in this division.

**Figure 10:**
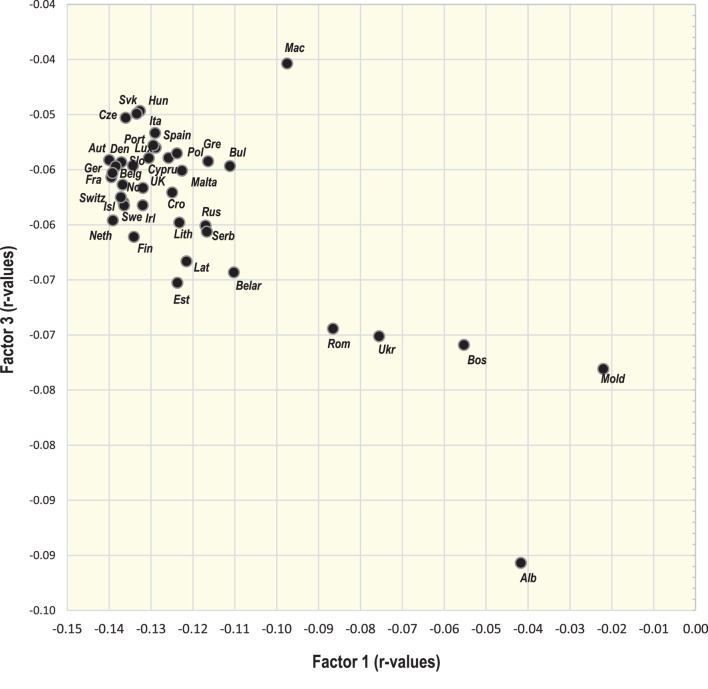
Projection of 39 countries on the factor plane of Figure 9.

Factor 3 explains 7.7% of variance. In the southern part of the graph (Figure 9), it highlights diets based on dairy products. The opposite, northern half of the graph is dominated by countries, where dairy consumption is the lowest (Hungary, Macedonia, Slovakia, etc.). At the same time, Factor 1 separates diets with high total dairy intake, but a relatively low intake of milk (Finland, the Netherlands, Sweden), from those, which consume dairy mostly in the form of liquid milk (Albania, Romania, Ukraine) (Figure 10). This division also displays the positive ecological relationship between certain cancers (prostate, esophagus) and high protein quality/total dairy intake, and the negative relationship between cancers of the digestive tract and milk. Apparently, milk is not closely associated with the incidence of any cancer type.

### Penalized Regression Models

Table 3 lists variables that appeared most frequently among the top 5 with the highest absolute *beta* coefficients, in all 12 penalized regression models that were computed for 12 cancer types and total cancer incidence (see Tables in Supplementary Material). These variables are further subdivided according to their role in the models (positive/negative *beta* coefficients). In summary, there are some differences in details from the Pearson linear correlations because these regression models have a tendency to select a common denominator out of a large set of variables. Such a selected factor can serve as a proxy for certain dietary patterns, but does not necessarily express direct causality. Moreover, in some complex models, where we find a pair of highly correlated variables, one of them can acquire a *beta* coefficient with a different sign than in the Pearson correlations (e.g., alcoholic beverages vs. beer in the case of men’s esophageal cancer). However, the results generally go in the same direction.

**Table 3:** Frequency of variables that appeared among top 5 with the highest absolute *beta* coefficients, in 12 models of the ridge regression, LASSO regression, and elastic net regression.

Cancer types	beta (+/−)	Optimal models	Optimal and parsimonious models total

		6 models total	12 models total
Prostate cancer	+	Beef and pork fat (6), soybean oil, raised cholesterol (5)	Beef and pork fat (12), soybean oil (11), raised cholesterol (9)
	−	% Plant food energy (5), smoking (4)	% Plant food energy (9), smoking (6), sunflower oil (2)

Testicular cancer	+	Animal fat, lard (3), cheese (2)	Animal fat (7), cheese, lard (5)
	−	Onions (4), bananas, MVC, % PC CARB energy, VC, etc. (2)	Onions (7), MVC (5), bananas, VC, % PC CARB energy etc. (4)

Breast cancer	+	Grapes (4), oilcrops, smoking (3)	Coffee (7), soybean oil (6), oilcrops (5)
	−	Dist. beverages (5), % CA energy, MVC, potatoes (3)	Dist. bev. (7), % CA energy (6), % PC CARB energy, potatoes (5)

Non-Hodgkin lymphoma (NHL) (men)	+	Pork (3), treenuts (2)	Pork (5), bananas, treenuts (4)
	−	Olives, smoking (4), vegetables, MVC (2)	Olives, smoking (7), MVC (4)

NHL (women)	+	Bananas, pork (5), soybean oil (4)	Soybean oil (9), pork, bananas (7)
	−	Distilled beverages, olives (4), % plant food energy (2)	Olives (7), distilled beverages, % plant food energy (6)

Multiple myeloma (men)	+	Anim. fat and anim. protein, soybean oil (3), meat, pork, etc. (2)	Anim. fat and anim. protein (6), fish and seafood fat, soybean oil (5)
	−	Smoking (3), olives, onions (2)	Smoking (5), onions (4), olives (3)

Multiple myeloma (women)	+	Meat (4), pork, wine (3)	Meat (9), pork (8), wine (6)
	−		Spices (3), plant fat, VC (2)

Leukemia (men)	+	Eggs (6), meat, pork (5)	Eggs (12), pork (10), meat (9)
	−		

Leukemia (women)	+	Anim. fat and anim. protein (6), Anim. protein, meat protein (4)	Anim. fat and anim. protein (12), anim. protein (9), meat protein (8)
	−		

Esophageal cancer (men)	+	Dairy (4), Anim. fat and Anim. protein, Dairy fat (3)	Dairy (9), dairy fat (7), lard (4)
	−	Beer, cocoa, coffee, grapes, tomatoes (2)	Grapes (6), beer, coffee, tomatoes (3)

Esophageal cancer (women)	+	Tea(6), raised cholesterol, ref. sugar and sweeteners (4)	Raised cholesterol, tea (10), Beef, ref. sugar and sweeteners (5)
	−	% Plant food energy (3)	% Plant food energy (6), grapes (4), pork (3)

Colorectal cancer (men)	+	Pork (5), eggs (3), poultry (2)	Pork (9), eggs (7), lard (3)
	−	Olives (5), oilcrops (4), MVC, VC, smoking (2)	Olives (11), oilcrops (8), MVC (7)

Colorectal cancer (women)	+	Pork (5), eggs (3), poultry (2)	Pork (11), eggs (7), poultry (2)
	−	Olives (5), oilcrops (3), dairy fat, MVC, VC (2)	Olives (10), MVC (6), oilcrops (5)

Gallbladder cancer (men)	+	BMI(5)	BMI (11)
	−	Raised chol., MV (4), vegetables, ref. sugar and sweeteners (3)	Raised chol. (6), vegetables, ref. sugar and sweeteners (5)

Gallbladder cancer (women)	+	Beer (4), Pork (3)	Beer (7), pork (6)
	−	Milk fat, ref. sugar and sweeteners, vegetables (3)	Milk fat (7), milk protein, ref. sugar and sweeteners (6)

Kidney cancer (men)	+	BMI(3), dairy (2)	BMI (7), dairy, eggs (3)
	−	Tomatoes (3), MV, oilcrops, vegetables (2)	Tomatoes (8), oilcrops (4), olives (3)

Kidney cancer (women)	+	BMI, total energy (4), beer (2)	BMI (7), raised cholesterol (5), beer, total energy (4)
	−	Vegetables (6), tomatoes (5), honey (3)	Vegetables (12), tomatoes (11), honey (5)

Bladder cancer (men)	+	Tomatoes (3), raised cholesterol, anim. fat and anim. protein (2)	Tomatoes (5), raised cholesterol, anim. fat and anim. protein (2)
	−	Tea (5), potatoes (4), milk (3)	Tea (9), potatoes (8), milk (7)

Bladder cancer (women)	+	Beer (5), lard (4), fruits (3)	Beer (9), lard (8), eggs (6)
	−	Potatoes (5), honey (2)	Potatoes (9), honey (3), % CA energy (2)

Stomach cancer (men)	+	% PC CARB energy, % CA energy (3), apples, smoking (2)	% PC CARB energy (8), % CA energy (7), apples (4)
	−	Cheese, dairy fat (4), oranges and mandarins (3)	Cheese (8), dairy fat (7), oranges and mandarins (6)

Stomach cancer (women)	+	% PC CARB energy (6), Dist. beverages (4), % CA energy (2)	% PC CARB energy (12), dist. beverages (8), % CA energy (4)
	−	Oranges and mandarins (5), alcoholic beverages, cheese (3)	Oranges and mandarins(10), alcoholic beverages, cheese (6)

Total cancer incidence (men)	+	Pork (5), lard (3)	Pork (10), lard (5)
	−	Olives (5), MVC (3), oilcrops, VC (2)	Olives (11), MVC (8), oilcrops (4)

Total cancer incidence (women)	+	Lard, raised cholesterol (4), soybean oil (3)	Soybean oil (9), lard, raised cholesterol (7)
	−	MVC, Dist. beverages (3), % CA energy, Onions (2)	MVC (9), Dist. bev. (6), %plant food en., VC, onions, %CA en. (2)

*Each row lists top 3 placed variables with positive or negative beta coefficients that appeared in this selection at least twice*.

*Beta coefficients express the influence of independent variables on the dependent variable. They show how many standard deviations the dependent variable will change, per 1 SD increase in the independent variable*.

*MV, Milk and Vegetables; MVC, Milk and Vegetables and Cereals; VC, Vegetables and Cereals; BMI, body mass index*.

Cancers of the prostate, breast, and white blood cells are most consistently linked to pork which is the main source of meat fat in the European diet and simultaneously one of the key sources of high-quality proteins supporting physical growth (16). The second most frequently identified variable is soybean oil which is used for various culinary purposes associated with animal food. The list of variables with negative *beta* coefficients is dominated by olives and smoking in men. The unexpected, seemingly protective position of distilled beverages in women finds an answer in the factor analysis (Figure 9) where distilled beverages cluster with multiple carbohydrate sources.

Pork, beer, eggs, and animal fat and animal protein/raised cholesterol are most often highlighted in connection with cancers of the digestive tract. Oilcrops and tomatoes have the highest number of negative *beta* coefficients, but milk and vegetables actually emerge even more frequently, either as individual items or in combination. Also noteworthy is the identification of high BMI as the main denominator of kidney cancer and men’s gallbladder cancer. This agrees with the WCRF data (14). Expectedly, relationships found in stomach cancer are completely different and confirm the link with carbohydrates (% PC CARB energy). Total cancer incidence primarily depends on the incidence of major cancer types such as that of prostate, breast, and colon and rectum, and this is reflected even by the spectrum of variables in the regression models.

### Temporal Changes of *r*-Values

Temporal relationships between some important variables and 12 cancers are displayed in Figures S27–S55 in Supplementary Material. Above all, the regression slope tests show a very close relationship between the trend lines of beer and pork (*p* = 0.96 in the case of women’s pancreatic cancer), or alcoholic beverages and pork (*p* = 0.86 in the case of men’s colorectal cancer). Furthermore, even mean consumption rates of beer and pork (*r* = 0.56, *p* < 0.001), and alcoholic beverages and pork (*r* = 0.67, *p* < 0.001) are mutually correlated. This suggests that when these items emerge together as significant correlates, the weaker of them will be associated only spuriously.

In contrast, the mutual connection between alcoholic beverages, lard and eggs is generally low. The mean supply of lard is quite weakly correlated with both alcohol (*r* = 0.40, *p* = 0.012) and eggs (*r* = 0.47, *p* = 0.003), and there is no close relationship between alcohol and eggs (*r* = 0.31, *p* = 0.053). In the temporal comparison, the trend line of eggs is often similar to that of alcohol, but these two items do not show any particular association with lard, except for men’s pancreatic cancer (*p* = 0.85 between beer and lard).

Remarkably, animal fat and animal protein, and coffee are one of the strongest correlates of the cancers of the prostate, breast, multiple myeloma, NHL, and testes, and their mean supply rates are mutually correlated as well (*r* = 0.69, *p* < 0.001), but apart from women’s multiple myeloma (*p* = 0.052), their trend lines are completely unrelated (*p* < 0.001). Other variables obviously do not affect *r*-values of coffee either, with the possible exception of alcohol (see Figures S34, S38, and S41 in Supplementary Material), but judging from the situation in men, the opposite direction of influence is more likely.

## Discussion

The results of our present study show that the incidence of 24 cancer types in Europe differs by geography and is characteristically tied to specific lifestyle factors. Because ecological findings alone cannot establish causality at an individual level, and even the most sophisticated statistical analyses are not likely to resolve all issues with multicollinearity within such a large sample, the interpretation of these results requires a meaningful rationale and support in the existing literature.

### “Cancers of Affluence”

The tumors of the prostate, breast, melanoma, and cancers of white blood cells are typical of countries with high GDP per capita and constitute a heterogenous group that seemingly has nothing in common. Despite that, it is the most strongly profiled group of cancers in our study, with a very robust relationship to the consumption of animal fat and animal products in general. This suggests that the incidence of these cancers may be influenced by some fundamental physiological mechanism that depends on dietary factors. Indeed, the consumption of saturated animal fat manifests by high total cholesterol and HDL-cholesterol (29, 30). Although some recent studies and reviews link high fat consumption and high total cholesterol with increased cancer risk (6, 11), and hypercholesterolemia in mammals indeed promotes cancer growth (31), this debate has not been definitely settled yet. Actually, some authors regard high HDL-cholesterol as a protective factor against both cancer and CVDs (32), but this opinion completely contradicts the ecological picture because the incidence of cancer and CVDs in Europe has an inverse geographical pattern (17). The possibility that HDL-cholesterol in cancer patients is decreased as a result of cholesterol accumulation in growing cancer cells (reverse causation) could reconcile clinical and ecological data (12).

In the temporal comparison, the correlations with animal fat and animal protein do not change much with time, but consistently peak ~7–15 years before detection. The role of animal fat is driven mainly by meat fat whose proportion in the modern Western diet is unnaturally high due to the consumption of fatty meat from domesticated animals. To illustrate this point, the average European in our sample had a mean supply of 25.7 g meat fat/day and 23.6 g meat protein/day. In contrast, the cooked meat of wild boar is composed of 28% protein and only 4% fat (33).

This group of cancers also correlates with high protein quality (represented by the “protein index”) and the fact that cancers of white blood cells in children are accompanied by excessively high IGF1 levels (13) is unlikely to be a mere coincidence. Nevertheless, the “protein index” is markedly associated only with prostate cancer and melanoma, and only in these two cases it retains significance (*p* < 0.05) after controlling for animal fat or raised cholesterol. Besides that, the obvious prerequisite for melanoma is skin depigmentation in the northern regions of Europe where dietary protein quality is the highest in the world (16), and hence these factors must inevitably interfere with each other.

Perhaps, the connection between high protein quality and cancer would be more apparent if we could take into account the diet of patients’ mothers during pregnancy or the patients’ diet during childhood because these are factors affecting hormonal levels and adult stature (10). However, this is possible only partially because the FAOSTAT statistics start in 1961. Still, long-term correlations between the “protein index” and some of the “cancers of affluence” (in a sample of 24 countries) peak after a longer interval than in other variables, especially in the case of prostate cancer (*r* = 0.77 in 1982—30 years before detection) (Figures S56 and S57 in Supplementary Material).

Another noteworthy finding is the highly significant role of coffee in relation to these cancers, especially prostate cancer (*r* = 0.71, *p* < 0.001). The ecological association between coffee and cancer is well-known, but so far, it has not been convincingly supported by other evidence. A recent metaanalysis by Arab (34) concluded that multiple observational and case-control studies were not able to find any causal connection between coffee drinking and prostate/breast cancer. Nevertheless, the author does not exclude a possible association between children’s leukemia and a very high coffee consumption in their mothers during pregnancy. In our study, the statistical significance of coffee mostly disappears after adjusting for animal fat and animal protein, with the exception of prostate cancer and melanoma (*p* < 0.01). Penalized regression models identify coffee as an important variable only in the case of breast cancer. On the other hand, there is a surprisingly weak relationship between coffee and other highly correlating variables in the temporal comparison. In fact, the temporal trend lines point to a potential problem with a long latency period because the *r*-values between coffee and the “cancers of affluence” mostly peak in the same year (1998—14 years before detection), with a subsequent rapid decrease.

In contrast with the conclusions of some metaanalyses, our study could not demonstrate any particular role of alcohol in relation to these cancers. Although some of the documented *r*-values are relatively high, reaching *r* = 0.62 (*p* < 0.001) between alcoholic beverages and NHL in women, the significance of these correlations disappears after adjusting for animal fat and animal protein. The only exception is just women’s NHL (*p* = 0.013). A noteworthy case is also that of testicular cancer in the temporal comparison (Figure S41 in Supplementary Material), but this tumor is not a model representative of this group even in some other aspects. Because alcohol consumption is connected mainly with breast cancer, and separate statistics of premenopausal and postmenopausal breast cancer are not available, we cannot say if the role of alcohol could depend on age and hormonal status. The updated WCRF project panel states that alcohol and higher attained height are convincing risk factors of breast cancer irrespective of menopausal status (14).

In general, these cancers occur rarely in countries whose diet is based on plant food (cereals and vegetables) which is in accordance with the poor biological quality of most plant proteins (16, 22). Carbohydrates (coming overwhelmingly from plant sources) decrease total cholesterol and HDL-cholesterol levels (30, 31). Furthermore, current evidence indicates that some types of vegetables such as tomatoes may decrease IGF1 levels (35). Interestingly, among individual food items, the penalized regression models highlight mainly olives (which may symbolize the “Mediterranean” dietary style), whereas Pearson linear correlations clearly point to onions whose anti-cancerogenic properties are already supported by a significant amount of data (36). On the other hand, our study shows that fruits do not prevent cancer in countries consuming large quantities of animal fat and animal protein.

### The Negative Ecological Relationship Between the “Cancers of Affluence” and Smoking

An unexpected finding worthy of a detailed discussion is the strongly negative correlation between these cancers and smoking in men. At the same time, the documented *r*-values are among the very highest in this study, reaching *r* = −0.78 (*p* < 0.001) with men’s NHL. Furthermore, these results are unequivocally supported even by the regression models. Moderately strong negative correlations can be observed even with men’s testicular and colorectal cancer. In women, who smoke much less than men, and whose geographical pattern of smoking is very different, the relationships are always opposite (positive), albeit much weaker and mostly barely significant (compare Figures S58A–F in Supplementary Material).

According to recent metaanalyses, the cumulative effect of heavy smoking may increase the risk of prostate cancer incidence and death (37), but the relative risk of current smoking in relation to the incidence of prostate cancer, colorectal cancer and cancers of white blood cells (myeloid leukemia) is the lowest out of all cancer types and insignificant (37, 38). Interestingly, current smokers have a lower risk of these cancers than former smokers, which also differs from other cancer types. These are intriguing conclusions indicating that our ecological findings may not be completely unfounded. Actually, the seemingly paradoxical link between smoking and a lower risk of some cancers might have a reasonable explanation because smoking demonstrably decreases HDL-cholesterol (39). At the same time, smoking is often closely associated with lifestyle factors increasing cancer risk (e.g., the lack of physical activity, alcoholism) and hence these relationships could be blurred in observational studies at the individual level.

Alternatively, men’s smoking would have to be very strongly tied to some powerful confounder. This assumption is certainly justified because men smoke mainly in countries consuming foods that are associated strongly negatively with the “cancers of affluence” (% CA energy; % PC CARB energy; % plant food energy; milk and vegetables and cereals; onions). Nevertheless, if we include smoking into a multiple regression that works with these food items, coffee, the “protein index” and animal fat and animal protein, we find that smoking and onions are the only variables that appear in all the best regression models of men’s cancers of the prostate, melanoma, NHL and multiple myeloma (data not shown). In addition, smoking and lard are the only variables that contribute to the best regression models of both testicular and men’s colorectal cancer (extended by alcohol and lard). On top of that, data on the actual smoking of any tobacco product from the WHO database (for 2013) (40) produce even more robust correlations, especially with men’s NHL (*r* = −0.86; *p* < 0.001). In fact, when such actual data are used, the positive trends documented in women show clear signs of reverse tendencies similar to those of men (compare Figures S59A–F in Supplementary Material). Therefore, it is by no means easy to explain these results as purely spurious. At the very least, our data practically exclude the possibility that smoking *per se* could be one of the major triggers of these cancers.

### “Cancers of Unhealthy Lifestyle”

The specific association of cancers of the digestive tract with alcoholic beverages supports the available evidence in this regard (41) which is linked with the direct exposure of cells to ethanol and the mutagenic properties of its metabolite acetaldehyde (42). Out of all alcoholic beverages, beer is by far the most frequent correlate of cancer incidence (especially esophageal cancer), which naturally follows from the high frequency of consumption. Wine is the strongest predictor of men’s liver cancer and even men’s thyroid cancer, but the latter finding would contradict the results of observational studies (43). Irrespective of the potentially contributing role of alcohol, thyroid cancer is usually linked with the frequent exposure to ionizing radiation (X-rays) (4).

In the majority of temporal comparisons, the *r*-values of alcohol rapidly increase with increasing time and peak ~15–20 years before detection. However, there are notable exceptions. Pancreatic cancer significantly correlates with mean alcohol/beer consumption only in women, but in the temporal comparison (Figure S50 in Supplementary Material), the trend line in men is cumulative and reaches a statistically significant peak with beer (*r* = 0.35, *p* = 0.027) only 4 years before detection (2008). This could indicate that pancreatic cancer in men is primarily an acute disease caused by heavy binge drinking. Indeed, observational studies show that the risk of pancreatic cancer is increased mainly when very large amounts of alcohol (>40 g/day) are consumed (43). The same cumulative trend appears in the case of men’s esophageal cancer and kidney cancer in both sexes (Figures S46, S52, and S53 in Supplementary Material).

In the light of these ecological data, the supposedly protective effect of alcohol in relation to kidney cancer, emerging from recent metaanalyses of observational studies (44), is quite surprising. The incidence of both kidney and pancreatic cancer has recently reached a global peak in the Czech Republic—a country competing with Ireland for the biggest consumer of beer in the world. These discrepancies could be reconciled, provided that the true risk agent is not alcohol *per se*, but some other factor such as the overload of kidneys by binge drinking. Furthermore, Czech men have the highest BMI in Europe—a convincing risk factor of kidney cancer according to the WCRF (14).

In contrast with beer and wine, distilled beverages rarely reach significant correlations with any type of cancer, despite that they are the most concentrated source of alcohol. In fact, most of these correlations are very weak and negative. At the same time, distilled beverages were associated with CVD risk in our previous study (17). This raises the possibility that chronic drinkers of distilled alcohol die earlier from CVDs and other alcohol-related health problems, which blurs the relationships with cancer at the ecological level. This assumption is supported by the fact that distilled beverages reach a significantly positive correlation only when their effect appears to be acute—in the case of kidney cancer in the temporal comparison, just 4 years before detection (*r* = 0.41, *p* = 0.010 in men; *r* = 0.36, *p* = 0.026 in women). Our experience also shows that the widespread home production of distilled beverages in Eastern Europe is mirrored by their natural substrates (potatoes and rye). Indeed, it is exactly these two items that are linked to kidney cancer.

The correlation between lard/eggs and cancers of the digestive tract is perhaps even more intriguing because it can be explained by their role during the frying of food or the use of lard in smoked meat. This assumption can be demonstrated by the example of Hungary—a country with moderate alcohol consumption, but a traditionally high consumption of smoked and fried foods. Soybean oil—the strongest correlate of colorectal cancer in women—is also used for frying.

A recent report of the International Agency for Research on Cancer (IARC) regards the connection between processed meat and cancer as sufficiently convincing, particularly in the case of colorectal cancer (45). It is attributed to various chemicals, which are formed *via* preservation methods or heat treatment. In contrast, the evidence for the carcinogenicity of unprocessed meat (and red meat in particular) is still limited and our results are similarly ambigious. Although meat and pork significantly correlate with many cancers of the digestive tract, their *r*-values are not among the highest. Pork emerges as the common denominator of many digestive cancers in the penalized regression models, but pork is also the strongest dietary correlate of both lard (*r* = 0.53) and alcoholic beverages (*r* = 0.67, *p* < 0.001), and hence it is basically a proxy for unhealthy dietary habits. Similar relationships between high red meat intake and unhealthy lifestyle routinely emerge in observational studies and constitute a serious problem during the interpretation of results. In fact, when alcohol, lard, eggs, and pork are included in a multiple regression model of digestive cancers, pork remains a weakly significant correlate (*p* = 0.039 in men, *p* = 0.041 in women) only in the case of esophageal cancer, where it is vastly overshadowed by alcohol.

A very important case that confirms the meaningfulness of our methodology is that of maize and liver cancer because it can be explained by the content of aflatoxins in maize (46). Similarly, a chronic irritation of the esophagus by a hot liquid is a very meaningful explanation of the ecological relationship between esophageal cancer and tea drinking. A recent IARC report came to the same conclusion (47).

Individual food items with the most consistent negative correlations are vegetables (onions and tomatoes), milk, cereals, and olives. This observation may not necessarily reflect a causal negative relationship, but the anti-cancerogenic effect of cereals and vegetables has a solid basis, and similar effects of milk and olives can also find support in the available literature. Although the role of olives in our study largely depends on three Mediterranean countries, a recent metaanalysis of Psaltopoulou et al. (48) showed that there is a consistent, negative relationship between olive oil and total cancer incidence. Still, our results highlight whole olives, which may be attributed to the combination of various beneficial ingredients (49). The case of milk deserves a special chapter.

### Dairy and the Anti-Cancerogenic Role of Whey

The current scientific evidence indicates a negative association between milk consumption and colorectal cancer (7, 50–52), and possibly even between milk/fermented milk and bladder cancer (50). These reviews agree with our data because milk is a negative correlate of colorectal, gallbladder, bladder, pancreatic, and testicular cancer. Other dairy products did not show such a negative correlation in our study, which also agrees with the results of recent metaanalyses, where cheese and other types of dairy showed at best neutral and incosistent relationship to colorectal cancer (7, 51, 52). In contrast with this beneficial effect, dairy products have been connected with the higher risk of prostate cancer (50, 53). Remarkably, total dairy and cheese consumption, but not necessarily milk intake, is one of the main correlates of prostate cancer in the present study, which is also highlighted by Factor 1 and Factor 3.

**Figure 11:**
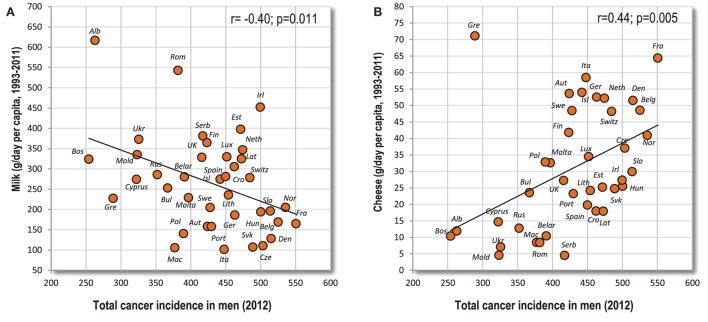
**(A,B)** Relationship between various dairy products (milk, cheese) and total cancer incidence.

The positive and negative role of dairy foods is often explained by the content of calcium (50, 51, 53), but the paradoxical relationships between cancer and various dairy products may better reflect the content of whey. Whey proteins have remarkable anti-cancerogenic properties (54, 55) and are present in milk and fermented whole milk (yogurt), but not in cheese, curd, butter or cream. A recent metaanalysis of 11 cohort studies by Lu et al. (56) gives some support to this hypothesis. Although the results were not statistically significant, milk and yogurt tended to decrease total cancer mortality, whereas cheese and butter tended to increase it. Our results indicate the same trends (Figures 11A,B).

At the same time, it is very important to note that the key whey proteins (immunoglubulins, lactoferrin, etc.) are thermolabile. While conventional pasteurization retains the majority of immunoglobulins in milk (57) and particularly careful methods can even preserve them without significant losses (58), sterilization at ultra high temperatures (UHT) destroys them completely, especially when combined with homogenization (59). This means that the consumption of pasteurized milk should not be grouped with the consumption of UHT milk.

**Figure 12:**
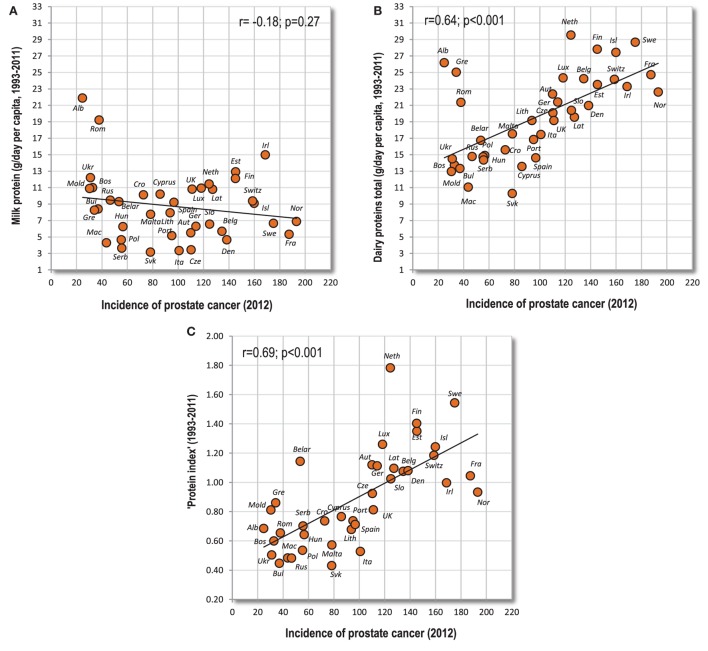
**(A–C)** Relationship between various variables associated with dairy consumption and the incidence of prostate cancer. *Note*: “Protein index” is an indicator of protein quality, and expresses the mean ratio between dairy and wheat proteins.

Because the consumption of different dairy products is often strongly tied at the individual level, it is very difficult to separate their individual effect on cancer. Ecological data do not allow a clear conclusion, either. For example, we could assume that the anti-cancerogenic properties of milk would be markedly weakened in relation to the “cancers of affluence” because milk is also a source of high-quality proteins and animal fat. Although the ecological relationship between milk and prostate cancer is basically neutral (Figures 12A,B), milk makes up only 14.6% of animal fat and animal protein intake, and its role can be influenced by various confounders. Indeed, the biggest consumers of milk in Europe (Albania and Romania) have a very low incidence of prostate cancer, but their diet is also characterized by low dietary protein quality symbolized by the “protein index” (Figure 12C). Similarly, the high consumption of dairy proteins in Greece (mostly from cheese) is probably counterbalanced by the very high consumption of vegetables and other protective factors. Resolving this important problem would thus require a long-term controlled study.

Oddly enough, even the temporal correlation between milk protein and prostate cancer (in a sample of 24 countries) strikingly differs from other foods and has completely reversed from *r* = 0.61 (*p* = 0.002) in 1961 to *r* = −0.37 (*p* = 0.07) in 2011 (see Figures S60A–F and S61 in Supplementary Material). Something similar applies to breast cancer and even to colorectal cancer (Figures S62–S64 in Supplementary Material), which contradicts our findings presented above. A more prosaic explanation is that the geographical pattern of milk consumption in Europe has dramatically changed during the 1980s, when wealthy Western nations started to replace milk with cheese (Figures S65–S68 in Supplementary Material). Because food consumption before the late 1980s correlates noticeably more weakly and sometimes insignificantly with the incidence of prostate, breast, and colorectal cancer in 2012, we can assume that even milk consumption before the late 1980s is largely irrelevant to the present cancer incidence.

Interestingly, if we subtract milk fat and milk protein from animal fat and animal protein, the positive correlations with the “cancers of affluence” mostly slightly increase or do not change (data not shown). In the case of prostate cancer, the positive *r*-values decrease only very slightly (from *r* = 0.80 to *r* = 0.78), which would suggest that the contribution of milk to the prevalence of this cancer is negligible at worst.

### Cancers Associated With Smoking

Although our statistics of smoking include only data on the average smoking prevalence, not total smoking exposure (pack-years), their expected, positive correlation with lung/larynx cancer (in both sexes) demonstrates their practical usability. The use of temporal comparisons between cancer and smoking was not possible because information for some countries was available from a limited number of years.

The lack of consistent relationships between food consumption and lung/larynx cancer could suggest that these tumors are not influenced by nutrition, but a visual inspection of Figures 3A–D indicates that their incidence is disproportionately lower in countries with the lowest cancer incidence (e.g., Greece, Albania, Russia) which can also explain the unimpressive correlation coefficients. This suggests that the manifestation of this disease is not so straightforward, and requires the presence of certain dietary factors. Alternatively, the disproportionately lower rates of men’s incidence in countries such as Russia, Belarus, and Ukraine could be due to high, premature CVD mortality which is also linked to high smoking rates (17).

Critical reviews of the available literature have also established a strong causal link between smoking and cancers of the upper digestive tract and bladder (38). Interestingly, this is reflected even in the present study because smoking in women correlates weakly positively with cancers of oral cavity and pharynx, esophagus, and bladder. Understandably, provided that these cancers also have other (dietary) triggers, the strength of ecological findings will be somewhat compromised. The lack of any significant association in men also points to the interaction with diet because men smoke mainly in countries that consume the most foods correlating negatively with these cancers. Somewhat weaker causal relationships were also proposed with other digestive organs, but except for men’s stomach cancer, they cannot be demonstrated in the present study.

### “Cancers of Poverty”

These types of cancer are represented mainly by stomach cancer. Its incidence increases with decreasing GDP per capita in Eastern Europe, and correlates most positively with smoking (in men only), and high carbohydrate consumption in general. These trends are apparent even at the global level because stomach cancer is typical of developing countries (60). At the same time, it is important to emphasize that this applies to the distal (noncardia) stomach cancer, whereas proximal (cardia) tumors prevail in developed countries and may represent a completely different disease. This could explain why our findings contrast with some recent metaanalyses that connected stomach cancer with red meat intake (61).

The most frequently cited hypothesis explaining distal stomach cancer is the infection by bacteria *Helicobacter pylori* associated with low socioeconomic status (60). Other possible causes include smoking, obesity, the consumption of salty foods (60), and a diet rich in starchy foods (62). The fact that the highest global incidence of stomach cancer occurs in wealthy countries such as South Korea and Japan suggests that bacterial infection is not the only factor. Interestingly, the proportion of cereal carbohydrates in the diet of these two countries is still twice higher than in Western Europe and mean cholesterol levels are markedly lower (data not shown). This would accord with the negative relationship between stomach cancer and total cholesterol (63) which we found even in the present study. In any case, it is clear that environmental, lifestyle and/or physiological prerequisites for this cancer must be completely different than in other cancers.

Remarkably, cervical cancer is also typical for less developed countries, both in the global and European context, and its incidence is also explained as a consequence of infection caused by the human *papillomavirus* (64). However, a potentially confounding factor of its incidence is the spread of screening programs and vaccination.

Correlations found between exogenous variables and ovarian cancer are substantially weaker, but its incidence also tends to be higher in Eastern Europe. This contradicts the WCRF conclusion stating that greater adult height (or high-quality protein diet, respectively) is a convincing risk factor (14), in addition to lifelong estrogen exposure (4). Because the highest global rates of ovarian cancer are reported in Eastern Europe, and the lowest in developing countries, there is a possibility that some protective confounder (e.g., a more frequent use of contraceptives) (65) reduces incidence rates in Western Europe. A similar geographical pattern emerges in the case of corpus uteri cancer, which is also linked to estrogen levels (4), and hence the same explanation can be proposed. Naturally, all these assumptions are relevant only if the current prevalence statistics are not too far from reality.

## Conclusion

In accordance with our previous positive experience, the present study demonstrated many strong associations that can find solid support in the available literature. One of the most convincing cases is that of tea drinking and esophageal cancer, which was independently confirmed by a recent IARC report. Such a remarkable agreement testifies that a complex ecological analysis based on good-quality data has a potential to produce valuable, biologically relevant results. In other words, even other findings should be taken seriously and their validity should be tested in clinical practice. It is not difficult to imagine that appropriate lifestyle changes inspired by such a research could be of critical importance for the survival of cancer patients.

Among the large number of findings, there are a few key points that should be highlighted. First of all, in the ongoing debate regarding the role of fat in cancer risk, our data unequivocally support the idea that high (animal) fat intake and high cholesterol levels are important factors involved in cancer progression. On the other hand, this relationship may not apply to all types of cancer and considering that high cholesterol usually mirrors high HDL-cholesterol (a major indicator of low CVD risk), the picture is not black-and-white. Ecological data indicate that the highest life expectancy is in countries with high fat consumption where part of animal fat is replaced by plant fat (17).

The mutual connection between alcohol and excessive food processing, and cancers of the digestive tract is also supported by sufficient evidence, but the harmful effect of unprocessed meat cannot be convincingly demonstrated. Findings from observational studies should, therefore, be taken with caution. If anything, unprocessed (red) meat may contribute to cancer indirectly, as a source of fat and high-quality proteins.

Another important observation is the potentially different nature of various milk products in relation to cancer risk. Previous ecological studies (18, 19) found a positive relationship between milk consumption and cancer, but our present study distinguished total dairy consumption (“Milk excluding butter total”) from milk consumption (“Whole milk”) in the FAOSTAT database. This differentiation may potentially be crucial and the illumination of this problem remains a serious challenge for future studies. A particularly promising strategy is the use of whey protein concentrates as sources of good-quality proteins for cachectic patients suffering from cancer, together with vegetables and olives [but less suitably cereals, with their high-glycemic carbohydrates (17, 31)].

Finally, we should also mention cases where our data do not accord with current views. With regard to the remarkable accordance in many other instances, the illumination of these discrepancies requires particular attention. This applies mainly to kidney cancer, where our results point to alcohol (beer) binge drinking as the major risk factor, whereas observational studies identify alcohol as a protective factor. The possible connection between coffee and some cancers will be particularly difficult to prove or disprove because it may manifest with a very long delay, or it may even have its roots in the mother’s diet during pregnancy. Perhaps the least expected result—which is simultaneously one of the strongest that have been documented in this study—concerns the negative relationship between several major cancers and smoking. Interestingly, this finding is not entirely without support in observational studies and can potentially be very important, provided that it reflects the role of cholesterol in cancer progression.

Understandably, it would not be sensible to look for a causal basis in all the results of this study. The accuracy of the available statistics is also beyond our responsibility. Nevertheless, as far as we can tell, the strong and meaningful findings can themselves serve as a testament to their practical usability. In general, it is mostly food items with very high consumption rates and especially essential nutrients (fat, protein, carbohydrates) that produce the highest correlation coefficients. The interpretation of relationships in foods with small consumption rates is more difficult due to the stronger role of confounding factors. Some variables (smoking, alcohol) can also influence premature mortality from other diseases which can weaken their ecological association with cancer prevalence. Still, as already demonstrated by the example of tea drinking and esophageal cancer, the inclusion of a large dataset from the FAOSTAT database can be very beneficial.

The broad spectrum of risk factors examined is thus the major advantage of our study, but there is no doubt that the list is not exhaustive—as evidenced by the example of brain cancer. Apart from genetic predispositions or internal disorders (immune suppression), these risk factors may include, e.g., solar/X-ray radiation, air pollution, working with toxic chemicals or drug abuse (66). Because they are generally difficult to identify or quantify, they lie beyond the scope of our methodology. Other interesting statistics (salt intake, physical activity) are still incomplete.

Remarkably, a recent longitudinal study found a dramatic 81% cancer mortality reduction in seniors in the highest tertile of physical strength (67), which demonstrates the importance of strength-related physical activity that utilizes growth factors in the human body. Because a recent metaanalysis also found a dramatic reduction in cancer mortality in individuals participating in strength exercises, but not in those participating in aerobic exercises (68), the use of generalized, self-reported data on physical activity is unlikely to produce any meaningful results, leaving aside their notorious unreliability (69). Similarly, we do not have sufficient data on some other protective factors, such as the use of oral contraceptives. The available literature shows that their more frequent use in the western half of Europe (70) could indeed explain some discrepancies between ecological and observational results.

At present, we are working on a similar comparison, using the GLOBOCAN database of cancer incidence in the whole world (71). Although not all international data in this database are regarded as highly reliable, it is noteworthy that preliminary results based on the highest quality statistics produce findings that are very similar to those from the present study which could provide additional support for their causality.

## Author Contributions

PG collected the data, drafted the manuscript, and created infographics. EH collected the data and prepared them for statistical analyses. MS and TK performed statistical analyses.

## Conflict of Interest Statement

The authors declare that the research was conducted in the absence of any commercial or financial relationships that could be construed as a potential conflict of interest. The reviewer AS and handling Editor declared their shared affiliation.
