# Supplementary material for: Cancer Incidence in Europe: An Ecological Analysis of Nutritional and Other Environmental Factors
Source: Front Oncol. 2018 Jun 13;8:151. doi: 10.3389/fonc.2018.00151 (PMC6008386; doi:10.3389/fonc.2018.00151)
Supplement: Supplementary file 2 [file data_sheet_2.PDF]

DATA SHEET 2
Supplementary Figures

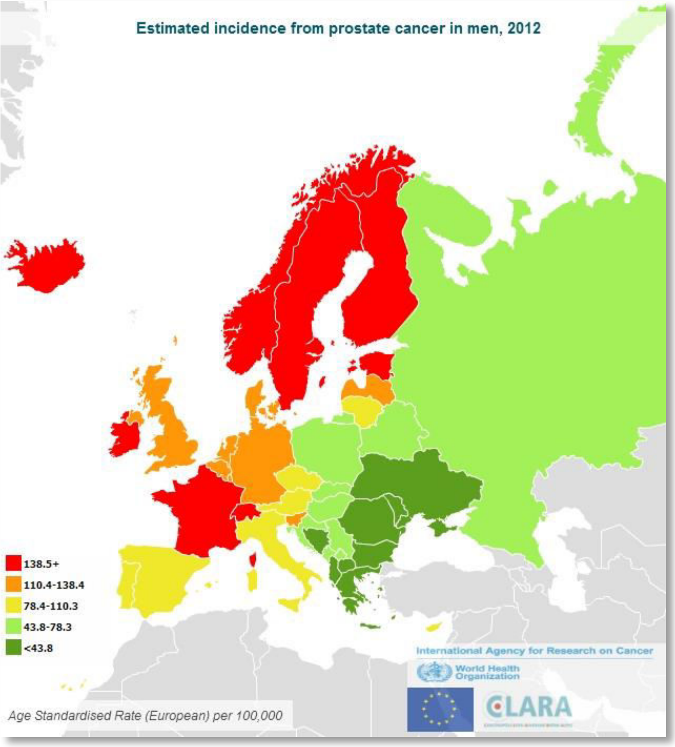

Figure S1. Estimated incidence from prostate cancer in men (2012).

Source: Steliarova-Foucher E, et al. European Cancer Observatory: Cancer Incidence, Mortality, Prevalence and Survival in Europe. Version 1.0 (September 2012), http://eco.iarc.fr/eucan/CancerSearch.aspx. All images use Creative Commons type licenses.

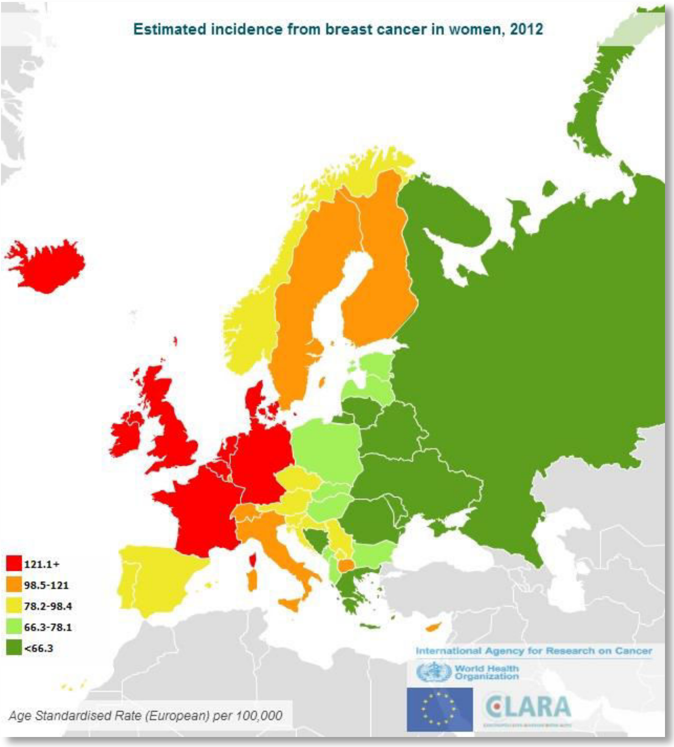

Figure S2. Estimated incidence from breast cancer in women (2012).

Source: Steliarova-Foucher E, et al. European Cancer Observatory: Cancer Incidence, Mortality, Prevalence and Survival in Europe. Version 1.0 (September 2012), http://eco.iarc.fr/eucan/CancerSearch.aspx. All images use Creative Commons type licenses.

.

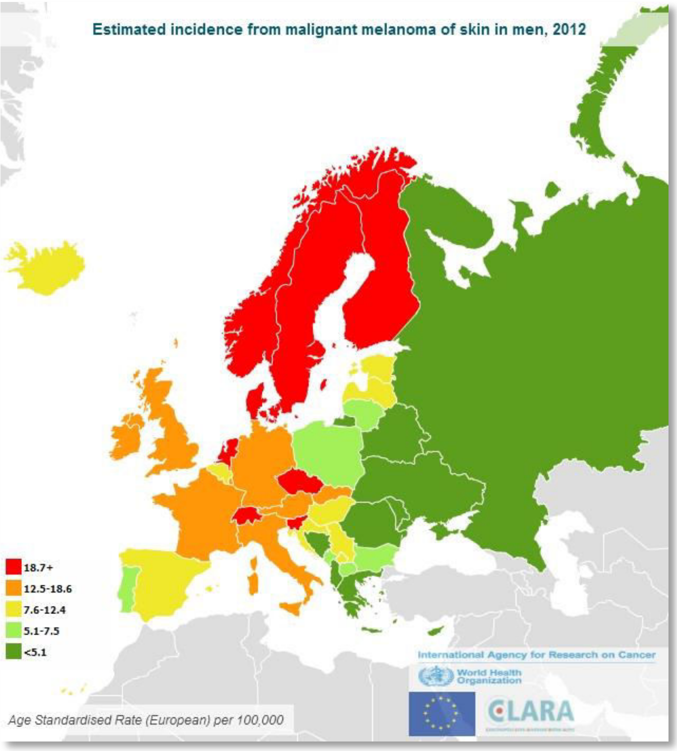

Figure S3. Estimated incidence from melanoma in men (2012).

Source: Steliarova-Foucher E, et al. European Cancer Observatory: Cancer Incidence, Mortality, Prevalence and Survival in Europe. Version 1.0 (September 2012), http://eco.iarc.fr/eucan/CancerSearch.aspx. All images use Creative Commons type licenses.

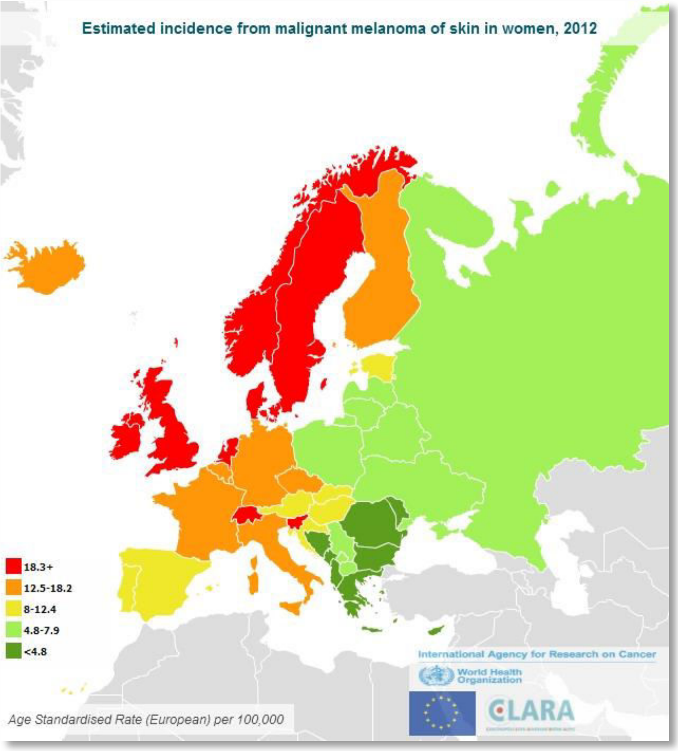

Figure S4. Estimated incidence from melanoma in women (2012).

Source: Steliarova-Foucher E, et al. European Cancer Observatory: Cancer Incidence, Mortality, Prevalence and Survival in Europe. Version 1.0 (September 2012), http://eco.iarc.fr/eucan/CancerSearch.aspx. All images use Creative Commons type licenses.

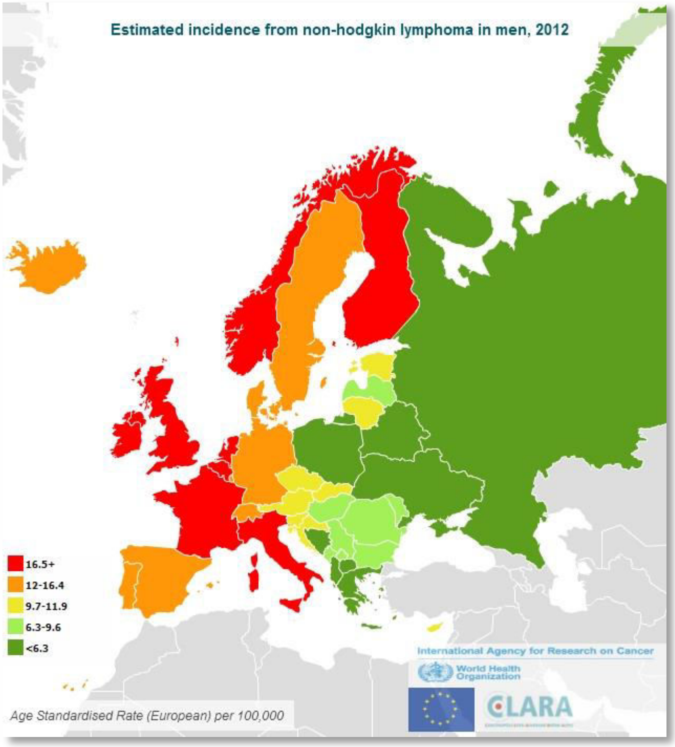

Figure S5. Estimated incidence from non-Hodgkin lymphoma in men (2012).

Source: Steliarova-Foucher E, et al. European Cancer Observatory: Cancer Incidence, Mortality, Prevalence and Survival in Europe. Version 1.0 (September 2012), http://eco.iarc.fr/eucan/CancerSearch.aspx. All images use Creative Commons type licenses.

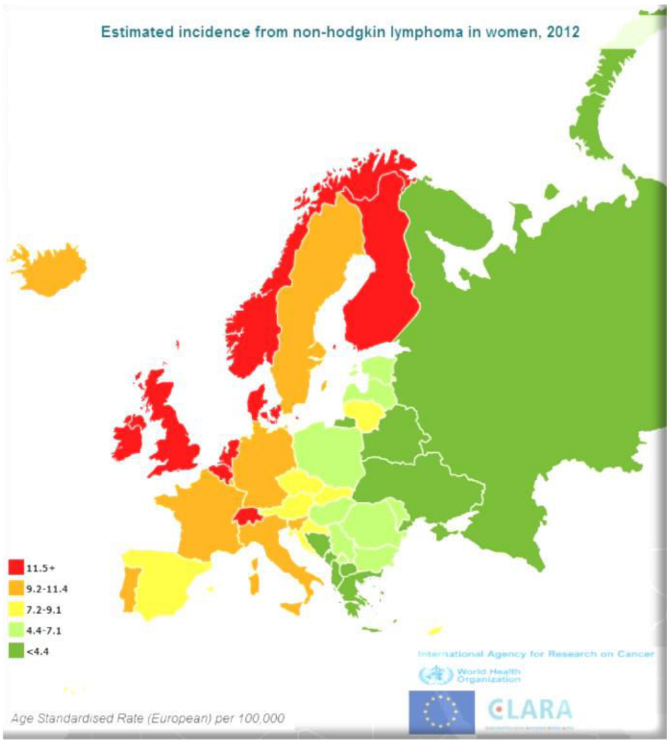

Figure S6. Estimated incidence from non-Hodgkin lymphoma in women (2012).

Source: Steliarova-Foucher E, et al. European Cancer Observatory: Cancer Incidence, Mortality, Prevalence and Survival in Europe. Version 1.0 (September 2012), http://eco.iarc.fr/eucan/CancerSearch.aspx. All images use Creative Commons type licenses.

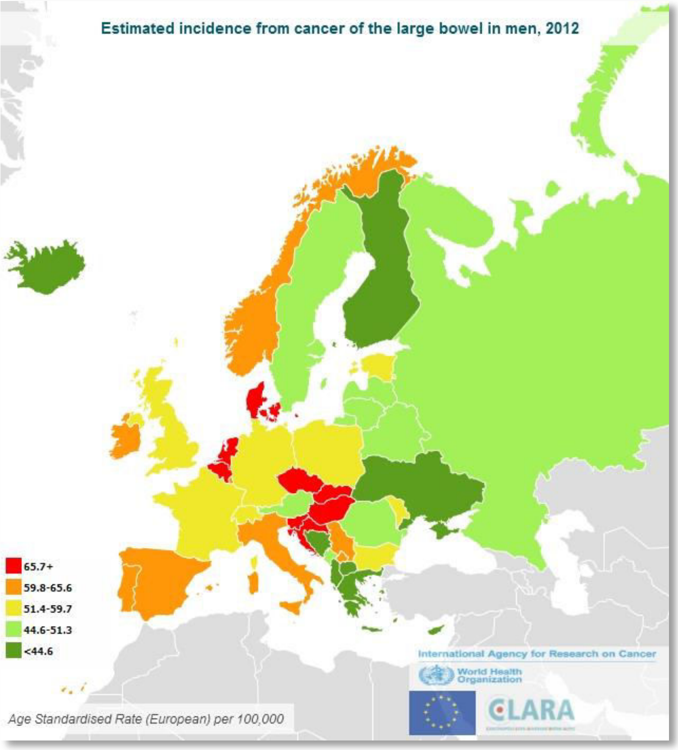

Figure S7. Estimated incidence from colorectal cancer in men (2012).

Source: Steliarova-Foucher E, et al. European Cancer Observatory: Cancer Incidence, Mortality, Prevalence and Survival in Europe. Version 1.0 (September 2012), http://eco.iarc.fr/eucan/CancerSearch.aspx. All images use Creative Commons type licenses.

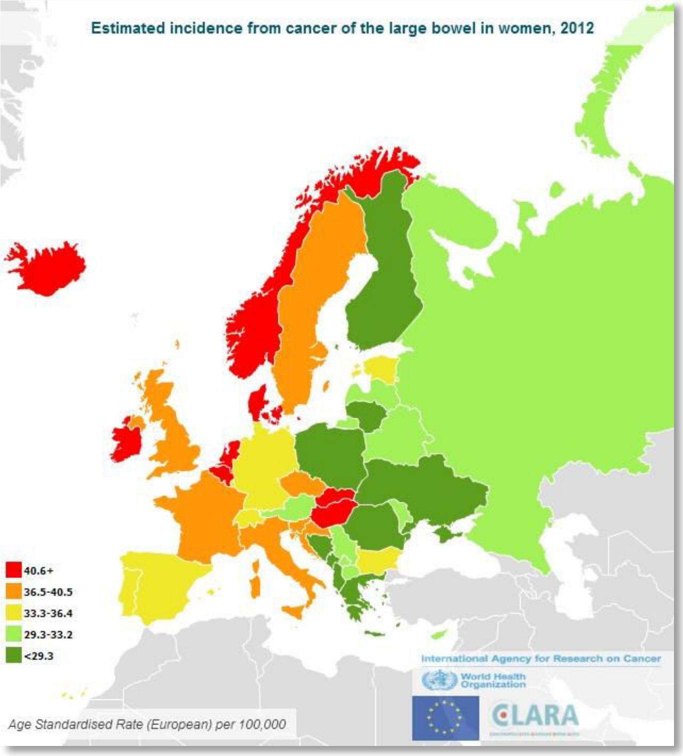

Figure S8. Estimated incidence from colorectal cancer in women (2012).

Source: Steliarova-Foucher E, et al. European Cancer Observatory: Cancer Incidence, Mortality, Prevalence and Survival in Europe. Version 1.0 (September 2012), http://eco.iarc.fr/eucan/CancerSearch.aspx. All images use Creative Commons type licenses.

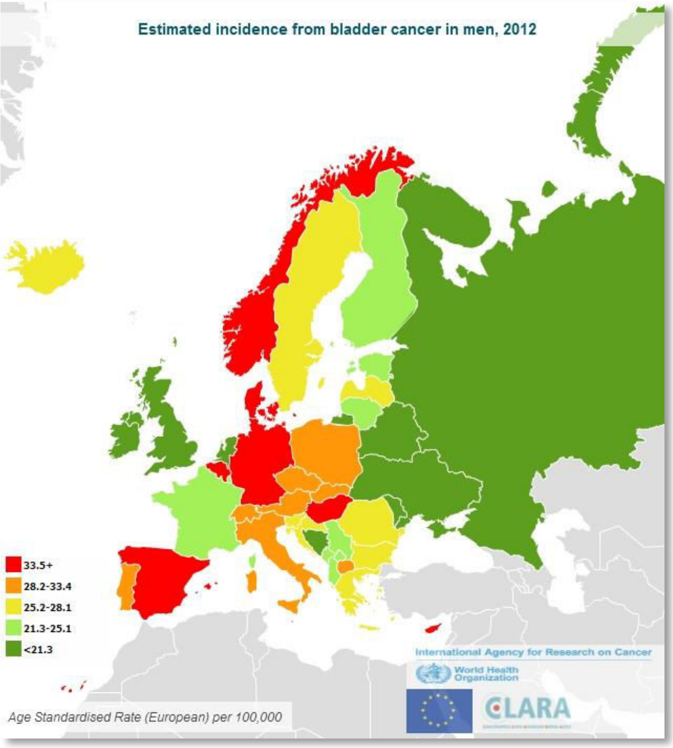

Figure S9. Estimated incidence from bladder cancer in men (2012).

Source: Steliarova-Foucher E, et al. European Cancer Observatory: Cancer Incidence, Mortality, Prevalence and Survival in Europe. Version 1.0 (September 2012), http://eco.iarc.fr/eucan/CancerSearch.aspx. All images use Creative Commons type licenses.

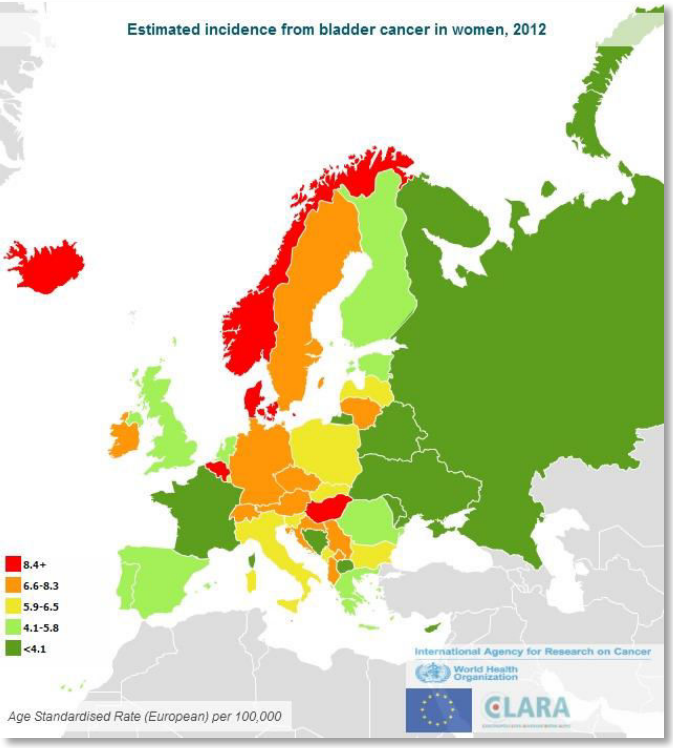

Figure S10. Estimated incidence from bladder cancer in women (2012).

Source: Steliarova-Foucher E, et al. European Cancer Observatory: Cancer Incidence, Mortality, Prevalence and Survival in Europe. Version 1.0 (September 2012), http://eco.iarc.fr/eucan/CancerSearch.aspx. All images use Creative Commons type licenses.

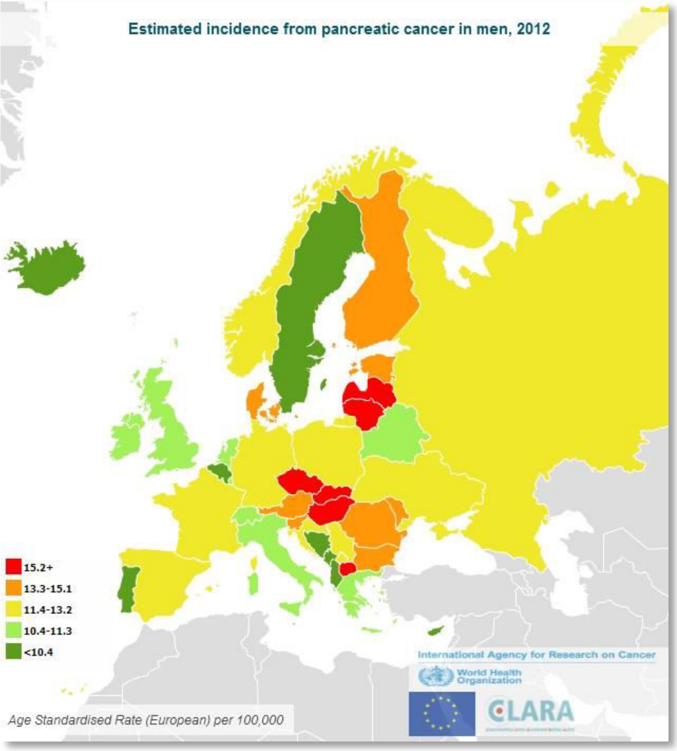

Figure S11. Estimated incidence from pancreatic cancer in men (2012).

Source: Steliarova-Foucher E, et al. European Cancer Observatory: Cancer Incidence, Mortality, Prevalence and Survival in Europe. Version 1.0 (September 2012), http://eco.iarc.fr/eucan/CancerSearch.aspx. All images use Creative Commons type licenses.

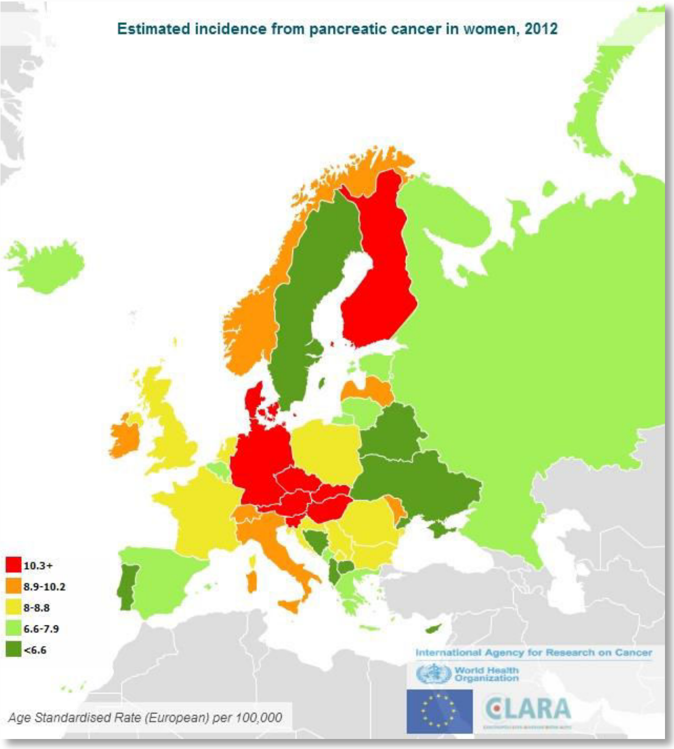

Figure S12. Estimated incidence from pancreatic cancer in women (2012).

Source: Steliarova-Foucher E, et al. European Cancer Observatory: Cancer Incidence, Mortality, Prevalence and Survival in Europe. Version 1.0 (September 2012), http://eco.iarc.fr/eucan/CancerSearch.aspx. All images use Creative Commons type licenses.

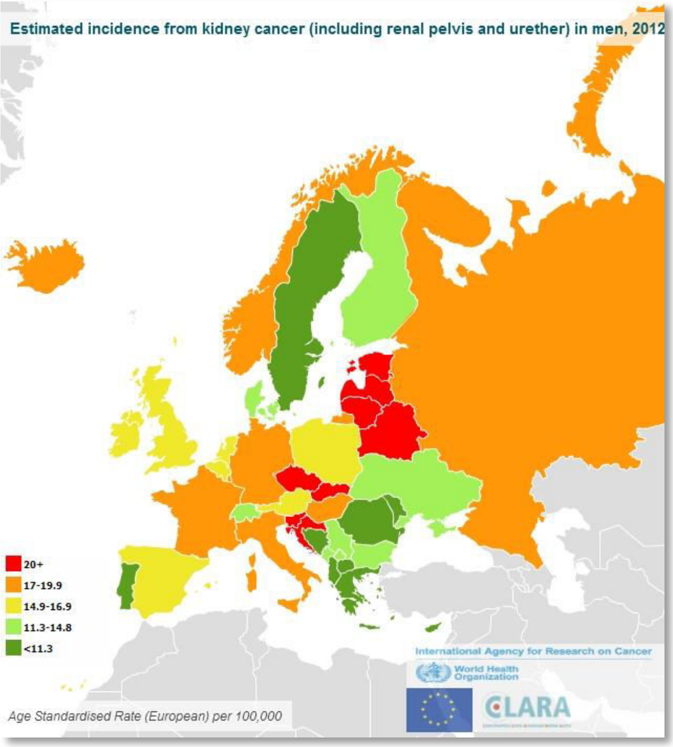

Figure S13. Estimated incidence from kidney cancer in men (2012).

Source: Steliarova-Foucher E, et al. European Cancer Observatory: Cancer Incidence, Mortality, Prevalence and Survival in Europe. Version 1.0 (September 2012), http://eco.iarc.fr/eucan/CancerSearch.aspx. All images use Creative Commons type licenses.

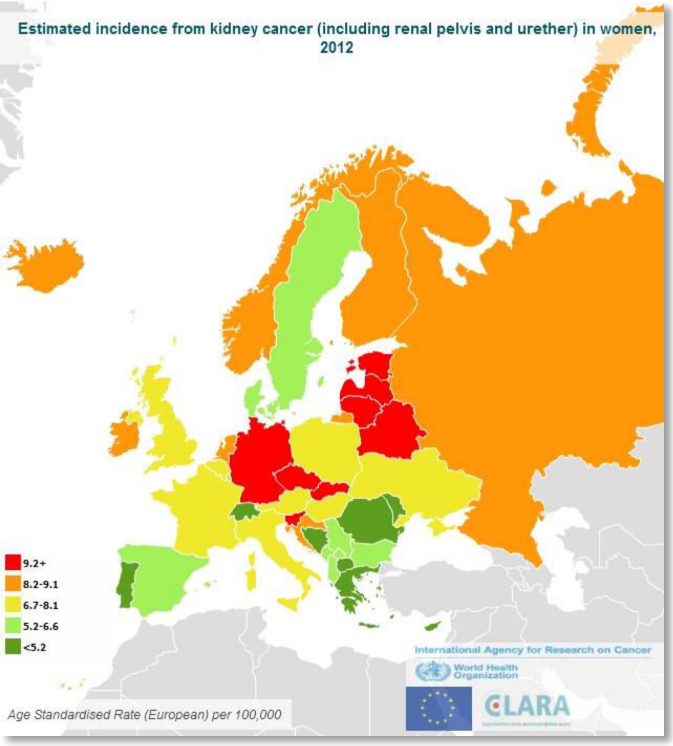

Figure S14. Estimated incidence from kidney cancer in women (2012).

Source: Steliarova-Foucher E, et al. European Cancer Observatory: Cancer Incidence, Mortality, Prevalence and Survival in Europe. Version 1.0 (September 2012), http://eco.iarc.fr/eucan/CancerSearch.aspx. All images use Creative Commons type licenses.

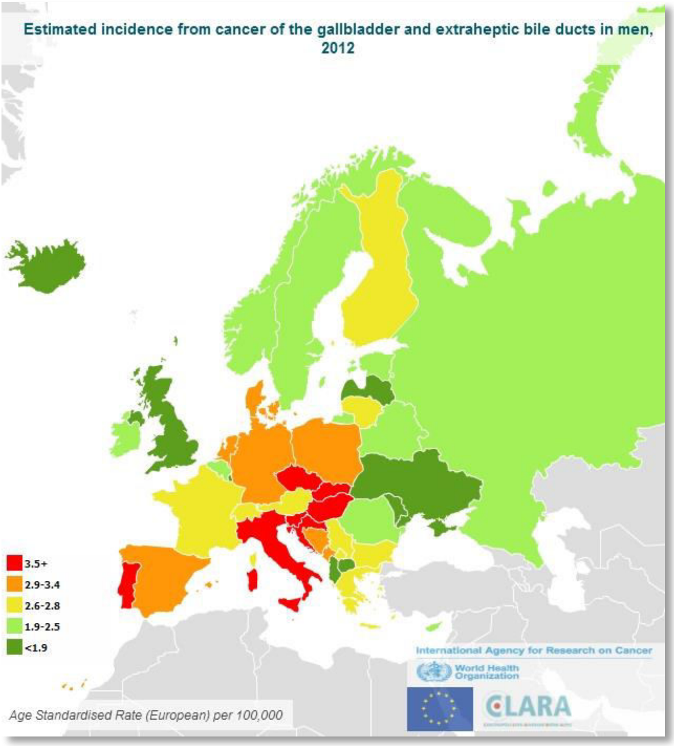

Figure S15. Estimated incidence from gallbladder cancer in men (2012).
Source: Steliarova-Foucher E, et al. European Cancer Observatory: Cancer Incidence, Mortality, Prevalence and Survival in Europe. Version 1.0 (September 2012), http://eco.iarc.fr/eucan/CancerSearch.aspx. All images use Creative Commons type licenses.

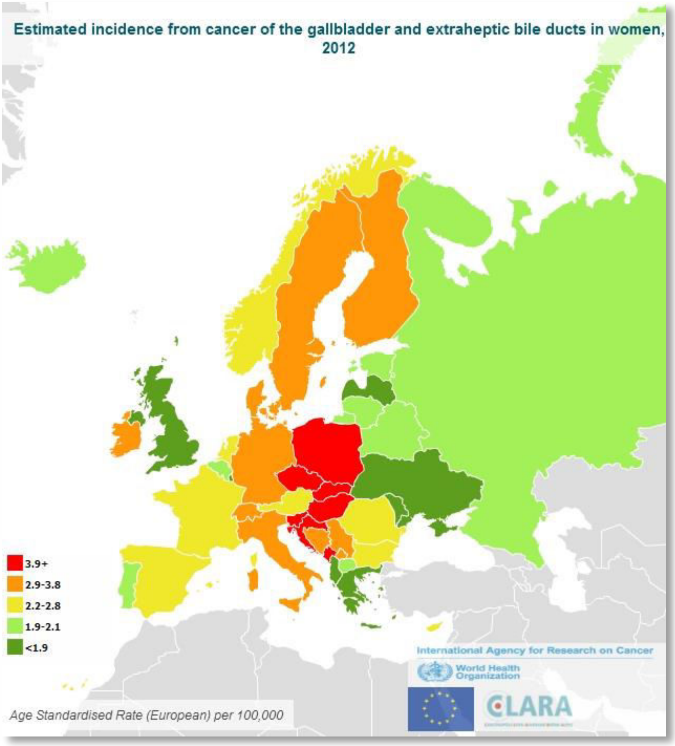

Figure S16. Estimated incidence from gallbladder cancer in women (2012).
Source: Steliarova-Foucher E, et al. European Cancer Observatory: Cancer Incidence, Mortality, Prevalence and Survival in Europe. Version 1.0 (September 2012), http://eco.iarc.fr/eucan/CancerSearch.aspx. All images use Creative Commons type licenses.

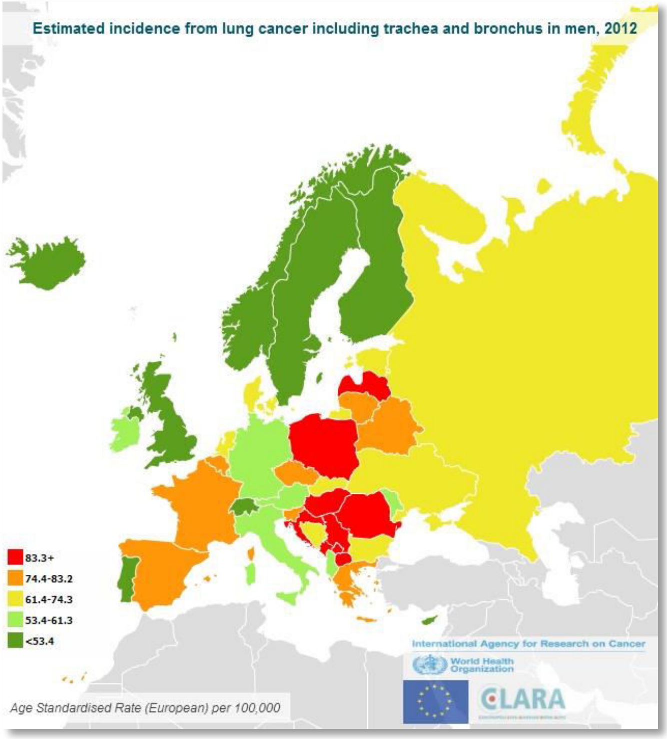

Figure S17. Estimated incidence from lung cancer in men (2012).
Source: Steliarova-Foucher E, et al. European Cancer Observatory: Cancer Incidence, Mortality, Prevalence and Survival in Europe. Version 1.0 (September 2012), http://eco.iarc.fr/eucan/CancerSearch.aspx. All images use Creative Commons type licenses.

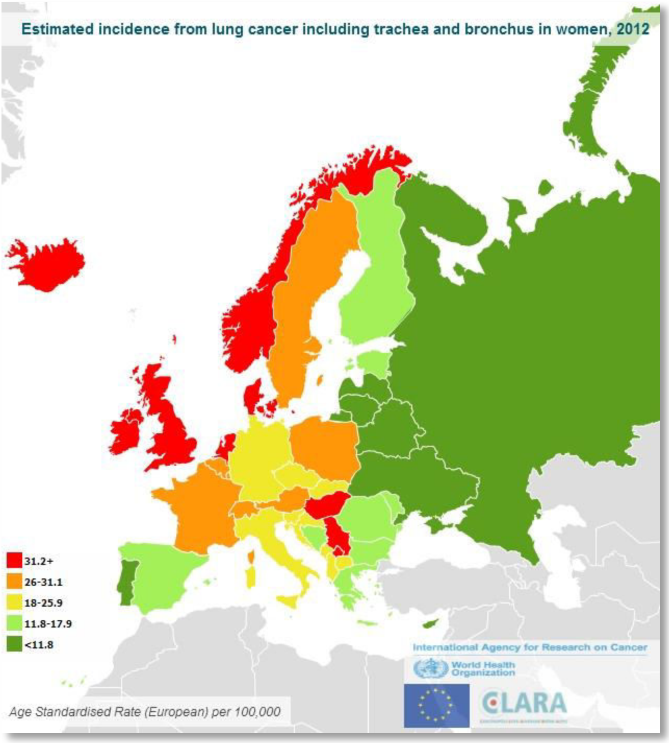

Figure S18. Estimated incidence from lung cancer in women (2012).
Source: Steliarova-Foucher E, et al. European Cancer Observatory: Cancer Incidence, Mortality, Prevalence and Survival in Europe. Version 1.0 (September 2012), http://eco.iarc.fr/eucan/CancerSearch.aspx. All images use Creative Commons type licenses.

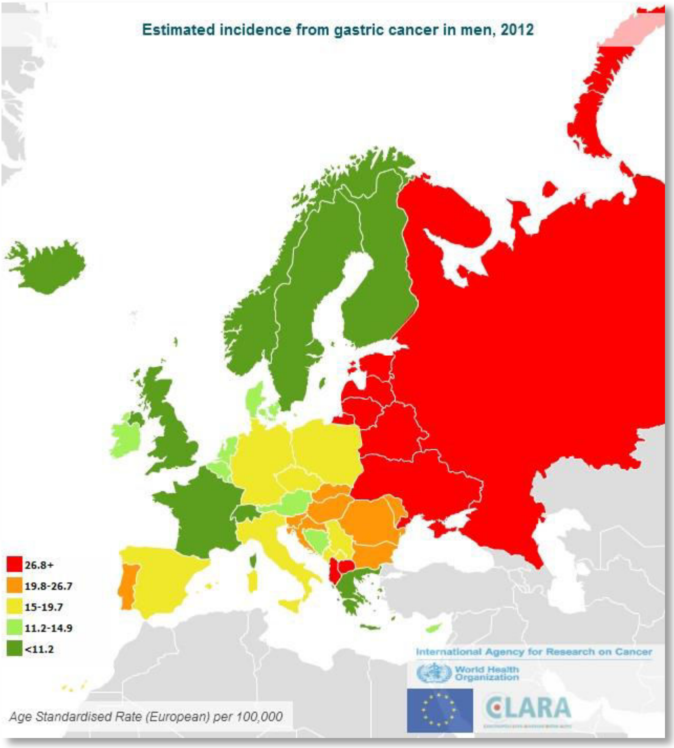

Figure S19. Estimated incidence from stomach cancer in men (2012).
Source: Steliarova-Foucher E, et al. European Cancer Observatory: Cancer Incidence, Mortality, Prevalence and Survival in Europe. Version 1.0 (September 2012), http://eco.iarc.fr/eucan/CancerSearch.aspx. All images use Creative Commons type licenses.

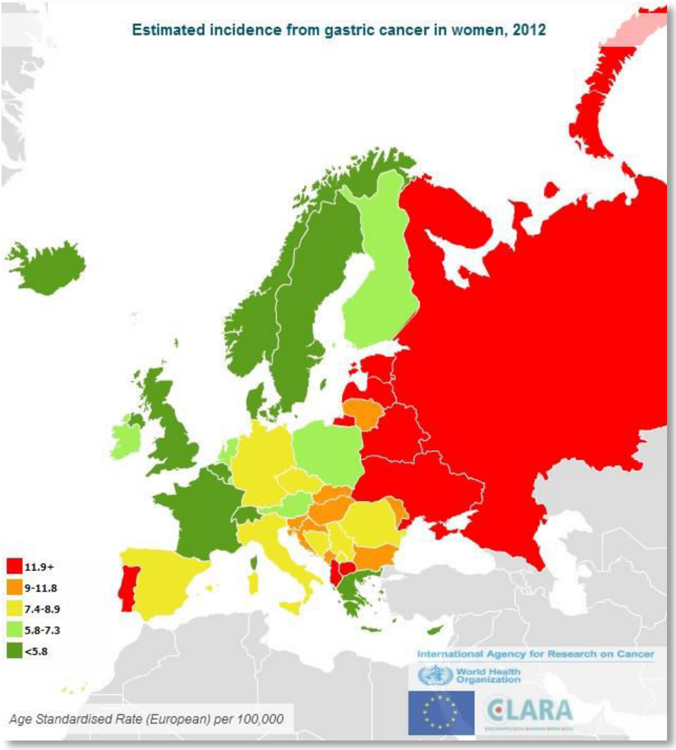

Figure S20. Estimated incidence from stomach cancer in women (2012).
Source: Steliarova-Foucher E, et al. European Cancer Observatory: Cancer Incidence, Mortality, Prevalence and Survival in Europe. Version 1.0 (September 2012), http://eco.iarc.fr/eucan/CancerSearch.aspx. All images use Creative Commons type licenses.

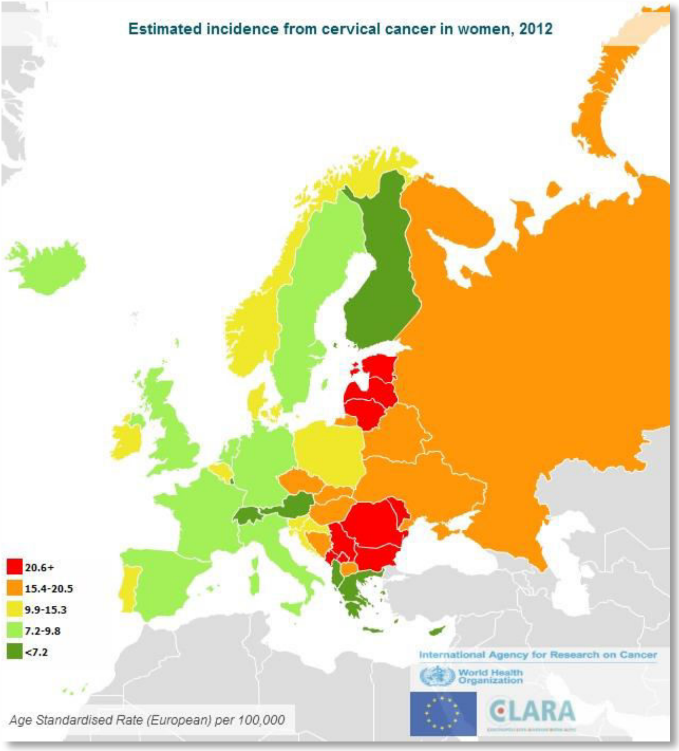

Figure S21. Estimated incidence from cervical cancer in women (2012).
Source: Steliarova-Foucher E, et al. European Cancer Observatory: Cancer Incidence, Mortality, Prevalence and Survival in Europe. Version 1.0 (September 2012), http://eco.iarc.fr/eucan/CancerSearch.aspx. All images use Creative Commons type licenses.

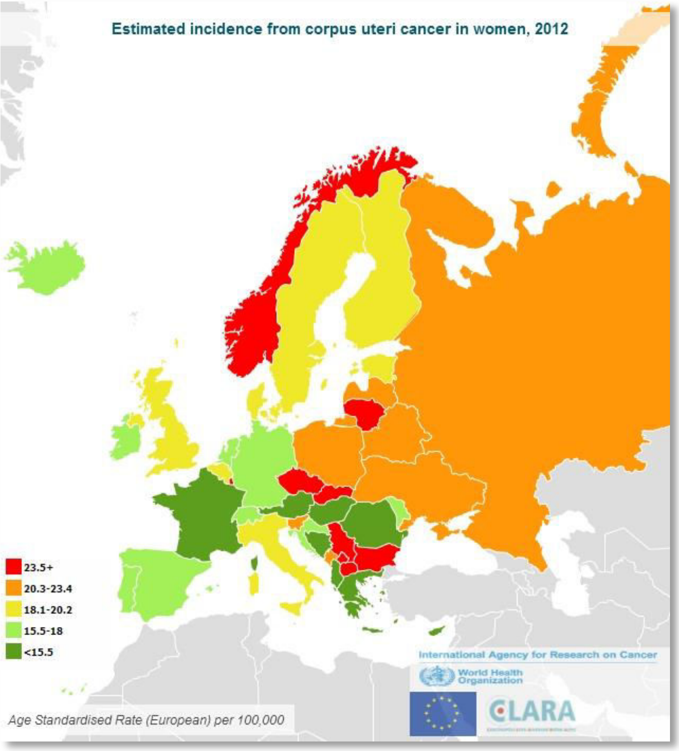

Figure S22. Estimated incidence from corpus uteri cancer in women (2012).
Source: Steliarova-Foucher E, et al. European Cancer Observatory: Cancer Incidence, Mortality, Prevalence and Survival in Europe. Version 1.0 (September 2012), http://eco.iarc.fr/eucan/CancerSearch.aspx. All images use Creative Commons type licenses.

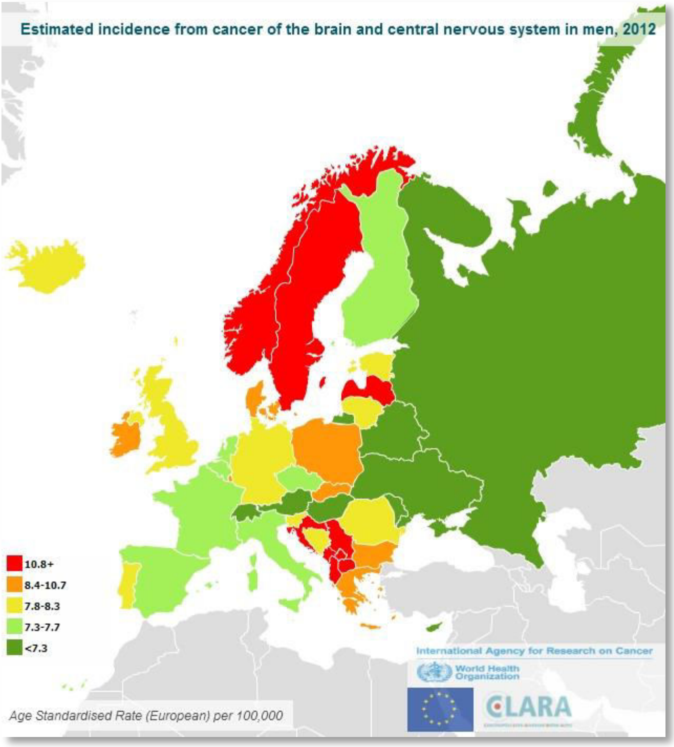

Figure S23. Estimated incidence from brain cancer in men (2012),
Source: Steliarova-Foucher E, et al. European Cancer Observatory: Cancer Incidence, Mortality, Prevalence and Survival in Europe. Version 1.0 (September 2012), http://eco.iarc.fr/eucan/CancerSearch.aspx. All images use Creative Commons type licenses.

.

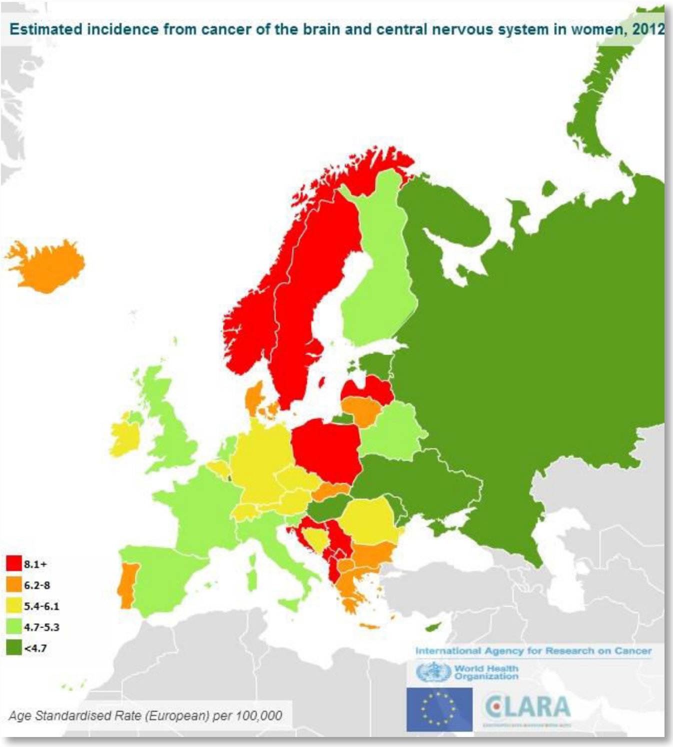

Figure S24. Estimated incidence from brain cancer in women (2012),
Source: Steliarova-Foucher E, et al. European Cancer Observatory: Cancer Incidence, Mortality, Prevalence and Survival in Europe. Version 1.0 (September 2012), http://eco.iarc.fr/eucan/CancerSearch.aspx. All images use Creative Commons type licenses.

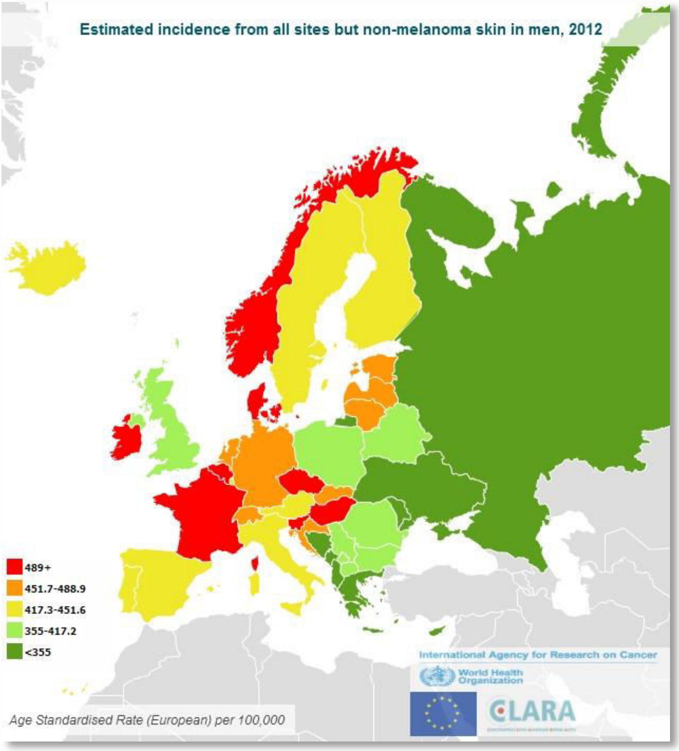

Figure S25. Estimated incidence from all sites (total incidence) excluding non-melanoma skin in men (2012).

Source: Steliarova-Foucher E, et al. European Cancer Observatory: Cancer Incidence, Mortality, Prevalence and Survival in Europe. Version 1.0 (September 2012), http://eco.iarc.fr/eucan/CancerSearch.aspx. All images use Creative Commons type licenses.

.

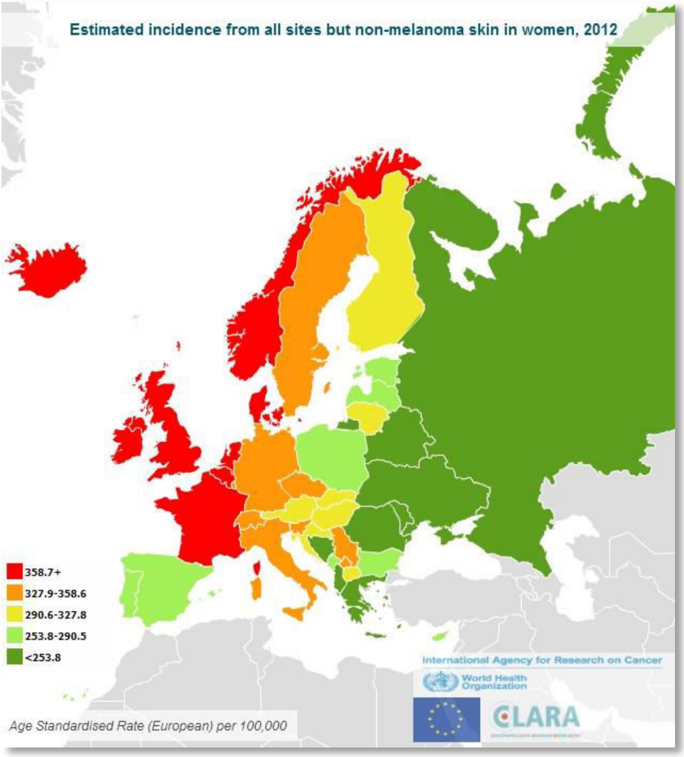

Figure S26. Estimated incidence from all sites (total incidence) excluding non-melanoma skin in women (2012).

Source: Steliarova-Foucher E, et al. European Cancer Observatory: Cancer Incidence, Mortality, Prevalence and Survival in Europe. Version 1.0 (September 2012), http://eco.iarc.fr/eucan/CancerSearch.aspx. All images use Creative Commons type licenses.

.

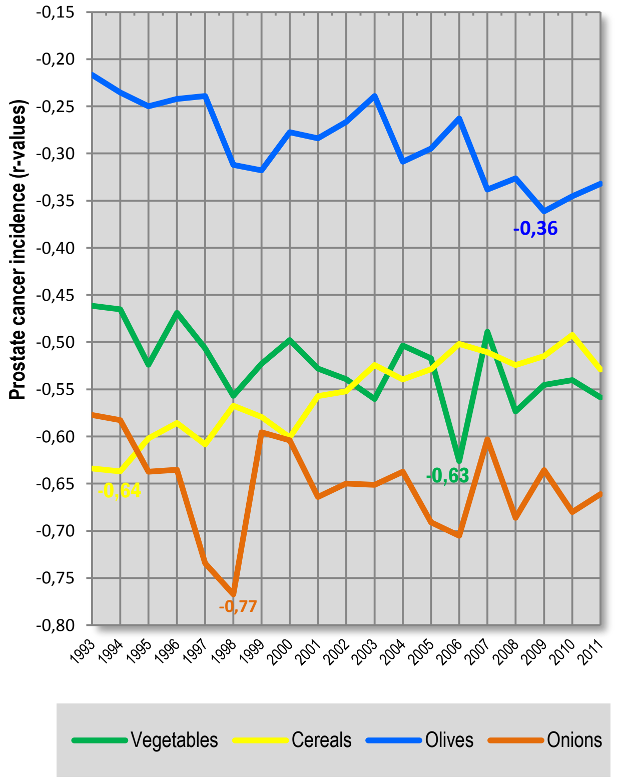

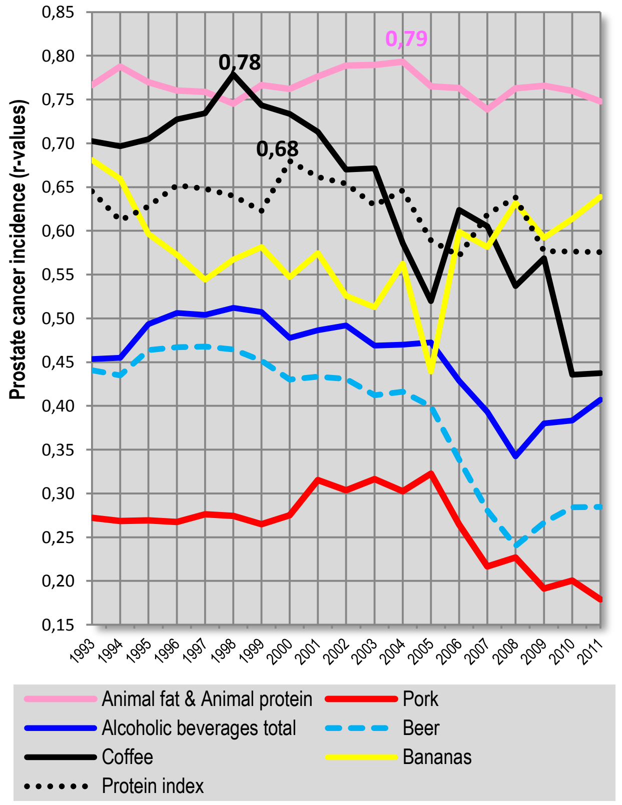

Figure S27. Temporal changes in the relationship between 4 negative correlates of prostate cancer incidence.

Figure S28. Temporal changes in the relationship between 7 positive correlates of prostate cancer incidence.

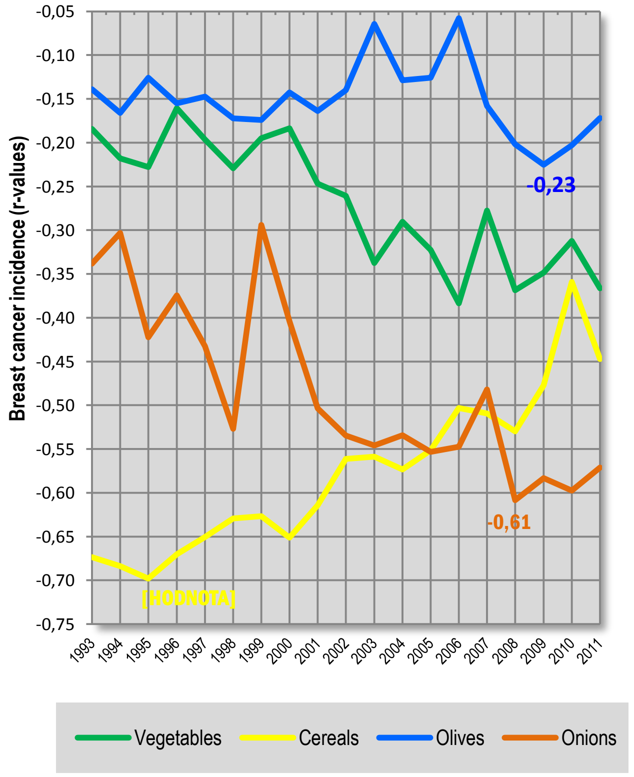

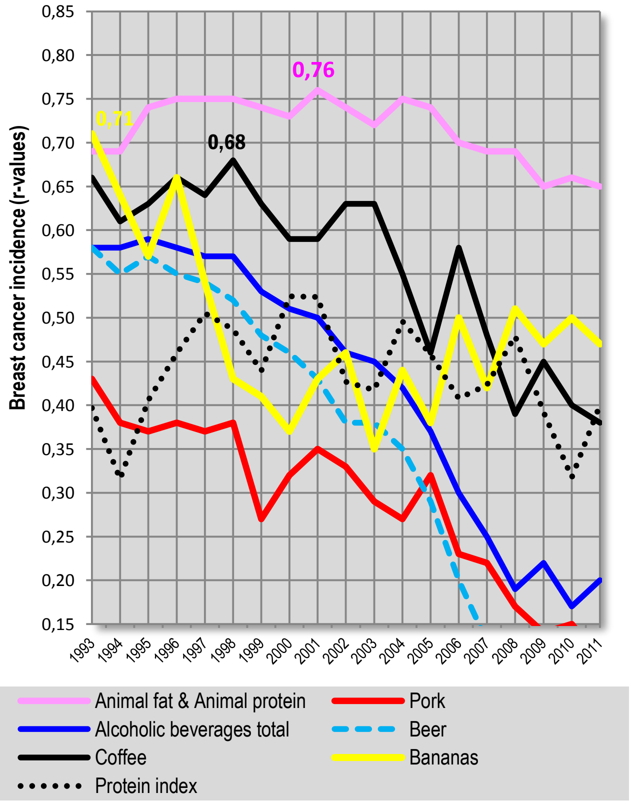

Figure S29. Temporal changes in the relationship between 4 negative correlates of breast cancer incidence.

Figure S30. Temporal changes in the relationship between 7 positive correlates of breast cancer incidence.

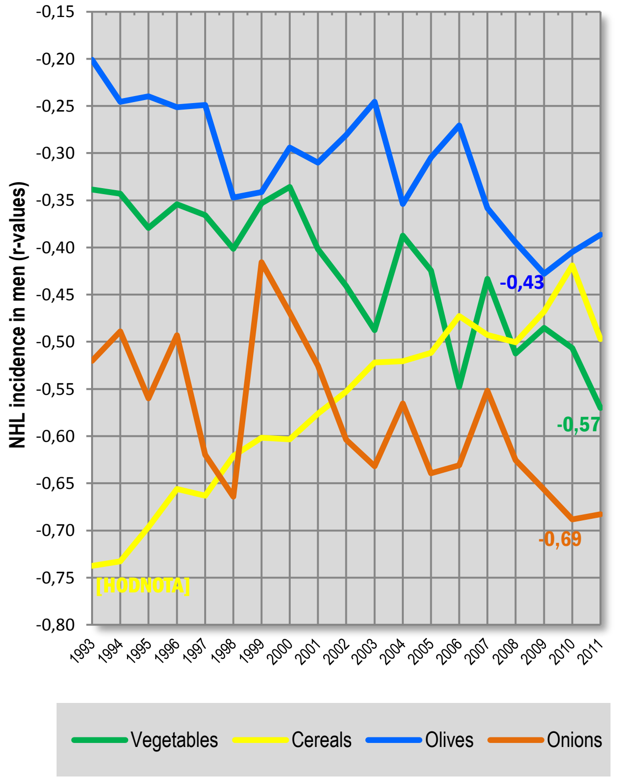

Figure S31. Temporal changes in the relationship between 4 negative correlates of NHL (non-Hodgkin lymphoma) incidence in men.

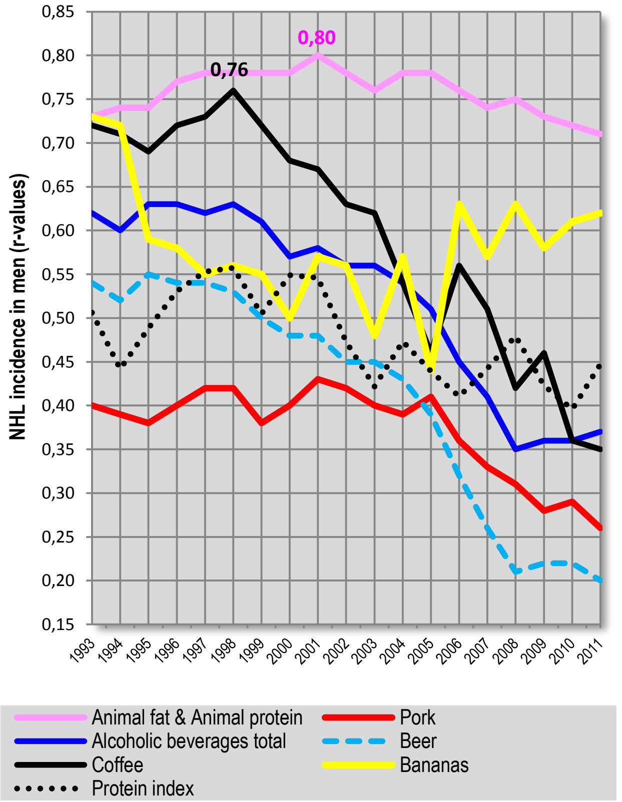

Figure S32. Temporal changes in the relationship between 7 positive correlates of NHL (non-Hodgkin lymphoma) incidence in men.

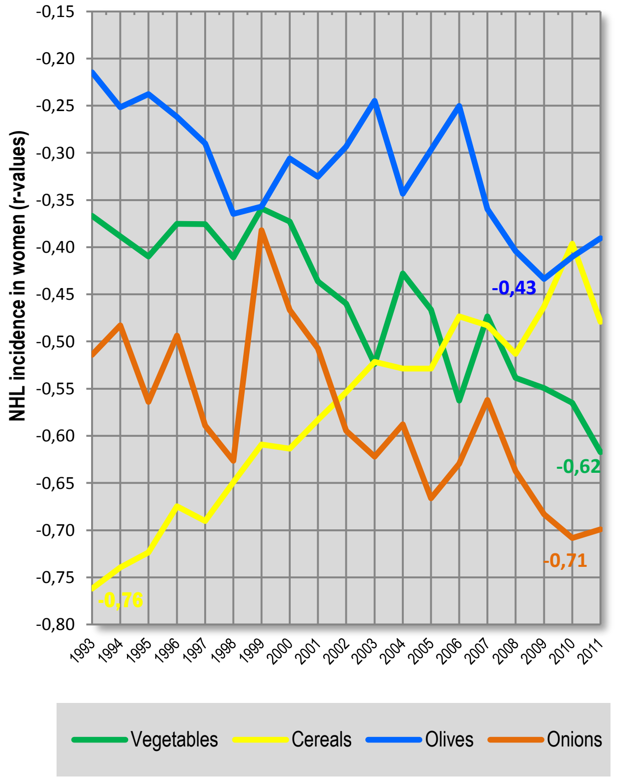

Figure S33. Temporal changes in the relationship between 4 negative correlates of NHL (non-Hodgkin lymphoma) incidence in women.

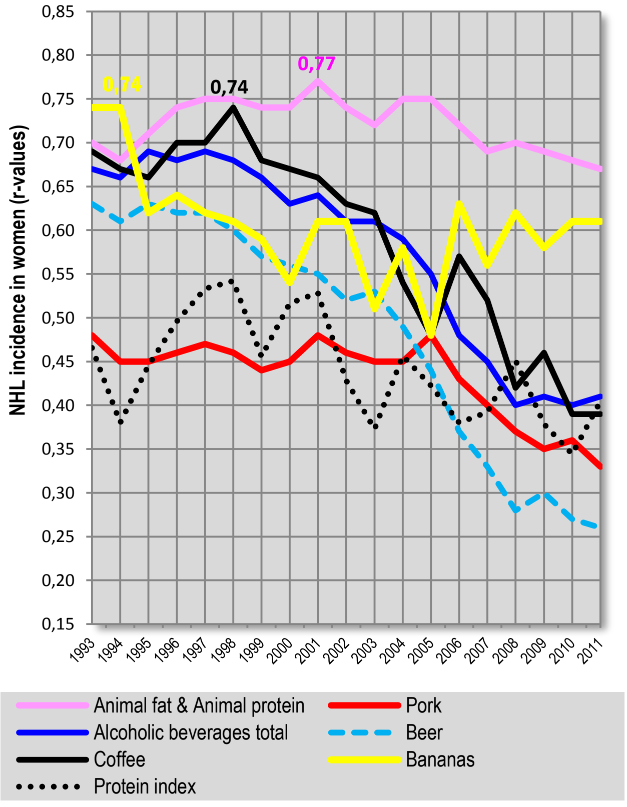

Figure S34. Temporal changes in the relationship between 7 positive correlates of NHL (non-Hodgkin lymphoma) incidence in women.

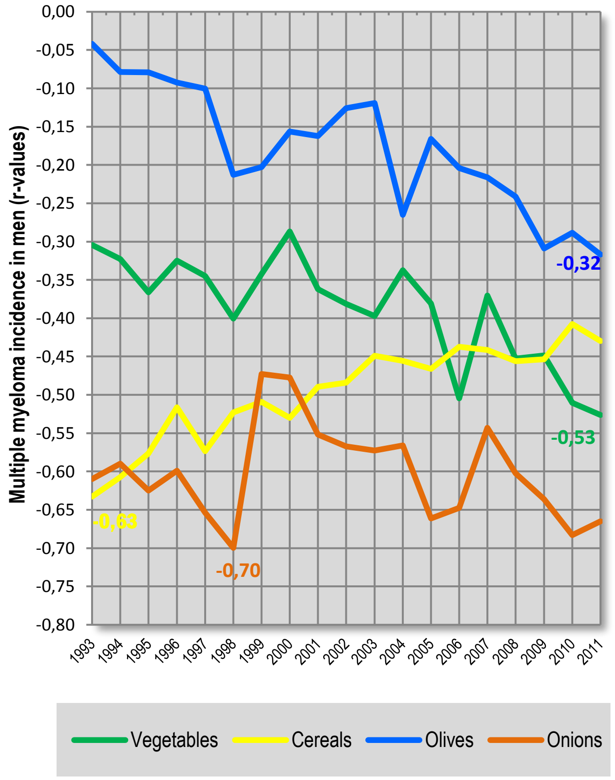

Figure S35. Temporal changes in the relationship between 4 negative correlates of multiple myeloma incidence in men.

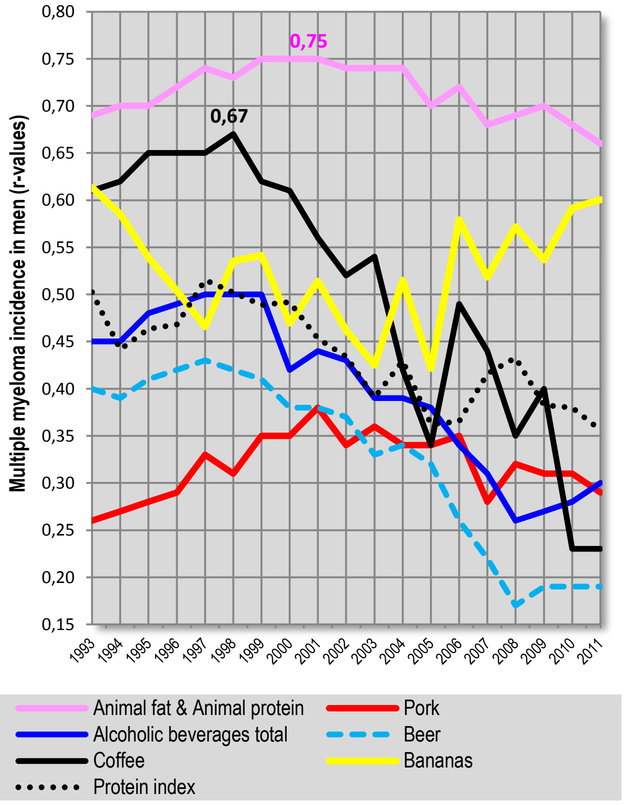

Figure S36. Temporal changes in the relationship between 7 positive correlates of multiple myeloma incidence in men.

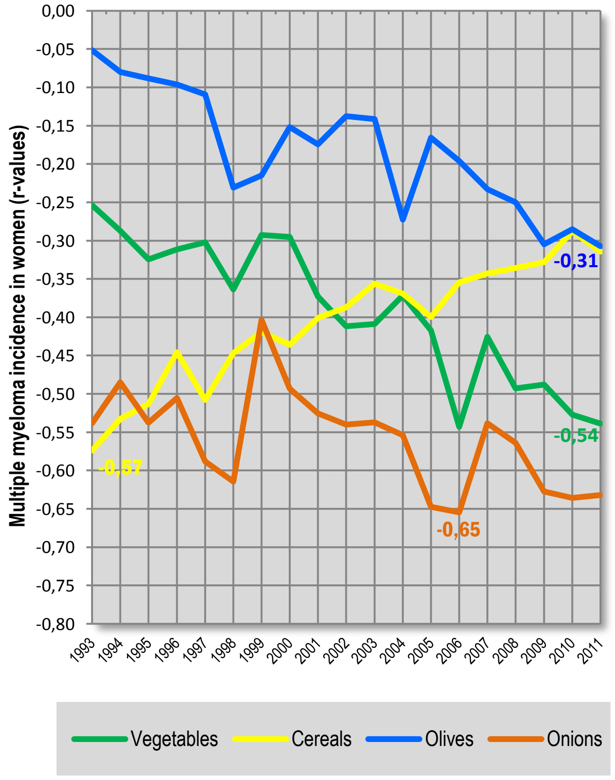

Figure S37. Temporal changes in the relationship between 4 negative correlates of multiple myeloma incidence in women.

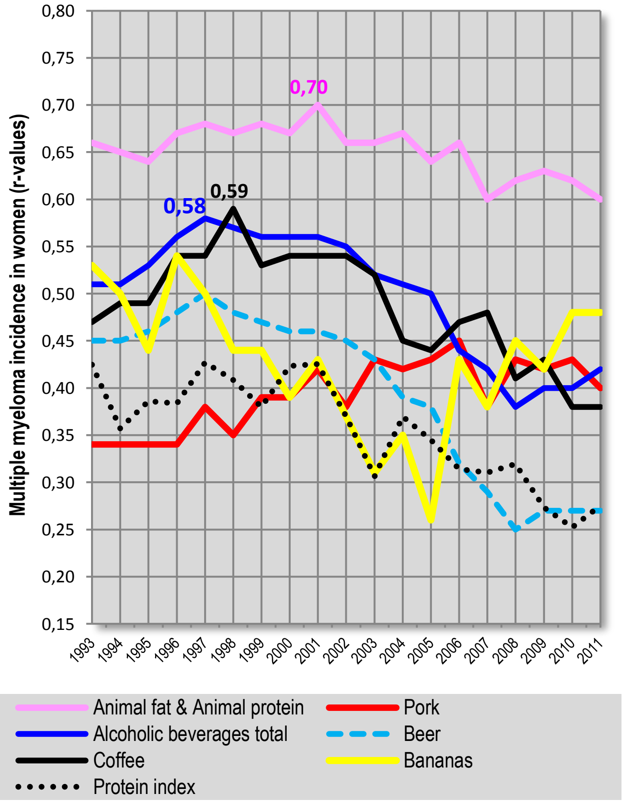

Figure S38. Temporal changes in the relationship between 7 positive correlates of multiple myeloma incidence in women.

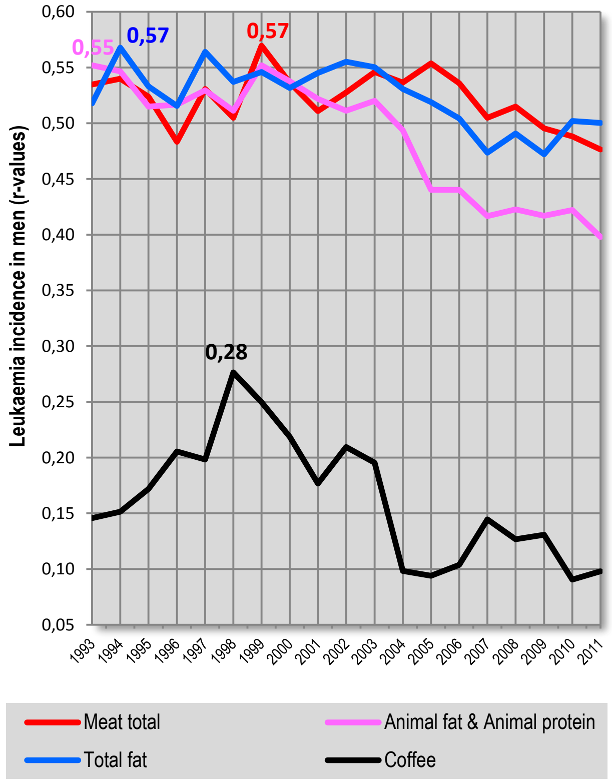

Figure S39. Temporal changes in the relationship between 4 positive correlates of leukaemia incidence in men.

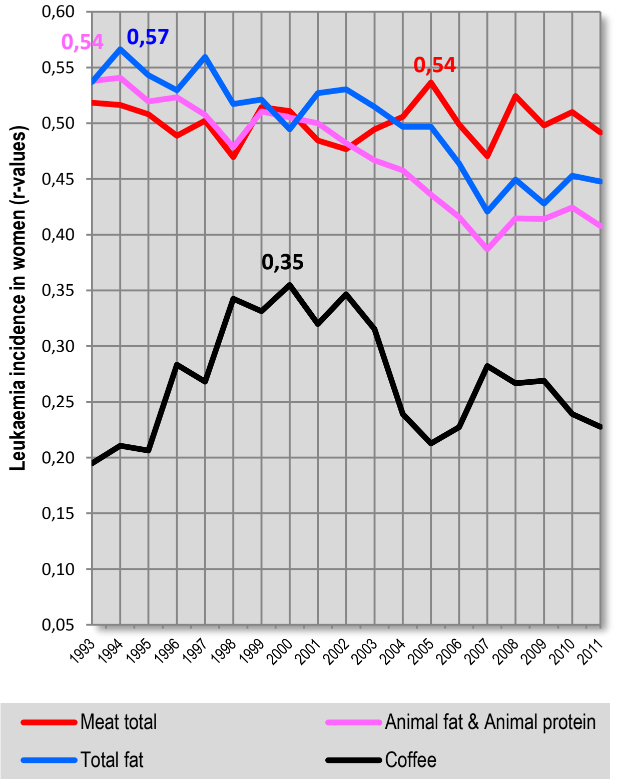

Figure S40. Temporal changes in the relationship between 4 positive correlates of leukaemia incidence in women.

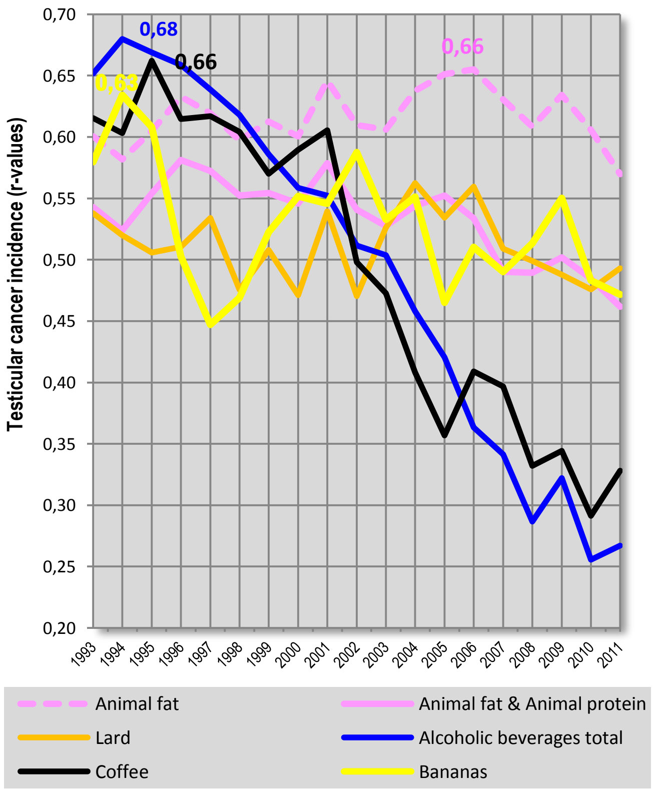

Figure S41. Temporal changes in the relationship between 6 positive correlates of testicular cancer incidence.

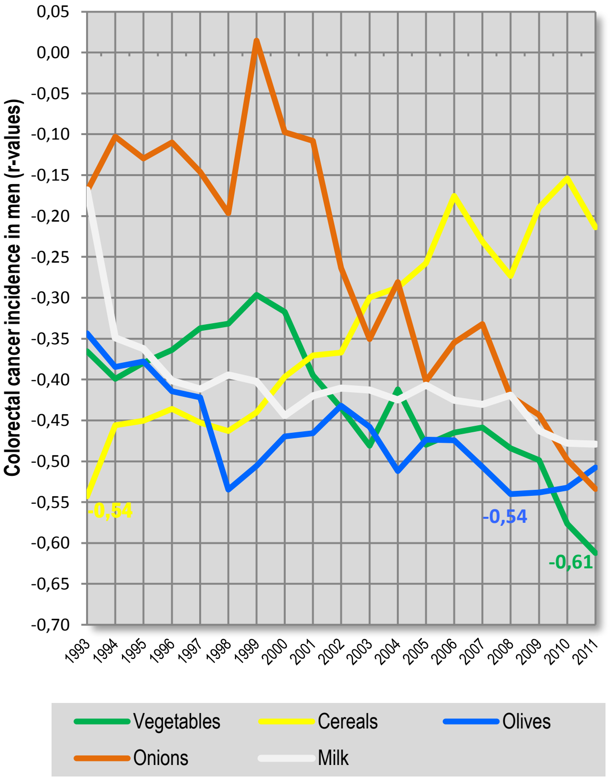

Figure S42. Temporal changes in the relationship between 5 negative correlates of colorectal cancer incidence in men.

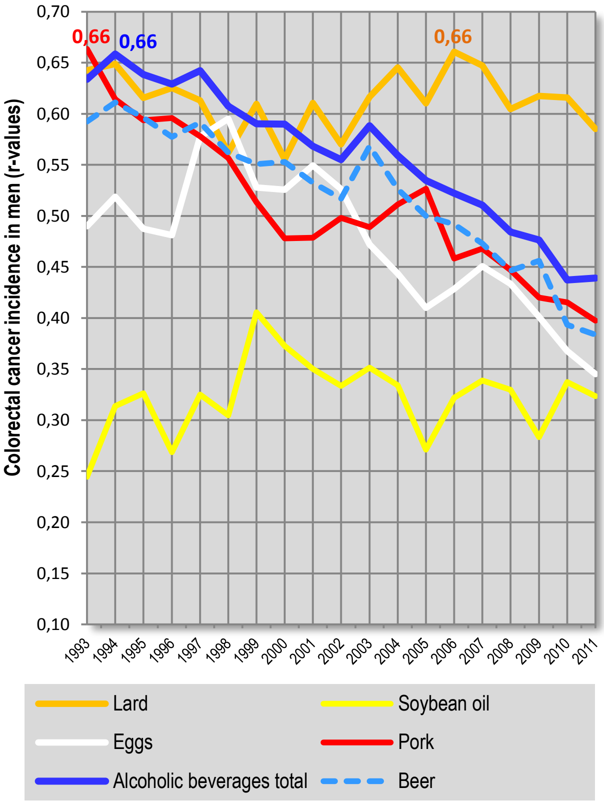

Figure S43. Temporal changes in the relationship between 6 positive correlates of colorectal cancer incidence in men.

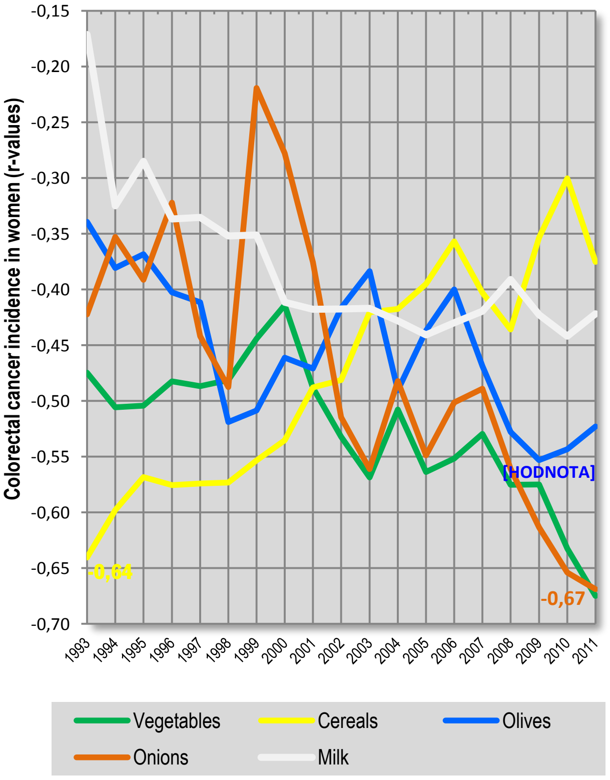

Figure S44. Temporal changes in the relationship between 5 negative correlates of colorectal cancer incidence in women.

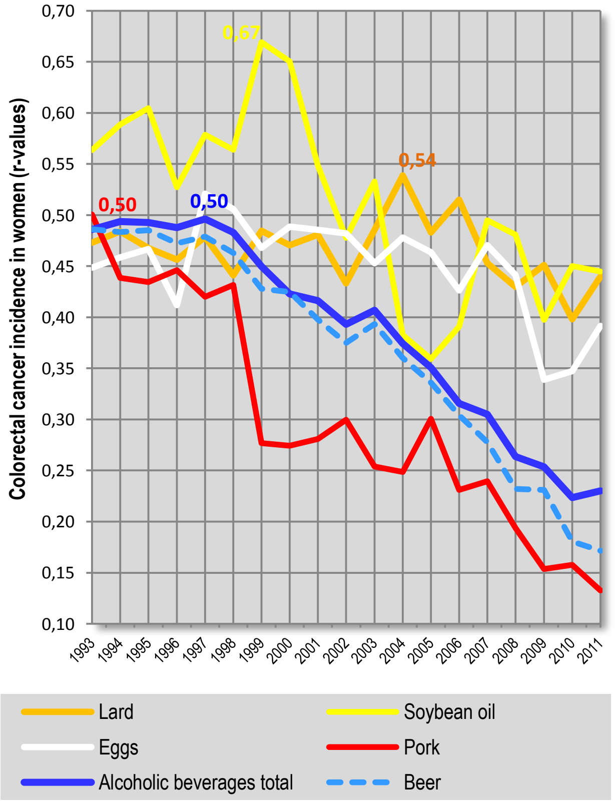

Figure S45. Temporal changes in the relationship between 6 positive correlates of colorectal cancer incidence in women.

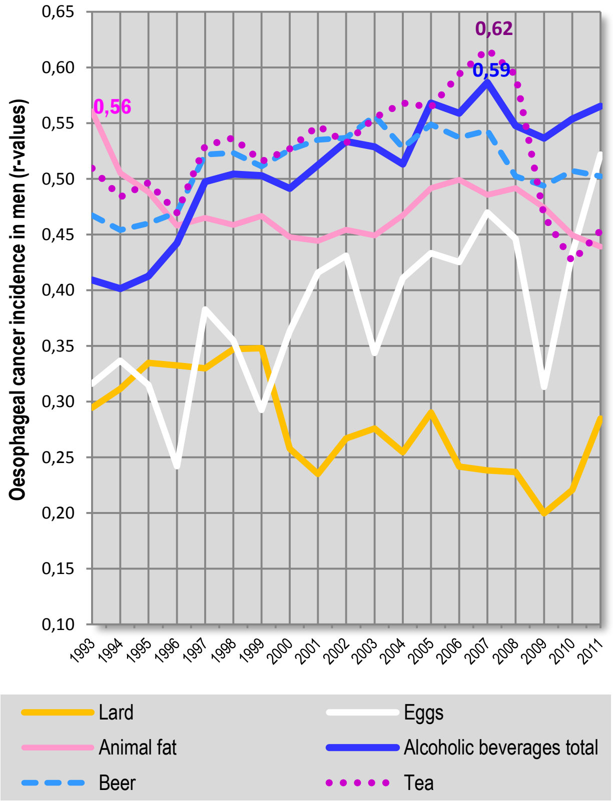

Figure S46. Temporal changes in the relationship between 6 correlates of esophageal cancer incidence in men.

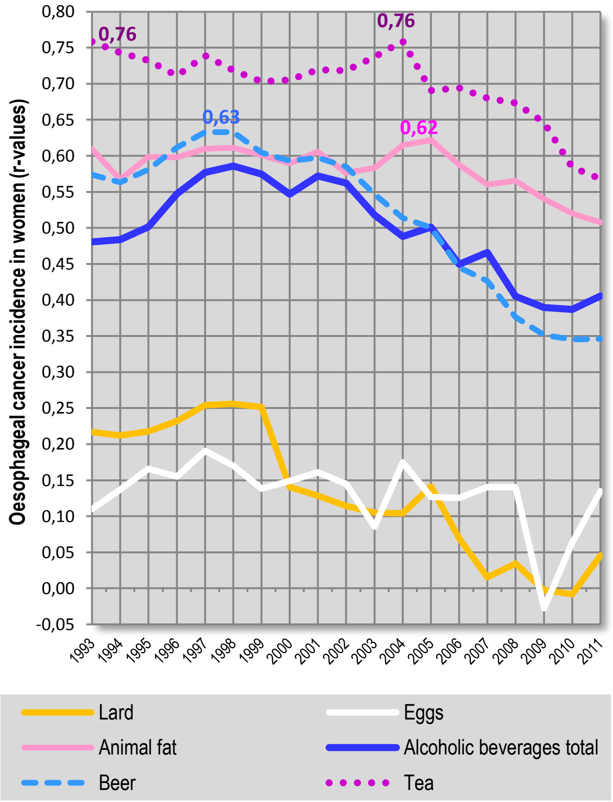

Figure S47. Temporal changes in the relationship between 6 correlates of esophageal cancer incidence in women.

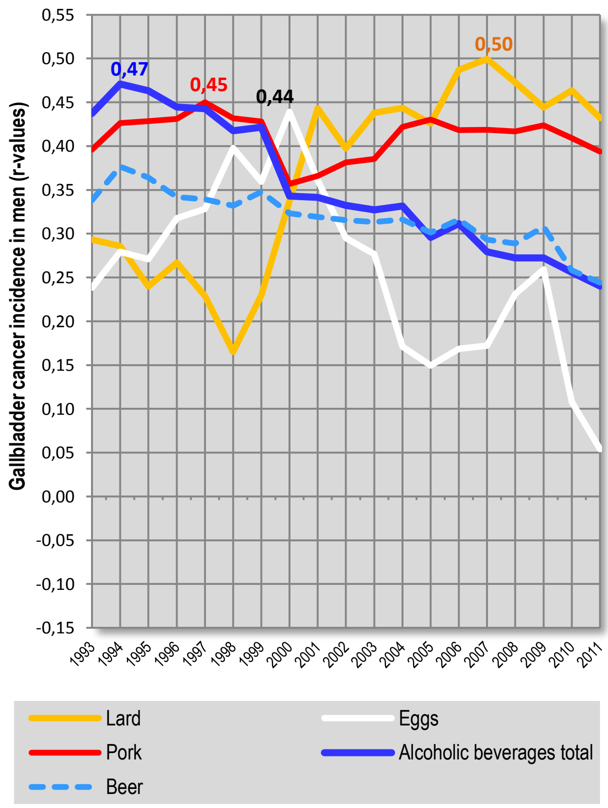

Figure S48. Temporal changes in the relationship between 5 correlates of gallbladder cancer incidence in men.

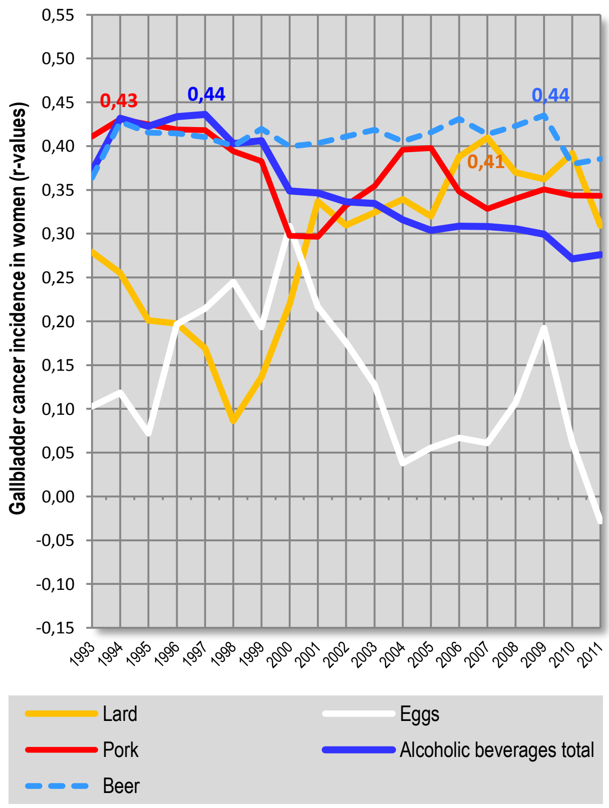

Figure S49. Temporal changes in the relationship between 5 correlates of gallbladder cancer incidence in women.

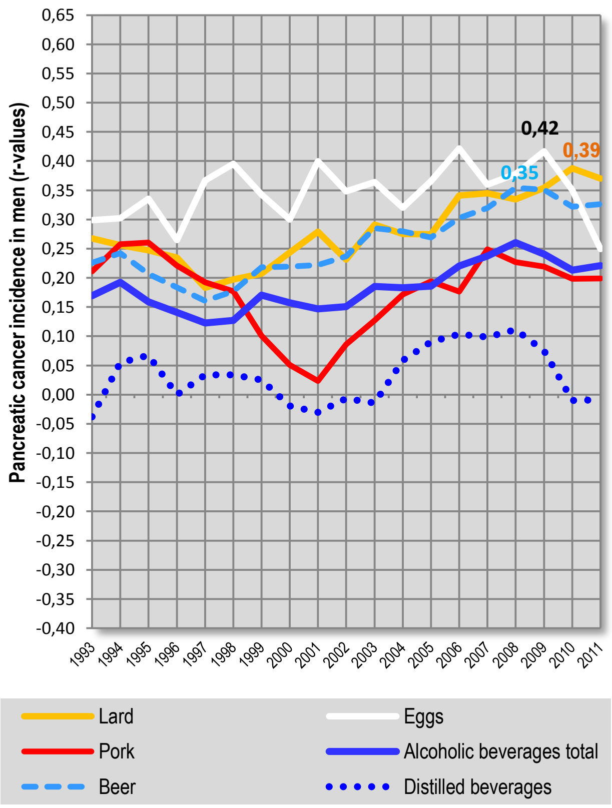

Figure S50. Temporal changes in the relationship between 6 correlates of pancreatic cancer incidence in men.

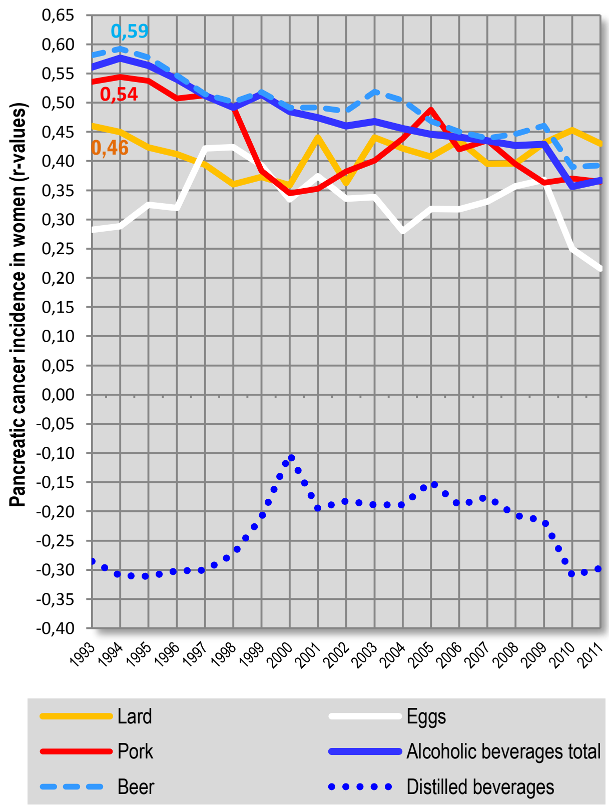

Figure S51. Temporal changes in the relationship between 6 correlates of pancreatic cancer incidence in women.

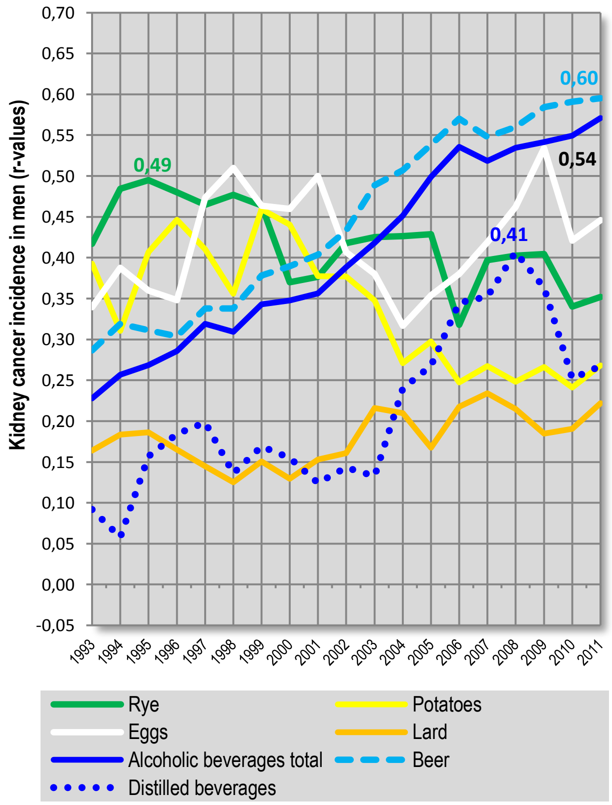

Figure S52. Temporal changes in the relationship between 7 correlates of kidney cancer incidence in men.

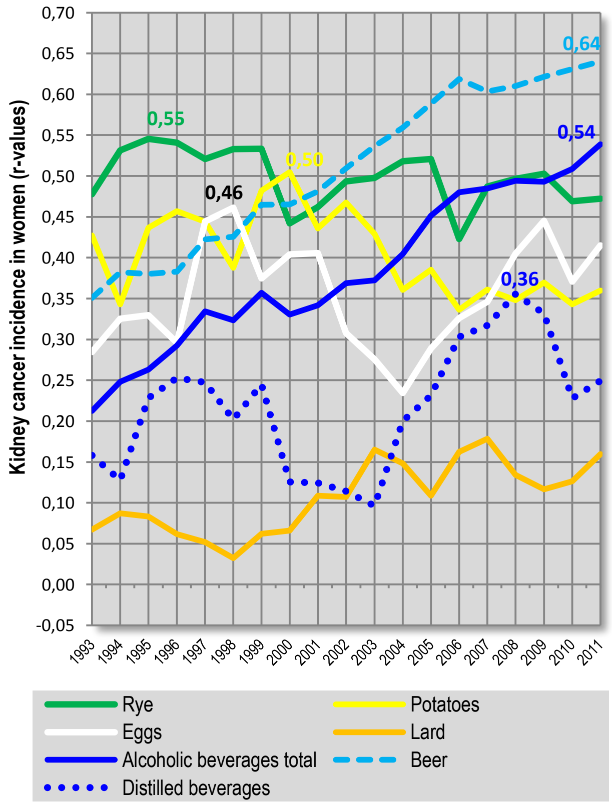

Figure S53. Temporal changes in the relationship between 7 correlates of kidney cancer incidence in women.

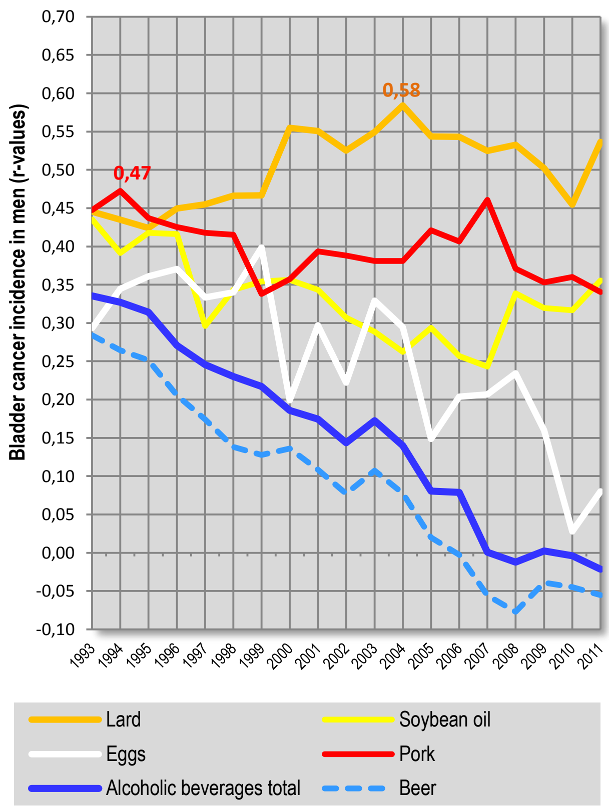

Figure S54. Temporal changes in the relationship between 6 correlates of bladder cancer incidence in men.

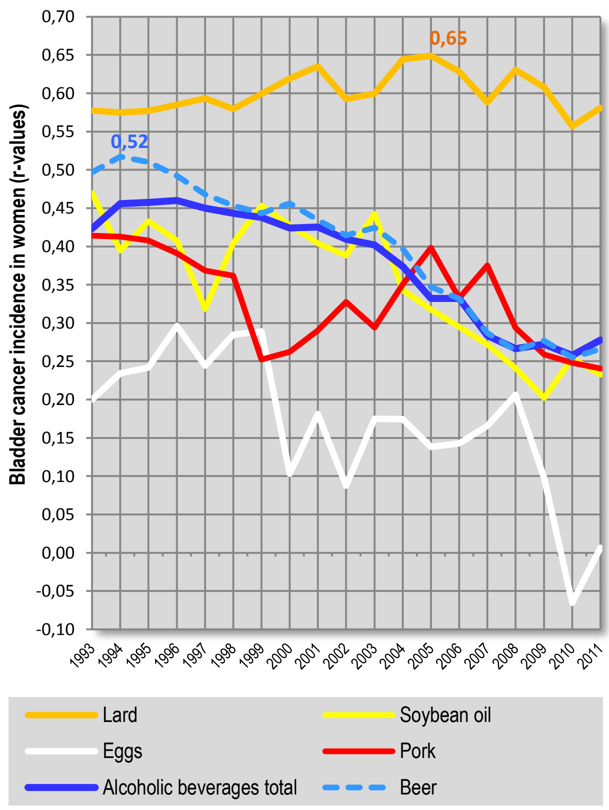

Figure S55. Temporal changes in the relationship between 6 correlates of bladder cancer incidence in women.

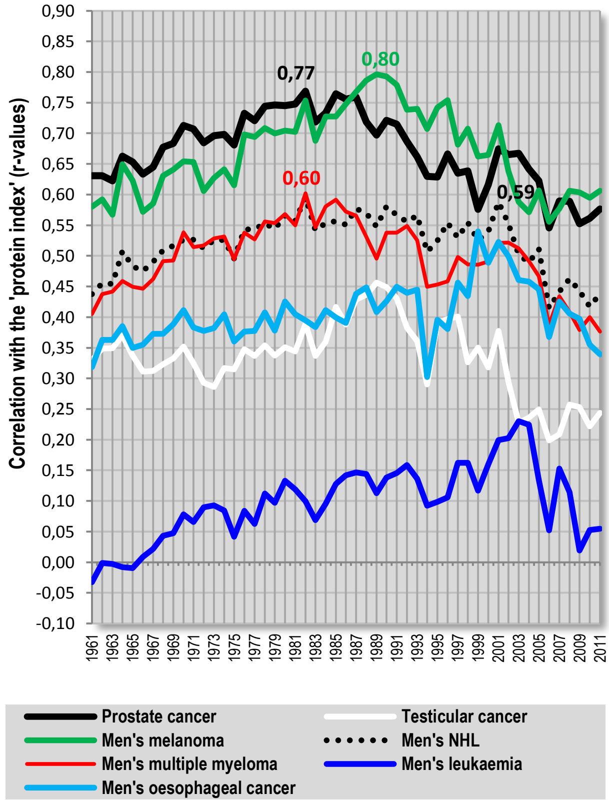

Figure S56. Temporal changes in the relationship between the ‚protein index‘ and 7 types of men's cancer (a sample of 24 countries).
Abbreviation: NHL = non-Hodgkin's lymphoma.

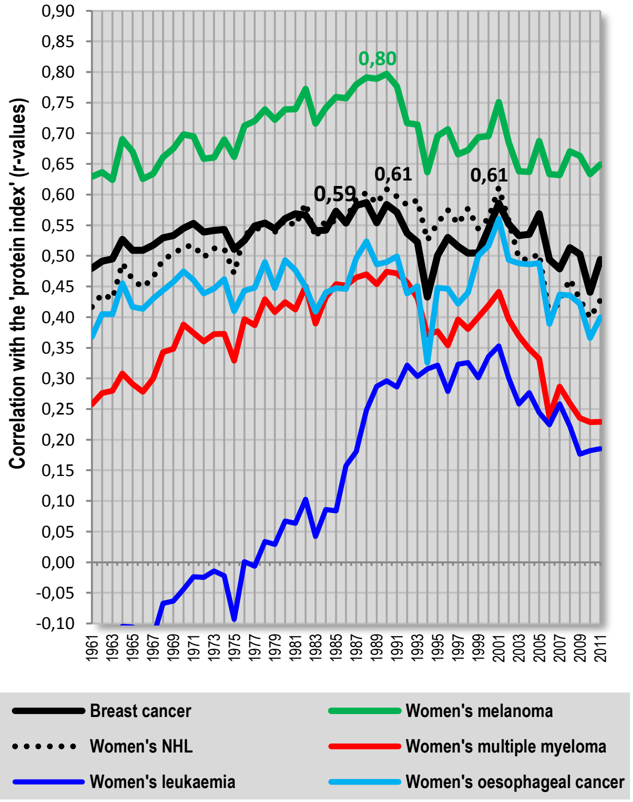

Figure S57. Temporal changes in the relationship between the ‚protein index‘ and 6 types of women's cancer (a sample of 24 countries). *Abbreviation:* NHL = non-Hodgkin's lymphoma.

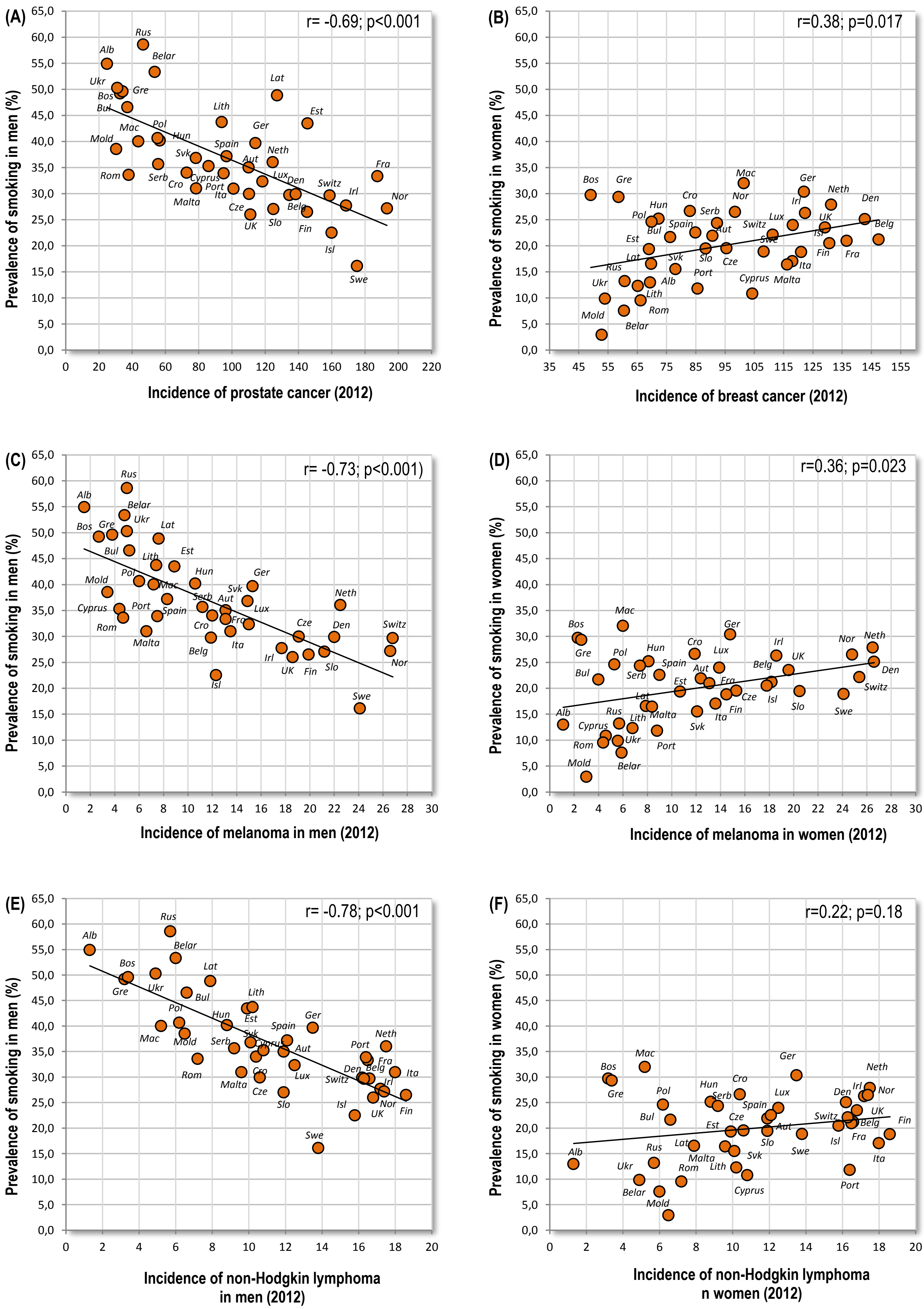

Figures S58A-S58F. Relationship between smoking and the incidence of several cancer types in men and women.

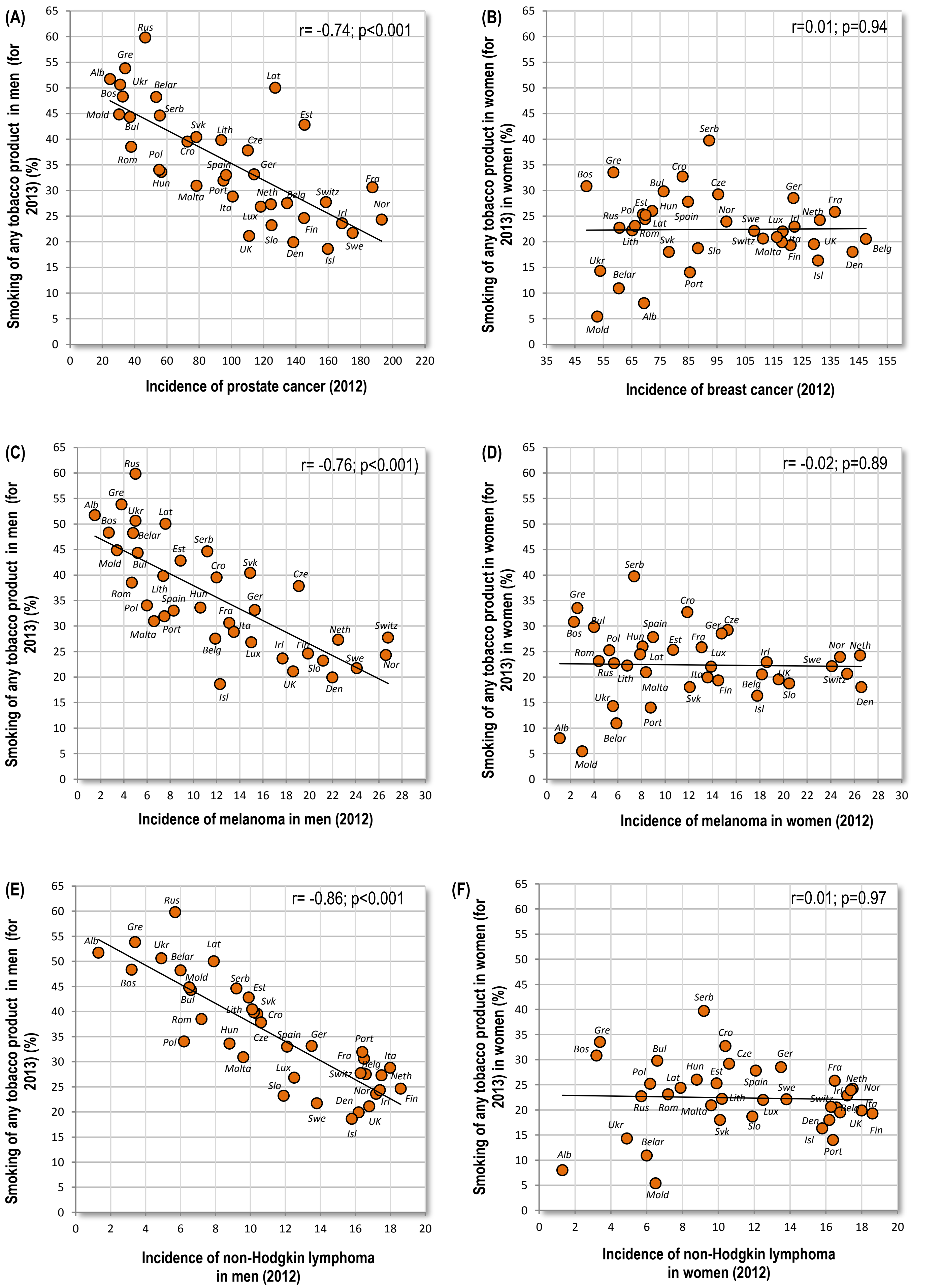

Figures S59A-S59F. Relationship between the actual smoking of any tobacco product (WHO, for 2013) and the incidence of several cancer types in men and women (a sample of 36 countries, without Austria, Cyprus and Macedonia).

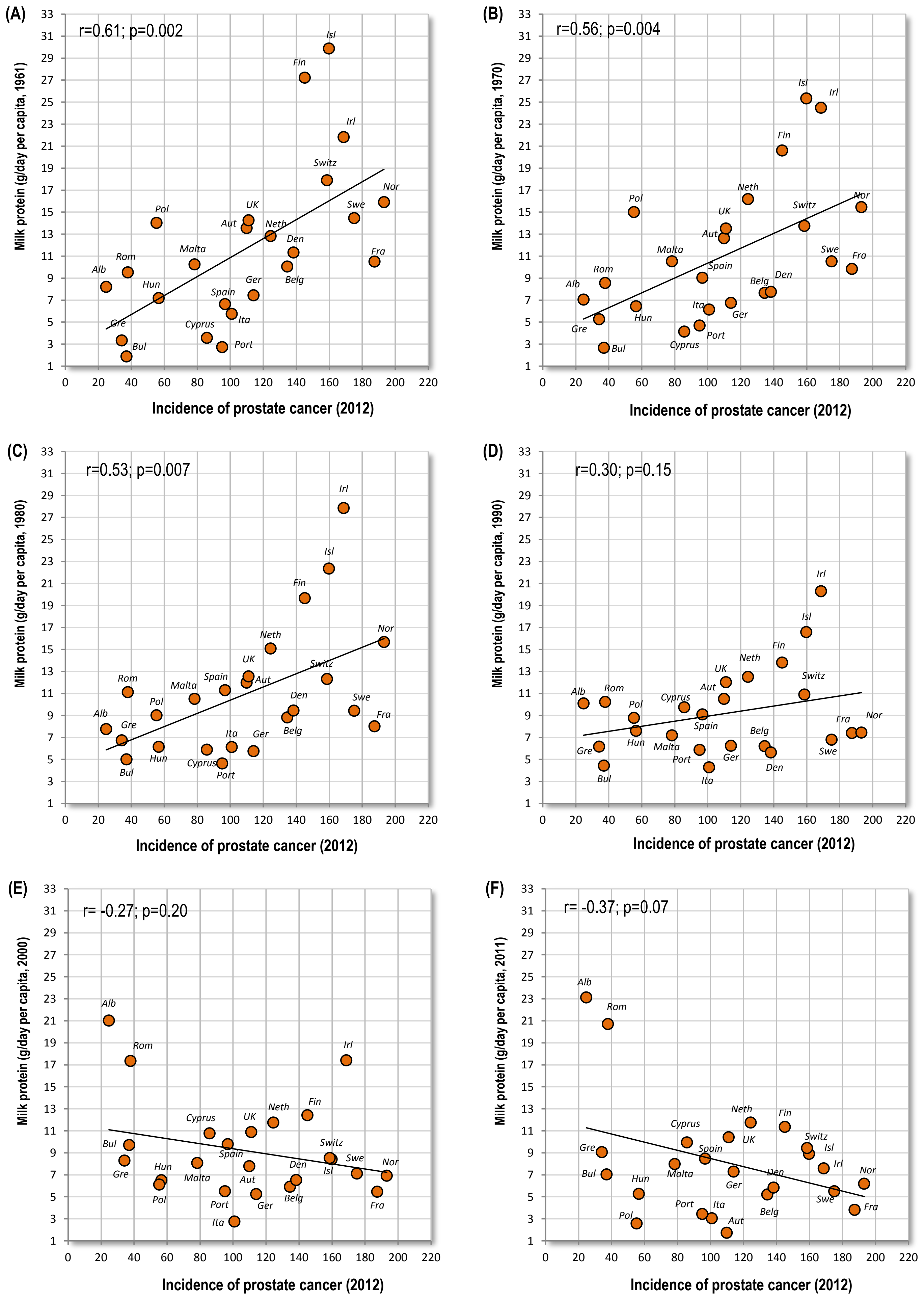

Figures S60A-S60F. Correlations between the historical consumption of milk protein and the current incidence of prostate cancer (a sample of 24 countries).

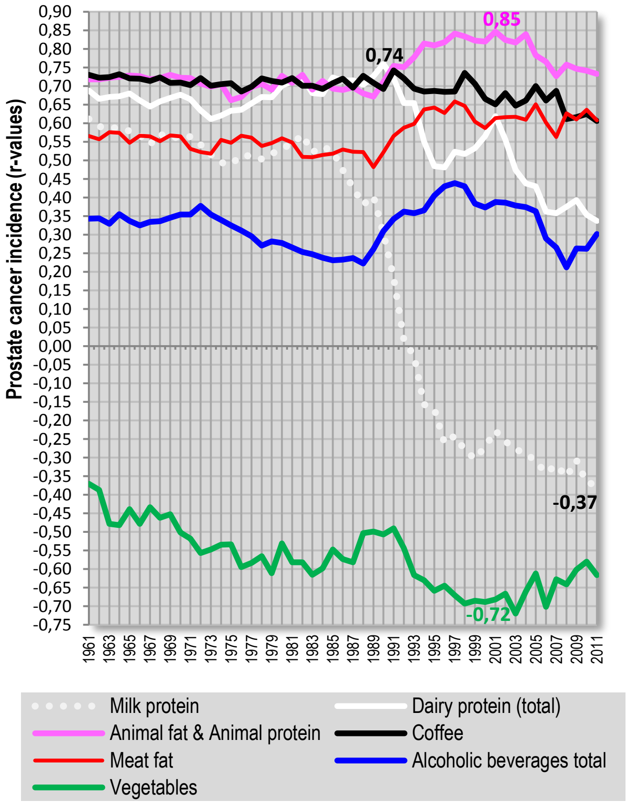

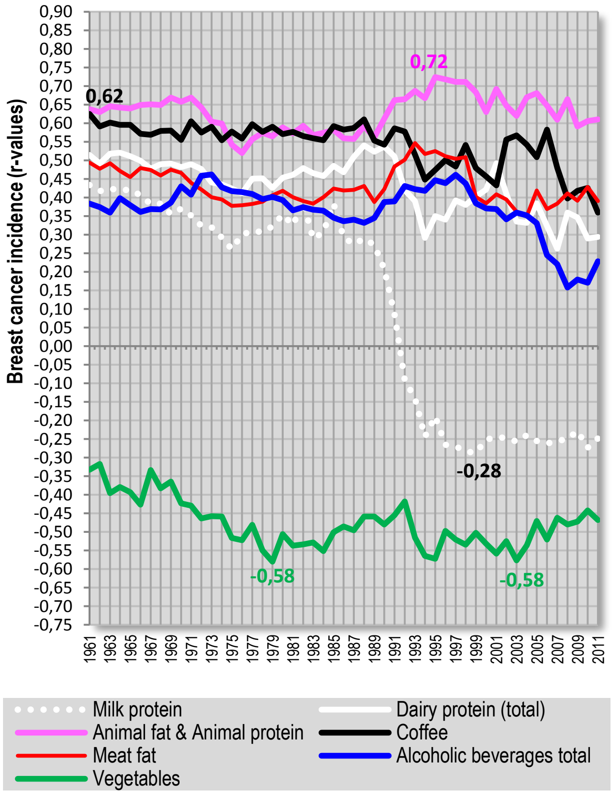

Figure S61. Temporal changes in the relationship between 7 correlates of prostate cancer incidence (a sample of 24 countries).

Figure S62. Temporal changes in the relationship between 7 correlates of breast cancer incidence (a sample of 24 countries).

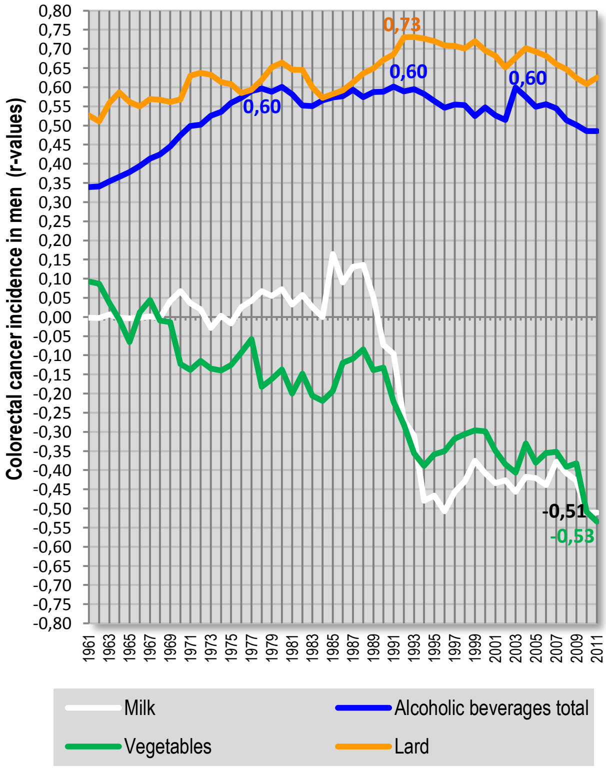

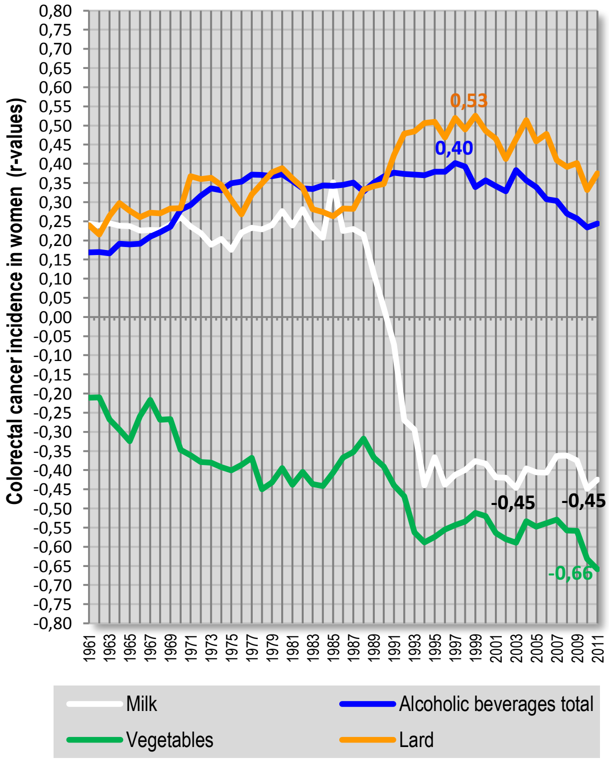

Figure S63. Temporal changes in the relationship between 4 correlates of colorectal cancer incidence in men (a sample of 24 countries).

Figure S64. Temporal changes in the relationship between 4 correlates of colorectal cancer incidence in women (a sample of 24 countries).

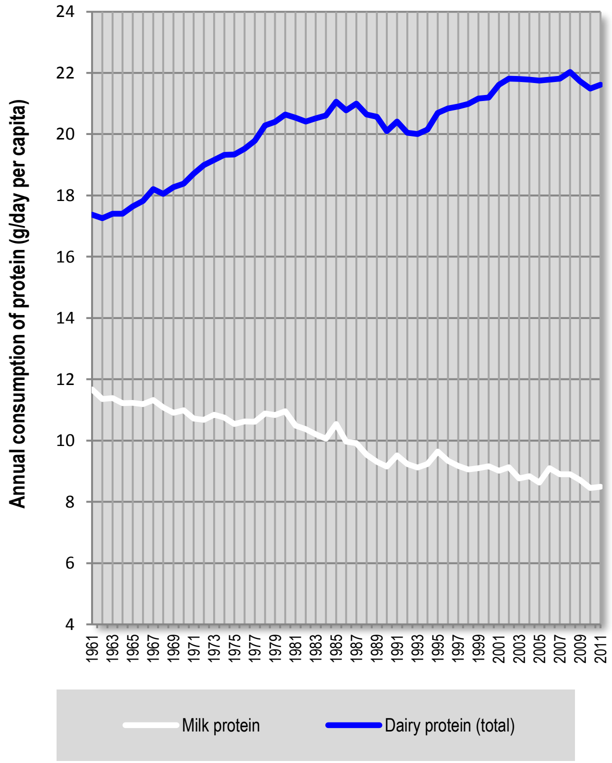

Figure S65. Trends in the consumption of milk protein and total dairy protein in 24 European countries between 1961-2011.

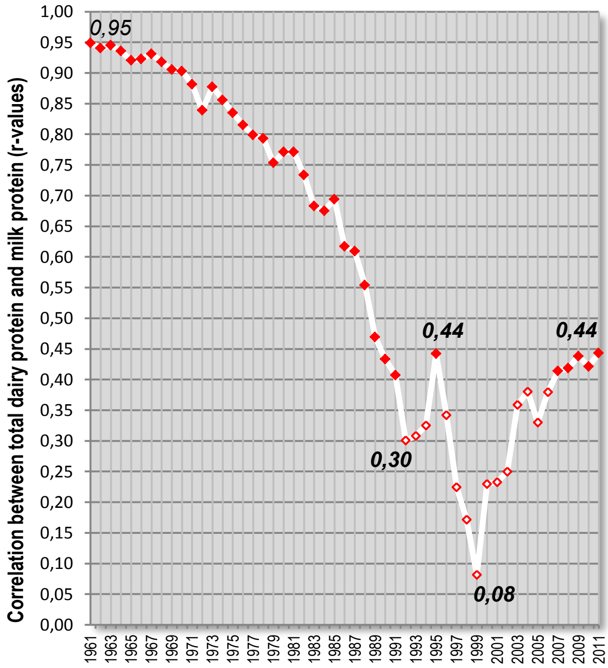

Figure S66. Correlation between the consumption of milk protein and total dairy protein in 24 European countries between 1961-2011. Significant correlations are marked with red points.

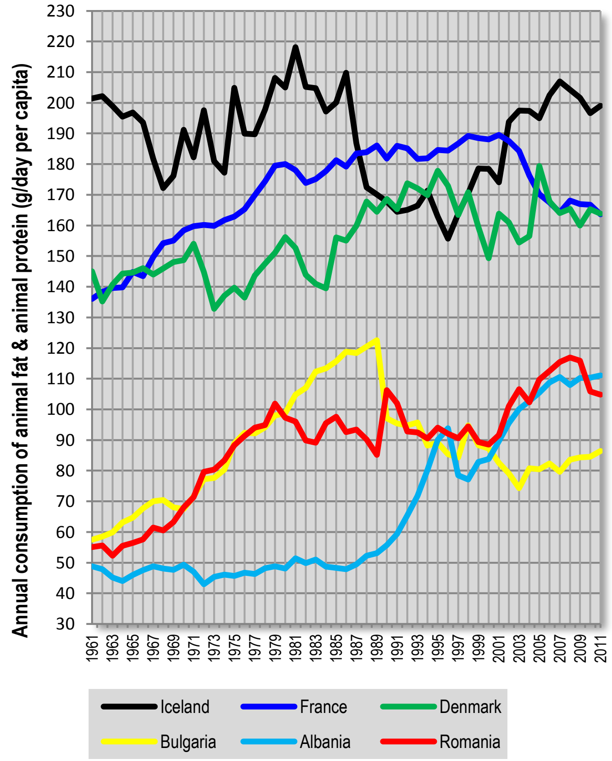

Figure S67. Annual consumption of animal fat & animal protein in six European countries between 1961-2011.

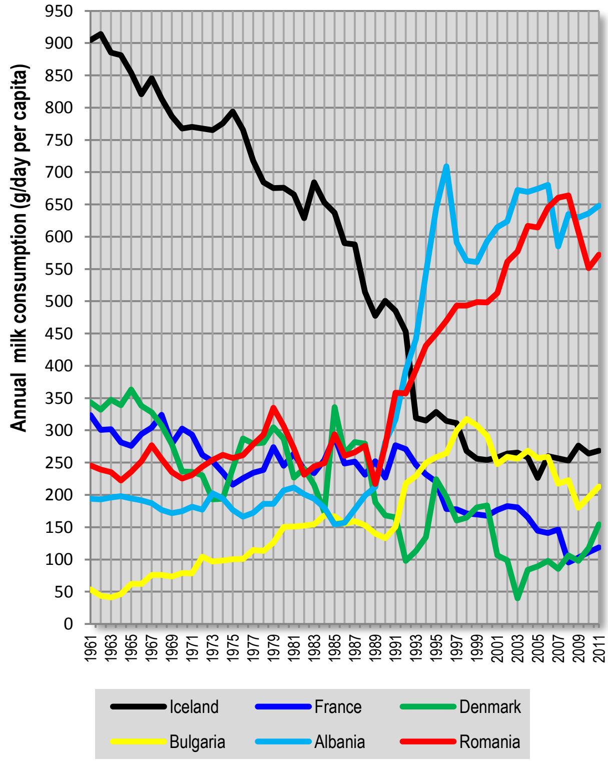

Figure S68. Annual consumption of milk in six European countries between 1961-2011.
