# Supplementary material for: Cancer Incidence in Europe: An Ecological Analysis of Nutritional and Other Environmental Factors
Source: Front Oncol. 2018 Jun 13;8:151. doi: 10.3389/fonc.2018.00151 (PMC6008386; doi:10.3389/fonc.2018.00151)
Supplement: Supplementary file 3 [file data_sheet_3.PDF]

DATA SHEET 3

Supplementary tables

(PENALIZED REGRESSION MODELS)

Notes:

opt = optimal models with the lowest prediction error

pars = parsimonious (economical) models with the best balance between the prediction error and the number of selected predictors

Each regression contains four models: optimal & crossvalidated, selected (parsimonious) & crossvalidated, optimal & bootstrapping, selected (parsimonious) & bootstrapping.

The selected (parsimonious) model in the ridge regression (where all variables remain in the model) is computed differently than in the LASSO regression and elastic net regression: It is based on the „Standardized Sum of Coefficients (SSC)“, and the „acceptable threshold“ computed from the „Expected Prediction Error“ of the optimal model. The parsimonious model has the lowest SSC out of all models below the „acceptable threshold“.

N is the number of countries in the model. Not all countries are included in every model. A country is excluded from the estimation of the expected prediction error provided that the prediction error could not be computed.

Prostate cancer

Ridge cross-validation

Ridge Models

Model	Penalty	Regularization "R Square" (1-Error)	Standardized Sum of Coefficients	Apparent Prediction Error	Expected Prediction Error		
					Estimate	Std. Error	N
24 (opt)	,460	,989	,004	,011	,300	,092	25
49 (pars)	,960	,980	,001	,020	,357	,113	25

Ridge bootstrap

Ridge Models

Model	Penalty	Regularization "R Square" (1-Error)	Standardized Sum of Coefficients	Apparent Prediction Error	Expected Prediction Error		
					Estimate	Std. Error	N
46 (opt)	,900	,981	,001	,019	,172	,071	27
51 (pars)	1,000	,980	,001	,020	,226	,098	28

LASSO cross-validation

LASSO Models

Model	Penalty	Regularization "R Square" (1-Error)	Number of Selected Predictors	Standardized Sum of Coefficients	Apparent Prediction Error	Expected Prediction Error		
						Estimate	Std. Error	N
16 (opt)	,300	,913	9	,018	,087	,377	,128	35
3d (pars)	,680	,764	7	,012	,236	,502	,142	36

LASSO bootstrap

LASSO Models

Model	Penalty	Regularization "R Square" (1-Error)	Number of Selected Predictors	Standardized Sum of Coefficients	Apparent Prediction Error	Expected Prediction Error		
						Estimate	Std. Error	N
34 (opt)	,660	,774	7	,013	,226	,615	,200	39
44 (pars)	,860	,666	5	,010	,334	,791	,272	39

Elastic net cross-validation

Elastic Net Models

Model	Ridge Penalty	LASSO Penalty	Regularization "R Square" (1-Error)	Number of Selected Predictors	Standardized Sum of Coefficients	Apparent Prediction Error	Expected Prediction Error		
							Estimate	Std. Error	N
378 (opt)	0,700	,400	,909	21	,404	,091	,243	,071	31
554 (pars)	1,000	,860	,885	12	,240	,115	,308	,098	34

Elastic net bootstrap

Elastic Net Models

Model	Ridge Penalty	LASSO Penalty	Regularization "R Square" (1-Error)	Number of Selected Predictors	Standardized Sum of Coefficients	Apparent Prediction Error	Expected Prediction Error		
							Estimate	Std. Error	N
215 (opt)	0,400	,200	,927	26	,212	,073	,192	,057	37
539 (pars)	1,000	,560	,907	19	,353	,093	,223	,071	38

Regression models with *beta* coefficients.

Model	24	49	46	51	16	35	34	44	378	554	215	539
**Prostate cancer**	ridge optimal cross	ridge selected cross	ridge optimal bootstrap	ridge selected bootstrap	LASSO optimal cross	LASSO selected cross	LASSO optimal bootstrap	LASSO selected bootstrap	elastic optimal cross	elastic selected cross	elastic optimal bootstrap	elastic selected bootstrap
FRUITS TOTAL	-0,033	-0,038	-0,037	-0,036								
APPLES	-0,070	-0,006	-0,006	-0,006								
BANANAS	0,109	0,039	0,040	0,037	0,040	0,031	0,033	0,007	0,094	0,075	0,068	0,099
GRAPES	0,034	0,038	0,042	0,038								
ORANGES & MANDARINS	-0,049	0,037	0,035	0,034					0,018		0,047	0,007
ALCOHOLIC BEVERAGES TOTAL	0,055	-0,002	-0,005	-0,003					0,008		0,009	
BEER	-0,071	0,019	0,019	0,019								
DISTILLED BEVERAGES	-0,060	-0,023	-0,022	-0,022								
WINE	-0,061	0,019	0,018	0,019								
COCOA BEANS	0,047	0,023	0,019	0,024								
COFFEE	0,016	0,050	0,050	0,050	0,061	0,027	0,029		0,104	0,075	0,108	0,105
TEA	-0,026	-0,010	-0,013	-0,012								
REFINED SUGAR & SWEETENERS TOTAL	0,059	0,013	0,010	0,012								
REFINED SUGAR	-0,035	-0,016	-0,013	-0,015								
OILCROPS TOTAL	-0,029	-0,016	-0,019	-0,017								
OLIVES	-0,013	-0,031	-0,031	-0,031								
TREENUTS	0,035	0,042	0,044	0,040								
PLANT OILS TOTAL	0,007	0,006	0,007	0,006								
OLIVE OIL	-0,015	0,006	0,005	0,005								
SOYBEAN OIL	0,040	0,060	0,056	0,059	0,272	0,225	0,230	0,187	0,197	0,167	0,208	0,192
SUNFLOWER OIL	-0,039	-0,047	-0,043	-0,046	-0,054				-0,067		-0,076	-0,047
CEREALS TOTAL	-0,119	0,003	0,005	0,004					-0,011			-0,013
MAIZE	0,039	-0,020	-0,019	-0,020								
RYE	0,004	0,020	0,023	0,021								
WHEAT	-0,008	-0,039	-0,038	-0,039							-0,027	
POTATOES	-0,041	-0,038	-0,037	-0,037								
LEGUMES TOTAL	-0,033	0,023	0,024	0,022								
VEGETABLES TOTAL	-0,047	-0,046	-0,047	-0,045							-0,055	
ONIONS	-0,079	-0,042	-0,047	-0,042					-0,026		-0,038	-0,012
TOMATOES	-0,028	-0,030	-0,032	-0,030								
SPICES	-0,039	-0,016	-0,015	-0,016								
PLANT PROTEIN	0,012	-0,034	-0,036	-0,033					-0,008		-0,025	
PLANT FAT	-0,025	-0,012	-0,014	-0,011								
MEAT TOTAL	-0,004	0,006	0,008	0,006								
BEEF	-0,009	0,020	0,025	0,021								
PORK	0,090	0,021	0,014	0,019								
POULTRY	0,041	0,031	0,030	0,029								
MEAT PROTEIN	-0,115	-0,009	-0,008	-0,009								
MEAT FAT	0,115	0,030	0,028	0,029					0,038	0,008	0,023	0,041
BEEF & PORK FAT	0,120	0,049	0,050	0,048	0,160	0,059	0,066	0,013	0,125	0,100	0,115	0,123
DAIRY TOTAL	-0,054	0,038	0,034	0,036							0,037	
MILK	-0,012	0,023	0,025	0,021								
CHEESE	0,118	0,034	0,030	0,034					0,038	0,016	0,007	0,046
DAIRY PROTEIN	0,084	0,029	0,026	0,028								
DAIRY FAT	0,099	0,036	0,034	0,035								
MILK PROTEIN	0,040	0,024	0,022	0,020								
MILK FAT	0,034	-0,004	0,022	0,021								
BUTTER & GHEE	0,009	0,043	0,040	0,043							0,005	
EDIBLE OFFALS	0,002	0,019	0,017	0,018								
FISH & SEAFOOD	-0,015	0,044	0,045	0,043							0,028	
FISH & SEAFOOD FAT	-0,022	0,043	0,043	0,042					0,027		0,053	0,014
EGGS TOTAL	-0,019	-0,028	-0,028	-0,027								
LARD	-0,025	0,014	0,018	0,015								
HONEY	0,028	-0,013	-0,013	-0,012								
ANIMAL PROTEIN	0,059	-0,004	0,025	0,035					0,011		0,029	0,008
ANIMAL FAT	0,017	0,042	0,033	0,041					0,082	0,069	0,081	0,088
ANIMAL FAT & ANIMAL PROTEIN	-0,030	0,031	0,038	0,031					0,047	0,042	0,022	0,061
TOTAL PROTEIN	0,089	0,022	0,016	-0,002								
TOTAL FAT	0,057	0,014	0,017	0,013								
TOTAL FAT & TOTAL PROTEIN	0,131	0,027	0,025	0,027								
% CA energy	0,083	-0,015	-0,018	-0,015								
% PC CARB energy	0,068	-0,015	-0,012	-0,015								
% Plant food energy	-0,144	-0,042	-0,041	-0,041	-0,164	-0,172	-0,170	-0,165	-0,150	-0,139	-0,125	-0,152
TOTAL ENERGY	0,004	-0,010	-0,014	-0,009								
VEGETABLES & CEREALS	-0,051	-0,043	-0,037	-0,042	-0,017				-0,083	-0,033	-0,067	-0,078
MILK & VEGETABLES	-0,034	-0,037	-0,040	-0,038								
MILK & VEG. & CEREALS	-0,001	-0,032	-0,035	-0,033					-0,009		-0,001	-0,009
PROTEIN INDEX	0,016	0,037	0,031	0,035							0,008	
Smoking - men (1990-2009)	-0,116	-0,059	-0,058	-0,058	-0,105	-0,007	-0,013		-0,108	-0,034	-0,122	-0,091
BMI – men (1990-2008)	0,068	0,019	0,018	0,019								
Raised cholesterol - men (2008)	-0,008	0,044	0,050	0,043	0,082	0,125	0,121	0,146	0,145	0,154	0,111	0,158

Testicular cancer

Ridge crossvalidation

Ridge Models

Model	Penalty	Regularization "R Square" (1-Error)	Standardized Sum of Coefficients	Apparent Prediction Error	Expected Prediction Error		
					Estimate	Std. Error	N
16 (opt & pars)	,300	,980	,019	,020	,583	,154	25

Ridge bootstrap

Ridge Models

Model	Penalty	Regularization "R Square" (1-Error)	Standardized Sum of Coefficients	Apparent Prediction Error	Expected Prediction Error		
					Estimate	Std. Error	N
41 (opt)	,800	,967	,004	,033	,349	,103	28
48 (pars)	,940	,963	,003	,037	,354	,112	26

Lasso crossvalidation

Lasso Models

Model	Penalty	Regularization "R Square" (1-Error)	Number of Selected Predictors	Standardized Sum of Coefficients	Apparent Prediction Error	Expected Prediction Error		
						Estimate	Std. Error	N
51 (opt & pars)	1,000	,317	5	,007	,683	1,049	,481	37

Lasso bootstrap

Lasso Models

Model	Penalty	Regularization "R Square" (1-Error)	Number of Selected Predictors	Standardized Sum of Coefficients	Apparent Prediction Error	Expected Prediction Error		
						Estimate	Std. Error	N
26 (opt)	,500	,695	10	,021	,305	,799	,268	39
50 (pars)	,980	,326	5	,007	,674	1,055	,388	39

Elastic net crossvalidation

Elastic Net Models

Model	Ridge Penalty	Lasso Penalty	Regularization "R Square" (1-Error)	Number of Selected Predictors	Standardized Sum of Coefficients	Apparent Prediction Error	Expected Prediction Error		
							Estimate	Std. Error	N
154 (opt & pars)	,300	,000	,930	71	1,000	,070	,731	,188	25

Elastic net bootstrap

Elastic Net Models

Model	Ridge Penalty	Lasso Penalty	Regularization "R Square" (1-Error)	Number of Selected Predictors	Standardized Sum of Coefficients	Apparent Prediction Error	Expected Prediction Error		
							Estimate	Std. Error	N
259 (opt)	,500	,060	,887	54	,708	,113	,446	,115	35
369 (pars)	,700	,220	,839	34	,451	,161	,523	,153	37

Regression models with *beta* coefficients.

Model	16	16	41	48	51	51	26	50	154	154	259	369
**Testes**	ridge optimal cross	ridge selected cross	ridge optimal bootstrap	ridge selected bootstrap	LASSO optimal cross	LASSO selected cross	LASSO optimal bootstrap	LASSO selected bootstrap	elastic optimal cross	elastic selected cross	elastic optimal bootstrap	elastic selected bootstrap
FRUITS TOTAL	-0,025	-0,025	-0,018	-0,016					-0,032	-0,032	0,016	
APPLES	0,027	0,027	-0,027	-0,023					0,035	0,035	-0,009	
BANANAS	-0,173	-0,173	0,046	0,041					-0,225	-0,225	0,071	0,068
GRAPES	0,028	0,028	0,065	0,070					0,036	0,036	0,115	0,062
ORANGES & MANDARINS	0,089	0,089	0,038	0,034			0,064		0,115	0,115	0,057	0,073
ALCOHOLIC BEVERAGES TOTAL	0,028	0,028	0,044	0,042			0,017		0,036	0,036	0,045	0,070
BEER	0,083	0,083	0,034	0,031					0,108	0,108	0,051	0,027
DISTILLED BEVERAGES	-0,088	-0,088	-0,081	-0,078					-0,115	-0,115	-0,118	-0,083
WINE	0,112	0,112	0,039	0,038			0,034		0,146	0,146	0,038	0,049
COCOA BEANS	-0,034	-0,034	-0,033	-0,020					-0,044	-0,044	0,031	
COFFEE	0,171	0,171	0,039	0,041					0,222	0,222	0,036	0,020
TEA	0,088	0,088	0,032	0,029					0,114	0,114	0,029	
REFINED SUGAR & SWEETENERS TOTAL	-0,001	-0,001	0,042	0,046					-0,001	-0,001	0,067	0,062
REFINED SUGAR	-0,066	-0,066	-0,020	-0,021					-0,085	-0,085		
OILCROPS TOTAL	-0,048	-0,048	-0,028	0,063					-0,063	-0,063	0,113	0,055
OLIVES	-0,017	-0,017	0,008	0,006					-0,022	-0,022		
TREENUTS	0,009	0,009	-0,042	-0,040					0,011	0,011	-0,055	
PLANT OILS TOTAL	-0,025	-0,025	-0,038	-0,034					-0,032	-0,032		
OLIVE OIL	0,069	0,069	0,030	-0,032					0,090	0,090	-0,079	-0,001
SOYBEAN OIL	0,166	0,166	0,056	0,058	0,013	0,013		0,003	0,216	0,216	0,080	0,086
SUNFLOWER OIL	-0,063	-0,063	-0,034	-0,032					-0,082	-0,082		
CEREALS TOTAL	0,109	0,109	-0,042	-0,038					0,141	0,141		
MAIZE	-0,093	-0,093	0,035	0,033					-0,121	-0,121	0,021	
RYE	-0,061	-0,061	-0,055	-0,050			-0,035		-0,080	-0,080	-0,080	-0,075
WHEAT	0,046	0,046	0,071	0,066					0,059	0,059	0,097	0,020
POTATOES	-0,118	-0,118	-0,073	-0,074					-0,153	-0,153	-0,123	-0,085
LEGUMES TOTAL	0,002	0,002	-0,053	-0,050					0,003	0,003	-0,070	-0,026
VEGETABLES TOTAL	-0,051	-0,051	-0,022	-0,023					-0,066	-0,066	-0,049	
ONIONS	0,102	0,102	-0,068	-0,064	-0,012	-0,012	-0,156	-0,023	0,132	0,132	-0,148	-0,204
TOMATOES	0,050	0,050	0,045	0,048					0,065	0,065	0,057	
SPICES	0,056	0,056	0,034	0,029					0,072	0,072	0,020	0,006
PLANT PROTEIN	-0,080	-0,080	-0,037	-0,038					-0,104	-0,104	-0,055	-0,008
PLANT FAT	-0,027	-0,027	-0,036	-0,032					-0,035	-0,035	0,019	
MEAT TOTAL	-0,125	-0,125	-0,022	-0,024					-0,163	-0,163	-0,030	
BEEF	0,014	0,014	-0,026	-0,029					0,018	0,018	0,018	0,005
PORK	-0,071	-0,071	-0,032	-0,027					-0,093	-0,093	-0,052	
POULTRY	0,035	0,035	-0,007	-0,009					0,046	0,046		
MEAT PROTEIN	0,059	0,059	-0,022	-0,021					0,076	0,076	-0,016	
MEAT FAT	-0,153	-0,153	0,029	0,030					-0,199	-0,199	0,014	
BEEF & PORK FAT	0,024	0,024	0,028	0,027					0,031	0,031	0,020	
DAIRY TOTAL	0,143	0,143	0,017	0,018					0,186	0,186	0,003	
MILK	-0,125	-0,125	-0,058	-0,054					-0,162	-0,162	-0,078	-0,050
CHEESE	-0,020	-0,020	-0,017	-0,018	0,133	0,133	0,163	0,167	-0,026	-0,026	0,105	0,111
DAIRY PROTEIN	-0,093	-0,093	0,017	0,017					-0,121	-0,121		
DAIRY FAT	0,020	0,020	0,013	0,015					0,026	0,026		
MILK PROTEIN	-0,054	-0,054	-0,029	-0,032					-0,071	-0,071	-0,034	-0,017
MILK FAT	-0,028	-0,028	-0,019	-0,024					-0,036	-0,036	0,011	
BUTTER & GHEE	0,138	0,138	0,012	0,014					0,180	0,180		
EDIBLE OFFALS	-0,015	-0,015	-0,027	-0,020					-0,020	-0,020	-0,010	0,038
FISH & SEAFOOD	0,021	0,021	-0,044	0,010					0,027	0,027	-0,051	
FISH & SEAFOOD FAT	-0,042	-0,042	-0,029	-0,034					-0,054	-0,054		
EGGS TOTAL	0,050	0,050	0,056	0,047					0,065	0,065	0,072	0,054
LARD	0,134	0,134	0,084	0,081			0,148		0,174	0,174	0,156	0,172
HONEY	0,036	0,036	-0,036	-0,035					0,047	0,047	-0,028	
ANIMAL PROTEIN	0,139	0,139	-0,016	-0,010					0,181	0,181		
ANIMAL FAT	0,249	0,249	0,047	0,049	0,060	0,060		0,023	0,324	0,324	0,077	0,086
ANIMAL FAT & ANIMAL PROTEIN	0,002	0,002	0,039	0,033					0,002	0,002	0,053	0,023
TOTAL PROTEIN	-0,101	-0,101	-0,032	-0,027					-0,131	-0,131	-0,027	
TOTAL FAT	-0,113	-0,113	0,037	0,033					-0,147	-0,147	0,010	
TOTAL FAT & TOTAL PROTEIN	-0,186	-0,186	0,014	-0,001					-0,242	-0,242		
% CA energy	-0,081	-0,081	-0,047	-0,042					-0,105	-0,105	-0,059	-0,039
% PC CARB energy	-0,194	-0,194	-0,035	-0,038					-0,252	-0,252		
% Plant food energy	0,159	0,159	-0,049	-0,046			-0,040		0,207	0,207	-0,086	-0,089
TOTAL ENERGY	-0,015	-0,015	-0,018	-0,014					-0,019	-0,019		
VEGETABLES & CEREALS	-0,186	-0,186	-0,025	-0,029					-0,242	-0,242		
MILK & VEGETABLES	-0,061	-0,061	-0,040	-0,039					-0,079	-0,079	-0,074	-0,076
MILK & VEG. & CEREALS	-0,071	-0,071	-0,047	-0,042	-0,048	-0,048	-0,116	-0,058	-0,092	-0,092	-0,100	-0,122
PROTEIN INDEX	-0,008	-0,008	-0,033	-0,028					-0,011	-0,011		
Smoking - men (1990-2009)	0,032	0,032	-0,035	-0,036			-0,014		0,042	0,042		-0,054
BMI – men (1990-2008)	-0,033	-0,033	0,056	0,052					-0,043	-0,043	0,063	
Raised cholesterol - men (2008)	-0,035	-0,035	0,046	0,040					-0,046	-0,046	0,044	0,009

Breast cancer

Ridge cross-validation

Ridge Models

Model	Penalty	Regularization "R Square" (1-Error)	Standardized Sum of Coefficients	Apparent Prediction Error	Expected Prediction Error Estimate	Std. Error	N
33 (opt)	,640	,983	,002	,017	,522	,191	26
51 (pars)	1,000	,970	,002	,030	,622	,220	26

Ridge bootstrap

Ridge Models

Model	Penalty	Regularization "R Square" (1-Error)	Standardized Sum of Coefficients	Apparent Prediction Error	Expected Prediction Error Estimate	Std. Error	N
42 (opt)	,820	,976	,002	,024	,229	,085	28
51 (pars)	1,000	,970	,002	,030	,297	,104	31

LASSO cross-validation

LASSO Models

Model	Penalty	Regularization "R Square" (1-Error)	Number of Selected Predictors	Standardized Sum of Coefficients	Apparent Prediction Error	Expected Prediction Error Estimate	Std. Error	N
31 (opt)	,600	,661	6	,013	,339	,660	,174	35
50 (pars)	,980	,420	4	,007	,580	,785	,159	36

LASSO bootstrap

LASSO Models

Model	Penalty	Regularization "R Square" (1-Error)	Number of Selected Predictors	Standardized Sum of Coefficients	Apparent Prediction Error	Expected Prediction Error Estimate	Std. Error	N
23 (opt)	,440	,764	9	,017	,236	,564	,156	39
44 (pars)	,860	,495	5	,008	,505	,716	,138	39

Elastic net cross-validation

Elastic Net Models

Model	Ridge Penalty	LASSO Penalty	Regularization "R Square" (1-Error)	Number of Selected Predictors	Standardized Sum of Coefficients	Apparent Prediction Error	Expected Prediction Error		
							Estimate	Std. Error	N
115 (opt)	0.2	,240	,916	22	,182	,084	,608	,243	32
459 (pars)	0.8	1,000	,546	4	,103	,454	,811	,169	35

Elastic net bootstrap

Elastic Net Models

Model	Ridge Penalty	LASSO Penalty	Regularization "R Square" (1-Error)	Number of Selected Predictors	Standardized Sum of Coefficients	Apparent Prediction Error	Expected Prediction Error		
							Estimate	Std. Error	N
260 (opt)	0.5	,080	,883	46	,402	,117	,394	,115	34
542 (pars)	1,0	,620	,792	17	,241	,208	,497	,172	39

Regression models with *beta* coefficients.

Model	33	51	42	51	31	50	23	44	115	459	260	542
**Breast cancer**	ridge optimal cross	ridge selected cross	ridge optimal bootstrap	ridge selected bootstrap	LASSO optimal cross	LASSO selected cross	LASSO optimal bootstrap	LASSO selected bootstrap	elastic optimal cross	elastic selected cross	elastic optimal bootstrap	elastic selected bootstrap
FRUITS TOTAL	-0,030	-0,030	-0,034	-0,030					0,019		0,060	0,067
APPLES	-0,027	-0,032	-0,030	-0,032							-0,016	
BANANAS	0,051	0,047	0,046	0,047					0,023		0,067	
GRAPES	0,074	0,063	0,069	0,063			0,044		0,118		0,127	0,007
ORANGES & MANDARINS	0,027	0,022	0,025	0,022								
ALCOHOLIC BEVERAGES TOTAL	0,022	0,026	0,021	0,026								
BEER	0,032	0,025	0,031	0,025							0,022	
DISTILLED BEVERAGES	-0,075	-0,079	-0,076	-0,079	-0,062		-0,090		-0,116		-0,141	-0,057
WINE	-0,004	0,006	0,003	0,006								
COCOA BEANS	-0,026	0,039	0,031	0,039							0,033	
COFFEE	0,066	0,064	0,061	0,064	0,149	0,069	0,169	0,086	0,104		0,107	0,144
TEA	0,041	0,036	0,039	0,036							0,027	
REFINED SUGAR & SWEETENERS TOTAL	-0,023	-0,010	-0,021	-0,010								
REFINED SUGAR	0,036	0,031	0,037	0,031							0,010	
OILCROPS TOTAL	0,071	0,065	0,071	0,065					0,041		0,116	
OLIVES	-0,046	-0,038	-0,043	-0,038								
TREENUTS	-0,031	-0,031	-0,031	-0,031							-0,022	
PLANT OILS TOTAL	0,014	0,021	0,015	0,021								
OLIVE OIL	-0,027	-0,025	-0,028	-0,025							-0,016	
SOYBEAN OIL	0,065	0,064	0,060	0,064	0,078	0,037	0,059	0,066	0,059		0,081	0,102
SUNFLOWER OIL	-0,022	-0,026	-0,021	-0,026								
CEREALS TOTAL	-0,035	-0,037	-0,033	-0,037							-0,041	-0,007
MAIZE	0,026	0,024	0,024	0,024								
RYE	-0,046	-0,044	-0,044	-0,044							-0,047	
WHEAT	0,028	0,025	0,028	0,025								
POTATOES	-0,069	-0,067	-0,066	-0,067					-0,056		-0,116	
LEGUMES TOTAL	-0,025	-0,015	-0,017	-0,015								
VEGETABLES TOTAL	-0,025	-0,023	-0,025	-0,023							-0,020	
ONIONS	-0,043	-0,042	-0,043	-0,042							-0,070	
TOMATOES	0,041	0,036	0,036	0,036							0,054	
SPICES	-0,025	-0,018	-0,023	-0,018								
PLANT PROTEIN	-0,034	-0,025	-0,031	-0,025							-0,044	
PLANT FAT	0,056	0,058	0,054	0,058					0,059		0,092	0,003
MEAT TOTAL	0,032	0,019	0,027	0,019							0,008	
BEEF	0,029	0,014	0,024	0,014								
PORK	-0,014	-0,011	-0,015	-0,011							0,017	
POULTRY	0,018	0,015	0,016	0,015								
MEAT PROTEIN	0,035	0,019	0,032	0,019							0,021	
MEAT FAT	0,028	0,031	0,026	0,031							0,032	
BEEF & PORK FAT	0,034	0,033	0,030	0,033							0,033	
DAIRY TOTAL	0,027	0,026	0,028	0,026								
MILK	-0,033	-0,037	-0,033	-0,037							-0,022	
CHEESE	-0,041	-0,033	-0,038	-0,033							-0,042	
DAIRY PROTEIN	-0,025	-0,022	-0,027	-0,022								
DAIRY FAT	-0,039	-0,034	-0,036	-0,034							-0,030	
MILK PROTEIN	-0,043	-0,039	-0,041	-0,039					-0,004		-0,046	
MILK FAT	-0,045	-0,044	-0,045	-0,044					-0,001		-0,050	
BUTTER & GHEE	0,021	0,026	0,023	0,026								
EDIBLE OFFALS	0,022	-0,008	-0,008	-0,008							0,006	
FISH & SEAFOOD	-0,026	-0,029	-0,025	-0,029								
FISH & SEAFOOD FAT	-0,016	-0,019	-0,014	-0,019							0,011	
EGGS TOTAL	-0,020	-0,019	-0,020	-0,019								
LARD	-0,010	-0,009	-0,008	-0,009			0,050		0,079		0,078	
HONEY	-0,052	-0,048	-0,049	-0,048					-0,030		-0,085	
ANIMAL PROTEIN	0,015	0,025	0,014	0,025								
ANIMAL FAT	0,041	0,051	0,039	0,051					0,007	0,051	0,053	0,092
ANIMAL FAT & ANIMAL PROTEIN	0,037	0,022	0,034	0,022						0,041	0,034	
TOTAL PROTEIN	-0,015	-0,009	-0,016	-0,009								
TOTAL FAT	0,042	0,038	0,039	0,038							0,047	0,049
TOTAL FAT & TOTAL PROTEIN	-0,006	0,016	0,019	0,016								
% CA energy	-0,068	-0,046	-0,061	-0,046	-0,133	-0,079	-0,173	-0,100	-0,194		-0,113	-0,140
% PC CARB energy	-0,045	-0,042	-0,040	-0,042	-0,140	-0,149	-0,092	-0,152	-0,102		-0,061	-0,143
% Plant food energy	-0,031	-0,034	-0,030	-0,034					-0,010	-0,071		-0,036
TOTAL ENERGY	-0,022	-0,013	-0,020	-0,013								
VEGETABLES & CEREALS	-0,040	-0,048	-0,038	-0,048					-0,023		-0,076	-0,046
MILK & VEGETABLES	-0,048	-0,043	-0,048	-0,043					-0,134		-0,101	-0,006
MILK & VEG. & CEREALS	-0,046	-0,036	-0,044	-0,036	-0,083		-0,148	-0,012	-0,139		-0,090	-0,089
PROTEIN INDEX	-0,021	0,010	0,006	0,010								
Smoking - women (1990 - 2009)	0,075	0,063	0,070	0,063			0,021		0,088		0,116	0,045
BMI - women (1990-2008)	-0,030	-0,024	-0,025	-0,024								
Raised cholesterol - women (2008)	0,044	0,037	0,040	0,037					0,009	0,284	0,046	0,070

Non-Hodgkin lymphoma - men

Ridge cross-validation

Ridge Models

Model	Penalty	Regularization "R Square" (1-Error)	Standardized Sum of Coefficients	Apparent Prediction Error	Expected Prediction Error		
					Estimate	Std. Error	N
31 (opt)	,600	,991	,001	,009	,463	,230	25
51 (pars)	1,000	,980	,001	,020	,607	,234	25

Ridge bootstrap

Ridge Models

Model	Penalty	Regularization "R Square" (1-Error)	Standardized Sum of Coefficients	Apparent Prediction Error	Expected Prediction Error		
					Estimate	Std. Error	N
36 (opt)	,700	,962	,007	,038	,194	,084	27
4 (pars)	,880	,973	,002	,027	,275	,137	28

LASSO cross-validation

LASSO Models

Model	Penalty	Regularization "R Square" (1-Error)	Number of Selected Predictors	Standardized Sum of Coefficients	Apparent Prediction Error	Expected Prediction Error		
						Estimate	Std. Error	N
29 (opt)	,560	,802	4	,013	,198	1,163	,745	38
51 (pars)	1,000	,552	3	,008	,448	1,505	,925	38

LASSO bootstrap

LASSO Models

Model	Penalty	Regularization "R Square" (1-Error)	Number of Selected Predictors	Standardized Sum of Coefficients	Apparent Prediction Error	Expected Prediction Error		
						Estimate	Std. Error	N
23 (opt)	,440	,863	5	,016	,137	,590	,363	39
50 (pars)	,980	,566	3	,008	,434	,894	,568	39

Elastic net cross-validation

Elastic Net Models

Model	Ridge Penalty	LASSO Penalty	Regularization "R Square" (1-Error)	Number of Selected Predictors	Standardized Sum of Coefficients	Apparent Prediction Error	Expected Prediction Error		
							Estimate	Std. Error	N
307 (opt)	,600	,000	,737	71	1,000	,263	,586	,251	25
561 (pars)	1,000	1,000	,743	10	,168	,257	,763	,220	34

Elastic net bootstrap

Elastic Net Models

Model	Ridge Penalty	LASSO Penalty	Regularization "R Square" (1-Error)	Number of Selected Predictors	Standardized Sum of Coefficients	Apparent Prediction Error	Expected Prediction Error		
							Estimate	Std. Error	N
276 (opt)	0,5	,400	,923	18	,243	,077	,326	,150	38
550 (pars)	1,0	,780	,872	16	,246	,128	,423	,191	39

Regression models with *beta* coefficients.

Model	31	51	36	45	29	51	23	50	307	561	276	550
**Non-Hodgkin lymphoma - men**	ridge optimal cross	ridge selected cross	ridge optimal bootstrap	ridge selected bootstrap	LASSO optimal cross	LASSO selected cross	LASSO optimal bootstrap	LASSO selected bootstrap	elastic optimal cross	elastic selected cross	elastic optimal bootstrap	elastic selected bootstrap
FRUITS TOTAL	-0,053	-0,045	-0,049	-0,049					-0,086			
APPLES	-0,025	-0,025	0,001	-0,033					-0,040			
BANANAS	0,046	0,053	-0,014	0,098					0,073	0,092	0,121	0,106
GRAPES	-0,049	-0,042	-0,060	-0,034					-0,079			
ORANGES & MANDARINS	0,048	0,042	0,028	-0,006					0,077	0,033	0,091	0,067
ALCOHOLIC BEVERAGES TOTAL	0,039	0,039	0,142	0,018					0,062		0,065	0,016
BEER	0,035	0,030	-0,039	0,033					0,056			
DISTILLED BEVERAGES	-0,026	-0,029	-0,059	-0,041					-0,042			
WINE	0,068	0,056	0,045	0,056					0,109		0,056	0,017
COCOA BEANS	0,003	0,007	0,085	-0,042					0,004			
COFFEE	0,028	0,022	0,046	-0,015					0,045	0,013	0,022	0,037
TEA	0,016	0,013	0,032	-0,012					0,026			
REFINED SUGAR & SWEETENERS TOTAL	0,015	0,016	0,096	0,013					0,024			
REFINED SUGAR	0,036	0,030	-0,083	0,045					0,057			
OILCROPS TOTAL	-0,051	-0,044	0,018	0,004					-0,082			
OLIVES	-0,072	-0,063	-0,042	-0,071	-0,268	-0,114	-0,361	-0,120	-0,116			
TREENUTS	0,059	0,055	0,036	0,079					0,094			
PLANT OILS TOTAL	-0,010	-0,009	0,019	-0,025					-0,016			
OLIVE OIL	0,017	0,013	0,003	-0,019					0,028			
SOYBEAN OIL	0,045	0,040	0,093	0,020					0,073	0,045	0,089	0,066
SUNFLOWER OIL	0,011	0,005	-0,055	-0,004					0,017			
CEREALS TOTAL	0,022	-0,022	-0,129	-0,019					0,035			
MAIZE	0,016	0,015	0,052	0,027					0,025			
RYE	-0,035	-0,033	-0,053	-0,054					-0,056			
WHEAT	0,005	-0,004	-0,012	0,025					0,008			
POTATOES	-0,017	-0,018	-0,031	-0,017					-0,028			
LEGUMES TOTAL	-0,030	-0,030	-0,029	-0,027					-0,048			
VEGETABLES TOTAL	-0,064	-0,056	-0,041	-0,068					-0,102		-0,013	
ONIONS	-0,046	-0,049	0,030	-0,097					-0,073		-0,081	-0,030
TOMATOES	-0,023	-0,023	-0,016	-0,026					-0,037			
SPICES	-0,033	-0,028	-0,030	-0,034					-0,053			
PLANT PROTEIN	-0,023	-0,017	0,014	0,023					-0,038			
PLANT FAT	-0,008	-0,007	0,066	-0,035					-0,013			
MEAT TOTAL	0,022	0,024	-0,076	0,071					0,036			
BEEF	0,016	0,014	-0,105	0,057					0,025			
PORK	-0,035	-0,030	0,170	0,003	0,202	0,097	0,171	0,103	-0,056			
POULTRY	0,035	0,033	0,027	0,063	0,039		0,134		0,056			
MEAT PROTEIN	0,018	0,011	-0,127	-0,008					0,028			
MEAT FAT	0,030	0,033	0,057	0,046					0,048			
BEEF & PORK FAT	0,027	0,032	-0,003	0,055					0,043		0,012	0,005
DAIRY TOTAL	0,029	0,022	0,007	-0,024					0,047			
MILK	-0,005	-0,007	-0,028	-0,002					-0,007			
CHEESE	-0,041	-0,034	-0,023	-0,033					-0,065			
DAIRY PROTEIN	-0,009	0,022	0,078	0,025					-0,014			
DAIRY FAT	-0,044	-0,040	-0,070	-0,031					-0,071			
MILK PROTEIN	-0,015	-0,016	-0,007	-0,021					-0,025			
MILK FAT	0,030	0,028	0,033	0,033					0,047			
BUTTER & GHEE	0,045	0,036	0,081	-0,015					0,072		0,017	
EDIBLE OFFALS	0,013	0,012	0,030	-0,014					0,020			
FISH & SEAFOOD	0,037	0,033	-0,019	0,044					0,060			
FISH & SEAFOOD FAT	0,050	0,043	0,018	0,035					0,081		0,087	0,028
EGGS TOTAL	0,040	0,036	-0,028	0,065					0,064			
LARD	0,030	0,027	0,002	-0,008					0,048			
HONEY	-0,024	-0,024	-0,048	-0,024					-0,038			
ANIMAL PROTEIN	0,018	0,020	0,187	-0,039					0,028	0,002	0,025	0,031
ANIMAL FAT	0,024	0,018	0,159	-0,043					0,038	0,031	0,026	0,049
ANIMAL FAT & ANIMAL PROTEIN	0,026	0,025	-0,170	0,086					0,042	0,069	0,061	0,086
TOTAL PROTEIN	0,031	0,034	0,106	0,030					0,049			
TOTAL FAT	0,017	0,022	-0,042	0,070					0,026			
TOTAL FAT & TOTAL PROTEIN	0,015	0,021	0,105	0,020					0,024			
% CA energy	-0,024	-0,018	0,154	-0,060					-0,039			
% PC CARB energy	-0,020	-0,010	-0,021	0,034					-0,032			
% Plant food energy	-0,037	-0,044	-0,096	-0,061					-0,059	-0,158	-0,176	-0,168
TOTAL ENERGY	-0,015	-0,013	0,119	-0,040					-0,024			
VEGETABLES & CEREALS	-0,036	-0,029	-0,116	0,030			-0,010		-0,058		-0,042	-0,015
MILK & VEGETABLES	-0,039	-0,031	-0,041	-0,023					-0,062			
MILK & VEG. & CEREALS	-0,044	-0,035	-0,012	-0,033	-0,233	-0,208	-0,190	-0,209	-0,071			
PROTEIN INDEX	0,018	0,012	0,063	0,011					0,029			
Smoking - men (1990-2009)	-0,080	-0,077	-0,184	-0,062					-0,128	-0,155	-0,224	-0,181
BMI – men (1990-2008)	-0,036	-0,024	-0,004	-0,019					-0,057			
Raised cholesterol - men (2008)	0,031	0,033	-0,114	0,100					0,049	0,072	0,057	0,082

Non-Hodgkin lymphoma - women

Ridge cross-validation

Ridge Models

Model	Penalty	Regularization "R Square" (1-Error)	Standardized Sum of Coefficients	Apparent Prediction Error	Expected Prediction Error		
					Estimate	Std. Error	N
34 (opt)	,660	,987	,001	,013	,434	,125	26
4 (pars)	,880	,980	,001	,020	,556	,196	26

Ridge bootstrap

Ridge Models

Model	Penalty	Regularization "R Square" (1-Error)	Standardized Sum of Coefficients	Apparent Prediction Error	Expected Prediction Error		
					Estimate	Std. Error	N
46 (opt)	,900	,979	,001	,021	,217	,093	28
51 (pars)	1,000	,976	,001	,024	,244	,100	28

LASSO cross-validation

LASSO Models

Model	Penalty	Regularization "R Square" (1-Error)	Number of Selected Predictors	Standardized Sum of Coefficients	Apparent Prediction Error	Expected Prediction Error		
						Estimate	Std. Error	N
7 (opt)	,120	,977	11	,027	,023	,805	,294	34
11 (pars)	,200	,950	10	,024	,050	1,064	,443	36

LASSO bootstrap

LASSO Models

Model	Penalty	Regularization "R Square" (1-Error)	Number of Selected Predictors	Standardized Sum of Coefficients	Apparent Prediction Error	Expected Prediction Error		
						Estimate	Std. Error	N
40 (opt)	,780	,615	4	,009	,385	,770	,349	39
51 (pars)	1,000	,482	2	,007	,518	,972	,432	39

Elastic net cross-validation

Elastic Net Models

Model	Ridge Penalty	LASSO Penalty	Regularization "R Square" (1-Error)	Number of Selected Predictors	Standardized Sum of Coefficients	Apparent Prediction Error	Expected Prediction Error		
							Estimate	Std. Error	N
280 (opt & pars)	0,5	,480	,863	16	,309	,137	,490	,153	34

Elastic net bootstrap

Elastic Net Models

Model	Ridge Penalty	LASSO Penalty	Regularization "R Square" (1-Error)	Number of Selected Predictors	Standardized Sum of Coefficients	Apparent Prediction Error	Expected Prediction Error		
							Estimate	Std. Error	N
207 (opt)	0,4	,040	,912	58	,805	,088	,371	,118	35
553 (pars)	1,0	,840	,778	13	,194	,222	,455	,148	38

Regression models with *beta* coefficients.

Model	34	45	46	51	7	11	40	51	280	280	207	553
**Non-Hodgkin lymphoma - women**	ridge optimal cross	ridge selected cross	ridge optimal bootstrap	ridge selected bootstrap	LASSO optimal cross	LASSO selected cross	LASSO optimal bootstrap	LASSO selected bootstrap	elastic optimal cross	elastic selected cross	elastic optimal bootstrap	elastic selected bootstrap
FRUITS TOTAL	-0,041	-0,040	-0,038	-0,039							-0,062	
APPLES	-0,036	-0,029	-0,033	-0,028							-0,040	
BANANAS	0,064	0,053	0,060	0,050			0,034		0,134	0,134	0,098	0,120
GRAPES	-0,056	-0,057	-0,053	-0,057	-0,075	-0,086						
ORANGES & MANDARINS	0,016	0,028	0,018	0,027							0,005	
ALCOHOLIC BEVERAGES TOTAL	0,050	0,033	0,048	0,034							0,043	
BEER	0,026	0,033	0,024	0,033							0,028	
DISTILLED BEVERAGES	-0,076	-0,067	-0,070	-0,063	-0,153	-0,106			-0,022	-0,022	-0,116	
WINE	0,057	0,062	0,055	0,061	0,037	0,001			0,076	0,076	0,122	0,015
COCOA BEANS	-0,027	-0,031	-0,023	-0,028							-0,038	
COFFEE	0,028	0,027	0,026	0,025							0,002	
TEA	0,019	0,020	0,020	0,021							0,002	
REFINED SUGAR & SWEETENERS TOTAL	0,022	0,018	0,021	0,016							0,006	
REFINED SUGAR	0,017	0,021	0,017	0,021							0,023	
OILCROPS TOTAL	0,014	0,012	0,013	0,011								
OLIVES	-0,069	-0,069	-0,064	-0,068	-0,366	-0,304			-0,031	-0,031	-0,161	
TREENUTS	-0,033	-0,028	-0,030	-0,027							-0,033	
PLANT OILS TOTAL	0,004	0,006	0,006	0,007								
OLIVE OIL	-0,008	-0,008	-0,006	-0,007								
SOYBEAN OIL	0,066	0,059	0,062	0,056		0,011	0,189	0,138	0,173	0,173	0,080	0,141
SUNFLOWER OIL	-0,022	-0,016	-0,022	-0,015								
CEREALS TOTAL	0,018	0,025	0,015	0,026							0,028	
MAIZE	0,035	0,021	0,031	0,021							0,017	
RYE	-0,042	-0,037	-0,040	-0,035							-0,033	
WHEAT	0,013	0,011	0,014	0,013								
POTATOES	-0,027	-0,026	-0,026	-0,025							-0,014	
LEGUMES TOTAL	0,010	0,015	-0,031	-0,020	-0,011							
VEGETABLES TOTAL	-0,041	-0,044	-0,037	-0,041							-0,064	
ONIONS	-0,035	-0,036	-0,033	-0,034							-0,061	
TOMATOES	-0,024	-0,026	-0,020	-0,025							-0,038	
SPICES	-0,039	-0,039	-0,036	-0,039							-0,049	
PLANT PROTEIN	-0,018	0,009	-0,016	-0,021							-0,017	
PLANT FAT	-0,008	-0,010	-0,006	-0,008								
MEAT TOTAL	0,045	0,046	0,042	0,046							0,084	
BEEF	0,018	0,026	0,018	0,026							0,019	
PORK	0,068	0,049	0,064	0,048	0,192	0,202	0,030		0,078	0,078	-0,031	0,020
POULTRY	0,028	0,033	0,027	0,032							0,026	
MEAT PROTEIN	-0,023	-0,019	-0,020	-0,017							0,048	
MEAT FAT	0,048	0,035	0,046	0,034					0,035	0,035	0,056	0,031
BEEF & PORK FAT	0,049	0,039	0,047	0,038					0,064	0,064	0,076	0,050
DAIRY TOTAL	0,040	0,042	0,039	0,042	0,123	0,057					0,080	
MILK	0,020	0,018	0,018	0,017							0,012	
CHEESE	-0,041	-0,040	-0,039	-0,039							-0,057	
DAIRY PROTEIN	0,019	0,014	0,020	0,015							0,012	
DAIRY FAT	-0,053	-0,041	-0,050	-0,042							-0,073	
MILK PROTEIN	-0,012	-0,013	-0,011	-0,012								
MILK FAT	0,031	0,025	0,027	0,023							0,040	
BUTTER & GHEE	0,021	0,020	0,019	0,019							0,001	
EDIBLE OFFALS	0,012	0,016	0,011	0,015								
FISH & SEAFOOD	0,036	0,044	0,032	0,042							0,050	
FISH & SEAFOOD FAT	0,048	0,058	0,045	0,055					0,075	0,075	0,093	0,025
EGGS TOTAL	-0,012	-0,014	-0,011	-0,013							0,050	
LARD	0,048	0,051	0,044	0,049							0,098	
HONEY	-0,023	-0,021	-0,023	-0,022							-0,014	
ANIMAL PROTEIN	0,037	0,025	0,037	0,025	0,057						0,010	
ANIMAL FAT	0,044	0,037	0,045	0,037					0,096	0,096	0,040	0,104
ANIMAL FAT & ANIMAL PROTEIN	0,030	0,042	0,029	0,041					0,076	0,076	0,063	0,091
TOTAL PROTEIN	0,038	0,029	0,036	0,029							0,026	
TOTAL FAT	0,042	0,039	0,038	0,036					0,020	0,020	0,061	0,040
TOTAL FAT & TOTAL PROTEIN	-0,002	-0,007	-0,002	-0,006							0,008	
% CA energy	-0,014	-0,033	-0,014	-0,030								-0,024
% PC CARB energy	-0,002	-0,011	-0,005	-0,010								
% Plant food energy	-0,063	-0,045	-0,060	-0,043	-0,136	-0,209	-0,265	-0,230	-0,148	-0,148	-0,095	-0,134
TOTAL ENERGY	-0,035	-0,036	-0,029	-0,032							-0,055	
VEGETABLES & CEREALS	-0,045	-0,037	-0,044	-0,035	-0,160	-0,134			-0,028	-0,028	-0,043	
MILK & VEGETABLES	-0,025	-0,030	-0,025	-0,029							-0,018	
MILK & VEG. & CEREALS	-0,031	-0,043	-0,031	-0,041					-0,023	-0,023	-0,048	
PROTEIN INDEX	0,011	0,013	0,012	0,010								
Smoking - women (1990 - 2009)	-0,059	-0,049	-0,055	-0,049	-0,172	-0,182					-0,110	
BMI - women (1990-2008)	-0,014	-0,010	-0,013	-0,007								
Raised cholesterol - women (2008)	0,043	0,040	0,040	0,040					0,032	0,032	0,067	0,036

Multiple myeloma - men

Ridge cross-validation

Ridge Models

Model	Penalty	Regularization "R Square" (1-Error)	Standardized Sum of Coefficients	Apparent Prediction Error	Expected Prediction Error		
					Estimate	Std. Error	N
13 (opt & pars)	,240	,987	,021	,013	,602	,128	25

Ridge bootstrap

Ridge Models

Model	Penalty	Regularization "R Square" (1-Error)	Standardized Sum of Coefficients	Apparent Prediction Error	Expected Prediction Error		
					Estimate	Std. Error	N
48 (opt)	,940	,956	,002	,044	,364	,146	27
51 (pars)	1,000	,953	,002	,047	,422	,131	28

LASSO cross-validation

LASSO Models

Model	Penalty	Regularization "R Square" (1-Error)	Number of Selected Predictors	Standardized Sum of Coefficients	Apparent Prediction Error	Expected Prediction Error		
						Estimate	Std. Error	N
10 (opt)	,180	,893	10	,026	,107	,884	,491	32
51 (pars)	1,000	,392	3	,006	,608	1,154	,777	38

LASSO bootstrap

LASSO Models

Model	Penalty	Regularization "R Square" (1-Error)	Number of Selected Predictors	Standardized Sum of Coefficients	Apparent Prediction Error	Expected Prediction Error		
						Estimate	Std. Error	N
19 (opt)	,360	,744	11	,022	,256	,709	,317	38
51 (pars)	1,000	,392	3	,006	,608	,991	,608	38

Elastic net cross-validation

Elastic Net Models

Model	Ridge Penalty	LASSO Penalty	Regularization "R Square" (1-Error)	Number of Selected Predictors	Standardized Sum of Coefficients	Apparent Prediction Error	Expected Prediction Error		
							Estimate	Std. Error	N
276 (opt)	0.5	,400	,897	9	,256	,103	,588	,242	35
433 (pars)	0.8	,480	,895	9	,260	,105	,777	,288	37

Elastic net bootstrap

Elastic Net Models

Model	Ridge Penalty	LASSO Penalty	Regularization "R Square" (1-Error)	Number of Selected Predictors	Standardized Sum of Coefficients	Apparent Prediction Error	Expected Prediction Error		
							Estimate	Std. Error	N
269 (opt)	0.5	,260	,896	17	,322	,104	,578	,246	38
554 (pars)	1.0	,860	,704	14	,160	,296	,797	,380	38

Regression models with *beta* coefficients.

Model	13	13	48	51	10	51	19	51	276	433	269	554
**Multiple myeloma - men**	**ridge optimal cross**	**ridge selected cross**	**ridge optimal bootstrap**	**ridge selected bootstrap**	**LASSO optimal cross**	**LASSO selected cross**	**LASSO optimal bootstrap**	**LASSO selected bootstrap**	**elastic optimal cross**	**elastic selected cross**	**elastic optimal bootstrap**	**elastic selected bootstrap**
FRUITS TOTAL	-0,121	-0,121	-0,045	-0,043								
APPLES	0,051	0,051	-0,054	-0,048	-0,086							
BANANAS	-0,197	-0,197	0,039	0,023			-0,040					0,018
GRAPES	-0,078	-0,078	-0,023	-0,033							-0,017	
ORANGES & MANDARINS	0,181	0,181	-0,013	-0,006	0,104		0,238					
ALCOHOLIC BEVERAGES TOTAL	-0,055	-0,055	-0,050	-0,046					0,468	0,425	0,405	
BEER	0,083	0,083	-0,013	-0,006								
DISTILLED BEVERAGES	0,103	0,103	0,040	0,040								
WINE	0,182	0,182	0,070	0,081								
COCOA BEANS	-0,009	-0,009	-0,015	-0,014							0,013	
COFFEE	0,269	0,269	0,058	0,069					0,047	0,011	0,109	0,081
TEA	0,117	0,117	0,034	0,040	0,059							
REFINED SUGAR & SWEETENERS TOTAL	-0,084	-0,084	-0,026	-0,028								
REFINED SUGAR	0,071	0,071	0,028	0,024							0,012	
OILCROPS TOTAL	-0,063	-0,063	0,026	0,025								
OLIVES	-0,086	-0,086	-0,046	-0,048					-0,110	-0,099	-0,123	
TREENUTS	0,084	0,084	0,071	0,071							0,011	
PLANT OILS TOTAL	0,168	0,168	0,010	0,014								
OLIVE OIL	0,088	0,088	0,027	0,033								
SOYBEAN OIL	0,207	0,207	0,076	0,083	0,251		0,106					0,084
SUNFLOWER OIL	-0,096	-0,096	-0,051	-0,049								
CEREALS TOTAL	0,280	0,280	0,008	0,017								
MAIZE	0,001	0,001	0,015	0,007								
RYE	0,086	0,086	0,026	0,027								
WHEAT	-0,124	-0,124	-0,013	-0,016							-0,016	
POTATOES	0,020	0,020	-0,020	-0,018								
LEGUMES TOTAL	0,118	0,118	0,034	0,039								
VEGETABLES TOTAL	-0,090	-0,090	-0,027	-0,032								
ONIONS	0,055	0,055	-0,075	-0,062	-0,224	-0,038	-0,031	-0,038			-0,018	
TOMATOES	-0,086	-0,086	-0,047	-0,045								
SPICES	-0,050	-0,050	-0,055	-0,054								
PLANT PROTEIN	-0,052	-0,052	0,027	0,022								
PLANT FAT	0,167	0,167	0,021	0,028								
MEAT TOTAL	0,036	0,036	0,047	0,042					0,088	0,103	0,129	0,010
BEEF	0,080	0,080	0,031	0,031								
PORK	-0,138	-0,138	-0,038	-0,035					0,172	0,181	0,172	
POULTRY	0,025	0,025	0,012	0,010								
MEAT PROTEIN	0,149	0,149	0,029	-0,006							0,001	
MEAT FAT	-0,174	-0,174	0,030	0,022								
BEEF & PORK FAT	-0,167	-0,167	-0,029	-0,031								
DAIRY TOTAL	0,236	0,236	0,046	0,064								
MILK	0,029	0,029	0,016	0,014						-0,076		
CHEESE	-0,098	-0,098	0,050	0,046								0,066
DAIRY PROTEIN	-0,200	-0,200	0,037	0,033								
DAIRY FAT	-0,082	-0,082	-0,029	-0,029								
MILK PROTEIN	-0,069	-0,069	-0,011	-0,013						-0,068		
MILK FAT	-0,040	-0,040	0,017	0,012								
BUTTER & GHEE	0,171	0,171	0,045	0,052			0,040					
EDIBLE OFFALS	-0,036	-0,036	-0,027	-0,025			0,006					
FISH & SEAFOOD	0,118	0,118	0,041	0,048					0,014		0,058	0,036
FISH & SEAFOOD FAT	0,117	0,117	0,050	0,058	0,145	0,074	0,255	0,074	0,013		0,058	0,070
EGGS TOTAL	0,050	0,050	-0,034	-0,033					0,083	0,103	0,080	
LARD	0,019	0,019	-0,046	-0,041								
HONEY	0,010	0,010	-0,018	-0,017								
ANIMAL PROTEIN	0,060	0,060	-0,007	-0,007			-0,046					0,021
ANIMAL FAT	0,257	0,257	0,035	0,048	0,395	0,206	0,225	0,206				0,031
ANIMAL FAT & ANIMAL PROTEIN	0,084	0,084	0,028	0,029			0,076					0,045
TOTAL PROTEIN	-0,189	-0,189	0,051	0,049								
TOTAL FAT	-0,133	-0,133	0,018	0,009								
TOTAL FAT & TOTAL PROTEIN	-0,257	-0,257	0,032	0,028	0,048							
% CA energy	-0,093	-0,093	-0,037	-0,039								
% PC CARB energy	-0,253	-0,253	0,020	0,010	0,023							
% Plant food energy	0,249	0,249	-0,041	-0,031			0,070					-0,066
TOTAL ENERGY	0,119	0,119	0,022									
VEGETABLES & CEREALS	-0,165	-0,165	-0,019	-0,022								
MILK & VEGETABLES	-0,010	-0,010	-0,021	-0,026								
MILK & VEG. & CEREALS	-0,029	-0,029	-0,030	-0,036								
PROTEIN INDEX	-0,013	-0,013	-0,002	-0,005								
Smoking - men (1990-2009)	0,018	0,018	-0,079	-0,076	-0,009				-0,089	-0,099	-0,132	-0,091
BMI – men (1990-2008)	-0,166	-0,166	-0,039	-0,038								
Raised cholesterol - men (2008)	-0,061	-0,061	0,051	0,039							0,009	0,132

Multiple myeloma - women

Ridge cross-validation

Ridge Models

Model	Penalty	Regularization "R Square" (1-Error)	Standardized Sum of Coefficients	Apparent Prediction Error	Expected Prediction Error		
					Estimate	Std. Error	N
17 (opt)	,320	,995	,002	,005	,250	,099	26
51 (pars)	1,000	,968	,001	,032	,311	,110	26

Ridge bootstrap

Ridge Models

Model	Penalty	Regularization "R Square" (1-Error)	Standardized Sum of Coefficients	Apparent Prediction Error	Expected Prediction Error		
					Estimate	Std. Error	N
34 (opt)	,660	,983	,001	,017	,240	,090	29
49 (pars)	,960	,970	,001	,030	,318	,114	29

LASSO cross-validation

LASSO Models

Model	Penalty	Regularization "R Square" (1-Error)	Number of Selected Predictors	Standardized Sum of Coefficients	Apparent Prediction Error	Expected Prediction Error		
						Estimate	Std. Error	N
10 (opt)	,180	,968	8	,020	,032	,603	,282	36
20 (pars)	,380	,902	5	,016	,098	,767	,433	36

LASSO bootstrap

LASSO Models

Model	Penalty	Regularization "R Square" (1-Error)	Number of Selected Predictors	Standardized Sum of Coefficients	Apparent Prediction Error	Expected Prediction Error		
						Estimate		
22 (opt)	,420	,885	5	,015	,115	,911	22 (opt)	,420
51 (pars)	1,000	,579	2	,007	,421	1,289	51 (pars)	1,000

Elastic net cross-validation

Elastic Net Models

Model	Ridge Penalty	LASSO Penalty	Regularization "R Square" (1-Error)	Number of Selected Predictors	Standardized Sum of Coefficients	Apparent Prediction Error	Expected Prediction Error		
							Estimate	Std. Error	N
56 (opt & pars)	0,1	,080	,962	58	,690	,038	,113	,050	29

Elastic net bootstrap

Elastic Net Models

Model	Ridge Penalty	LASSO Penalty	Regularization "R Square" (1-Error)	Number of Selected Predictors	Standardized Sum of Coefficients	Apparent Prediction Error	Expected Prediction Error		
							Estimate	Std. Error	N
206 (opt)	0,4	,020	,901	70	3,267	,099	,537	,147	32
473 (pars)	0,9	,260	,806	24	,462	,194	,674	,324	38

Regression models with *beta* coefficients.

Model	17	51	34	49	10	20	22	51	56	56	206	473
**Multiple myeloma - women**	ridge optimal cross	ridge selected cross	ridge optimal bootstrap	ridge selected bootstrap	LASSO optimal cross	LASSO selected cross	LASSO optimal bootstrap	LASSO selected bootstrap	elastic optimal cross	elastic selected cross	elastic optimal bootstrap	elastic selected bootstrap
FRUITS TOTAL	0,032	0,020	0,020	0,023							-0,369	
APPLES	-0,025	-0,042	-0,048	-0,044					0,271	0,271	0,180	
BANANAS	0,015	0,029	0,032	0,026							-0,316	
GRAPES	-0,051	-0,049	-0,051	-0,051					0,153	0,153	-0,030	-0,041
ORANGES & MANDARINS	-0,010	0,024	0,022	0,023					0,260	0,260	0,302	
ALCOHOLIC BEVERAGES TOTAL	-0,026	-0,021	-0,018	-0,020	0,013				-0,229	-0,229	-0,075	0,155
BEER	-0,005	0,035	-0,014	0,036					0,378	0,378	0,207	
DISTILLED BEVERAGES	-0,016	-0,023	-0,027	-0,022					0,046	0,046	0,113	
WINE	0,104	0,072	0,085	0,070	0,008				0,180	0,180	0,381	0,117
COCOA BEANS	-0,035	-0,006	-0,010	-0,004					-0,246	-0,246	-0,032	
COFFEE	-0,006	0,027	0,029	0,030					-0,072	-0,072	0,302	
TEA	-0,023	-0,032	-0,039	-0,034					0,038	0,038	0,133	
REFINED SUGAR & SWEETENERS TOTAL	-0,046	0,006	0,004	0,012					-0,081	-0,081	-0,137	
REFINED SUGAR	0,087	0,049	0,057	0,048					0,066	0,066	0,121	0,028
OILCROPS TOTAL	-0,068	-0,038	-0,045	-0,038							-0,071	
OLIVES	-0,046	-0,043	-0,045	-0,043							-0,025	
TREENUTS	0,039	0,016	0,023	0,016					0,127	0,127	0,137	
PLANT OILS TOTAL	0,017	0,021	0,023	0,024					-0,019	-0,019	0,085	
OLIVE OIL	0,015	0,019	0,024	0,019							0,146	
SOYBEAN OIL	0,041	0,044	0,044	0,049					-0,284	-0,284	0,238	0,072
SUNFLOWER OIL	-0,012	-0,033	-0,031	-0,037					0,373	0,373	-0,097	
CEREALS TOTAL	0,061	0,021	0,026	0,019					0,064	0,064	0,435	
MAIZE	0,032	0,024	0,031	0,025							-0,238	
RYE	0,031	0,035	0,035	0,038					0,008	0,008	0,066	
WHEAT	-0,052	-0,040	-0,045	-0,044					-0,055	-0,055	-0,167	
POTATOES	0,023	0,020	0,022	-0,011					0,163	0,163	0,038	
LEGUMES TOTAL	-0,031	0,004	-0,049	0,005					-0,027	-0,027	0,092	-0,028
VEGETABLES TOTAL	-0,040	-0,033	-0,032	-0,033					-0,298	-0,298	-0,093	-0,010
ONIONS	-0,038	-0,033	-0,034	-0,029					-0,303	-0,303	0,088	
TOMATOES	-0,052	-0,036	-0,038	-0,037							-0,059	
SPICES	-0,073	-0,066	-0,067	-0,064					-0,127	-0,127	-0,103	-0,078
PLANT PROTEIN	0,010	0,018	0,022	0,019					-0,392	-0,392	-0,196	
PLANT FAT	0,039	0,030	0,035	0,034					-0,490	-0,490	0,130	
MEAT TOTAL	0,111	0,079	0,086	0,076	0,131	0,214	0,228	0,272	0,364	0,364	0,098	0,187
BEEF	0,030	0,018	0,017	0,014					0,190	0,190	0,080	
PORK	0,076	0,094	0,103	0,100	0,588	0,453	0,425	0,147	-0,076	-0,076	-0,225	0,234
POULTRY	0,058	0,039	0,040	0,034					0,049	0,049	-0,027	0,005
MEAT PROTEIN	0,093	-0,012	0,049	-0,015					0,357	0,357	0,373	0,082
MEAT FAT	-0,010	-0,003	-0,005	-0,005							-0,273	
BEEF & PORK FAT	-0,015	-0,005	-0,005	-0,005					0,003	0,003	-0,275	
DAIRY TOTAL	0,072	0,055	0,055	0,059	0,129	0,182	0,181		0,235	0,235	0,438	0,111
MILK	0,033	0,012	0,015	0,010							0,009	
CHEESE	0,025	0,045	0,045	0,051					-0,193	-0,193	-0,308	0,032
DAIRY PROTEIN	0,015	0,013	0,009	0,015					0,076	0,076	-0,294	
DAIRY FAT	0,059	0,060	0,062	0,063	0,102				0,144	0,144	0,026	0,075
MILK PROTEIN	-0,019	-0,014	-0,019	-0,014					-0,067	-0,067	-0,065	
MILK FAT	-0,011	-0,001	-0,004	0,006							-0,066	
BUTTER & GHEE	-0,047	-0,013	-0,021	-0,009					-0,163	-0,163	0,161	
EDIBLE OFFALS	-0,009	0,020	0,017	0,022					0,007	0,007	0,079	
FISH & SEAFOOD	0,068	0,054	0,054	0,054					0,287	0,287	0,182	0,098
FISH & SEAFOOD FAT	0,060	0,053	0,052	0,054	0,060	0,025	0,024		0,318	0,318	0,184	0,100
EGGS TOTAL	0,116	-0,034	-0,035	-0,034					0,098	0,098	0,099	0,144
LARD	-0,011	0,037	0,040	0,035					0,170	0,170	0,107	
HONEY	-0,024	-0,014	-0,019	-0,012					0,014	0,014	0,058	
ANIMAL PROTEIN	-0,043	-0,005	-0,008	-0,003					-0,558	-0,558	-0,058	0,032
ANIMAL FAT	-0,041	0,022	0,017	0,032					-0,968	-0,968	0,244	0,007
ANIMAL FAT & ANIMAL PROTEIN	0,093	0,046	0,047	0,042					0,428	0,428	0,224	0,071
TOTAL PROTEIN	0,016	0,037	0,037	0,038							-0,255	
TOTAL FAT	0,026	0,024	0,024	0,020					0,300	0,300	-0,202	
TOTAL FAT & TOTAL PROTEIN	-0,029	0,022	0,019	0,025							-0,384	
% CA energy	-0,073	-0,025	-0,025	-0,018					-0,267	-0,267	-0,205	
% PC CARB energy	0,012	-0,020	0,015	-0,022					-0,129	-0,129	-0,420	
% Plant food energy	-0,034	-0,054	-0,053	-0,055					-0,006	-0,006	0,345	-0,098
TOTAL ENERGY	-0,042	-0,027	-0,032	-0,019					-0,290	-0,290	0,062	
VEGETABLES & CEREALS	0,015	0,007	0,008	0,006					0,650	0,650	-0,194	-0,029
MILK & VEGETABLES	-0,014	-0,020	-0,014	-0,020					-0,283	-0,283	-0,030	
MILK & VEG. & CEREALS	-0,023	-0,019	-0,017	-0,017					-0,148	-0,148	-0,066	
PROTEIN INDEX	-0,017	0,012	0,011	0,015					-0,311	-0,311		
Smoking - women (1990 - 2009)	-0,058	-0,046	-0,044	-0,047	-0,124	-0,040	-0,013		0,169	0,169	-0,130	-0,060
BMI - women (1990-2008)	-0,002	-0,012	0,009	0,005							0,167	
Raised cholesterol - women (2008)	0,023	0,025	0,025	0,020					0,130	0,130	-0,162	

Leukaemia – men

Ridge cross-validation

Ridge Models

Model	Penalty	Regularization "R Square" (1-Error)	Standardized Sum of Coefficients	Apparent Prediction Error	Expected Prediction Error		
					Estimate	Std. Error	N
30 (opt)	,580	,989	,003	,011	,364	,176	23
51 (pars)	1,000	,977	,002	,023	,396	,209	23

Ridge bootstrap

Ridge Models

Model	Penalty	Regularization "R Square" (1-Error)	Standardized Sum of Coefficients	Apparent Prediction Error	Expected Prediction Error		
					Estimate	Std. Error	N
50 (opt)	,980	,977	,002	,023	,192	,123	27
51 (pars)	1,000	,977	,002	,023	,295	,159	28

LASSO cross-validation

LASSO Models

Model	Penalty	Regularization "R Square" (1-Error)	Number of Selected Predictors	Standardized Sum of Coefficients	Apparent Prediction Error	Expected Prediction Error		
						Estimate	Std. Error	N
50 (opt)	,980	,620	5	,011	,380	,216	,062	36
51 (pars)	1,000	,607	5	,011	,393	,217	,058	36

LASSO bootstrap

LASSO Models

Model	Penalty	Regularization "R Square" (1-Error)	Number of Selected Predictors	Standardized Sum of Coefficients	Apparent Prediction Error	Expected Prediction Error		
						Estimate	Std. Error	N
37 (opt)	,720	,766	4	,016	,234	,884	,529	39
44 (pars)	,860	,694	4	,013	,306	1,161	,596	39

Elastic net cross-validation

Elastic Net Models

Model	Ridge Penalty	LASSO Penalty	Regularization "R Square" (1-Error)	Number of Selected Predictors	Standardized Sum of Coefficients	Apparent Prediction Error	Expected Prediction Error		
							Estimate	Std. Error	N
153 (opt & pars)	0,2	1,000	,673	7	,040	,327	,194	,057	36

Elastic net bootstrap

Elastic Net Models

Model	Ridge Penalty	LASSO Penalty	Regularization "R Square" (1-Error)	Number of Selected Predictors	Standardized Sum of Coefficients	Apparent Prediction Error	Expected Prediction Error		
							Estimate	Std. Error	N
257 (opt)	0,5	,020	,829	57	,771	,171	,449	,249	31
547 (pars)	1,0	,720	,931	13	,293	,069	,660	,350	39

Regression models with *beta* coefficients.

Model	30	51	50	51	50	51	37	44	153	153	257	547
**Leukaemia - men**	ridge optimal cross	ridge selected cross	ridge optimal bootstrap	ridge selected bootstrap	LASSO optimal cross	LASSO selected cross	LASSO optimal bootstrap	LASSO selected bootstrap	elastic optimal cross	elastic selected cross	elastic optimal bootstrap	elastic selected bootstrap
FRUITS TOTAL	0,017	0,016	0,016	0,016								
APPLES	-0,017	-0,018	-0,018	-0,018								
BANANAS	0,076	0,065	0,063	0,065							0,112	0,012
GRAPES	-0,032	-0,029	-0,029	-0,029							-0,036	
ORANGES & MANDARINS	-0,009	-0,009	-0,007	-0,009							-0,003	
ALCOHOLIC BEVERAGES TOTAL	0,086	0,083	0,082	0,083							0,146	0,072
BEER	-0,011	-0,009	-0,008	-0,009								
DISTILLED BEVERAGES	-0,005	0,011	0,011	0,011								
WINE	-0,011	-0,010	-0,009	-0,010							-0,003	
COCOA BEANS	0,042	0,041	0,042	0,041							0,053	
COFFEE	-0,008	-0,007	-0,006	-0,007							0,008	
TEA	0,011	0,008	0,008	0,008							0,009	
REFINED SUGAR & SWEETENERS TOTAL	0,037	0,032	0,029	0,032							0,045	
REFINED SUGAR	0,054	0,051	0,052	0,051							0,086	0,009
OILCROPS TOTAL	-0,026	-0,024	-0,025	-0,024							-0,025	
OLIVES	-0,049	-0,045	-0,045	-0,045							-0,073	
TREENUTS	0,026	0,020	0,018	0,020							0,023	
PLANT OILS TOTAL	0,017	0,018	0,018	0,018								
OLIVE OIL	0,013	0,014	0,015	0,014								
SOYBEAN OIL	0,013	0,011	0,012	0,011							0,003	
SUNFLOWER OIL	0,038	0,034	0,036	0,034							0,051	
CEREALS TOTAL	-0,026	-0,026	-0,020	-0,026							-0,016	
MAIZE	0,017	-0,044	-0,052	-0,044							0,001	-0,036
RYE	0,011	0,009	0,010	0,009								
WHEAT	-0,034	-0,033	-0,033	-0,033							-0,049	
POTATOES	0,046	0,040	0,038	0,040							0,062	
LEGUMES TOTAL	0,028	0,024	0,025	0,024							0,035	
VEGETABLES TOTAL	0,026	0,025	0,025	0,025							0,031	
ONIONS	-0,011	-0,010	-0,009	-0,010								
TOMATOES	-0,017	-0,015	-0,017	-0,015							0,027	
SPICES	-0,014	-0,012	-0,012	-0,012							-0,014	
PLANT PROTEIN	-0,022	-0,017	-0,019	-0,017							0,025	
PLANT FAT	0,042	0,041	0,043	0,041							0,062	
MEAT TOTAL	0,078	-0,013	-0,014	-0,013	0,266	0,267	0,288	0,278	0,203	0,203	0,119	0,189
BEEF	0,052	0,043	0,047	0,043							0,083	
PORK	0,079	0,077	0,074	0,077	0,064	0,059			0,064	0,064	0,125	0,139
POULTRY	0,030	0,028	0,028	0,028							0,020	
MEAT PROTEIN	-0,017	-0,012	-0,011	-0,012							0,049	
MEAT FAT	0,064	0,057	0,053	0,057	0,084	0,085	0,169	0,122	0,079	0,079	0,082	0,102
BEEF & PORK FAT	-0,003	0,020	0,017	0,020								
DAIRY TOTAL	-0,024	-0,018	-0,013	-0,018							-0,027	
MILK	-0,013	-0,016	-0,015	-0,016							-0,020	
CHEESE	0,015	0,014	0,014	0,014							0,006	
DAIRY PROTEIN	-0,020	-0,018	-0,019	-0,018							-0,026	
DAIRY FAT	-0,029	-0,027	-0,026	-0,027							-0,030	
MILK PROTEIN	-0,016	-0,018	-0,018	-0,018							-0,026	
MILK FAT	-0,018	-0,015	-0,016	-0,015							-0,021	
BUTTER & GHEE	-0,006	-0,005	-0,005	-0,005							0,008	
EDIBLE OFFALS	-0,007	-0,007	-0,004	-0,007								
FISH & SEAFOOD	-0,011	-0,008	0,020	-0,008							0,024	
FISH & SEAFOOD FAT	0,021	0,020	0,019	0,020							0,022	
EGGS TOTAL	0,082	0,078	0,077	0,078	0,008	0,005	0,155	0,111	0,067	0,067	0,142	0,148
LARD	-0,011	0,002	0,005	0,002								
HONEY	0,024	0,023	0,023	0,023							0,024	
ANIMAL PROTEIN	-0,009	0,034	-0,007	0,034							0,031	
ANIMAL FAT	0,059	0,054	0,058	0,054	0,031	0,023	0,011	0,021	0,060	0,060	0,100	0,096
ANIMAL FAT & ANIMAL PROTEIN	0,039	0,037	0,044	0,037							0,059	
TOTAL PROTEIN	0,020	0,019	0,017	0,019							0,005	
TOTAL FAT	0,064	0,055	0,055	0,055							0,086	0,076
TOTAL FAT & TOTAL PROTEIN	0,067	0,062	0,058	0,062					0,023	0,023	0,090	0,085
% CA energy	-0,017	-0,016	-0,023	-0,016								
% PC CARB energy	-0,049	-0,044	-0,051	-0,044					-0,015	-0,015	-0,096	-0,077
% Plant food energy	-0,048	-0,046	-0,043	-0,046								
TOTAL ENERGY	0,027	0,026	0,028	0,026							0,013	
VEGETABLES & CEREALS	-0,011	-0,011	-0,015	-0,011							-0,014	
MILK & VEGETABLES	0,013	-0,001	0,007	-0,001								
MILK & VEG. & CEREALS	0,014	0,013	0,011	0,013							0,014	
PROTEIN INDEX	-0,018	-0,015	-0,014	-0,015							-0,009	
Smoking - men (1990-2009)	-0,038	-0,037	-0,036	-0,037							-0,049	
BMI – men (1990-2008)	-0,026	-0,020	-0,023	-0,020							-0,027	
Raised cholesterol - men (2008)	0,046	0,043	0,045	0,043							0,073	0,073

Leukaemia - women

Ridge cross-validation

Ridge Models

Model	Penalty	Regularization "R Square" (1-Error)	Standardized Sum of Coefficients	Apparent Prediction Error	Expected Prediction Error		
					Estimate	Std. Error	N
36 (opt)	,700	,991	,002	,009	,097	,025	26
51 (pars)	1,000	,986	,002	,014	,106	,027	26

Ridge bootstrap

Ridge Models

Model	Penalty	Regularization "R Square" (1-Error)	Standardized Sum of Coefficients	Apparent Prediction Error	Expected Prediction Error		
					Estimate	Std. Error	N
51 (opt & pars)	1,000	,986	,002	,014	,097	,036	30

LASSO cross-validation

LASSO Models

Model	Penalty	Regularization "R Square" (1-Error)	Number of Selected Predictors	Standardized Sum of Coefficients	Apparent Prediction Error	Expected Prediction Error		
						Estimate	Std. Error	N
12 (opt)	,220	,955	4	,023	,045	,935	,771	36
33 (pars)	,640	,862	2	,017	,138	1,239	,865	38

LASSO bootstrap

LASSO Models

Model	Penalty	Regularization "R Square" (1-Error)	Number of Selected Predictors	Standardized Sum of Coefficients	Apparent Prediction Error	Expected Prediction Error		
						Estimate	Std. Error	N
17 (opt)	,320	,938	3	,021	,062	,604	,413	39
50 (pars)	,980	,724	2	,013	,276	,954	,623	39

Elastic net cross-validation

Elastic Net Models

Model	Ridge Penalty	LASSO Penalty	Regularization "R Square" (1-Error)	Number of Selected Predictors	Standardized Sum of Coefficients	Apparent Prediction Error	Expected Prediction Error		
							Estimate	Std. Error	N
162 (opt & pars)	0,3	,160	,954	19	,129	,046	,064	,026	33

Elastic net bootstrap

Elastic Net Models

Model	Ridge Penalty	LASSO Penalty	Regularization "R Square" (1-Error)	Number of Selected Predictors	Standardized Sum of Coefficients	Apparent Prediction Error	Expected Prediction Error		
							Estimate	Std. Error	N
313 (opt)	0,6	,180	,858	19	,499	,142	,199	,118	37
531 (pars)	1,0	,900	,939	10	,230	,061	,316	,248	39

Regression models with *beta* coefficients.

Model	36	51	51	51	12	33	17	50	162	162	313	531
**Leukaemia - women**	ridge optimal cross	ridge selected cross	ridge optimal bootstrap	ridge selected bootstrap	LASSO optimal cross	LASSO selected cross	LASSO optimal bootstrap	LASSO selected bootstrap	elastic optimal cross	elastic selected cross	elastic optimal bootstrap	elastic selected bootstrap
FRUITS TOTAL	0,007	0,005	0,005	0,005								
APPLES	-0,024	-0,021	-0,021	-0,021								
BANANAS	0,042	0,071	0,071	0,071	0,031		0,003		0,061	0,061	0,064	
GRAPES	-0,034	-0,020	-0,020	-0,020								
ORANGES & MANDARINS	0,023	-0,004	-0,004	-0,004								
ALCOHOLIC BEVERAGES TOTAL	0,016	0,007	0,007	0,007								
BEER	0,023	0,015	0,015	0,015								
DISTILLED BEVERAGES	-0,021	0,022	0,022	0,022								
WINE	0,033	0,022	0,022	0,022								
COCOA BEANS	-0,011	-0,026	-0,026	-0,026								
COFFEE	-0,008	-0,025	-0,025	-0,025								
TEA	-0,013	-0,020	-0,020	-0,020								
REFINED SUGAR & SWEETENERS TOTAL	0,020	0,006	0,006	0,006								
REFINED SUGAR	-0,015	-0,006	-0,006	-0,006								
OILCROPS TOTAL	0,029	0,023	0,023	0,023								
OLIVES	0,022	0,022	0,022	0,022								
TREENUTS	0,003	-0,001	-0,001	-0,001								
PLANT OILS TOTAL	0,027	0,020	0,020	0,020					0,023	0,023	0,014	
OLIVE OIL	0,014	0,021	0,021	0,021								
SOYBEAN OIL	0,024	0,006	0,006	0,006								
SUNFLOWER OIL	-0,015	-0,012	-0,012	-0,012								
CEREALS TOTAL	-0,024	-0,038	-0,038	-0,038					-0,047	-0,047	-0,045	
MAIZE	-0,070	-0,053	-0,053	-0,053					-0,065	-0,065	-0,090	-0,021
RYE	0,027	0,017	0,017	0,017								
WHEAT	0,019	0,023	0,023	0,023								
POTATOES	0,022	0,017	0,017	0,017								
LEGUMES TOTAL	0,025	0,017	0,017	0,017								
VEGETABLES TOTAL	0,016	0,009	0,009	0,009								
ONIONS	-0,012	-0,035	-0,035	-0,035								
TOMATOES	-0,023	-0,022	-0,022	-0,022								
SPICES	-0,030	-0,027	-0,027	-0,027								
PLANT PROTEIN	-0,042	-0,025	-0,025	-0,025	-0,009				-0,009	-0,009	-0,015	
PLANT FAT	-0,007	-0,017	-0,017	-0,017								
MEAT TOTAL	0,058	0,083	0,083	0,083					0,098	0,098	0,120	0,088
BEEF	0,048	0,056	0,056	0,056					0,008	0,008	0,031	
PORK	0,021	0,020	0,020	0,020					0,010	0,010	0,027	
POULTRY	0,017	0,050	0,050	0,050								
MEAT PROTEIN	0,092	0,084	0,084	0,084					0,186	0,186	0,192	0,206
MEAT FAT	0,035	0,061	0,061	0,061					0,068	0,068	0,078	0,047
BEEF & PORK FAT	0,009	0,042	0,042	0,042								
DAIRY TOTAL	0,064	0,048	0,048	0,048					0,094	0,094	0,110	
MILK	0,026	0,028	0,028	0,028								
CHEESE	0,009	0,012	0,012	0,012								
DAIRY PROTEIN	0,026	0,028	0,028	0,028								
DAIRY FAT	0,043	0,053	0,053	0,053					0,036	0,036	0,060	
MILK PROTEIN	0,012	0,015	0,015	0,015								
MILK FAT	0,019	0,018	0,018	0,018								
BUTTER & GHEE	-0,020	-0,033	-0,033	-0,033								
EDIBLE OFFALS	0,014	-0,003	-0,003	-0,003								
FISH & SEAFOOD	0,027	0,028	0,028	0,028								
FISH & SEAFOOD FAT	-0,007											
EGGS TOTAL	0,029	0,038	0,038	0,038								
LARD	0,032	0,026	0,026	0,026								
HONEY	0,029	0,027	0,027	0,027								
ANIMAL PROTEIN	0,078	0,041	0,041	0,041	0,197	0,048	0,183	0,027	0,210	0,210	0,194	0,211
ANIMAL FAT	-0,014	0,012	0,012	0,012					0,031	0,031	0,060	0,025
ANIMAL FAT & ANIMAL PROTEIN	0,085	0,091	0,091	0,091	0,653	0,615	0,638	0,465	0,201	0,201	0,202	0,222
TOTAL PROTEIN	0,014	0,008	0,008	0,008								
TOTAL FAT	0,034	0,075	0,075	0,075					0,017	0,017	0,048	0,006
TOTAL FAT & TOTAL PROTEIN	0,035	0,037	0,037	0,037					0,021	0,021	0,045	0,011
% CA energy	-0,044	0,004	0,004	0,004								
% PC CARB energy	0,011	-0,024	-0,024	-0,024								
% Plant food energy	-0,058	0,021	0,021	0,021								
TOTAL ENERGY	-0,011	-0,024	-0,024	-0,024								
VEGETABLES & CEREALS	-0,033	-0,008	-0,008	-0,008								
MILK & VEGETABLES	0,011	0,015	0,015	0,015								
MILK & VEG. & CEREALS	-0,018	-0,011	-0,011	-0,011								
PROTEIN INDEX	-0,009	0,012	0,012	0,012								
Smoking - women (1990 - 2009)	-0,058	-0,068	-0,068	-0,068					-0,053	-0,053	-0,078	
BMI - women (1990-2008)	-0,010	-0,014	-0,014	-0,014								
Raised cholesterol - women (2008)	-0,026	-0,011	-0,011	-0,011					0,072	0,072	0,090	0,041

Esophageal cancer - men

Ridge cross-validation

Ridge Models

Model	Penalty	Regularization "R Square" (1-Error)	Standardized Sum of Coefficients	Apparent Prediction Error	Expected Prediction Error		
					Estimate	Std. Error	N
32 (opt)	,620	,972	,006	,028	,342	,107	25
51 (pars)	1,000	,952	,005	,048	,388	,114	25

Ridge bootstrap

Ridge Models

Model	Penalty	Regularization "R Square" (1-Error)	Standardized Sum of Coefficients	Apparent Prediction Error	Expected Prediction Error		
					Estimate	Std. Error	N
32 (opt)	,620	,972	,006	,028	,354	,128	27
51 (pars)	1,000	,952	,005	,048	,477	,178	28

LASSO cross-validation

LASSO Models

Model	Penalty	Regularization "R Square" (1-Error)	Number of Selected Predictors	Standardized Sum of Coefficients	Apparent Prediction Error	Expected Prediction Error		
						Estimate	Std. Error	N
8 (opt & pars)	,140	,954	47	,428	,046	,263	,100	32

LASSO bootstrap

LASSO Models

Model	Penalty	Regularization "R Square" (1-Error)	Number of Selected Predictors	Standardized Sum of Coefficients	Apparent Prediction Error	Expected Prediction Error		
						Estimate	Std. Error	N
29 (opt)	,560	,765	3	,020	,235	,558	,152	38
50 (pars)	,980	,561	1	,012	,439	,664	,147	38

Elastic net cross-validation

Elastic Net Models

Model	Ridge Penalty	LASSO Penalty	Regularization "R Square" (1-Error)	Number of Selected Predictors	Standardized Sum of Coefficients	Apparent Prediction Error	Expected Prediction Error		
							Estimate	Std. Error	N
8 (opt)	0.0	,140	,954	47	,428	,046	,263	,100	32
363 (pars)	0.7	,100	,824	40	,583	,176	,358	,076	27

Elastic net bootstrap

Elastic Net Models

Model	Ridge Penalty	LASSO Penalty	Regularization "R Square" (1-Error)	Number of Selected Predictors	Standardized Sum of Coefficients	Apparent Prediction Error	Expected Prediction Error		
							Estimate	Std. Error	N
158 (opt)	0.3	,080	,963	34	,552	,037	,430	,127	36
500 (pars)	0.9	,800	,752	4	,121	,248	,548	,159	38

Regression models with *beta* coefficients.

Model	32	51	32	51	8	8	29	51	8	363	158	500
**Esophageal cancer - men**	ridge optimal cross	ridge selected cross	ridge optimal bootstrap	ridge selected bootstrap	LASSO optimal cross	LASSO selected cross	LASSO optimal bootstrap	LASSO selected bootstrap	elastic optimal cross	elastic selected cross	elastic optimal bootstrap	elastic selected bootstrap
FRUITS TOTAL	-0,056	-0,038	-0,056	-0,038	0,237	0,237			0,237	-0,033	-0,029	
APPLES	-0,003	-0,006	-0,003	-0,006	-0,312	-0,312			-0,312			
BANANAS	0,029	0,020	0,029	0,020	0,198	0,198			0,198			
GRAPES	-0,077	-0,073	-0,077	-0,073			-0,061			-0,168	-0,115	-0,055
ORANGES & MANDARINS	-0,012	-0,014	-0,012	-0,014	-0,451	-0,451			-0,451			
ALCOHOLIC BEVERAGES TOTAL	-0,010	0,053	-0,010	0,053	1,010	1,010			1,010	0,068		
BEER	0,064	0,049	0,064	0,049	-0,783	-0,783			-0,783	0,075	0,064	
DISTILLED BEVERAGES	0,053	0,046	0,053	0,046	0,098	0,098			0,098	0,017		
WINE	0,077	0,058	0,077	0,058						0,063	0,066	
COCOA BEANS	-0,078	-0,058	-0,078	-0,058						-0,083	-0,099	
COFFEE	-0,063	-0,056	-0,063	-0,056	-0,755	-0,755			-0,755	-0,076	-0,094	
TEA	0,032	0,034	0,032	0,034	-0,618	-0,618			-0,618	0,028		
REFINED SUGAR & SWEETENERS TOTAL	0,019	0,034	0,019	0,034	0,019	0,019			0,019			
REFINED SUGAR	-0,013	-0,009	-0,013	-0,009	0,236	0,236			0,236			
OILCROPS TOTAL	-0,055	-0,048	-0,055	-0,048	-0,009	-0,009			-0,009	-0,054	-0,048	
OLIVES	-0,057	-0,054	-0,057	-0,054	-0,693	-0,693			-0,693	-0,099	-0,092	
TREENUTS	-0,035	-0,034	-0,035	-0,034						-0,039	-0,022	
PLANT OILS TOTAL	-0,026	-0,025	-0,026	-0,025						-0,001		
OLIVE OIL	-0,020	-0,024	-0,020	-0,024	-0,424	-0,424			-0,424			
SOYBEAN OIL	0,022	0,023	0,022	0,023	-0,114	-0,114			-0,114			
SUNFLOWER OIL	-0,059	-0,054	-0,059	-0,054	-0,539	-0,539			-0,539	-0,072	-0,045	
CEREALS TOTAL	-0,018	-0,029	-0,018	-0,029							0,002	
MAIZE	-0,032	-0,026	-0,032	-0,026						0,009	0,027	
RYE	-0,029	-0,019	-0,029	-0,019								
WHEAT	0,034	0,023	0,034	0,023	0,517	0,517			0,517			
POTATOES	0,067	0,066	0,067	0,066						0,080	0,029	
LEGUMES TOTAL	-0,025	-0,033	-0,025	-0,033						-0,037		
VEGETABLES TOTAL	-0,042	-0,034	-0,042	-0,034								
ONIONS	0,016	0,023	0,016	0,023								
TOMATOES	-0,062	-0,053	-0,062	-0,053	-0,069	-0,069	-0,034		-0,069	-0,119	-0,113	
SPICES	-0,013	-0,014	-0,013	-0,014								
PLANT PROTEIN	-0,032	-0,025	-0,032	-0,025								
PLANT FAT	-0,040	-0,031	-0,040	-0,031						-0,014	-0,006	
MEAT TOTAL	0,058	0,039	0,058	0,039	0,035	0,035			0,035	0,040	0,080	
BEEF	-0,014	-0,017	-0,014	-0,017	0,233	0,233			0,233			
PORK	0,012	0,017	0,012	0,017	-0,160	-0,160			-0,160			
POULTRY	0,030	0,021	0,030	0,021	0,017	0,017			0,017	0,010	0,015	
MEAT PROTEIN	0,067	0,046	0,067	0,046						0,068	0,095	
MEAT FAT	0,022	0,021	0,022	0,021								
BEEF & PORK FAT	-0,004	-0,003	-0,004	-0,003						0,024	0,022	
DAIRY TOTAL	0,121	0,101	0,121	0,101	-0,608	-0,608	0,585	0,395	-0,608	0,294	0,294	0,362
MILK	0,046	0,037	0,046	0,037						0,059	0,048	
CHEESE	0,021	0,024	0,021	0,024	0,070	0,070			0,070			
DAIRY PROTEIN	0,035	0,036	0,035	0,036	0,012	0,012			0,012	0,072	0,024	
DAIRY FAT	0,089	0,077	0,089	0,077	0,008	0,008			0,008	0,235	0,239	0,247
MILK PROTEIN	0,024	0,023	0,024	0,023	0,214	0,214			0,214	0,008		
MILK FAT	0,041	0,037	0,041	0,037	0,014	0,014			0,014	0,052	0,035	
BUTTER & GHEE	0,017	0,019	0,017	0,019	-0,658	-0,658			-0,658		-0,055	
EDIBLE OFFALS	0,018	0,022	0,018	0,022	-0,580	-0,580			-0,580			
FISH & SEAFOOD	0,063	0,051	0,063	0,051	-0,093	-0,093			-0,093	0,064	0,070	
FISH & SEAFOOD FAT	0,056	0,047	0,056	0,047	-0,043	-0,043			-0,043	0,062	0,048	
EGGS TOTAL	0,066	0,060	0,066	0,060						0,075	0,049	
LARD	0,086	0,071	0,086	0,071						0,087	0,056	
HONEY	0,033	0,024	0,033	0,024	0,159	0,159			0,159			
ANIMAL PROTEIN	0,030	0,045	0,030	0,045	0,493	0,493			0,493	0,056		
ANIMAL FAT	-0,045	-0,017	-0,045	-0,017							-0,048	
ANIMAL FAT & ANIMAL PROTEIN	0,082	0,055	0,082	0,055	0,653	0,653			0,653	0,084	0,150	
TOTAL PROTEIN	0,016	0,017	0,016	0,017	0,382	0,382			0,382			
TOTAL FAT	0,026	0,013	0,026	0,013								
TOTAL FAT & TOTAL PROTEIN	0,017	0,015	0,017	0,015								
% CA energy	0,018	0,030	0,018	0,030	-0,110	-0,110			-0,110	0,020		
% PC CARB energy	0,051	0,032	0,051	0,032	0,867	0,867			0,867	0,022	0,076	
% Plant food energy	-0,066	-0,064	-0,066	-0,064						-0,091	-0,067	-0,019
TOTAL ENERGY	0,015	0,025	0,015	0,025	-0,061	-0,061			-0,061			
VEGETABLES & CEREALS	-0,021	-0,033	-0,021	-0,033	-0,476	-0,476			-0,476	-0,037		
MILK & VEGETABLES	0,016	0,012	0,016	0,012	0,156	0,156			0,156			
MILK & VEG. & CEREALS	0,015	0,012	0,015	0,012	-0,040	-0,040			-0,040			
PROTEIN INDEX	0,043	0,042	0,043	0,042	0,112	0,112			0,112	0,046	0,012	
Smoking - men (1990-2009)	0,033	0,027	0,033	0,027	-0,048	-0,048			-0,048			
BMI – men (1990-2008)	-0,049	-0,040	-0,049	-0,040	-0,165	-0,165			-0,165			
Raised cholesterol - men (2008)	-0,005	-0,017	-0,005	-0,017	0,946	0,946			0,946			

Esophageal cancer – women

Ridge cross-validation

Ridge Models

Model	Penalty	Regularization "R Square" (1-Error)	Standardized Sum of Coefficients	Apparent Prediction Error	Expected Prediction Error		
					Estimate	Std. Error	N
16 (opt)	,300	,985	,011	,015	,544	,147	26
4 (pars)	,880	,967	,002	,033	,645	,318	26

Ridge bootstrap

Ridge Models

Model	Penalty	Regularization "R Square" (1-Error)	Standardized Sum of Coefficients	Apparent Prediction Error	Expected Prediction Error		
					Estimate	Std. Error	N
39 (opt)	,760	,972	,003	,028	,190	,056	29
51 (pars)	1,000	,963	,002	,037	,193	,060	29

LASSO cross-validation

LASSO Models

Model	Penalty	Regularization "R Square" (1-Error)	Number of Selected Predictors	Standardized Sum of Coefficients	Apparent Prediction Error	Expected Prediction Error		
						Estimate	Std. Error	N
25 (opt)	,480	,723	6	,017	,277	1,764	,940	38
51 (pars)	1,000	,413	2	,007	,587	1,945	,942	37

LASSO bootstrap

LASSO Models

Model	Penalty	Regularization "R Square" (1-Error)	Number of Selected Predictors	Standardized Sum of Coefficients	Apparent Prediction Error	Expected Prediction Error		
						Estimate	Std. Error	N
28 (opt)	,540	,689	4	,015	,311	1,050	,440	39
51 (pars)	1,000	,413	2	,007	,587	1,265	,550	39

Elastic net cross-validation

Elastic Net Models

Model	Ridge Penalty	LASSO Penalty	Regularization "R Square" (1-Error)	Number of Selected Predictors	Standardized Sum of Coefficients	Apparent Prediction Error	Expected Prediction Error		
							Estimate	Std. Error	N
107 (opt & pars)	0.2	,080	,964	37	,150	,036	,350	,071	26

Elastic net bootstrap

Elastic Net Models

Model	Ridge Penalty	LASSO Penalty	Regularization "R Square" (1-Error)	Number of Selected Predictors	Standardized Sum of Coefficients	Apparent Prediction Error	Expected Prediction Error		
							Estimate	Std. Error	N
205 (opt)	,400	,000	,897	71	1,000	,103	,400	,120	29
359 (pars)	0.7	,020	,725	69	,878	,275	,463	,100	29

Regression models with *beta* coefficients.

Model	16	45	39	51	25	51	28	51	107	107	205	359
**Esophageal cancer - women**	ridge optimal cross	ridge selected cross	ridge optimal bootstrap	ridge selected bootstrap	LASSO optimal cross	LASSO selected cross	LASSO optimal bootstrap	LASSO selected bootstrap	elastic optimal cross	elastic selected cross	elastic optimal bootstrap	elastic selected bootstrap
FRUITS TOTAL	-0,030	-0,039	-0,036	-0,037							-0,072	-0,073
APPLES	-0,087	-0,040	-0,033	-0,035					-0,061	-0,061	-0,045	-0,069
BANANAS	-0,055	0,046	0,052	0,043							0,061	0,082
GRAPES	-0,088	-0,059	-0,055	-0,055					-0,078	-0,078	-0,108	-0,108
ORANGES & MANDARINS	0,007	0,016	-0,008	0,018							-0,008	0,005
ALCOHOLIC BEVERAGES TOTAL	0,122	0,042	0,036	0,042					0,071	0,071	0,056	0,072
BEER	0,038	0,047	0,063	0,048	0,002				0,125	0,125	0,098	0,079
DISTILLED BEVERAGES	0,005	-0,018	-0,016	-0,018							-0,022	
WINE	-0,013	-0,023	-0,015	-0,020							-0,021	0,003
COCOA BEANS	0,085	0,010	-0,016	-0,004							-0,014	
COFFEE	0,042	-0,025	-0,041	-0,017							-0,018	0,005
TEA	0,149	0,073	0,076	0,070	0,120		0,101		0,207	0,207	0,134	0,135
REFINED SUGAR & SWEETENERS TOTAL	0,135	0,051	0,035	0,052	0,103		0,073		0,115	0,115	0,066	0,093
REFINED SUGAR	-0,081	0,048	0,063	0,042					0,032	0,032	0,057	0,081
OILCROPS TOTAL	-0,017	-0,028	-0,025	-0,025					0,081	0,081	-0,043	-0,040
OLIVES	-0,027	-0,031	-0,030	-0,025							-0,037	-0,047
TREENUTS	-0,073	-0,024	-0,016	-0,023							-0,032	-0,036
PLANT OILS TOTAL	0,097	0,023	0,018	0,025							0,061	0,025
OLIVE OIL	-0,038	-0,042	-0,039	-0,041					-0,052	-0,052	-0,058	-0,076
SOYBEAN OIL	0,115	0,034	0,020	0,035					0,038	0,038	0,083	0,056
SUNFLOWER OIL	-0,072	0,022	0,031	-0,013							-0,019	0,025
CEREALS TOTAL	-0,103	-0,034	-0,036	-0,032					-0,049	-0,049	-0,037	-0,059
MAIZE	-0,035	-0,039	-0,061	-0,036					-0,009	-0,009	-0,076	-0,071
RYE	-0,115	-0,049	-0,054	-0,048					-0,041	-0,041	-0,087	-0,080
WHEAT	0,077	0,047	0,046	0,043							0,089	0,083
POTATOES	-0,049	0,027	0,032	0,022							0,041	0,038
LEGUMES TOTAL	0,020	-0,034	-0,027	-0,026					-0,008	-0,008	-0,024	-0,049
VEGETABLES TOTAL	-0,025	-0,031	-0,035	-0,035							-0,073	-0,063
ONIONS	0,116	-0,046	-0,067	-0,044					-0,044	-0,044	-0,054	-0,084
TOMATOES	-0,021	-0,044	-0,050	-0,043							-0,069	-0,087
SPICES	0,056	0,039	0,033	0,033							0,057	0,057
PLANT PROTEIN	-0,007	-0,026	0,013	0,016							0,024	0,019
PLANT FAT	-0,013	-0,031	-0,040	-0,034							-0,042	-0,052
MEAT TOTAL	-0,121	-0,030	-0,027	-0,031							-0,058	-0,046
BEEF	-0,066	0,064	0,087	0,060					0,006	0,006	0,110	0,115
PORK	-0,015	-0,055	-0,076	-0,057					-0,111	-0,111	-0,132	-0,101
POULTRY	0,033	0,031	0,045	0,030					0,029	0,029	0,061	0,051
MEAT PROTEIN	-0,108	-0,026	-0,019	-0,026					-0,024	-0,024	-0,036	-0,038
MEAT FAT	0,020	0,023	0,025	0,024							0,020	0,025
BEEF & PORK FAT	-0,007	0,039	0,042	0,035							0,029	0,066
DAIRY TOTAL	0,022	0,033	0,021	0,035							0,093	0,053
MILK	0,033	0,038	0,058	0,040					0,071	0,071	0,072	0,069
CHEESE	-0,015	-0,030	-0,033	-0,030							-0,075	-0,060
DAIRY PROTEIN	0,090	0,031	0,036	0,032					0,044	0,044	0,040	0,050
DAIRY FAT	0,092	0,047	0,049	0,047					0,033	0,033	0,079	0,084
MILK PROTEIN	0,036	0,028	0,033	0,027							0,045	0,044
MILK FAT	0,047	0,034	0,044	0,034							0,060	0,057
BUTTER & GHEE	0,122	0,048	0,035	0,053					0,095	0,095	0,078	0,075
EDIBLE OFFALS	0,049	0,051	0,044	0,051					0,071	0,071	0,099	0,088
FISH & SEAFOOD	-0,095	-0,029	-0,012	-0,023							-0,040	-0,043
FISH & SEAFOOD FAT	0,032	0,046	0,061	0,044					0,030	0,030	0,092	0,075
EGGS TOTAL	0,058	-0,038	0,064	0,054					0,098	0,098	0,089	-0,061
LARD	0,013	0,032	0,038	0,025					0,028	0,028	0,051	0,052
HONEY	-0,065	-0,032	-0,028	-0,029							-0,056	-0,053
ANIMAL PROTEIN	0,193	0,032	0,005	0,034					0,005	0,005	-0,010	0,050
ANIMAL FAT	0,210	0,039	-0,025	0,042	0,010				0,071	0,071	0,051	0,054
ANIMAL FAT & ANIMAL PROTEIN	-0,128	0,047	0,088	0,041					0,047	0,047	0,058	0,084
TOTAL PROTEIN	0,118	0,053	0,040	0,051					0,053	0,053	0,083	0,089
TOTAL FAT	-0,076	-0,026	-0,029	-0,025							0,012	0,050
TOTAL FAT & TOTAL PROTEIN	0,094	0,012	0,001	0,009							-0,031	
% CA energy	0,101	-0,036	-0,078	-0,030							-0,067	-0,054
% PC CARB energy	-0,058	-0,022	0,003	-0,024							-0,080	-0,020
% Plant food energy	-0,073	-0,054	-0,052	-0,051	-0,370	-0,251	-0,358	-0,251	-0,151	-0,151	-0,045	-0,098
TOTAL ENERGY	0,164	0,043	0,021	0,041					0,013	0,013	0,081	0,067
VEGETABLES & CEREALS	-0,127	-0,032	-0,016	-0,037					-0,069	-0,069	-0,070	-0,054
MILK & VEGETABLES	-0,051	-0,018	-0,013	-0,021							-0,042	-0,021
MILK & VEG. & CEREALS	-0,010	-0,031	-0,035	-0,027					-0,001	-0,001	-0,064	-0,046
PROTEIN INDEX	0,057	0,015	0,012	0,013							0,023	0,020
Smoking - women (1990 - 2009)	-0,067	0,038	0,059	0,038							0,057	0,061
BMI - women (1990-2008)	0,027	0,019	0,015	0,015							0,037	0,020
Raised cholesterol - women (2008)	-0,062	0,067	0,085	0,062	0,154	0,069	0,159	0,069	0,170	0,170	0,078	0,122

Colorectal cancer - men

Ridge cross-validation

Ridge Models

Model	Penalty	Regularization "R Square" (1-Error)	Standardized Sum of Coefficients	Apparent Prediction Error	Expected Prediction Error		
					Estimate	Std. Error	N
26 (opt)	,500	,993	,006	,007	,207	,070	25
41 (pars)	,800	,984	,006	,016	,249	,082	25

Ridge bootstrap

Ridge Models

Model	Penalty	Regularization "R Square" (1-Error)	Standardized Sum of Coefficients	Apparent Prediction Error	Expected Prediction Error		
					Estimate	Std. Error	N
36 (opt)	,700	,988	,006	,012	,168	,075	28
44 (pars)	,860	,982	,005	,018	,220	,142	27

LASSO cross-validation

LASSO Models

Model	Penalty	Regularization "R Square" (1-Error)	Number of Selected Predictors	Standardized Sum of Coefficients	Apparent Prediction Error	Expected Prediction Error		
						Estimate	Std. Error	N
5 (opt)	,080	,986	18	,057	,014	1,051	,387	31
49 (pars)	,960	,574	3	,017	,426	1,419	,897	38

LASSO bootstrap

LASSO Models

Model	Penalty	Regularization "R Square" (1-Error)	Number of Selected Predictors	Standardized Sum of Coefficients	Apparent Prediction Error	Expected Prediction Error		
						Estimate	Std. Error	N
17 (opt)	,320	,902	5	,037	,098	,719	,474	39
51 (pars)	1,000	,547	3	,016	,453	1,088	,546	39

Elastic net cross-validation

Elastic Net Models

Model	Ridge Penalty	LASSO Penalty	Regularization "R Square" (1-Error)	Number of Selected Predictors	Standardized Sum of Coefficients	Apparent Prediction Error	Expected Prediction Error		
							Estimate	Std. Error	N
256 (opt & pars)	,500	,000	,824	71	1,000	,176	,420	,132	25

Elastic net bootstrap

Elastic Net Models

Model	Ridge Penalty	LASSO Penalty	Regularization "R Square" (1-Error)	Number of Selected Predictors	Standardized Sum of Coefficients	Apparent Prediction Error	Expected Prediction Error		
							Estimate	Std. Error	N
206 (opt)	0.4	,020	,853	71	3,520	,147	,367	,108	33
410 (pars)	0.8	,020	,613	68	,864	,387	,468	,161	31

Regression models with *beta* coefficients.

Model	26	41	36	41	5	49	17	51	256	256	206	410
**Colorectal cancer - men**	ridge optimal cross	ridge selected cross	ridge optimal bootstrap	ridge selected bootstrap	LASSO optimal cross	LASSO selected cross	LASSO optimal bootstrap	LASSO selected bootstrap	elastic optimal cross	elastic selected cross	elastic optimal bootstrap	elastic selected bootstrap
FRUITS TOTAL	-0,048	-0,048	-0,045	-0,048	-0,039				-0,072	-0,072	0,112	-0,088
APPLES	0,026	0,027	0,026	0,027					0,040	0,040	0,066	0,031
BANANAS	-0,033	-0,029	-0,031	-0,029					-0,049	-0,049	0,102	-0,042
GRAPES	-0,049	-0,048	-0,048	-0,048	-0,018				-0,073	-0,073	0,079	-0,086
ORANGES & MANDARINS	-0,021	-0,017	-0,019	-0,017					-0,031	-0,031	-0,083	-0,023
ALCOHOLIC BEVERAGES TOTAL	0,044	0,029	0,041	0,029					0,066	0,066	-0,130	0,059
BEER	0,013	0,016	0,014	0,016					0,019	0,019	0,157	0,024
DISTILLED BEVERAGES	-0,035	-0,029	-0,032	-0,029	-0,057				-0,052	-0,052	-0,091	-0,057
WINE	0,052	0,046	0,049	0,046	0,011				0,077	0,077	-0,055	0,077
COCOA BEANS	-0,008	-0,009	-0,007	-0,009					-0,011	-0,011	-0,120	0,003
COFFEE	-0,045	-0,043	-0,041	-0,043	-0,061				-0,067	-0,067	-0,346	-0,062
TEA	0,011	0,013	0,010	0,013					0,017	0,017	-0,056	0,008
REFINED SUGAR & SWEETENERS TOTAL	0,022	0,013	0,022	0,013					0,034	0,034	-0,039	0,028
REFINED SUGAR	0,043	0,053	0,043	0,053					0,065	0,065	0,151	0,068
OILCROPS TOTAL	-0,094	-0,091	-0,089	-0,091	-0,112				-0,141	-0,141	-0,269	-0,165
OLIVES	-0,090	-0,091	-0,086	-0,091	-0,383	-0,130	-0,434	-0,118	-0,134	-0,134	-0,144	-0,164
TREENUTS	-0,031	-0,030	-0,030	-0,030	-0,010				-0,046	-0,046	-0,082	-0,053
PLANT OILS TOTAL	-0,013	0,002	-0,013	0,002					-0,019	-0,019	-0,152	-0,023
OLIVE OIL	-0,024	-0,023	-0,024	-0,023					-0,036	-0,036	0,015	-0,032
SOYBEAN OIL	0,022	0,010	0,021	0,010					0,033	0,033	-0,390	0,037
SUNFLOWER OIL	0,053	0,052	0,050	0,052					0,079	0,079	0,351	0,084
CEREALS TOTAL	-0,006	-0,005	-0,007	-0,005					-0,010	-0,010	-0,125	0,006
MAIZE	0,026	0,021	0,023	0,021					0,039	0,039	-0,201	0,023
RYE	-0,021	-0,017	-0,018	-0,017					-0,031	-0,031	-0,087	-0,009
WHEAT	0,023	0,014	0,018	0,014					0,035	0,035	0,111	0,008
POTATOES	-0,019	-0,018	-0,017	-0,018					-0,029	-0,029	0,039	-0,011
LEGUMES TOTAL	-0,041	-0,036	-0,041	-0,036					-0,061	-0,061	-0,032	-0,067
VEGETABLES TOTAL	-0,035	-0,040	-0,035	-0,040	-0,009				-0,052	-0,052	-0,314	-0,063
ONIONS	-0,025	-0,027	-0,024	-0,027					-0,037	-0,037	-0,105	-0,028
TOMATOES	-0,024	-0,030	-0,025	-0,030					-0,036	-0,036	-0,209	0,007
SPICES	-0,040	-0,038	-0,037	-0,038	-0,017				-0,059	-0,059	0,036	-0,070
PLANT PROTEIN	-0,024	-0,027	-0,027	-0,027					-0,036	-0,036	-0,200	-0,048
PLANT FAT	0,017	0,013	0,017	0,013					0,025	0,025	-0,195	0,024
MEAT TOTAL	0,037	0,044	0,036	0,044					0,055	0,055	0,091	0,055
BEEF	-0,040	-0,022	-0,038	-0,022					-0,060	-0,060	0,086	-0,050
PORK	0,076	0,057	0,073	0,057	0,233	0,104	0,141	0,092	0,115	0,115	0,128	0,127
POULTRY	0,068	0,067	0,065	0,067	0,206		0,203		0,102	0,102	0,056	0,119
MEAT PROTEIN	0,023	0,033	0,022	0,033					0,034	0,034	0,290	0,036
MEAT FAT	0,018	0,012	0,019	0,012					0,027	0,027	0,033	0,021
BEEF & PORK FAT	0,030	0,028	0,030	0,028					0,045	0,045	0,015	0,042
DAIRY TOTAL	-0,016	-0,017	-0,015	-0,017					-0,024	-0,024	-0,263	
MILK	0,019	0,022	0,016	0,022	0,030				0,028	0,028	0,178	0,016
CHEESE	-0,052	-0,052	-0,050	-0,052	-0,037				-0,078	-0,078	0,017	-0,095
DAIRY PROTEIN	-0,028	-0,030	-0,027	-0,030					-0,043	-0,043	0,215	-0,041
DAIRY FAT	-0,066	-0,055	-0,062	-0,055					-0,099	-0,099	0,131	-0,106
MILK PROTEIN	0,014	-0,008	0,011	-0,008					0,020	0,020	-0,065	0,004
MILK FAT	-0,007	-0,010	-0,008	-0,010					-0,011	-0,011	-0,052	
BUTTER & GHEE	0,012	-0,007	0,013	-0,007					0,019	0,019	-0,229	0,013
EDIBLE OFFALS	0,032	0,029	0,030	0,029					0,048	0,048	-0,093	0,050
FISH & SEAFOOD	0,019	0,025	0,018	0,025					0,028	0,028	0,220	0,017
FISH & SEAFOOD FAT	0,017	0,023	0,017	0,023					0,026	0,026	0,171	0,016
EGGS TOTAL	0,081	0,084	0,078	0,084					0,122	0,122	0,165	0,140
LARD	0,066	0,069	0,065	0,069	0,032		0,005		0,098	0,098	0,231	0,121
HONEY	-0,040	-0,036	-0,039	-0,036					-0,060	-0,060	0,084	-0,066
ANIMAL PROTEIN	0,017	0,002	0,015	0,002					0,026	0,026	-0,292	0,021
ANIMAL FAT	0,020	-0,012	0,019	-0,012					0,030	0,030	-0,433	0,033
ANIMAL FAT & ANIMAL PROTEIN	0,018	0,037	0,018	0,037					0,027	0,027	0,225	0,030
TOTAL PROTEIN	-0,021	-0,023	-0,020	-0,023					-0,031	-0,031	0,157	-0,025
TOTAL FAT	0,018	0,023	0,019	0,023					0,028	0,028	0,099	0,026
TOTAL FAT & TOTAL PROTEIN	0,022	0,010	0,022	0,010					0,033	0,033	0,197	0,026
% CA energy	-0,012	-0,023	-0,011	-0,023					-0,018	-0,018	-0,293	-0,007
% PC CARB energy	0,023	0,021	0,020	0,021					0,034	0,034	0,211	0,014
% Plant food energy	-0,030	-0,021	-0,031	-0,021	-0,051				-0,045	-0,045	-0,143	-0,044
TOTAL ENERGY	-0,017	-0,029	-0,018	-0,029					-0,026	-0,026	-0,278	-0,022
VEGETABLES & CEREALS	-0,065	-0,051	-0,063	-0,051	-0,189		-0,200		-0,098	-0,098	0,426	-0,124
MILK & VEGETABLES	-0,040	-0,042	-0,041	-0,042					-0,060	-0,060	-0,315	-0,072
MILK & VEG. & CEREALS	-0,069	-0,069	-0,067	-0,069	-0,005	-0,210		-0,206	-0,103	-0,103	-0,380	-0,133
PROTEIN INDEX	0,009	-0,012	-0,007	-0,012					0,013	0,013	-0,360	
Smoking - men (1990-2009)	-0,070	-0,055	-0,065	-0,055					-0,104	-0,104	-0,042	-0,114
BMI – men (1990-2008)	0,016	0,012	0,015	0,012					0,023	0,023	0,133	0,016
Raised cholesterol - men (2008)	0,012	0,031	0,013	0,031					0,018	0,018	0,177	0,015

Colorectal cancer - women

Ridge cross-validation

Ridge Models

Model	Penalty	Regularization "R Square" (1-Error)	Standardized Sum of Coefficients	Apparent Prediction Error	Expected Prediction Error		
					Estimate	Std. Error	N
28 (opt)	,540	,991	,004	,009	,793	,489	26
51 (pars)	1,000	,977	,003	,023	,930	,639	26

Ridge bootstrap

Ridge Models

Model	Penalty	Regularization "R Square" (1-Error)	Standardized Sum of Coefficients	Apparent Prediction Error	Expected Prediction Error		
					Estimate	Std. Error	N
38 (opt)	,740	,984	,003	,016	,275	,147	29
51 (pars)	1,000	,977	,003	,023	,339	,185	30

LASSO cross-validation

LASSO Models

Model	Penalty	Regularization "R Square" (1-Error)	Number of Selected Predictors	Standardized Sum of Coefficients	Apparent Prediction Error	Expected Prediction Error		
						Estimate	Std. Error	N
5 (opt)	,080	,983	19	,040	,017	1,283	,442	32
51 (pars)	1,000	,556	3	,011	,444	1,515	,944	38

LASSO bootstrap

LASSO Models

Model	Penalty	Regularization "R Square" (1-Error)	Number of Selected Predictors	Standardized Sum of Coefficients	Apparent Prediction Error	Expected Prediction Error		
						Estimate		
19 (opt)	,360	,897	4	,026	,103	,720	,373	39
51 (pars)	1,000	,556	3	,011	,444	,939	,547	39

Elastic net cross-validation

Elastic Net Models

Model	Ridge Penalty	LASSO Penalty	Regularization "R Square" (1-Error)	Number of Selected Predictors	Standardized Sum of Coefficients	Apparent Prediction Error	Expected Prediction Error		
							Estimate	Std. Error	N
55 (opt)	0,1	,060	,975	57	,534	,025	,550	,210	31
210 (pars)	0,4	,100	,932	33	,299	,068	,552	,325	33

Elastic net bootstrap

Elastic Net Models

Model	Ridge Penalty	LASSO Penalty	Regularization "R Square" (1-Error)	Number of Selected Predictors	Standardized Sum of Coefficients	Apparent Prediction Error	Expected Prediction Error		
							Estimate	Std. Error	N
309 (opt)	0.6	,040	,797	56	,798	,203	,526	,261	35
560 (pars)	1,0	,980	,766	5	,160	,234	,691	,364	38

Regression models with *beta* coefficients.

Model	28	51	38	51	5	51	19	51	55	210	309	560
**Colorectal cancer - women**	ridge optimal cross	ridge selected cross	ridge optimal bootstrap	ridge selected bootstrap	LASSO optimal cross	LASSO selected cross	LASSO optimal bootstrap	LASSO selected bootstrap	elastic optimal cross	elastic selected cross	elastic optimal bootstrap	elastic selected bootstrap
FRUITS TOTAL	-0,054	-0,049	-0,053	-0,049	-0,023				-0,120	-0,063	-0,083	
APPLES	-0,009	-0,010	-0,005	-0,010					0,243		0,008	
BANANAS	0,023	0,018	0,007	0,018							0,001	
GRAPES	-0,042	-0,040	-0,045	-0,040					0,081		-0,060	
ORANGES & MANDARINS	0,010	0,008	0,018	0,008					0,122			
ALCOHOLIC BEVERAGES TOTAL	0,042	0,037	0,042	0,037					-0,137		0,051	
BEER	-0,010	0,010	-0,004	0,010					0,254			
DISTILLED BEVERAGES	-0,049	-0,043	-0,044	-0,043	-0,063					-0,049	-0,078	
WINE	0,051	0,046	0,057	0,046	0,005				0,168	0,047	0,065	
COCOA BEANS	0,022	0,018	0,021	0,018					-0,133		0,015	
COFFEE	-0,030	-0,024	-0,021	-0,024								
TEA	0,007	0,009	0,014	0,009					0,050			
REFINED SUGAR & SWEETENERS TOTAL	0,029	0,026	0,025	0,026	0,013				-0,014	0,012	0,043	
REFINED SUGAR	0,048	0,047	0,046	0,047					0,077	0,053	0,064	
OILCROPS TOTAL	-0,077	-0,069	-0,073	-0,069	-0,025				-0,125	-0,109	-0,122	
OLIVES	-0,096	-0,087	-0,093	-0,087	-0,434	-0,096	-0,402	-0,096	-0,022	-0,238	-0,183	-0,154
TREENUTS	-0,035	-0,033	-0,033	-0,033					0,049	-0,037	-0,052	
PLANT OILS TOTAL	0,004	0,003	0,003	0,003	-0,005					-0,009	-0,021	
OLIVE OIL	-0,024	-0,020	-0,020	-0,020						-0,004	-0,019	
SOYBEAN OIL	0,042	0,037	0,043	0,037					-0,003	0,009	0,048	
SUNFLOWER OIL	0,053	0,047	0,051	0,047	0,014				0,374	0,033	0,071	
CEREALS TOTAL	-0,019	-0,016	-0,012	-0,016					0,017			
MAIZE	0,042	0,031	0,033	0,031					-0,056		0,040	
RYE	-0,021	-0,019	-0,017	-0,019							-0,014	
WHEAT	0,012	-0,010	-0,015	-0,010					-0,019			
POTATOES	-0,022	-0,022	-0,018	-0,022					0,069		-0,011	
LEGUMES TOTAL	-0,040	-0,035	-0,033	-0,035					-0,011	-0,038	-0,060	
VEGETABLES TOTAL	-0,041	-0,036	-0,039	-0,036	-0,015				-0,155	-0,043	-0,060	
ONIONS	-0,027	-0,028	-0,018	-0,028					-0,117		-0,015	
TOMATOES	-0,032	-0,028	-0,030	-0,028					0,007		0,012	
SPICES	-0,044	-0,039	-0,045	-0,039	-0,012				-0,081	-0,048	-0,065	
PLANT PROTEIN	-0,025	-0,027	-0,031	-0,027					-0,283	-0,018	-0,035	
PLANT FAT	0,021	0,023	0,024	0,023					-0,270		0,021	
MEAT TOTAL	0,027	0,027	0,020	0,027					0,028		0,011	
BEEF	-0,040	-0,033	-0,037	-0,033					0,110			
PORK	0,082	0,072	0,074	0,072	0,247	0,067	0,130	0,067		0,159	0,156	0,098
POULTRY	0,066	0,060	0,056	0,060	0,194		0,182		0,047	0,128	0,113	
MEAT PROTEIN	0,009	0,010	0,015	0,010					0,261			
MEAT FAT	0,023	0,018	0,011	0,018							0,015	
BEEF & PORK FAT	0,027	0,023	0,015	0,023							0,032	
DAIRY TOTAL	-0,025	-0,022	-0,017	-0,022					0,114			
MILK	0,025	0,015	0,020	0,015	0,014						0,002	
CHEESE	-0,062	-0,054	-0,063	-0,054	-0,056				-0,303	-0,086	-0,102	
DAIRY PROTEIN	-0,026	-0,026	-0,030	-0,026					-0,006		-0,011	
DAIRY FAT	-0,075	-0,066	-0,073	-0,066					0,139	-0,085	-0,120	
MILK PROTEIN	-0,007	-0,010	-0,011	-0,010	0,049				-0,003			
MILK FAT	-0,013	-0,014	-0,016	-0,014							0,005	
BUTTER & GHEE	-0,009	-0,008	-0,003	-0,008					0,017			
EDIBLE OFFALS	0,026	0,023	0,027	0,023					0,013		0,021	
FISH & SEAFOOD	0,014	0,015	0,017	0,015					0,161			
FISH & SEAFOOD FAT	0,018	0,019	0,025	0,019					0,197		0,009	
EGGS TOTAL	0,082	0,075	0,075	0,075					0,061	0,152	0,136	0,093
LARD	0,067	0,064	0,067	0,064	0,014				0,192	0,086	0,098	
HONEY	-0,039	-0,038	-0,041	-0,038					-0,012	-0,034	-0,058	
ANIMAL PROTEIN	0,012	0,009	0,010	0,009					-0,400		0,009	
ANIMAL FAT	-0,003	0,021	-0,001	0,021					-0,499	0,015	0,032	
ANIMAL FAT & ANIMAL PROTEIN	-0,006	0,007	0,006	0,007					0,204			
TOTAL PROTEIN	0,014	0,008	0,005	0,008							-0,016	
TOTAL FAT	0,026	-0,014	0,014	-0,014					0,097	0,001	0,020	
TOTAL FAT & TOTAL PROTEIN	0,031	0,025	0,023	0,025					0,037	0,001	0,039	
% CA energy	-0,005	-0,007	-0,005	-0,007					-0,133			
% PC CARB energy	-0,018	-0,018	-0,034	-0,018					-0,126	-0,012	-0,021	
% Plant food energy	-0,031	-0,026	-0,018	-0,026						-0,023	-0,043	
TOTAL ENERGY	-0,029	-0,025	-0,021	-0,025					-0,176		-0,022	
VEGETABLES & CEREALS	-0,065	-0,061	-0,065	-0,061	-0,175				0,235	-0,141	-0,132	-0,159
MILK & VEGETABLES	-0,041	-0,042	-0,045	-0,042					-0,285	-0,026	-0,057	
MILK & VEG. & CEREALS	-0,067	-0,065	-0,072	-0,065	-0,084	-0,254	-0,227	-0,254	-0,207	-0,138	-0,121	-0,178
PROTEIN INDEX	0,011	0,010	0,014	0,010					-0,256			
Smoking - women (1990 - 2009)	-0,039	-0,036	-0,044	-0,036	-0,016					-0,033	-0,061	
BMI - women (1990-2008)	0,032	0,031	0,034	0,031							0,040	
Raised cholesterol - women (2008)	0,034	0,031	0,020	0,031					0,071	0,026	0,032	

Gallbladder cancer - men

Ridge cross-validation

Ridge Models

Model	Penalty	Regularization "R Square" (1-Error)	Standardized Sum of Coefficients	Apparent Prediction Error	Expected Prediction Error		
					Estimate	Std. Error	N
48 (opt)	,940	,919	,017	,081	1,260	,526	25
51 (pars)	1,000	,913	,016	,087	1,336	,630	25

Ridge bootstrap

Ridge Models

Model	Penalty	Regularization "R Square" (1-Error)	Standardized Sum of Coefficients	Apparent Prediction Error	Expected Prediction Error		
					Estimate	Std. Error	N
48 (opt)	,940	,919	,017	,081	,678	,291	28
51 (pars)	1,000	,913	,016	,087	,848	,476	28

LASSO cross-validation

LASSO Models

Model	Penalty	Regularization "R Square" (1-Error)	Number of Selected Predictors	Standardized Sum of Coefficients	Apparent Prediction Error	Expected Prediction Error		
						Estimate	Std. Error	N
47 (opt)	,920	,117	2	,005	,883	1,131	,562	39
51 (pars)	1,000	,071	1	,003	,929	1,433	,623	39

LASSO bootstrap

LASSO Models

Model	Penalty	Regularization "R Square" (1-Error)	Number of Selected Predictors	Standardized Sum of Coefficients	Apparent Prediction Error	Expected Prediction Error		
						Estimate	Std. Error	N
15 (opt)	,280	,703	11	,061	,297	1,248	,380	39
51 (pars)	1,000	,071	1	,003	,929	1,277	,452	39

Elastic net cross-validation

Elastic Net Models

Model	Ridge Penalty	LASSO Penalty	Regularization "R Square" (1-Error)	Number of Selected Predictors	Standardized Sum of Coefficients	Apparent Prediction Error	Expected Prediction Error		
							Estimate	Std. Error	N
47 (opt)	0,0	,920	,117	2	,005	,883	1,131	,562	39
561 (pars)	1,0	1,000	,071	1	,011	,929	1,519	,658	39

Elastic net bootstrap

Elastic Net Models

Model	Ridge Penalty	LASSO Penalty	Regularization "R Square" (1-Error)	Number of Selected Predictors	Standardized Sum of Coefficients	Apparent Prediction Error	Expected Prediction Error		
							Estimate	Std. Error	N
155 (opt)	0,3	,020	,951	69	1,757	,049	,939	,396	29
559 (pars)	1,0	,960	,098	2	,015	,902	1,307	,469	39

Regression models with *beta* coefficients.

Model	48	51	48	51	47	51	15	51	47	561	155	559
**Gallbladder cancer - men**	ridge optimal cross	ridge selected cross	ridge optimal bootstrap	ridge selected bootstrap	LASSO optimal cross	LASSO selected cross	LASSO optimal bootstrap	LASSO selected bootstrap	elastic optimal cross	elastic selected cross	elastic optimal bootstrap	elastic selected bootstrap
FRUITS TOTAL	-0,020	-0,019	-0,020	-0,019							0,192	
APPLES	0,043	0,042	0,043	0,042							-0,034	
BANANAS	0,052	0,053	0,052	0,053							0,199	
GRAPES	0,053	0,052	0,053	0,052							0,150	
ORANGES & MANDARINS	-0,042	-0,039	-0,042	-0,039							-0,186	
ALCOHOLIC BEVERAGES TOTAL	0,063	0,063	0,063	0,063							0,124	
BEER	0,078	0,077	0,078	0,077			0,113					
DISTILLED BEVERAGES	0,035	0,034	0,035	0,034							0,085	
WINE	0,060	0,060	0,060	0,060							-0,093	
COCOA BEANS	-0,032	-0,032	-0,032	-0,032							-0,113	
COFFEE	-0,041	-0,039	-0,041	-0,039							-0,313	
TEA	-0,071	-0,069	-0,071	-0,069			-0,075				-0,186	
REFINED SUGAR & SWEETENERS TOTAL	-0,090	-0,088	-0,090	-0,088							0,030	
REFINED SUGAR	-0,067	-0,066	-0,067	-0,066							0,129	
OILCROPS TOTAL	-0,044	-0,046	-0,044	-0,046							-0,210	
OLIVES	-0,029	-0,030	-0,029	-0,030							-0,090	
TREENUTS	0,020	0,019	0,020	0,019							0,053	
PLANT OILS TOTAL	-0,024	-0,023	-0,024	-0,023							-0,220	
OLIVE OIL	0,019	0,017	0,019	0,017							-0,018	
SOYBEAN OIL	0,062	0,063	0,062	0,063			0,081					
SUNFLOWER OIL	0,034	0,034	0,034	0,034							-0,003	
CEREALS TOTAL	0,029	0,029	0,029	0,029							-0,185	
MAIZE	-0,012	-0,012	-0,012	-0,012							0,106	
RYE	-0,039	-0,039	-0,039	-0,039							-0,040	
WHEAT	0,049	0,048	0,049	0,048			0,021				0,263	
POTATOES	0,044	0,040	0,044	0,040							0,037	
LEGUMES TOTAL	0,064	0,063	0,064	0,063							0,099	
VEGETABLES TOTAL	-0,097	-0,096	-0,097	-0,096			-0,095				-0,208	
ONIONS	-0,025	0,011	-0,025	0,011							-0,165	
TOMATOES	-0,019	-0,020	-0,019	-0,020							-0,102	
SPICES	0,052	0,053	0,052	0,053							0,163	
PLANT PROTEIN	-0,042	-0,043	-0,042	-0,043							0,128	
PLANT FAT	-0,029	-0,028	-0,029	-0,028							-0,176	
MEAT TOTAL	-0,037	-0,039	-0,037	-0,039							0,131	
BEEF	-0,054	-0,054	-0,054	-0,054							0,046	
PORK	0,073	0,071	0,073	0,071							0,271	
POULTRY	0,019	0,018	0,019	0,018							0,008	
MEAT PROTEIN	0,033	0,032	0,033	0,032							0,163	
MEAT FAT	-0,026	-0,026	-0,026	-0,026							0,056	
BEEF & PORK FAT	-0,037	-0,037	-0,037	-0,037							-0,024	
DAIRY TOTAL	0,044	0,043	0,044	0,043							-0,322	
MILK	-0,088	-0,086	-0,088	-0,086			-0,013				-0,033	
CHEESE	0,065	0,065	0,065	0,065							0,271	
DAIRY PROTEIN	-0,055	-0,053	-0,055	-0,053			-0,136				0,172	
DAIRY FAT	0,012	0,010	0,012	0,010							0,047	
MILK PROTEIN	-0,069	-0,067	-0,069	-0,067							-0,053	
MILK FAT	-0,056	-0,056	-0,056	-0,056			-0,021				0,027	
BUTTER & GHEE	0,009	0,010	0,009	0,010							-0,320	
EDIBLE OFFALS	0,011	0,011	0,011	0,011							-0,111	
FISH & SEAFOOD	0,036	0,034	0,036	0,034							0,188	
FISH & SEAFOOD FAT	0,033	0,032	0,033	0,032							-0,030	
EGGS TOTAL	0,083	0,081	0,083	0,081							0,188	
LARD	0,042	0,042	0,042	0,042							0,135	
HONEY	0,031	0,030	0,031	0,030							0,127	
ANIMAL PROTEIN	0,017	0,016	0,017	0,016							-0,045	
ANIMAL FAT	-0,054	-0,051	-0,054	-0,051							-0,321	
ANIMAL FAT & ANIMAL PROTEIN	0,033	0,032	0,033	0,032							0,140	
TOTAL PROTEIN	-0,063	-0,062	-0,063	-0,062							0,123	
TOTAL FAT	-0,023	-0,022	-0,023	-0,022							0,101	
TOTAL FAT & TOTAL PROTEIN	-0,035	-0,035	-0,035	-0,035							0,105	
% CA energy	0,038	0,036	0,038	0,036							-0,143	
% PC CARB energy	-0,035	-0,034	-0,035	-0,034							0,412	
% Plant food energy	0,016	0,016	0,016	0,016							-0,095	
TOTAL ENERGY	-0,049	-0,048	-0,049	-0,048							-0,204	
VEGETABLES & CEREALS	-0,027	-0,028	-0,027	-0,028							0,331	
MILK & VEGETABLES	-0,077	-0,076	-0,077	-0,076	-0,012		-0,243		-0,012		-0,115	-0,010
MILK & VEG. & CEREALS	-0,051	-0,052	-0,051	-0,052							-0,201	
PROTEIN INDEX	-0,033	-0,032	-0,033	-0,032							-0,113	
Smoking - men (1990-2009)	0,036	0,036	0,036	0,036							0,054	
BMI – men (1990-2008)	0,096	0,095	0,096	0,095	0,103	0,066	0,286	0,066	0,103	0,066	0,176	0,085
Raised cholesterol - men (2008)	-0,093	-0,089	-0,093	-0,089			-0,224				-0,089	

Gallbladder cancer – women

Ridge cross-validation

Ridge Models

Model	Penalty	Regularization "R Square" (1-Error)	Standardized Sum of Coefficients	Apparent Prediction Error	Expected Prediction Error		
					Estimate	Std. Error	N
14 (opt)	,260	,983	,031	,017	1,262	,476	26
51 (pars)	1,000	,911	,014	,089	1,399	,571	26

Ridge bootstrap

Ridge Models

Model	Penalty	Regularization "R Square" (1-Error)	Standardized Sum of Coefficients	Apparent Prediction Error	Expected Prediction Error		
					Estimate	Std. Error	N
41 (opt)	,800	,929	,016	,071	,661	,239	30
51 (pars)	1,000	,911	,014	,089	,840	,340	30

LASSO cross-validation

LASSO Models

Model	Penalty	Regularization "R Square" (1-Error)	Number of Selected Predictors	Standardized Sum of Coefficients	Apparent Prediction Error	Expected Prediction Error		
						Estimate	Std. Error	N
49 (opt)	,960	,093	1	,004	,907	1,041	,379	39
50 (pars)	,980	,083	1	,003	,917	1,301	,406	39

LASSO bootstrap

LASSO Models

Model	Penalty	Regularization "R Square" (1-Error)	Number of Selected Predictors	Standardized Sum of Coefficients	Apparent Prediction Error	Expected Prediction Error		
						Estimate	Std. Error	N
39 (opt)	,760	,232	4	,010	,768	1,087	,302	39
51 (pars)	1,000	,073	2	,003	,927	1,260	,352	39

Elastic net cross-validation

Elastic Net Models

Model	Ridge Penalty	LASSO Penalty	Regularization "R Square" (1-Error)	Number of Selected Predictors	Standardized Sum of Coefficients	Apparent Prediction Error	Expected Prediction Error		
							Estimate	Std. Error	N
357 (opt)	0.6	1,000	,088	2	,015	,912	,957	,379	39
561 (pars)	1,0	1,000	,095	2	,015	,905	,964	,379	39

Elastic net bootstrap

Elastic Net Models

Model	Ridge Penalty	LASSO Penalty	Regularization "R Square" (1-Error)	Number of Selected Predictors	Standardized Sum of Coefficients	Apparent Prediction Error	Expected Prediction Error		
							Estimate	Std. Error	N
206 (opt)	0.4	,020	,945	66	,877	,055	,747	,320	32
362 (pars)	0.7	,080	,887	52	,666	,113	1,013	,422	34

Regression models with *beta* coefficients.

Model	14	51	41	51	49	50	39	51	357	561	206	362
**Gallbladder cancer - women**	ridge optimal cross	ridge selected cross	ridge optimal bootstrap	ridge selected bootstrap	LASSO optimal cross	LASSO selected cross	LASSO optimal bootstrap	LASSO selected bootstrap	elastic optimal cross	elastic selected cross	elastic optimal bootstrap	elastic selected bootstrap
FRUITS TOTAL	-0,061	-0,031	-0,031	-0,031								-0,054
APPLES	-0,083	-0,066	-0,082	-0,066							-0,135	
BANANAS	-0,057	0,030	0,048	0,030							0,027	
GRAPES	0,055	0,032	0,041	0,032							0,070	0,056
ORANGES & MANDARINS	-0,057	-0,066	-0,081	-0,066							-0,105	-0,136
ALCOHOLIC BEVERAGES TOTAL	0,108	0,079	0,081	0,079							0,109	0,107
BEER	0,135	0,097	0,097	0,097			0,031				0,163	0,172
DISTILLED BEVERAGES	0,013	-0,045	-0,051	-0,045							-0,079	-0,047
WINE	0,060	0,016	0,008	0,016							-0,006	-0,019
COCOA BEANS	-0,060	-0,055	-0,062	-0,055							-0,076	0,097
COFFEE	0,055	0,036	0,030	0,036							0,038	0,021
TEA	-0,046	-0,046	-0,063	-0,046							-0,076	-0,040
REFINED SUGAR & SWEETENERS TOTAL	-0,118	-0,088	-0,096	-0,088							-0,156	-0,169
REFINED SUGAR	-0,102	-0,064	-0,060	-0,064							-0,097	-0,103
OILCROPS TOTAL	-0,084	-0,067	-0,072	-0,067							-0,101	-0,030
OLIVES	-0,071	-0,055	-0,053	-0,055							-0,053	-0,063
TREENUTS	0,047	0,017	0,018	0,017							0,020	0,008
PLANT OILS TOTAL	0,058	0,035	0,033	0,035							0,021	0,019
OLIVE OIL	0,068	0,039	0,039	0,039							0,037	
SOYBEAN OIL	0,054	0,028	0,019	0,028							0,013	0,025
SUNFLOWER OIL	-0,067	-0,036	-0,039	-0,036							-0,074	-0,034
CEREALS TOTAL	0,064	0,019	0,011	0,019							0,017	0,003
MAIZE	0,023	-0,002	-0,003	-0,002							-0,010	
RYE	0,055	0,041	0,044	0,041							0,085	0,066
WHEAT	0,065	0,046	0,043	0,046							0,048	0,044
POTATOES	0,032	-0,035	0,014	-0,035								
LEGUMES TOTAL	0,081	0,044	0,050	0,044							0,077	0,076
VEGETABLES TOTAL	-0,152	-0,082	-0,092	-0,082							-0,146	-0,137
ONIONS	0,042	0,032	0,026	0,032							0,003	
TOMATOES	-0,071	-0,065	-0,068	-0,065							-0,102	-0,090
SPICES	-0,031	0,038	0,041	0,038							0,026	
PLANT PROTEIN	-0,065	-0,026	-0,034	-0,026							-0,048	
PLANT FAT	-0,013	-0,028	-0,039	-0,028							0,058	0,051
MEAT TOTAL	0,004	-0,008	-0,010	-0,008							0,064	0,033
BEEF	0,032	-0,071	-0,073	-0,071							-0,132	-0,144
PORK	0,109	0,104	0,117	0,104			0,098				0,178	0,206
POULTRY	0,013	-0,011	0,017	-0,011								
MEAT PROTEIN	0,083	0,043	0,049	0,043							0,081	0,039
MEAT FAT	-0,061	-0,012	-0,009	-0,012							0,014	
BEEF & PORK FAT	-0,071	-0,025	-0,027	-0,025							0,047	
DAIRY TOTAL	0,097	0,041	0,033	0,041							0,081	0,056
MILK	-0,115	-0,077	-0,082	-0,077							-0,141	-0,141
CHEESE	-0,009	0,058	0,063	0,058							0,092	0,096
DAIRY PROTEIN	-0,108	-0,053	-0,053	-0,053							-0,088	-0,068
DAIRY FAT	0,038	0,037	0,043	0,037							0,073	0,059
MILK PROTEIN	-0,110	-0,083	-0,087	-0,083			-0,023	-0,038	-0,044	-0,047	-0,140	-0,138
MILK FAT	0,044	0,017	0,016	0,017	-0,088	-0,078	-0,101	-0,030	-0,040	-0,044	0,033	-0,201
BUTTER & GHEE	0,098	0,030	0,025	0,030							0,031	
EDIBLE OFFALS	-0,006	-0,022	-0,024	-0,022							0,020	
FISH & SEAFOOD	-0,100	-0,087	-0,090	-0,087							-0,137	
FISH & SEAFOOD FAT	-0,068	-0,070	-0,078	-0,070							-0,108	
EGGS TOTAL	-0,036	0,017	0,026	0,017							0,008	0,005
LARD	0,028	0,022	0,021	0,022							0,029	
HONEY	-0,120	-0,062	-0,069	-0,062							0,021	0,046
ANIMAL PROTEIN	-0,015	0,024	0,020	0,024							0,008	
ANIMAL FAT	0,058	0,013	0,004	0,013								
ANIMAL FAT & ANIMAL PROTEIN	0,029	0,031	0,044	0,031							-0,064	0,031
TOTAL PROTEIN	-0,090	-0,041	-0,042	-0,041							-0,070	-0,060
TOTAL FAT	-0,057	-0,020	-0,012	-0,020							-0,012	
TOTAL FAT & TOTAL PROTEIN	-0,072	-0,016	-0,007	-0,016								-0,024
% CA energy	-0,036	0,030	0,028	0,030							0,028	0,029
% PC CARB energy	-0,092	-0,023	-0,012	-0,023							0,054	0,018
% Plant food energy	0,090	0,022	0,017	0,022							0,027	0,024
TOTAL ENERGY	-0,072	-0,047	-0,054	-0,047							-0,096	-0,068
VEGETABLES & CEREALS	-0,107	-0,043	-0,035	-0,043							-0,037	-0,019
MILK & VEGETABLES	-0,112	-0,078	-0,082	-0,078							-0,148	-0,145
MILK & VEG. & CEREALS	-0,086	-0,039	-0,042	-0,039							-0,062	-0,042
PROTEIN INDEX	-0,078	-0,064	-0,076	-0,064							-0,127	-0,107
Smoking - women (1990 - 2009)	0,032	0,056	0,063	0,056							0,107	0,080
BMI - women (1990-2008)	-0,031	-0,030	-0,035	-0,030							0,099	0,110
Raised cholesterol - women (2008)	-0,102	-0,051	-0,046	-0,051							-0,079	-0,133

Bladder cancer - men

Ridge cross-validation

Ridge Models

Model	Penalty	Regularization "R Square" (1-Error)	Standardized Sum of Coefficients	Apparent Prediction Error	Expected Prediction Error		
					Estimate	Std. Error	N
43 (opt)	,840	,945	,010	,055	,598	,131	24
51 (pars)	1,000	,934	,009	,066	,687	,162	24

Ridge bootstrap

Ridge Models

Model	Penalty	Regularization "R Square" (1-Error)	Standardized Sum of Coefficients	Apparent Prediction Error	Expected Prediction Error		
					Estimate	Std. Error	N
41 (opt)	,800	,949	,010	,051	,494	,145	27
42 (pars)	,820	,950	,009	,050	,535	,178	27

LASSO cross-validation

LASSO Models

Model	Penalty	Regularization "R Square" (1-Error)	Number of Selected Predictors	Standardized Sum of Coefficients	Apparent Prediction Error	Expected Prediction Error		
						Estimate	Std. Error	N
4 (opt)	,060	,981	35	,134	,019	,725	,186	27
8 (pars)	,140	,922	18	,073	,078	,887	,287	33

LASSO bootstrap

LASSO Models

Model	Penalty	Regularization "R Square" (1-Error)	Number of Selected Predictors	Standardized Sum of Coefficients	Apparent Prediction Error	Expected Prediction Error		
						Estimate	Std. Error	N
37 (opt)	,720	,374	4	,015	,626	,837	,243	38
50 (pars)	,980	,187	1	,006	,813	,855	,173	38

Elastic net cross-validation

Elastic Net Models

Model	Ridge Penalty	LASSO Penalty	Regularization "R Square" (1-Error)	Number of Selected Predictors	Standardized Sum of Coefficients	Apparent Prediction Error	Expected Prediction Error		
							Estimate	Std. Error	N
4 (opt)	0.0	,060	,981	35	,134	,019	,725	,186	27
504 (pars)	0.9	,880	,294	4	,053	,706	,728	,153	38

Elastic net bootstrap

Elastic Net Models

Model	Ridge Penalty	LASSO Penalty	Regularization "R Square" (1-Error)	Number of Selected Predictors	Standardized Sum of Coefficients	Apparent Prediction Error	Expected Prediction Error		
							Estimate	Std. Error	N
156 (opt)	0,3	,040	,961	53	,474	,039	,678	,196	32
560 (pars)	1,0	,980	,130	1	,021	,870	,848	,181	38

Regression models with *beta* coefficients.

Model	47	51	41	42	4	8	37	51	4	504	156	560
**Bladder cancer - men**	ridge optimal cross	ridge selected cross	ridge optimal bootstrap	ridge selected bootstrap	LASSO optimal cross	LASSO selected cross	LASSO optimal bootstrap	LASSO selected bootstrap	elastic optimal cross	elastic selected cross	elastic optimal bootstrap	elastic selected bootstrap
FRUITS TOTAL	-0,032	-0,031	-0,032	-0,033							0,062	
APPLES	0,041	0,040	0,041	0,044	0,028	0,007			0,028		0,023	
BANANAS	0,030	0,033	0,035	0,032							0,082	
GRAPES	0,063	0,063	0,070	0,067	0,103				0,103		0,033	
ORANGES & MANDARINS	-0,038	-0,039	-0,041	-0,040							-0,114	
ALCOHOLIC BEVERAGES TOTAL	-0,027	-0,027	-0,026	-0,028								
BEER	0,027	0,026	0,027	0,028								
DISTILLED BEVERAGES	-0,061	-0,059	-0,064	-0,058		-0,049					-0,093	
WINE	0,021	0,019	0,020	0,018							-0,075	
COCOA BEANS	0,059	0,057	0,064	0,061	0,052	0,146			0,052		0,143	
COFFEE	0,068	0,064	0,066	0,064	0,002				0,002		0,028	
TEA	-0,116	-0,113	-0,123	-0,116	-0,348	-0,341			-0,348		-0,293	-0,119
REFINED SUGAR & SWEETENERS TOTAL	0,046	0,044	0,047	0,046							0,105	
REFINED SUGAR	0,068	0,067	0,067	0,070	0,213	0,177			0,213		0,093	
OILCROPS TOTAL	0,040	0,039	0,042	0,037							0,008	
OLIVES	-0,005	-0,005	-0,005	0,084							0,104	
TREENUTS	0,070	0,067	0,070	0,070	0,135				0,135		0,053	
PLANT OILS TOTAL	-0,010	0,022	0,022	-0,015								
OLIVE OIL	0,023	0,020	0,021	0,027								
SOYBEAN OIL	-0,022	-0,023	-0,023	-0,021		0,044					0,049	
SUNFLOWER OIL	0,032	0,032	0,036	0,033	0,079				0,079		0,009	
CEREALS TOTAL	0,025	0,022	0,022	0,021	0,034				0,034			
MAIZE	-0,039	-0,039	-0,038	-0,038		-0,043					-0,001	
RYE	-0,008	-0,007	0,023	0,025							0,023	
WHEAT	-0,023	-0,021	-0,024	-0,024							-0,001	
POTATOES	-0,103	-0,100	-0,110	-0,104	-0,174	-0,235	-0,109		-0,174	-0,069	-0,196	
LEGUMES TOTAL	0,012	0,010	0,007	0,015								
VEGETABLES TOTAL	0,053	0,051	0,055	-0,023							0,073	
ONIONS	-0,040	-0,040	-0,045	-0,043	0,051	0,019			0,051		0,020	
TOMATOES	0,083	0,079	0,086	0,082	0,047	0,097			0,047		0,135	
SPICES	-0,015	-0,013	-0,014	0,004								
PLANT PROTEIN	-0,032	-0,033	-0,035	-0,031	-0,012				-0,012		0,024	
PLANT FAT	0,036	0,032	0,034	0,036	-0,005				-0,005			
MEAT TOTAL	0,015	0,017	0,018	0,017							0,009	
BEEF	-0,073	-0,069	-0,077	-0,071							-0,134	
PORK	0,044	0,043	0,044	0,043							0,114	
POULTRY	-0,025	-0,024	-0,027	-0,028							0,030	
MEAT PROTEIN	0,006	-0,012	-0,015	0,008							-0,029	
MEAT FAT	-0,067	-0,063	-0,067	-0,066	-0,285				-0,285		-0,068	
BEEF & PORK FAT	-0,084	-0,079	-0,085	-0,083	-0,069	-0,208			-0,069		-0,130	
DAIRY TOTAL	-0,024	-0,025	-0,030	-0,027	-0,073				-0,073		-0,067	
MILK	-0,080	-0,078	-0,078	-0,078		-0,151	-0,254	-0,153		-0,192	-0,116	
CHEESE	-0,024	-0,025	-0,027	-0,025	-0,072				-0,072		0,086	
DAIRY PROTEIN	-0,034	-0,033	-0,034	-0,038	-0,009				-0,009		-0,006	
DAIRY FAT	-0,018	-0,018	-0,016	-0,016	-0,004				-0,004		0,008	
MILK PROTEIN	-0,047	-0,047	-0,046	-0,047						-0,014	-0,039	
MILK FAT	-0,045	-0,045	-0,044	-0,043							-0,014	
BUTTER & GHEE	-0,047	-0,048	-0,053	-0,048	-0,085				-0,085		0,003	
EDIBLE OFFALS	0,026	0,025	0,027	0,027								
FISH & SEAFOOD	0,041	0,042	0,042	0,042							0,048	
FISH & SEAFOOD FAT	0,049	0,047	0,047	0,046	0,032				0,032		0,039	
EGGS TOTAL	0,035	0,034	0,034	0,034							0,039	
LARD	0,066	0,066	0,068	0,073	0,065	0,138			0,065		0,122	
HONEY	-0,063	-0,062	-0,063	-0,065							-0,082	
ANIMAL PROTEIN	0,017	0,015	0,013	0,014	-0,076				-0,076		0,027	
ANIMAL FAT	0,046	0,043	0,041	0,042							0,057	
ANIMAL FAT & ANIMAL PROTEIN	-0,011	-0,007	0,018	-0,009	0,288				0,288			
TOTAL PROTEIN	0,022	0,021	0,022	0,023							0,025	
TOTAL FAT	-0,024	-0,021	-0,022	-0,023							-0,002	
TOTAL FAT & TOTAL PROTEIN	-0,018	-0,019	-0,020	-0,021	-0,014	0,028			-0,014		0,117	
% CA energy	-0,062	-0,065	-0,069	-0,064	-0,161		-0,003		-0,161	-0,011		
% PC CARB energy	-0,047	-0,045	-0,043	-0,043	-0,007	-0,125	-0,021		-0,007			
% Plant food energy	0,037	0,034	0,035	0,036	0,059				0,059			
TOTAL ENERGY	0,014	0,012	0,011	0,011	-0,022				-0,022			
VEGETABLES & CEREALS	-0,012	-0,013	-0,013	-0,013	0,053				0,053			
MILK & VEGETABLES	-0,042	-0,042	-0,041	-0,039	-0,054				-0,054			
MILK & VEG. & CEREALS	-0,029	-0,032	-0,031	-0,030	-0,082				-0,082			
PROTEIN INDEX	-0,038	-0,038	-0,040	-0,042	-0,257				-0,257		-0,045	
Smoking - men (1990-2009)	-0,059	-0,057	-0,060	-0,059		-0,043					-0,087	
BMI – men (1990-2008)	-0,011	-0,010	-0,012	-0,014		0,032						
Raised cholesterol - men (2008)	0,051	0,055	0,058	0,054	0,499	0,054			0,499		0,078	

Bladder cancer - women

Ridge cross-validation

Ridge Models

Model	Penalty	Regularization "R Square" (1-Error)	Standardized Sum of Coefficients	Apparent Prediction Error	Expected Prediction Error		
					Estimate	Std. Error	N
48 (opt)	,940	,936	,005	,064	,929	,355	26
51 (pars)	1,000	,932	,005	,068	,943	,350	26

Ridge bootstrap

Ridge Models

Model	Penalty	Regularization "R Square" (1-Error)	Standardized Sum of Coefficients	Apparent Prediction Error	Expected Prediction Error		
					Estimate	Std. Error	N
32 (opt)	,620	,965	,006	,035	,384	,141	30
51 (pars)	1,000	,932	,005	,068	,385	,137	30

LASSO cross-validation

LASSO Models

Model	Penalty	Regularization "R Square" (1-Error)	Number of Selected Predictors	Standardized Sum of Coefficients	Apparent Prediction Error	Expected Prediction Error		
						Estimate	Std. Error	N
9 (opt)	,160	,913	19	,046	,087	,601	,225	36
10 (pars)	,180	,913	8	,034	,087	,779	,416	35

LASSO bootstrap

LASSO Models

Model	Penalty	Regularization "R Square" (1-Error)	Number of Selected Predictors	Standardized Sum of Coefficients	Apparent Prediction Error	Expected Prediction Error		
						Estimate	Std. Error	N
11 (opt)	,200	,903	8	,032	,097	,619	,212	38
34 (pars)	,660	,515	2	,015	,485	,798	,363	38

Elastic net cross-validation

Elastic Net Models

Model	Ridge Penalty	LASSO Penalty	Regularization "R Square" (1-Error)	Number of Selected Predictors	Standardized Sum of Coefficients	Apparent Prediction Error	Expected Prediction Error		
							Estimate	Std. Error	N
451 (opt)	0.8	,840	,461	2	,077	,539	,195	,044	38
499 (pars)	0.9	,780	,500	2	,085	,500	,228	,052	38

Elastic net bootstrap

Elastic Net Models

Model	Ridge Penalty	LASSO Penalty	Regularization "R Square" (1-Error)	Number of Selected Predictors	Standardized Sum of Coefficients	Apparent Prediction Error	Expected Prediction Error		
							Estimate	Std. Error	N
158 (opt)	0.3	,080	,956	41	,315	,044	,406	,155	36
548 (pars)	1,0	,740	,771	5	,132	,229	,556	,272	38

Regression models with *beta* coefficients.

Model	48	51	32	51	9	10	11	34	451	499	158	548
**Bladder cancer - women**	ridge optimal cross	ridge selected cross	ridge optimal bootstrap	ridge selected bootstrap	LASSO optimal cross	LASSO selected cross	LASSO optimal bootstrap	LASSO selected bootstrap	elastic optimal cross	elastic selected cross	elastic optimal bootstrap	elastic selected bootstrap
FRUITS TOTAL	-0,033	-0,033	-0,034	-0,033	0,304	0,687	0,688				0,433	0,418
APPLES	0,052	0,052	0,046	0,052	0,099						0,100	
BANANAS	0,036	0,033	0,035	0,033								
GRAPES	0,024	0,023	0,028	0,023								
ORANGES & MANDARINS	0,034	0,036	-0,018	0,036								
ALCOHOLIC BEVERAGES TOTAL	-0,048	-0,048	0,073	-0,048								
BEER	0,086	0,086	0,086	0,086	0,112	0,200	0,197				0,172	0,042
DISTILLED BEVERAGES	-0,059	-0,058	-0,068	-0,058								
WINE	0,033	0,035	-0,031	0,035							-0,002	
COCOA BEANS	0,021	0,021	0,010	0,021							0,011	
COFFEE	0,040	0,041	0,039	0,041	0,012						0,003	
TEA	0,016	0,018	0,019	0,018							-0,024	
REFINED SUGAR & SWEETENERS TOTAL	0,066	0,065	0,073	0,065							0,003	
REFINED SUGAR	0,057	0,057	0,069	0,057								
OILCROPS TOTAL	-0,025	-0,024	-0,025	-0,024								
OLIVES	0,038	0,037	0,047	0,037							0,013	
TREENUTS	-0,037	-0,035	-0,033	-0,035							-0,018	
PLANT OILS TOTAL	-0,037	-0,037	-0,039	-0,037								
OLIVE OIL	-0,024	-0,024	-0,021	-0,024								
SOYBEAN OIL	-0,022	-0,020	0,085	-0,020	0,154						0,075	0,004
SUNFLOWER OIL	-0,040	-0,041	-0,041	-0,041	-0,029	-0,003	-0,002				-0,036	
CEREALS TOTAL	-0,024	-0,021	-0,032	-0,021								
MAIZE	-0,029	-0,029	-0,042	-0,029							-0,043	
RYE	0,008	0,008	-0,020	0,008	0,030	0,067	0,051				0,042	
WHEAT	0,072	0,071	0,096	0,071							0,041	
POTATOES	-0,096	-0,095	-0,107	-0,095	-0,231	-0,067	-0,052				-0,242	-0,162
LEGUMES TOTAL	-0,059	-0,057	-0,055	-0,057	-0,026						-0,076	
VEGETABLES TOTAL	-0,026	-0,026	-0,020	-0,026								
ONIONS	-0,055	-0,052	-0,060	-0,052							-0,033	
TOMATOES	-0,038	-0,037	-0,040	-0,037							-0,020	
SPICES	0,057	0,055	0,047	0,055								
PLANT PROTEIN	-0,056	-0,057	-0,068	-0,057							-0,014	
PLANT FAT	-0,027	-0,024	-0,019	-0,024							-0,014	
MEAT TOTAL	0,013	0,010	0,008	0,010								
BEEF	-0,023	-0,023	-0,021	-0,023								
PORK	0,054	0,053	0,056	0,053							0,016	
POULTRY	-0,024	-0,025	-0,047	-0,025								
MEAT PROTEIN	-0,004	-0,004	0,014	-0,004								
MEAT FAT	-0,028	-0,028	-0,026	-0,028								
BEEF & PORK FAT	-0,027	-0,028	-0,022	-0,028							0,010	
DAIRY TOTAL	-0,034	-0,033	0,041	-0,033	0,070						0,021	
MILK	-0,031	-0,032	-0,032	-0,032								
CHEESE	-0,051	-0,051	-0,058	-0,051	0,025	0,037	0,033				0,110	
DAIRY PROTEIN	0,046	0,045	0,037	0,045								
DAIRY FAT	0,032	0,032	0,036	0,032								
MILK PROTEIN	-0,053	-0,053	-0,057	-0,053							-0,016	
MILK FAT	0,037	0,036	0,038	0,036							0,024	
BUTTER & GHEE	-0,062	-0,058	-0,066	-0,058							-0,014	
EDIBLE OFFALS	0,033	0,033	0,019	0,033							0,013	
FISH & SEAFOOD	-0,064	-0,062	-0,068	-0,062							-0,042	
FISH & SEAFOOD FAT	0,021	0,021	0,029	0,021								
EGGS TOTAL	0,073	0,071	-0,066	0,071	0,018			0,375	0,308	0,331	0,040	
LARD	0,111	0,109	0,120	0,109	0,061	0,035	0,029	0,117	0,110	0,142	0,123	
HONEY	-0,055	-0,054	-0,061	-0,054	-0,078	-0,050	-0,044				-0,133	
ANIMAL PROTEIN	0,024	0,025	0,021	0,025								
ANIMAL FAT	0,038	0,040	0,031	0,040								
ANIMAL FAT & ANIMAL PROTEIN	-0,025	-0,025	-0,026	-0,025								
TOTAL PROTEIN	0,012	0,011	-0,012	0,011								
TOTAL FAT	-0,035	-0,035	-0,031	-0,035	-0,037						-0,002	
TOTAL FAT & TOTAL PROTEIN	-0,050	-0,049	-0,049	-0,049	-0,078						-0,074	
% CA energy	-0,061	-0,059	-0,065	-0,059	-0,164							-0,121
% PC CARB energy	-0,029	-0,032	0,026	-0,032								
% Plant food energy	-0,045	-0,042	-0,042	-0,042							-0,024	
TOTAL ENERGY	0,027	0,028	0,031	0,028								
VEGETABLES & CEREALS	-0,020	-0,022	-0,016	-0,022								
MILK & VEGETABLES	-0,056	-0,057	-0,055	-0,057	-0,008						-0,064	
MILK & VEG. & CEREALS	-0,060	-0,059	-0,066	-0,059							-0,009	
PROTEIN INDEX	-0,013	-0,013	0,040	-0,013	0,007						0,020	
Smoking - women (1990 - 2009)	0,064	0,060	0,065	0,060							0,035	
BMI - women (1990-2008)	-0,040	-0,039	-0,042	-0,039								
Raised cholesterol - women (2008)	0,029	0,026	0,025	0,026							0,015	

Kidney cancer – men

Ridge cross-validation

Ridge Models

Model	Penalty	Regularization "R Square" (1-Error)	Standardized Sum of Coefficients	Apparent Prediction Error	Expected Prediction Error		
					Estimate	Std. Error	N
12 (opt)	,220	,991	,065	,009	,990	,310	25
50 (pars)	,980	,921	,009	,079	1,297	,755	25

Ridge bootstrap

Ridge Models

Model	Penalty	Regularization "R Square" (1-Error)	Standardized Sum of Coefficients	Apparent Prediction Error	Expected Prediction Error		
					Estimate	Std. Error	N
51 (opt & pars)	1,000	,921	,009	,079	,468	,178	28

LASSO cross-validation

LASSO Models

Model	Penalty	Regularization "R Square" (1-Error)	Number of Selected Predictors	Standardized Sum of Coefficients	Apparent Prediction Error	Expected Prediction Error		
						Estimate	Std. Error	N
49 (opt & pars)	,960	,403	2	,014	,597	,252	,085	39

LASSO bootstrap

LASSO Models

Model	Penalty	Regularization "R Square" (1-Error)	Number of Selected Predictors	Standardized Sum of Coefficients	Apparent Prediction Error	Expected Prediction Error		
						Estimate	Std. Error	N
23 (opt)	,440	,737	3	,033	,263	1,023	,547	39
51 (pars)	1,000	,362	2	,012	,638	1,422	,684	39

Elastic net cross-validation

Elastic Net Models

Model	Ridge Penalty	LASSO Penalty	Regularization "R Square" (1-Error)	Number of Selected Predictors	Standardized Sum of Coefficients	Apparent Prediction Error	Expected Prediction Error		
							Estimate	Std. Error	N
49 (opt)	0,0	,960	,403	2	,014	,597	,252	,085	39
408 (pars)	0.7	1,000	,321	2	,050	,679	,267	,089	39

Elastic net bootstrap

Elastic Net Models

Model	Ridge Penalty	LASSO Penalty	Regularization "R Square" (1-Error)	Number of Selected Predictors	Standardized Sum of Coefficients	Apparent Prediction Error	Expected Prediction Error		
							Estimate	Std. Error	N
157 (opt)	0.3	,060	,942	58	1,055	,058	,815	,595	38
561 (pars)	1,0	1,000	,358	2	,053	,642	1,194	,484	39

Regression models with *beta* coefficients.

Model	12	50	51	51	49	49	23	51	49	408	157	561
**Kidney cancer - men**	ridge optimal cross	ridge selected cross	ridge optimal bootstrap	ridge selected bootstrap	LASSO optimal cross	LASSO selected cross	LASSO optimal bootstrap	LASSO selected bootstrap	elastic optimal cross	elastic selected cross	elastic optimal bootstrap	elastic selected bootstrap
FRUITS TOTAL	0,094	-0,061	-0,060	-0,060							-0,175	
APPLES	0,050	0,030	0,030	0,030							0,125	
BANANAS	0,031	0,032	0,031	0,031								
GRAPES	-0,131	-0,045	-0,046	-0,046							-0,061	
ORANGES & MANDARINS	-0,093	0,011	0,013	0,013							0,133	
ALCOHOLIC BEVERAGES TOTAL	0,002	0,057	0,060	0,060							-0,051	
BEER	0,158	0,058	0,059	0,059							0,311	
DISTILLED BEVERAGES	-0,104	-0,062	-0,059	-0,059							0,001	
WINE	0,017	0,019	0,020	0,020							0,053	
COCOA BEANS	-0,126	-0,032	-0,030	-0,030							-0,094	
COFFEE	-0,183	-0,056	-0,052	-0,052								
TEA	-0,017	0,018	0,019	0,019							0,072	
REFINED SUGAR & SWEETENERS TOTAL	-0,018	-0,019	-0,019	-0,019							-0,043	
REFINED SUGAR	0,037	-0,021	-0,022	-0,022								
OILCROPS TOTAL	-0,184	-0,097	-0,093	-0,093			-0,233				-0,141	
OLIVES	-0,052	-0,070	-0,069	-0,069								
TREENUTS	0,069	-0,067	-0,066	-0,066							0,045	
PLANT OILS TOTAL	-0,104	-0,017	-0,014	-0,014								
OLIVE OIL	0,086	0,040	0,041	0,041							0,129	
SOYBEAN OIL	-0,178	0,050	0,053	0,053							0,034	
SUNFLOWER OIL	0,158	0,042	0,041	0,041							0,141	
CEREALS TOTAL	-0,092	0,020	0,021	0,021							0,139	
MAIZE	-0,113	-0,046	-0,049	-0,049							-0,114	
RYE	0,098	0,044	0,046	0,046							0,039	
WHEAT	0,137	0,062	0,060	0,060							0,009	
POTATOES	0,024	0,067	0,066	0,066							0,101	
LEGUMES TOTAL	0,058	0,019	0,019	0,019							0,086	
VEGETABLES TOTAL	-0,247	-0,051	-0,050	-0,050							-0,210	
ONIONS	-0,087	-0,065	-0,062	-0,062							-0,026	
TOMATOES	-0,199	-0,081	-0,082	-0,082	-0,228	-0,228		-0,207	-0,228		-0,160	-0,206
SPICES	0,047	-0,045	-0,045	-0,045								
PLANT PROTEIN	-0,035	-0,018	-0,018	-0,018							-0,187	
PLANT FAT	0,072	0,043	0,045	0,045								
MEAT TOTAL	0,041	0,038	0,036	0,036							0,006	
BEEF	0,093	0,040	0,038	0,038							0,153	
PORK	0,037	0,022	0,025	0,025							-0,053	
POULTRY	-0,069	-0,036	-0,035	-0,035							-0,007	
MEAT PROTEIN	0,130	0,044	0,044	0,044							0,126	
MEAT FAT	0,026	-0,008	-0,008	-0,008								
BEEF & PORK FAT	-0,002	-0,035	-0,035	-0,035								
DAIRY TOTAL	-0,050	-0,040	-0,038	-0,038			0,430			0,208	0,228	
MILK	0,206	0,051	0,046	0,046							0,010	
CHEESE	0,106	0,060	0,061	0,061							-0,073	
DAIRY PROTEIN	0,199	0,032	0,033	0,033							0,005	
DAIRY FAT	0,091	0,039	0,038	0,038							0,025	
MILK PROTEIN	0,008	0,045	0,043	0,043								
MILK FAT	-0,026	-0,044	-0,044	-0,044							-0,133	
BUTTER & GHEE	-0,128	0,045	0,048	0,048							0,053	
EDIBLE OFFALS	-0,069	0,009	0,010	0,010							0,004	
FISH & SEAFOOD	0,066	0,020	0,020	0,020							0,022	
FISH & SEAFOOD FAT	0,071	0,021	0,022	0,022							0,045	
EGGS TOTAL	0,075	0,093	0,092	0,092							0,070	
LARD	0,155	0,063	0,062	0,062							0,121	
HONEY	-0,033	-0,041	-0,041	-0,041							-0,068	
ANIMAL PROTEIN	-0,142	-0,029	-0,026	-0,026							-0,202	
ANIMAL FAT	-0,242	0,021	0,028	0,028							-0,255	
ANIMAL FAT & ANIMAL PROTEIN	0,089	0,046	0,045	0,045							0,132	
TOTAL PROTEIN	0,105	-0,015	-0,011	-0,011								
TOTAL FAT	0,024	0,018	-0,039	-0,039							0,008	
TOTAL FAT & TOTAL PROTEIN	0,136	0,043	0,044	0,044							0,075	
% CA energy	-0,111	-0,022	-0,021	-0,021							-0,019	
% PC CARB energy	0,131	0,039	0,034	0,034								
% Plant food energy	-0,082	-0,056	-0,055	-0,055							-0,015	
TOTAL ENERGY	-0,145	-0,037	-0,032	-0,032			0,197			0,064	-0,117	
VEGETABLES & CEREALS	0,248	-0,045	-0,047	-0,047								
MILK & VEGETABLES	-0,218	-0,045	-0,047	-0,047							-0,229	
MILK & VEG. & CEREALS	-0,205	-0,021	-0,021	-0,021							-0,059	
PROTEIN INDEX	-0,164	-0,018	-0,014	-0,014							-0,072	
Smoking - men (1990-2009)	0,068	0,036	0,036	0,036							0,179	
BMI – men (1990-2008)	0,134	0,065	0,068	0,068	0,123	0,123		0,102	0,123		0,097	0,100
Raised cholesterol - men (2008)	0,086	0,028	0,024	0,024							0,020	

Kidney cancer – women

Ridge cross-validation

Ridge Models

Model	Penalty	Regularization "R Square" (1-Error)	Standardized Sum of Coefficients	Apparent Prediction Error	Expected Prediction Error		
					Estimate	Std. Error	N
22 (opt)	,420	,974	,034	,026	,403	,112	25
51 (pars)	1,000	,945	,007	,055	,468	,131	25

Ridge bootstrap

Ridge Models

Model	Penalty	Regularization "R Square" (1-Error)	Standardized Sum of Coefficients	Apparent Prediction Error	Expected Prediction Error		
					Estimate	Std. Error	N
31 (opt)	,600	,972	,009	,028	,342	,110	29
51 (pars)	1,000	,945	,007	,055	,415	,119	29

LASSO cross-validation

LASSO Models

Model	Penalty	Regularization "R Square" (1-Error)	Number of Selected Predictors	Standardized Sum of Coefficients	Apparent Prediction Error	Expected Prediction Error		
						Estimate	Std. Error	N
9 (opt)	,160	,884	15	,057	,116	,526	,133	28
32 (pars)	,620	,555	4	,021	,445	,650	,200	35

LASSO bootstrap

LASSO Models

Model	Penalty	Regularization "R Square" (1-Error)	Number of Selected Predictors	Standardized Sum of Coefficients	Apparent Prediction Error	Expected Prediction Error		
						Estimate	Std. Error	N
7 (opt)	,120	,915	16	,064	,085	,534	,124	38
3 (pars)	,680	,513	3	,018	,487	,638	,116	38

Elastic net cross-validation

Elastic Net Models

Model	Ridge Penalty	LASSO Penalty	Regularization "R Square" (1-Error)	Number of Selected Predictors	Standardized Sum of Coefficients	Apparent Prediction Error	Expected Prediction Error Estimate	Expected Prediction Error Std. Error	Expected Prediction Error N
120 (opt)	0.2	,340	,797	14	,181	,203	,422	,089	32
339 (pars)	0.6	,640	,632	10	,150	,368	,498	,101	33

Elastic net bootstrap

Elastic Net Models

Model	Ridge Penalty	LASSO Penalty	Regularization "R Square" (1-Error)	Number of Selected Predictors	Standardized Sum of Coefficients	Apparent Prediction Error	Expected Prediction Error Estimate	Expected Prediction Error Std. Error	Expected Prediction Error N
212 (opt)	0.4	,140	,896	37	,461	,104	,382	,081	38
476 (pars)	0.9	,320	,794	31	,335	,206	,442	,113	38

Regression models with *beta* coefficients.

Model	22	51	31	51	9	32	7	35	120	339	212	476
**Kidney cancer - women**	ridge optimal cross	ridge selected cross	ridge optimal bootstrap	ridge selected bootstrap	LASSO optimal cross	LASSO selected cross	LASSO optimal bootstrap	LASSO selected bootstrap	elastic optimal cross	elastic selected cross	elastic optimal bootstrap	elastic selected bootstrap
FRUITS TOTAL	0,009	-0,022	-0,028	-0,022								
APPLES	0,098	0,015	0,019	0,015			-0,019					
BANANAS	-0,093	0,027	-0,048	0,027								
GRAPES	-0,078	-0,032	-0,031	-0,032							-0,014	
ORANGES & MANDARINS	0,039	-0,050	-0,048	-0,050							-0,035	
ALCOHOLIC BEVERAGES TOTAL	-0,084	0,033	0,038	0,033								
BEER	0,252	0,068	0,086	0,068							0,068	0,048
DISTILLED BEVERAGES	0,092	-0,059	0,030	-0,059								
WINE	-0,073	-0,054	-0,055	-0,054							-0,052	
COCOA BEANS	-0,122	-0,030	-0,038	-0,030								
COFFEE	-0,081	-0,032	-0,029	-0,032								
TEA	0,063	0,047	0,057	0,047							0,017	0,015
REFINED SUGAR & SWEETENERS TOTAL	-0,112	-0,062	-0,069	-0,062					0,005	0,015	-0,061	0,050
REFINED SUGAR	-0,018	0,040	-0,018	0,040	0,004		0,020		0,045	0,024	0,057	0,058
OILCROPS TOTAL	-0,067	-0,055	-0,064	-0,055							-0,074	-0,044
OLIVES	0,067	-0,046	-0,045	-0,046							-0,020	-0,037
TREENUTS	0,054	-0,047	0,015	-0,047								-0,030
PLANT OILS TOTAL	-0,106	-0,030	-0,026	-0,030								
OLIVE OIL	0,106	0,007	0,009	0,007								
SOYBEAN OIL	-0,048	0,055	0,067	0,055	0,046		0,068				0,083	0,034
SUNFLOWER OIL	0,080	0,013	0,014	0,013								-0,018
CEREALS TOTAL	-0,025	-0,046	-0,048	-0,046							-0,053	
MAIZE	-0,080	-0,021	-0,041	-0,021	-0,003						-0,012	-0,014
RYE	0,024	0,031	0,029	0,031						0,016	0,017	0,058
WHEAT	0,010	-0,019	0,037	-0,019								
POTATOES	0,110	0,061	0,065	0,061		0,020			0,074	0,065	0,073	0,102
LEGUMES TOTAL	0,030	0,014	0,015	0,014								
VEGETABLES TOTAL	-0,219	-0,069	-0,089	-0,069	-0,182	-0,021	-0,130	-0,012	-0,141	-0,131	-0,156	-0,159
ONIONS	-0,071	-0,063	-0,073	-0,063								
TOMATOES	-0,100	-0,085	-0,098	-0,085	-0,313	-0,356	-0,349	-0,349	-0,307	-0,266	-0,253	-0,256
SPICES	-0,039	-0,043	-0,055	-0,043	-0,087		-0,111				-0,078	-0,002
PLANT PROTEIN	-0,158	-0,030	-0,044	-0,030								
PLANT FAT	-0,132	-0,034	-0,035	-0,034								
MEAT TOTAL	0,028	0,030	0,027	0,030							0,003	0,026
BEEF	0,048	0,026	0,029	0,026								
PORK	-0,053	-0,008	-0,017	-0,008								
POULTRY	0,015	-0,043	-0,051	-0,043								
MEAT PROTEIN	0,072	0,030	0,042	0,030							0,022	0,023
MEAT FAT	0,039	0,028	-0,009	0,028								
BEEF & PORK FAT	-0,016	-0,030	-0,037	-0,030								
DAIRY TOTAL	-0,006	0,032	0,047	0,032							0,023	
MILK	0,045	0,041	0,049	0,041							0,001	
CHEESE	0,003	0,052	0,058	0,052	0,082		0,099		0,039		0,079	0,052
DAIRY PROTEIN	0,066	-0,016	-0,018	-0,016								
DAIRY FAT	0,107	0,021	0,024	0,021								
MILK PROTEIN	0,022	0,044	0,052	0,044	0,002		0,061				0,036	
MILK FAT	-0,128	-0,034	-0,043	-0,034								
BUTTER & GHEE	-0,011	0,039	0,049	0,039	0,047		0,029				0,035	0,021
EDIBLE OFFALS	-0,032	-0,035	-0,042	-0,035								
FISH & SEAFOOD	0,067	0,037	0,046	0,037							0,017	0,015
FISH & SEAFOOD FAT	0,084	0,037	0,049	0,037					0,010		0,027	0,035
EGGS TOTAL	0,049	0,050	0,051	0,050	0,127		0,115		0,043		0,076	0,029
LARD	0,081	0,008	0,010	0,008								
HONEY	-0,082	-0,064	-0,081	-0,064	-0,125		-0,148		-0,061		-0,141	-0,084
ANIMAL PROTEIN	-0,175	0,013	-0,028	0,013								0,006
ANIMAL FAT	-0,215	0,013	0,022	0,013								
ANIMAL FAT & ANIMAL PROTEIN	0,045	0,034	0,044	0,034					0,005		0,042	0,049
TOTAL PROTEIN	0,073	-0,031	-0,046	-0,031								
TOTAL FAT	0,039	-0,025	-0,041	-0,025								
TOTAL FAT & TOTAL PROTEIN	0,086	0,037	0,037	0,037							0,012	
% CA energy	-0,084	-0,033	-0,046	-0,033								
% PC CARB energy	0,053	0,050	0,050	0,050			0,004				0,030	
% Plant food energy	-0,054	-0,037	-0,036	-0,037						-0,001		
TOTAL ENERGY	-0,115	0,055	0,069	0,055	0,189		0,221		0,086	0,010	0,129	0,097
VEGETABLES & CEREALS	0,034	-0,047	-0,061	-0,047	-0,058		-0,085		-0,080	-0,069	-0,102	-0,104
MILK & VEGETABLES	-0,246	-0,029	-0,038	-0,029							-0,019	-0,039
MILK & VEG. & CEREALS	-0,155	-0,027	-0,041	-0,027							-0,009	-0,030
PROTEIN INDEX	-0,123	-0,022	-0,024	-0,022								
Smoking - women (1990 - 2009)	0,057	0,042	0,046	0,042								
BMI - women (1990-2008)	0,027	0,071	0,084	0,071	0,212		0,216		0,089		0,130	0,097
Raised cholesterol - women (2008)	0,039	0,056	0,054	0,056	0,084	0,172	0,092	0,144	0,180	0,137	0,114	0,130

Stomach cancer - men

Ridge cross-validation

Ridge Models

Model	Penalty	Regularization "R Square" (1-Error)	Standardized Sum of Coefficients	Apparent Prediction Error	Expected Prediction Error		
					Estimate	Std. Error	N
32 (opt)	,620	,977	,002	,023	,424	,120	25
51 (pars)	1,000	,959	,002	,041	,530	,140	25

Ridge bootstrap

Ridge Models

Model	Penalty	Regularization "R Square" (1-Error)	Standardized Sum of Coefficients	Apparent Prediction Error	Expected Prediction Error		
					Estimate	Std. Error	N
34 (opt)	,660	,976	,002	,024	,331	,123	28
50 (pars)	,980	,959	,002	,041	,392	,110	28

LASSO cross-validation

LASSO Models

Model	Penalty	Regularization "R Square" (1-Error)	Number of Selected Predictors	Standardized Sum of Coefficients	Apparent Prediction Error	Expected Prediction Error		
						Estimate	Std. Error	N
43 (opt)	,840	,562	4	,009	,438	,532	,076	37
50 (pars)	,980	,482	4	,007	,518	,604	,080	37

LASSO bootstrap

LASSO Models

Model	Penalty	Regularization "R Square" (1-Error)	Number of Selected Predictors	Standardized Sum of Coefficients	Apparent Prediction Error	Expected Prediction Error		
						Estimate	Std. Error	N
19 (opt)	,360	,894	2	,018	,106	,499	,279	39
24 (pars)	,460	,854	2	,017	,146	,604	,245	39

Elastic net cross-validation

Elastic Net Models

Model	Ridge Penalty	LASSO Penalty	Regularization "R Square" (1-Error)	Number of Selected Predictors	Standardized Sum of Coefficients	Apparent Prediction Error	Expected Prediction Error		
							Estimate	Std. Error	N
60 (opt)	0,1	,160	,956	9	,068	,044	,266	,082	35
551 (pars)	1,0	,800	,739	7	,156	,261	,346	,064	37

Elastic net bootstrap

Elastic Net Models

Model	Ridge Penalty	LASSO Penalty	Regularization "R Square" (1-Error)	Number of Selected Predictors	Standardized Sum of Coefficients	Apparent Prediction Error	Expected Prediction Error		
							Estimate	Std. Error	N
282 (opt)	0.5	,520	,790	7	,212	,210	,432	,121	39
558 (pars)	1,0	,940	,675	7	,125	,325	,527	,112	39

Regression models with *beta* coefficients.

Model	32	51	34	50	43	50	19	24	60	551	282	558
**Stomach cancer - men**	ridge optimal cross	ridge selected cross	ridge optimal bootstrap	ridge selected bootstrap	LASSO optimal cross	LASSO selected cross	LASSO optimal bootstrap	LASSO selected bootstrap	elastic optimal cross	elastic selected cross	elastic optimal bootstrap	elastic selected bootstrap
FRUITS TOTAL	0,016	0,010	0,020	0,012								
APPLES	0,083	0,068	0,075	0,066								
BANANAS	-0,038	-0,022	-0,018	-0,025								
GRAPES	-0,051	-0,043	-0,049	-0,047					0,037			
ORANGES & MANDARINS	-0,057	-0,060	-0,071	-0,062	-0,031	-0,005				-0,127	-0,142	-0,106
ALCOHOLIC BEVERAGES TOTAL	-0,046	-0,047	-0,047	-0,040					-0,058			
BEER	-0,032	-0,030	-0,039	-0,034								
DISTILLED BEVERAGES	0,050	0,047	0,048	0,043								
WINE	0,026	0,018	0,020	0,018					-0,085			
COCOA BEANS	-0,022	-0,027	-0,030	-0,026								
COFFEE	-0,055	-0,059	-0,070	-0,058					-0,039	-0,043	-0,076	-0,008
TEA	-0,010	-0,016	-0,020	-0,015								
REFINED SUGAR & SWEETENERS TOTAL	-0,010	-0,014	-0,009	-0,007								
REFINED SUGAR	-0,030	-0,024	-0,030	-0,029								
OILCROPS TOTAL	-0,052	-0,045	-0,052	-0,045								
OLIVES	-0,026	-0,023	-0,023	-0,022								
TREENUTS	-0,029	-0,027	-0,031	-0,030								
PLANT OILS TOTAL	-0,040	-0,044	-0,046	-0,041								
OLIVE OIL	-0,036	-0,037	-0,042	-0,038								
SOYBEAN OIL	0,044	0,030	0,038	0,034								
SUNFLOWER OIL	0,042	0,045	0,049	0,040								
CEREALS TOTAL	-0,044	-0,039	-0,054	-0,044								
MAIZE	0,026	0,021	0,027	0,026					-0,026			
RYE	0,024	0,025	0,023	0,025								
WHEAT	0,035	0,028	0,032	0,028								
POTATOES	0,043	0,041	0,044	0,040								
LEGUMES TOTAL	-0,025	-0,027	-0,033	-0,028								
VEGETABLES TOTAL	0,019	0,015	0,022	0,020								
ONIONS	0,072	0,051	0,052	0,056								
TOMATOES	0,039	0,029	0,037	0,033								
SPICES	-0,051	-0,043	-0,046	-0,045					-0,027			
PLANT PROTEIN	-0,076	-0,059	-0,068	-0,061								
PLANT FAT	-0,038	-0,043	-0,042	-0,037								
MEAT TOTAL	0,009	0,008	0,016	0,009								
BEEF	0,027	0,030	0,035	0,024								
PORK	-0,014	-0,014	-0,009	-0,008								
POULTRY	-0,028	-0,029	-0,028	-0,028								
MEAT PROTEIN	-0,035	-0,028	-0,040	-0,039								
MEAT FAT	-0,052	-0,041	-0,037	-0,039								
BEEF & PORK FAT	-0,044	-0,031	-0,030	-0,033								
DAIRY TOTAL	-0,036	-0,041	-0,050	-0,040								
MILK	0,038	0,037	0,039	0,035								
CHEESE	-0,074	-0,068	-0,064	-0,061	-0,045	-0,009			-0,044	-0,118	-0,148	-0,094
DAIRY PROTEIN	-0,028	-0,023	-0,019	-0,020								
DAIRY FAT	-0,094	-0,079	-0,084	-0,077			-0,344	-0,295	-0,394		-0,052	
MILK PROTEIN	0,040	0,036	0,040	0,036								
MILK FAT	0,031	0,026	0,028	0,024								
BUTTER & GHEE	-0,014	-0,021	-0,021	-0,019								
EDIBLE OFFALS	0,069	0,058	0,063	0,060								
FISH & SEAFOOD	0,018	0,018	0,021	0,017								
FISH & SEAFOOD FAT	-0,033	-0,029	-0,036	-0,034								
EGGS TOTAL	0,016	0,022	0,026	0,020								
LARD	0,046	0,039	0,040	0,039								
HONEY	-0,026	-0,023	-0,024	-0,024								
ANIMAL PROTEIN	-0,021	-0,032	-0,024	-0,021								
ANIMAL FAT	-0,027	-0,039	-0,037	-0,029								
ANIMAL FAT & ANIMAL PROTEIN	-0,037	-0,033	-0,040	-0,045								
TOTAL PROTEIN	-0,027	-0,025	-0,020	-0,020								
TOTAL FAT	0,021	0,027	0,031	0,023						-0,047		-0,028
TOTAL FAT & TOTAL PROTEIN	0,036	0,034	0,043	0,036						-0,036		-0,017
% CA energy	0,073	0,064	0,074	0,075	0,172	0,178				0,195	0,222	0,180
% PC CARB energy	0,063	0,070	0,080	0,069	0,203	0,172				0,210	0,242	0,190
% Plant food energy	0,033	0,022	0,016	0,020								
TOTAL ENERGY	0,051	0,035	0,040	0,040								
VEGETABLES & CEREALS	0,007	0,023	0,023	0,018								
MILK & VEGETABLES	0,020	0,018	0,024	0,022								
MILK & VEG. & CEREALS	-0,045	-0,036	-0,040	-0,036								
PROTEIN INDEX	0,038	0,018	0,024	0,023								
Smoking - men (1990-2009)	0,069	0,064	0,065	0,059			0,632	0,584	0,594		0,033	
BMI – men (1990-2008)	-0,045	-0,038	-0,039	-0,038								
Raised cholesterol - men (2008)	-0,082	-0,057	-0,061	-0,063								

Stomach cancer - women

Ridge cross-validation

Ridge Models

Model	Penalty	Regularization "R Square" (1-Error)	Standardized Sum of Coefficients	Apparent Prediction Error	Expected Prediction Error Estimate	Expected Prediction Error Std. Error	Expected Prediction Error N
15 (opt)	,280	,990	,004	,010	,731	,197	26
48 (pars)	,940	,938	,002	,062	,920	,186	26

Ridge bootstrap

Ridge Models

Model	Penalty	Regularization "R Square" (1-Error)	Standardized Sum of Coefficients	Apparent Prediction Error	Expected Prediction Error Estimate	Expected Prediction Error Std. Error	Expected Prediction Error N
43 (opt)	,840	,945	,002	,055	,539	,186	30
51 (pars)	1,000	,933	,002	,067	,678	,215	29

LASSO cross-validation

LASSO Models

Model	Penalty	Regularization "R Square" (1-Error)	Number of Selected Predictors	Standardized Sum of Coefficients	Apparent Prediction Error	Expected Prediction Error Estimate	Expected Prediction Error Std. Error	Expected Prediction Error N
11 (opt & pars)	,200	,850	11	,024	,150	,465	,128	33

LASSO bootstrap

LASSO Models

Model	Penalty	Regularization "R Square" (1-Error)	Number of Selected Predictors	Standardized Sum of Coefficients	Apparent Prediction Error	Expected Prediction Error Estimate	Expected Prediction Error Std. Error	Expected Prediction Error N
35 (opt)	,680	,582	5	,010	,418	,655	,143	39
51 (pars)	1,000	,413	2	,006	,587	,780	,181	39

Elastic net cross-validation

Elastic Net Models

Model	Ridge Penalty	LASSO Penalty	Regularization "R Square" (1-Error)	Number of Selected Predictors	Standardized Sum of Coefficients	Apparent Prediction Error	Expected Prediction Error		
							Estimate	Std. Error	N
225 (opt)	0.4	,400	,768	11	,222	,232	,426	,106	34
546 (pars)	1,0	,700	,709	11	,161	,291	,521	,117	34

Elastic net bootstrap

Elastic Net Models

Model	Ridge Penalty	LASSO Penalty	Regularization "R Square" (1-Error)	Number of Selected Predictors	Standardized Sum of Coefficients	Apparent Prediction Error	Expected Prediction Error		
							Estimate	Std. Error	N
397 (opt)	0.7	,780	,649	6	,126	,351	,548	,110	39
507 (pars)	0.9	,940	,593	6	,106	,407	,649	,126	39

Regression models with *beta* coefficients.

Model	15	48	43	51	11	11	35	51	225	546	397	507
**Stomach cancer - women**	ridge optimal cross	ridge selected cross	ridge optimal bootstrap	ridge selected bootstrap	LASSO optimal cross	LASSO selected cross	LASSO optimal bootstrap	LASSO selected bootstrap	elastic optimal cross	elastic selected cross	elastic optimal bootstrap	elastic selected bootstrap
FRUITS TOTAL	-0,052	-0,033	-0,035	-0,033								
APPLES	0,070	0,042	0,044	0,041								
BANANAS	0,044	0,022	0,022	0,020								
GRAPES	-0,040	-0,063	-0,064	-0,062								
ORANGES & MANDARINS	-0,072	-0,070	-0,070	-0,069	-0,133	-0,133	-0,052		-0,144	-0,130	-0,112	-0,089
ALCOHOLIC BEVERAGES TOTAL	-0,132	-0,070	-0,074	-0,068	-0,120	-0,120			-0,006			
BEER	-0,054	-0,037	-0,037	-0,036								
DISTILLED BEVERAGES	0,148	0,101	0,106	0,099	0,236	0,236	0,003		0,114	0,035		
WINE	0,056	0,043	0,046	0,043								
COCOA BEANS	-0,046	-0,019	-0,018	-0,019								
COFFEE	-0,093	-0,052	-0,052	-0,050								
TEA	-0,043	-0,024	-0,023	-0,023								
REFINED SUGAR & SWEETENERS TOTAL	-0,057	-0,032	-0,033	-0,031								
REFINED SUGAR	-0,014	-0,035	-0,036	-0,035								
OILCROPS TOTAL	-0,055	-0,041	-0,042	-0,040								
OLIVES	-0,023	-0,018	-0,017	-0,017								
TREENUTS	-0,028	-0,045	-0,046	-0,045								
PLANT OILS TOTAL	-0,037	-0,022	-0,020	-0,021								
OLIVE OIL	0,014	0,011	0,013	0,011								
SOYBEAN OIL	0,010	0,027	0,028	0,027								
SUNFLOWER OIL	0,076	0,058	0,058	0,056	0,047	0,047			0,012			
CEREALS TOTAL	-0,086	-0,052	-0,054	-0,049								
MAIZE	0,003	0,010	0,009	0,010								
RYE	-0,044	-0,024	-0,027	-0,023								
WHEAT	-0,053	-0,037	-0,040	-0,036	0,118	0,118			0,037			
POTATOES	0,053	0,031	0,032	0,031								
LEGUMES TOTAL	-0,063	-0,027	-0,028	-0,026								
VEGETABLES TOTAL	0,043	0,035	0,036	0,034								
ONIONS	-0,075	-0,020	-0,020	-0,019								
TOMATOES	0,069	0,058	0,061	0,056								
SPICES	-0,020	-0,038	-0,039	-0,038								
PLANT PROTEIN	-0,093	-0,061	-0,066	-0,060								
PLANT FAT	-0,069	-0,031	-0,030	-0,029								
MEAT TOTAL	0,014	-0,040	-0,043	-0,041								
BEEF	0,096	-0,016	-0,017	-0,016								
PORK	-0,076	-0,034	-0,036	-0,033								
POULTRY	-0,066	-0,061	-0,063	-0,060								
MEAT PROTEIN	-0,026	-0,046	-0,047	-0,046								
MEAT FAT	0,063	0,034	0,035	0,032								
BEEF & PORK FAT	-0,032	-0,024	-0,025	-0,024								
DAIRY TOTAL	-0,076	-0,036	-0,035	-0,034								
MILK	0,035	0,027	0,028	0,026								
CHEESE	-0,095	-0,063	-0,064	-0,062	-0,096	-0,096	-0,117	-0,045	-0,146	-0,125	-0,113	-0,085
DAIRY PROTEIN	0,013	-0,016	-0,016	-0,016								
DAIRY FAT	-0,100	-0,072	-0,074	-0,071	-0,165	-0,165			-0,073	-0,015		
MILK PROTEIN	0,030	0,027	0,028	0,027								
MILK FAT	0,044	0,028	0,029	0,026								
BUTTER & GHEE	0,020	0,029	0,032	0,029								
EDIBLE OFFALS	0,030	0,032	0,034	0,032								
FISH & SEAFOOD	0,063	0,031	0,032	0,029								
FISH & SEAFOOD FAT	-0,013	-0,042	-0,044	-0,042								
EGGS TOTAL	0,045	0,019	0,020	0,019								
LARD	0,034	0,027	0,028	0,027								
HONEY	0,064	0,045	0,046	0,043	0,006	0,006						
ANIMAL PROTEIN	-0,087	-0,041	-0,041	-0,040					-0,005	-0,009		
ANIMAL FAT	-0,108	-0,044	-0,043	-0,043						-0,009		
ANIMAL FAT & ANIMAL PROTEIN	-0,013	-0,041	-0,043	-0,042						-0,022		
TOTAL PROTEIN	-0,039	-0,021	-0,022	-0,021								
TOTAL FAT	0,058	0,015	0,015	0,014	-0,016	-0,016	-0,014		-0,112	-0,112	-0,094	-0,079
TOTAL FAT & TOTAL PROTEIN	0,005	0,022	0,024	0,022						-0,032	-0,002	
% CA energy	0,025	0,062	0,065	0,063					0,115	0,144	0,130	0,118
% PC CARB energy	0,117	0,077	0,077	0,075	0,288	0,288	0,334	0,274	0,246	0,215	0,204	0,178
% Plant food energy	-0,016	0,021	0,022	0,022								
TOTAL ENERGY	0,049	0,065	0,069	0,064								
VEGETABLES & CEREALS	0,075	0,027	0,027	0,026								
MILK & VEGETABLES	0,040	-0,012	-0,012	-0,011								
MILK & VEG. & CEREALS	0,033	0,054	0,055	0,053								
PROTEIN INDEX	0,005	-0,021	-0,020	-0,020								
Smoking - women (1990 - 2009)	0,054	0,040	0,042	0,040								
BMI - women (1990-2008)	0,046	0,041	0,043	0,041								
Raised cholesterol - women (2008)	0,097	0,047	0,049	0,045	0,028	0,028						

CANCER TOTAL - men

Ridge cross-validation

Ridge Models

Model	Penalty	Regularization "R Square" (1-Error)	Standardized Sum of Coefficients	Apparent Prediction Error	Expected Prediction Error		
					Estimate	Std. Error	N
18 (opt)	,340	,991	,012	,009	,382	,094	25
32 (pars)	,620	,987	,002	,013	,432	,197	25

Ridge bootstrap

Ridge Models

Model	Penalty	Regularization "R Square" (1-Error)	Standardized Sum of Coefficients	Apparent Prediction Error	Expected Prediction Error		
					Estimate	Std. Error	N
43 (opt)	,840	,981	,002	,019	,189	,061	27
51 (pars)	1,000	,976	,002	,024	,232	,083	26

LASSO cross-validation

LASSO Models

Model	Penalty	Regularization "R Square" (1-Error)	Number of Selected Predictors	Standardized Sum of Coefficients	Apparent Prediction Error	Expected Prediction Error		
						Estimate	Std. Error	N
51 (opt & pars)	1,000	,545	3	,009	,455	,206	,106	36

LASSO bootstrap

LASSO Models

Model	Penalty	Regularization "R Square" (1-Error)	Number of Selected Predictors	Standardized Sum of Coefficients	Apparent Prediction Error	Expected Prediction Error		
						Estimate	Std. Error	N
16 (opt)	,300	,920	7	,023	,080	,509	,182	38
46 (pars)	,900	,612	3	,011	,388	,660	,380	38

Elastic net cross-validation

Elastic Net Models

Model	Ridge Penalty	LASSO Penalty	Regularization "R Square" (1-Error)	Number of Selected Predictors	Standardized Sum of Coefficients	Apparent Prediction Error	Expected Prediction Error		
							Estimate	Std. Error	N
51 (opt & pars)	,0	1,000	,545	3	,009	,455	,206	,106	36

Elastic net bootstrap

Elastic Net Models

Model	Ridge Penalty	LASSO Penalty	Regularization "R Square" (1-Error)	Number of Selected Predictors	Standardized Sum of Coefficients	Apparent Prediction Error	Expected Prediction Error		
							Estimate	Std. Error	N
257 (opt)	0,5	,020	,826	61	,564	,174	,449	,128	31
547 (pars)	1,0	,720	,889	11	,255	,111	,511	,244	38

Regression models with *beta* coefficients.

Model	18	32	43	51	51	51	16	46	51	51	257	547
**CANCER TOTAL - men**	ridge optimal cross	ridge selected cross	ridge optimal bootstrap	ridge selected bootstrap	LASSO optimal cross	LASSO selected cross	LASSO optimal bootstrap	LASSO selected bootstrap	elastic optimal cross	elastic selected cross	elastic optimal bootstrap	elastic selected bootstrap
FRUITS TOTAL	-0,046	-0,061	-0,055	-0,051							-0,086	
APPLES	0,041	-0,001	-0,010	-0,009							0,035	
BANANAS	-0,147	-0,025	-0,014	-0,013							-0,020	
GRAPES	-0,072	-0,033	-0,027	-0,025								
ORANGES & MANDARINS	0,046	-0,007	-0,024	-0,021							0,009	
ALCOHOLIC BEVERAGES TOTAL	0,111	0,034	0,046	0,046							0,079	
BEER	0,046	0,038	0,024	0,025							0,041	
DISTILLED BEVERAGES	-0,044	-0,023	-0,026	-0,028							-0,015	
WINE	0,093	0,059	0,041	0,041							0,080	
COCOA BEANS	0,091	0,017	0,025	0,025							0,034	
COFFEE	0,082	-0,008	0,008	0,010							0,002	
TEA	0,051	-0,006	-0,011	-0,010								
REFINED SUGAR & SWEETENERS TOTAL	-0,001	0,017	0,022	0,022							0,018	
REFINED SUGAR	0,015	-0,010	-0,016	-0,015							0,028	
OILCROPS TOTAL	-0,083	-0,094	-0,087	-0,083							-0,150	-0,077
OLIVES	-0,079	-0,085	-0,077	-0,073	-0,114	-0,114	-0,452	-0,145	-0,114	-0,114	-0,136	-0,189
TREENUTS	0,008	0,039	0,026	0,026							0,028	
PLANT OILS TOTAL	0,065	-0,019	0,002	0,002								
OLIVE OIL	0,025	-0,015	-0,022	-0,020							-0,018	
SOYBEAN OIL	0,138	0,045	0,040	0,042							0,089	
SUNFLOWER OIL	-0,043	0,038	0,033	0,029							0,051	
CEREALS TOTAL	0,071	0,022	-0,015	-0,016								
MAIZE	-0,078	0,013	0,021	0,021							0,015	
RYE	0,007	-0,007	-0,008	0,010							0,007	
WHEAT	-0,059	-0,020	-0,013	-0,013								
POTATOES	-0,061	-0,013	-0,015	-0,016								
LEGUMES TOTAL	0,042	-0,012	-0,026	-0,026							-0,016	
VEGETABLES TOTAL	-0,058	-0,043	-0,038	-0,037							-0,061	
ONIONS	0,075	-0,027	-0,036	-0,035							-0,019	
TOMATOES	-0,033	-0,029	-0,032	-0,030							-0,036	
SPICES	-0,024		-0,018	0,004							-0,033	
PLANT PROTEIN	-0,047	-0,045	-0,029	-0,031							-0,060	
PLANT FAT	0,107	0,026	0,019	0,019							-0,008	
MEAT TOTAL	-0,120	0,017	0,022	0,021							0,003	
BEEF	-0,051	0,023	0,015	0,015								
PORK	0,080	0,057	0,066	0,064	0,122	0,122	0,128	0,153	0,122	0,122	0,121	0,165
POULTRY	0,031	0,056	0,054	0,051			0,249				0,066	0,035
MEAT PROTEIN	0,033	0,028	0,012	0,011							0,008	
MEAT FAT	-0,113	0,008	0,026	0,026							0,017	
BEEF & PORK FAT	-0,107	0,007	0,028	0,026							0,006	
DAIRY TOTAL	0,068	-0,008	-0,015	-0,013							0,028	
MILK	-0,010	0,020	0,017	0,016							0,013	
CHEESE	-0,047	-0,041	-0,035	-0,033							-0,075	
DAIRY PROTEIN	-0,046	0,006	0,016	0,016								
DAIRY FAT	-0,071	-0,065	-0,060	-0,056							-0,094	
MILK PROTEIN	-0,012	0,014	-0,006	-0,007							0,014	
MILK FAT	-0,030	-0,022	-0,014	-0,015							-0,014	
BUTTER & GHEE	0,106	0,023	0,014	0,016							0,034	
EDIBLE OFFALS	0,090	0,030	0,022	0,023							0,044	
FISH & SEAFOOD	0,008	-0,003	-0,006	-0,005								
FISH & SEAFOOD FAT	0,016	0,035	0,021	0,020							0,016	
EGGS TOTAL	0,017	0,066	0,061	0,057							0,074	0,128
LARD	0,080	0,088	0,076	0,074			0,039				0,136	0,038
HONEY	-0,094	-0,066	-0,061	-0,058							-0,104	
ANIMAL PROTEIN	0,159	-0,008	0,004	0,007							0,025	
ANIMAL FAT	0,234	0,040	0,031	0,032							0,097	
ANIMAL FAT & ANIMAL PROTEIN	-0,026	0,028	0,012	0,012								
TOTAL PROTEIN	-0,026	0,003	0,014	0,013							-0,032	
TOTAL FAT	-0,166	0,023	0,027	0,026							0,015	
TOTAL FAT & TOTAL PROTEIN	-0,118	0,021	0,036	0,035							0,054	
% CA energy	-0,014	-0,055	-0,029	-0,028							-0,033	
% PC CARB energy	-0,137	0,014	0,026	0,023								
% Plant food energy	0,101	-0,026	-0,051	-0,049			-0,007				-0,061	-0,025
TOTAL ENERGY	0,139	0,016	0,020	0,020							-0,006	
VEGETABLES & CEREALS	-0,163	-0,054	-0,053	-0,053							-0,109	-0,160
MILK & VEGETABLES	-0,051	-0,054	-0,040	-0,039							-0,059	-0,033
MILK & VEG. & CEREALS	-0,091	-0,075	-0,055	-0,054	-0,180	-0,180	-0,149	-0,189	-0,180	-0,180	-0,097	-0,179
PROTEIN INDEX	0,134	-0,006	0,019	0,020							0,039	
Smoking - men (1990-2009)	0,005	-0,059	-0,065	-0,064			-0,010				-0,105	-0,002
BMI – men (1990-2008)	-0,047	0,002	0,013	0,013							0,015	
Raised cholesterol - men (2008)	-0,123	0,038	0,030	0,028							0,013	

CANCER TOTAL - women

Ridge cross-validation

Ridge Models

Model	Penalty	Regularization "R Square" (1-Error)	Standardized Sum of Coefficients	Apparent Prediction Error	Expected Prediction Error		
					Estimate	Std. Error	N
26 (opt)	,500	,992	,002	,008	,209	,054	24
51 (pars)	1,000	,975	,001	,025	,254	,061	24

Ridge bootstrap

Ridge Models

Model	Penalty	Regularization "R Square" (1-Error)	Standardized Sum of Coefficients	Apparent Prediction Error	Expected Prediction Error		
					Estimate	Std. Error	N
42 (opt)	,820	,977	,002	,023	,167	,054	28
51 (pars)	1,000	,975	,001	,025	,172	,058	29

LASSO cross-validation

LASSO Models

Model	Penalty	Regularization "R Square" (1-Error)	Number of Selected Predictors	Standardized Sum of Coefficients	Apparent Prediction Error	Expected Prediction Error		
						Estimate	Std. Error	N
37 (opt)	,720	,623	5	,011	,377	,363	,061	35
50 (pars)	,980	,465	3	,007	,535	,377	,067	35

LASSO bootstrap

LASSO Models

Model	Penalty	Regularization "R Square" (1-Error)	Number of Selected Predictors	Standardized Sum of Coefficients	Apparent Prediction Error	Expected Prediction Error		
						Estimate	Std. Error	N
35 (opt)	,680	,643	5	,011	,357	,828	,359	38
51 (pars)	1,000	,451	3	,007	,549	1,103	,677	38

Elastic net cross-validation

Elastic Net Models

Model	Ridge Penalty	LASSO Penalty	Regularization "R Square" (1-Error)	Number of Selected Predictors	Standardized Sum of Coefficients	Apparent Prediction Error	Expected Prediction Error		
							Estimate	Std. Error	N
156 (opt)	0.3	,040	,950	58	,213	,050	,350	,092	24
554 (pars)	1,0	,860	,722	8	,164	,278	,393	,082	33

Elastic net bootstrap

Elastic Net Models

Model	Ridge Penalty	LASSO Penalty	Regularization "R Square" (1-Error)	Number of Selected Predictors	Standardized Sum of Coefficients	Apparent Prediction Error	Expected Prediction Error		
							Estimate	Std. Error	N
205 (opt)	,400	,000	,903	71	1,000	,097	,441	,117	28
358 (pars)	,700	,000	,669	71	1,000	,331	,446	,084	28

Regression models with *beta* coefficients.

Model	26	51	42	51	37	50	35	51	156	554	205	358
**CANCER TOTAL - women**	ridge optimal cross	ridge selected cross	ridge optimal bootstrap	ridge selected bootstrap	LASSO optimal cross	LASSO selected cross	LASSO optimal bootstrap	LASSO selected bootstrap	elastic optimal cross	elastic selected cross	elastic optimal bootstrap	elastic selected bootstrap
FRUITS TOTAL	-0,038	-0,032	-0,041	-0,032					-0,048		-0,064	-0,063
APPLES	-0,019	-0,019	-0,016	-0,019							-0,023	-0,029
BANANAS	0,048	0,031	0,023	0,031					0,047		0,051	0,053
GRAPES	0,048	0,043	0,054	0,043					0,053		0,083	0,078
ORANGES & MANDARINS	0,050	0,053	0,065	0,053					0,098		0,089	0,097
ALCOHOLIC BEVERAGES TOTAL	0,034	0,024	0,011	0,024					0,031		0,010	0,044
BEER	0,029	0,036	0,052	0,036					0,018		0,070	0,065
DISTILLED BEVERAGES	-0,079	-0,068	-0,067	-0,068					-0,131		-0,096	-0,123
WINE	0,032	0,033	0,032	0,033					0,027		0,031	0,061
COCOA BEANS	-0,030	0,021	0,010	0,021					0,029		-0,073	0,036
COFFEE	0,033	0,039	0,028	0,039					0,048		-0,017	0,063
TEA	0,035	0,034	0,043	0,034					0,044		0,061	0,063
REFINED SUGAR & SWEETENERS TOTAL	0,024	0,017	0,008	0,017					0,019		0,009	0,028
REFINED SUGAR	0,013	0,018	0,037	0,018							0,060	0,031
OILCROPS TOTAL	-0,047	-0,042	-0,045	-0,042							-0,068	-0,078
OLIVES	-0,040	-0,036	-0,041	-0,036					-0,066		-0,067	-0,067
TREENUTS	-0,039	-0,032	-0,028	-0,032					-0,045		-0,039	-0,061
PLANT OILS TOTAL	-0,024	-0,020	-0,032	-0,020					-0,020		-0,054	-0,038
OLIVE OIL	-0,035	-0,030	-0,032	-0,030					-0,045		-0,046	-0,055
SOYBEAN OIL	0,060	0,057	0,043	0,057	0,155	0,090	0,162	0,085	0,099	0,151	0,056	0,100
SUNFLOWER OIL	-0,032	-0,029	-0,024	-0,029							-0,022	-0,047
CEREALS TOTAL	-0,035	-0,019	0,010	-0,019					-0,026		0,008	-0,029
MAIZE	0,021	0,012	0,010	0,012							0,016	0,022
RYE	-0,028	-0,024	-0,021	-0,024					-0,013		-0,034	-0,044
WHEAT	0,055	0,043	0,048	0,043					0,063		0,077	0,083
POTATOES	-0,062	-0,050	-0,045	-0,050					-0,089		-0,078	-0,093
LEGUMES TOTAL	0,020	0,026	0,037	0,026							0,056	0,050
VEGETABLES TOTAL	-0,042	-0,040	-0,055	-0,040					-0,036		-0,087	-0,076
ONIONS	-0,065	-0,050	-0,060	-0,050					-0,095		-0,116	-0,092
TOMATOES	-0,055	-0,049	-0,054	-0,049					-0,085		-0,080	-0,093
SPICES	-0,027	0,016	0,023	0,016							-0,073	-0,051
PLANT PROTEIN	-0,031	-0,042	-0,054	-0,042					-0,036		-0,072	-0,074
PLANT FAT	-0,015	-0,016	-0,033	-0,016							-0,061	-0,030
MEAT TOTAL	-0,012	-0,011	0,029	-0,011					-0,008		0,073	-0,018
BEEF	0,027	0,026	0,056	0,026					0,007		0,097	0,047
PORK	-0,011	-0,013	-0,030	-0,013					0,036		-0,052	-0,022
POULTRY	0,035	0,027	0,038	0,027					0,019		0,071	0,050
MEAT PROTEIN	-0,011	-0,004	0,050	-0,004							0,080	-0,005
MEAT FAT	0,051	0,033	0,018	0,033					0,052		0,031	0,057
BEEF & PORK FAT	0,052	0,035	0,026	0,035					0,038		0,050	0,063
DAIRY TOTAL	-0,030	-0,020	-0,022	-0,020					0,006		-0,049	-0,032
MILK	0,018	-0,010	-0,009	-0,010					0,002		0,056	-0,009
CHEESE	-0,039	-0,036	-0,047	-0,036					-0,042		-0,074	-0,070
DAIRY PROTEIN	-0,023	0,014	0,003	0,014					-0,015		0,002	-0,037
DAIRY FAT	-0,034	-0,028	-0,029	-0,028					-0,043		-0,044	-0,049
MILK PROTEIN	-0,036	-0,040	-0,043	-0,040					-0,046		-0,064	-0,065
MILK FAT	-0,050	-0,051	-0,053	-0,051					-0,080		-0,076	-0,086
BUTTER & GHEE	0,015	0,018	-0,009	0,018					0,012		-0,028	0,027
EDIBLE OFFALS	0,034	0,036	0,042	0,036					0,042		0,060	0,064
FISH & SEAFOOD	-0,035	-0,023	-0,011	-0,023					-0,026		-0,010	-0,048
FISH & SEAFOOD FAT	0,033	0,031	0,047	0,031					0,004		0,069	0,058
EGGS TOTAL	0,027	0,026	0,038	0,026							0,075	0,052
LARD	0,078	0,070	0,088	0,070					0,142		0,141	0,131
HONEY	-0,048	-0,039	-0,035	-0,039					-0,064		-0,056	-0,070
ANIMAL PROTEIN	0,026	0,019	-0,028	0,019					0,021		-0,082	0,030
ANIMAL FAT	0,036	0,034	-0,004	0,034	0,055		0,060		0,072	0,084	-0,019	0,058
ANIMAL FAT & ANIMAL PROTEIN	0,022	0,026	0,071	0,026							0,132	0,049
TOTAL PROTEIN	-0,031	-0,034	-0,044	-0,034					-0,033		-0,069	-0,054
TOTAL FAT	0,035	0,025	0,028	0,025					0,012		0,047	0,045
TOTAL FAT & TOTAL PROTEIN	0,036	0,022	-0,021	0,022					0,037		-0,025	0,038
% CA energy	-0,025	-0,033	-0,077	-0,033							-0,141	-0,059
% PC CARB energy	-0,015	-0,031	0,009	-0,031					-0,018		0,028	-0,051
% Plant food energy	-0,059	-0,037	-0,023	-0,037	-0,003		-0,008		-0,094	-0,087	-0,046	-0,067
TOTAL ENERGY	-0,018	-0,013	-0,039	-0,013							-0,085	-0,027
VEGETABLES & CEREALS	-0,050	-0,045	-0,025	-0,045					-0,108	-0,117	-0,022	-0,077
MILK & VEGETABLES	-0,043	-0,045	-0,055	-0,045					-0,035	-0,022	-0,087	-0,076
MILK & VEG. & CEREALS	-0,050	-0,054	-0,071	-0,054	-0,274	-0,233	-0,280	-0,229	-0,075	-0,167	-0,109	-0,094
PROTEIN INDEX	0,014	0,012	0,010	0,012					0,009		0,007	0,029
Smoking - women (1990 - 2009)	0,042	0,040	0,048	0,040					0,068		0,099	0,071
BMI - women (1990-2008)	-0,029	-0,024	-0,026	-0,024					-0,011		-0,044	-0,045
Raised cholesterol - women (2008)	0,065	0,050	0,059	0,050	0,065	0,042	0,070	0,037	0,093	0,104	0,113	0,091
